# Adaptive Synthesis, Supramolecular Behavior, and Biological Properties of Amphiphilic Carbosilane-Phosphonium Dendrons with Tunable Structure

**DOI:** 10.1021/acs.biomac.4c01092

**Published:** 2024-11-11

**Authors:** Antonín Edr, Dominika Wrobel, Alena Krupková, Lucie Červenková Št′astná, Evgeny Apartsin, Michaela Hympánová, Jan Marek, Jan Malý, Marek Malý, Tomáš Strašák

**Affiliations:** † The Czech Academy of Sciences, Institute of Chemical Process Fundamentals, 165 02 Prague, Czech Republic; ‡ Centre for Nanomaterials and Biotechnology Faculty of Science, Jan Evangelista Purkyně University in Ústí nad Labem, Pasteurova 3632/15, 400 96 Ústí nad Labem, Czech Republic; § Department of Physics, University of Jan Evangelista Purkyně in Ústí nad Labem, 400 96 Ústí nad Labem, Czech Republic; ∥ Biomedical Research Centre, University Hospital Hradec Králové, Sokolská 581, 500 05 Hradec Králové, Czech Republic; ⊥ Université Bordeaux, CNRS, Bordeaux INP, CBMN, UMR 5248, F-33600 Pessac, France; # Department of Epidemiology, Military Faculty of Medicine, University of Defence, Třebešská 1575, 500 05 Hradec Králové, Czech Republic

## Abstract

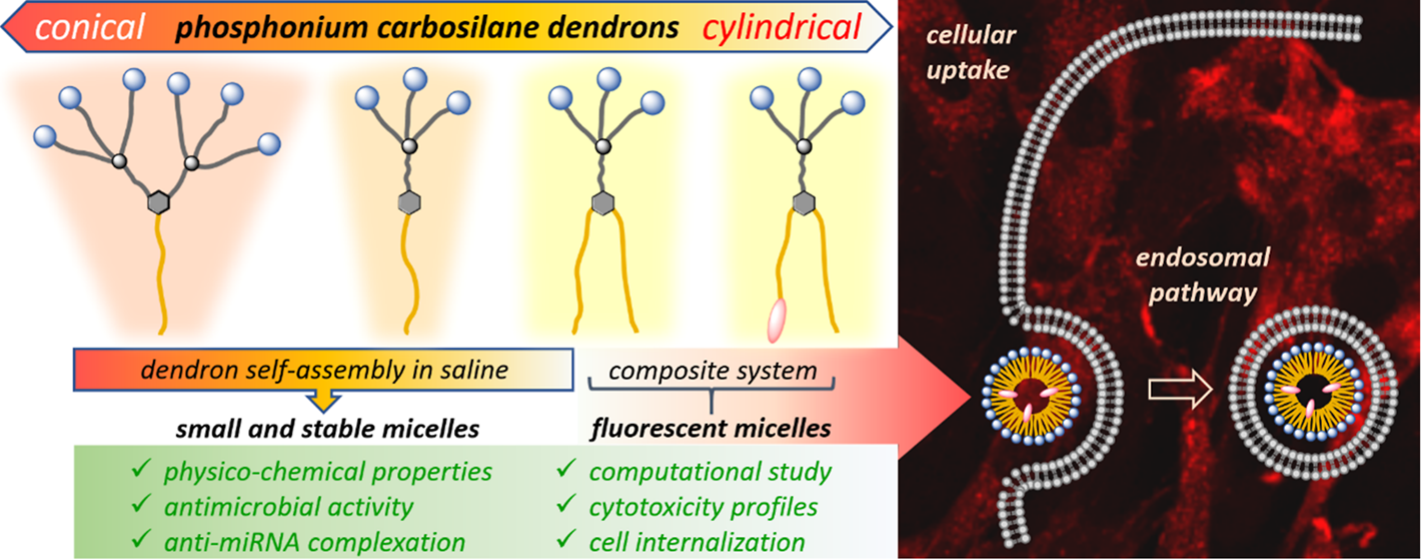

Here, we present a modular synthesis as well as physicochemical and biological evaluation of a new series of amphiphilic dendrons carrying triphenylphosphonium groups at their periphery. Within the series, the size and mutual balance of lipophilic and hydrophilic domains are systematically varied, changing the dendron shape from cylindrical to conical. In physiological solution, the dendrons exhibit very low critical micelle concentrations (2.6–4.9 μM) and form stable and uniform micelles 6–12 nm in diameter, depending on dendron shape; the results correlate well with molecular dynamics simulations. The compounds show relatively high cytotoxicity (IC_50_ 1.2–21.0 μM) associated with micelle formation and inversely related to the size of assembled particles. Depending on their shape, the dendrons show promising results in terms of dendriplex formation and antibacterial activity. In addition to simple amphiphilic dendrons, a fluorescently labeled analogue was also prepared and utilized as an additive visualizing the dendron’s cellular uptake.

## 1 Introduction

Self-assembled nano systems present a rapidly developing technology aiming at delivery of therapeutics. They can act as nonviral vectors for nucleic acids and ensure drug targeting, thus significantly improving its efficiency and reducing side effects; they can also serve as theranostics, combining the drug load with imaging moieties.^1,2^ Among other types, amphiphilic dendrons (amp-DDNs) can be beneficially used as such nano systems.^3^ Self-assembled into various vesicles, they lose some of the benefits of monodispersity compared to dendrimers, but they are still chemically unequivocal due to precisely controlled synthesis which also allows manipulation of the structure of dendrons in many ways. The variation of their scaffold can offer tailored, highly ordered supramolecular systems, which have already been widely utilized in biomimicking, drug delivery, and bioimaging.^4,5^ Their potential in combating multiresistant bacteria has also been recently recognized, as they are able to efficiently mimic the function of antimicrobial proteins while showing higher proteolytic stability.

As the structure of amp-DDNs is generally more complex compared to that of dendrimers, with a lower degree of symmetry and at least two chemically distinct domains, synthetic approaches aiming at dendrons also mostly show a higher level of complexity, employing a broader arsenal of chemical transformations. Their branched (dendritic) domain is nevertheless mostly structurally derived from the corresponding dendrimers.^3,6^ For example, poly(amidoamine)-based amp-DDNs were developed as building blocks of nanoassemblies for the delivery of doxorubicin and siRNA into cancer cells.^7,8^ Besides this, the hydrophobic domain incorporated in their structure was recognized as a prerequisite of their antimicrobial activity.^9^ Various dendrons stemming from the structure of phosphorus dendrimers were reported;^10,11^ recently, amp-DDNs of this type decorated with protonated cyclic amines and incorporating a fluorescent moiety were tested as theranostics with an antiproliferative activity.^12^ Many types of amp-DDNs also beneficially use silicon as a flexible and adaptive branching point, typically combining simple carbosilane dendritic wedges with a charged periphery and a long hydrophobic tail attached in their focal point.^13−16^ These materials demonstrated their ability to form complexes with therapeutic biomacromolecules, including siRNA, and showed a high loading capacity for lipophilic drugs.^17,18^

Amp-DDNs typically host hydrophilic groups at the periphery of their branched domains. These are then consequently displayed on the surface of the assembled particles, and thus, their design is a crucial factor defining the suitability of a particular dendritic structure for a specific application. Cationic nanoparticles are able to interact via electrostatic interactions with negatively charged cell membranes, nucleic acids, and proteins. Up to now, most of the studied systems of this type were surface modified by quaternary ammonium groups and other nitrogen-containing cations. Phosphonium groups appear as attractive alternatives due to their higher thermal stability^19^ and observed lower cytotoxicity compared to ammonium analogues,^20−23^ possibly originating from the differences in interactions with lipid membranes.^24^ However, as we already observed on a series of carbosilane dendrimers with aliphatic and aromatic phosphonium groups at the periphery, the cytotoxicity strongly depends on the substitution of the cationic center.^21,25^ Triarylphosphonium moieties combine the charge with the high lipophilicity of aromatic substituents and represent a unique “delocalized lipophilic charge” with the ability to facilitate the internalization of nanoparticles and transfection of nucleic acids. Triphenylphosphonium (TPP) cation was also recognized as a mitochondrion-specific targeting moiety,^26−29^ which can be utilized in cancer therapy^26,30^ as well as treatment of diabetes or neurodegenerative diseases.^31^ Beside this, phosphonium-functionalized materials constitute a promising class of antibacterial agents with a potential against multidrug-resistant bacteria.^32−34^

The self-assembly pattern of amphiphiles including dendrons is controlled by structural parameters, such as size, shape, and flexibility of both domains, pH, and surface chemistry.^4,15^ As the self-assembly behavior in turn affects the performance, fine-tuning of amphiphiles’ structural features is a key to optimizing their properties to a specific task. To achieve this, the variability of the employed synthetic approach is a big advantage. Building on versatile synthetic methodology introduced in our previous work,^35,36^ we present the preparation of novel library of silicon-branched amp-DDNs with TPP peripheral groups and systematically varying selected structural parameters. In addition, we have prepared a derivative of the parent structure labeled with a fluorescent probe to enable the tracking of the formed assemblies in living cells. So far, these dendrons have no structural analogues among dendrimers. Unlike typical silicon-based dendrons,^13,17^ which rely solely on silicon atoms as branching points, our structures use aromatic core as the second branching motive, introducing more polar regions into the dendritic scaffold. All reported amp-DDNs readily self-assembled into stable nanoscale micelles with a structure-correlated mean size and critical micelle concentration (CMC). As their prospective field of application is very broad, we performed a screening of their biological properties including cell internalization, cytotoxicity, and antimicrobial activity as well as interaction with nucleic acids; a separate study aiming at drug encapsulation and delivery is currently underway. The results imply promising potential of the presented compounds, which can be further optimized for a particular task on the basis of observed structure–activity correlations.

## 2 Experimental Part

### 2.1 General Remarks to Synthesis and Characterization

All of the commercially available chemicals were used without further purification. Chemicals were purchased from the following distributors: thioglycolic acid from TCI Chemicals; THF-*d*_8_, octadecan-1-amine, and 2,2-dimethoxy-2-phenylacetophenone (DMPA) from Acros Organics; palladium on active carbon from Apollo Scientific; TBTU from Carbosynth; BODIPY 630/650 amine from Lumiprobe; 2-mercaptoacetic acid, 4-bromopropylammonium bromide, Amberlite IRA-400 (can be substituted with IRA-402), and CDI from Merck; and CDCl_3_ and DMSO-*d*_6_ from VWR International. All other chemicals were from laboratory stock. THF was purified by distillation from sodium and benzophenone under argon. Experiments in an inert atmosphere were performed using a standard septum technique. TLC was carried out with Sigma-Aldrich TLC Silica gel 60 F254, and spots were detected by UV lamp 254 nm or visualized with KMnO_4_ solution (H_2_O/K_2_CO_3_/NaOH). Organic solvent nanofiltration (OSN) was carried out using solvent-resistant stirred cell (Millipore) equipped with 1 kDa MWCO-regenerated cellulose ultrafiltration discs (Ultracel, Millipore, Merck KGaA, Darmstadt, Germany) and PTFE-encapsulated O-rings (ERIKS), with nitrogen as a driving gas.^37^ Separated mixture was quantitatively transferred into the cell, diluted with the solvent to a total volume of 10–20 mL, and passed through the membrane until approximately 1/10 of the initial volume was left. The cycle was repeated, until sufficient purity was reached. Removal of solvents was performed on a rotary evaporator at a maximum of 50 °C, if not stated otherwise.

#### 2.1.1 NMR Spectra

NMR spectra were measured on a Bruker AVANCE 400 spectrometer (^1^H at 400.1 MHz; ^13^C{^1^H} at 100.6 MHz; ^19^F at 376.4 MHz, ^29^Si{^1^H} gated decoupling at 79.5 MHz) at 25 °C. ^1^H and ^13^C NMR signals were assigned to the corresponding atoms utilizing HSQC, COSY, and HMBC 2D NMR correlation spectra. ^1^H and ^13^C chemical shifts (δ/ppm) are given relative to solvent signals (δ_H_/δ_C_: DMSO-*d*_6_ 2.50/39.52, CDCl_3_ 7.26/77.16, MeOH-*d*_4_ 3.31/49.00, THF-*d*_8_ 3.58/137.86); ^29^Si spectra were referenced to external standard hexamethyldisilane (δ/ppm; −19.87 ppm). The ^19^F NMR spectra were referenced to the external standard hexafluorobenzene (δ/ppm; −163.86). CDCl_3_ and DMSO-*d*_6_ were dried over molecular sieves. THF-*d*_8_ and MeOH-*d*_4_ were used as received.

#### 2.1.2 HRMS Spectra

HRMS spectra were measured on a MicroTOF III spectrometer (Bruker) in the range of *m*/*z* 80–3000 Da. For ionization, APCI in positive mode or ESI in positive or negative mode was used with nitrogen as a nebulizer and dry gas. For calibration of accurate masses, an ESI–APCI Low Concentration Tuning Mix (Agilent) was used.

#### 2.1.3 Emission Spectra and Quantum Yields

Emission spectra and quantum yields (QYs) were measured using a Jasco FP-8300 spectrofluorometer additionally equipped with an ILF-835 100 mm diameter integrating sphere accessory for quantification. All measurements were conducted at room temperature in a standard 10 mm quartz cuvette. Light sources (Jasco ESC-842, ESC-843) were utilized to calibrate the instrument for measuring QYs. Data acquisition took place under a nitrogen atmosphere (purity 99.999%) with the following setup: excitation and emission bandwidths of 5 nm, a scanning speed of 50 nm/min, and a data interval of 0.2 nm. Samples were measured at concentrations of 10^–6^ M in degassed demineralized water, with degassing achieved via the freeze–pump–thaw method. Absolute QYs for the red emission were determined by using an excitation wavelength of 600 nm. For each sample, the incident light and fluorescence intensities (under direct and indirect excitation) were recorded. QYs were calculated using Jasco’s Quantum Yield Calculation Program (FWQE-880), and the values were corrected for indirect excitation.

#### 2.1.4 Thiol–Ene Coupling: Preparation of Dendritic Carboxylic Acids **2a**, **2b**, **5a**, **5b**, **8a**, **8b**, and **14**

Thiol–ene coupling (TEC) was carried out in thin-wall vials; the reaction mixtures were irradiated by a 400 W mercury lamp through a PYREX filter (main transmitted wavelength 350 nm). Allyl-terminated dendrons **1a**, **1b**, **4a**, **4b**, **7a**, **7b**, and **13** were each mixed with 2-mercaptoacetic acid (3 equiv per double bond) and DMPA (0.033 equiv per double bond) in distilled THF (1 mL per 60 mg of **1a**, **1b**, **4a**, or **4b**; 1 mL per 30 mg of **7a**, **7b**, or **13**) and deoxygenated by argon. The reaction mixture was stirred in an argon atmosphere and irradiated for 15 min. DCM was added to the reaction mixture and the solution was washed three times by water and dried by anhydrous MgSO_4_. In the case of the most lipophilic dendron **2b**, Et_2_O was used instead of DCM. All dendrons **2a, 2b, 5a, 5b, 8a, 8b**, and **14** were obtained after removal of the solvents as either colorless or yellowish viscous substances in almost quantitative yields.

#### 2.1.5 Symmetrical amp-DDNs **3a**, **3b**, **6a**, **6b**, **9a**, and **9b**

Dendritic carboxylic acids **2a, 2b, 5a, 5b, 8a**, and **8b** were each mixed with (3-aminopropyl)triphenylphosphonium bromide hydrobromide (1.5 equiv per carboxyl group), dissolved in dry DMF (1 mL per 40 mg of **2a**, **2b**, **5a**, or **5b**; 1 mL per 30 mg of **8a** or **8b**) in argon atmosphere and stirred for 5 min. TBTU (1.2 equiv per carboxyl group) in dry DMF (1 mL per 40 mg of **2a**, **2b**, **5a**, or **5b**; 1 mL per 30 mg of **8a** or **8b**) was slowly added and the solution was stirred for another 15 min. DIPEA (2.5 equiv per carboxyl group) was added, and the reaction mixture was heated to 40 °C for 4 h. Then, the volume of solvent was reduced and excess reagents were removed by OSN. MeOH was used as a solvent for dendrons **3a**, **6b**, **9a**, and **9b**; dendrons **3b** and **6a** were filtered in a 1:1 mixture of MeOH and DCM. The retentate was applied to a column of ion-exchange resin Amberlite IRA-400 in Cl^–^ cycle and let to flow slowly through. In the case of MeOH/DCM solvent mixtures, the retentate was evaporated to dryness and redissolved in pure MeOH prior to ion exchange. The product was washed from the column by additional MeOH, the volume of solvent was reduced, and the procedure was repeated once. Finally, the solvent was removed to afford symmetrical dendrons **3a**, **3b**, **6a**, **6b**, **9a**, and **9b** as white foams in yields of 80–87%.

Detailed synthetic procedures and characterization data of all new compounds can be found in the Supporting Information.

### 2.2 Critical Micelle Concentration

Critical micelle concentrations (CMCs) were determined in Dulbecco’s phosphate-buffered saline using a fluorescence method based on significant difference between Nile Red fluorescence intensity in a hydrophilic aqueous environment and in a hydrophobic environment of a micelle core.^38,39^ Fluorescence was measured on a Horiba FluoroMax-4 fluorometer at an excitation wavelength of 550 nm and an emission wavelength always at the maximum intensity (625–635 nm). Dendron solutions were prepared by stepwise 2-fold dilution of 1000 μM starting solution giving samples of 15 different concentrations in total, with a minimum concentration of 0.06 μM. The concentration of Nile Red was always 0.88 mg/L. The CMC values were calculated from the general equations of the two trendlines of linear regions as their intersection (Figures S1–S6).

### 2.3 Dynamic Light Scattering

The size of micelles and micellar aggregates was estimated by the dynamic light scattering (DLS) method using NanoBrook OMNI ZetaPALS (Brookhaven Instruments, USA) with a detection angle 90° and 640 nm laser. The presented diameters are mean values from the intensity-weighted size distribution. Representative results from day 1 and day 75 are shown in Figures S7–S12. The composition of the samples was as follows: dendron (1.0 mM) and NaCl (0.9%) in deionized water. The solutions were sonicated for 30 min, filtered through 0.45 μm Nylon syringe filters (Avantor, USA), placed in standard polystyrene cuvettes (BI-SCP, Brookhaven Instruments), and stored at 4 °C. All measurements were conducted at 25 °C. Measurement time was set to 5 min, and each measurement was repeated 5 times. The analysis of data was performed using Brookhaven Instruments software Particle Solutions v3.6.07136.

MADLS and size dependence on concentration and pH were measured on Zetasizer Ultra Red (Malvern Panalytical, UK) equipped with a 632.8 nm laser, applying detection angles 12.78°, 90°, and 174.7° in the case of MADLS measurements and 12.78° for size dependence measurements. MADLS results are shown in Figures S13 and S14. MADLS and concentration dependence were measured in DTS0012 polystyrene cuvettes (Malvern Panalytical, UK); pH dependence was studied by acidobasic titration in DTS1070 folded capillary Zeta cell, using MPT-3 Multipurpose Titrator and DEG0003 Auto Degasser (all Malvern Panalytical, UK). The concentration dependence measurement started at 1.0 mM and the samples were diluted 2-fold until the minimum concentration 0.06 mM. For each concentration, the measurement was repeated 3 times giving 15 measurements in total. The pH dependence measurement started at pH 12; the titration was performed with a step of 2 and at each step, the measurement was repeated 3 times, giving 18 measurements in total. Malvern Panalytical software ZS Xplorer version 3.3.1.5 was used for the analysis of data.

Dendriplexes were characterized by DLS using Zetasizer Nano-S (Malvern Panalytical, UK) equipped with a 632.8 nm laser, applying detection angle 173°. Mean diameters from the intensity-weighted size distribution are given. Selected results are shown in Figures S24–S33. Average results from three consecutive sets of measurements generated by Malvern Zetasizer Software version 7.13 are presented. The result of a particular measurement was considered reliable if (1) three consecutive sets of measurements gave similar results (mean diameter ±10%) and (2) the correlogram of the measurement showed fluent flow.^40,41^

### 2.4 Oligonucleotide Complexation

Amphiphilic dendrons (1.5–73 μM) were mixed with the anti-miR-21:5′-m(UCAACAUCAGUCUGAUAAGCUA) (500 nM) in 0.9% NaCl solution in milli-Q water. The dendrons’ concentrations were chosen to attain the charge ratio (CR, the ratio of cations over anions in the sample) of 1.2, 2.5, 5, 10, or 20. The samples were incubated for 15 min at rt prior to measurements.

### 2.5 Molecular Modeling

Three-dimensional computer models of dendron structures were created by using the dendrimer builder, as implemented in the Materials Studio software package from BIOVIA, San Diego, CA, USA (formerly Accelrys). General amber force field^42^ was used for parametrization of all dendrons. The RESP technique^43^ was used to calculate partial charges of dendrons. The pmemd.cuda module^44^ from Amber20 package^45^ was used for molecular dynamics simulations (explicit water, *NPT*, *P* = 1 bar, *T* = 295 K (in homogeneous cases also 310 and 320 K), length of simulation 400 ns). All technical details regarding simulations can be found in the Supporting Information and in the literature.^35,42−54^

### 2.6 Cell Culture

Human foreskin fibroblast (BJ), breast cancer (MCF7), and lung carcinoma (NCI-H460) cell lines were cultured in high glucose DMEM. The glioblastoma (U373) cell line was cultured in RPMI medium. All media were supplemented with 10% (v/v) fetal bovine serum, 4 mM glutamine, 0.1% (w/v) penicillin, and 0.1% (w/v) streptomycin. Medium for the U373 cell line was also supplemented with 5% of nonessential amino acid solution (NEAA). Cells were maintained at 37 °C in humidified atmosphere with 5% CO_2_/95% air and passaged every 3–4 days. Finally, cells were harvested at 80–90% confluence, and their viability was determined using trypan blue exclusion. Subsequently, cells were suspended at a concentration of 1.0 × 10^5^ cells/mL and seeded in 96-well plates for adherence.

### 2.7 MTT Assay

Cytotoxicity assessment of amp-DDNs was conducted using the MTT (3-(4,5-dimethylthiazol-2-yl)-2,5-diphenyltetrazolium bromide) assay. Following a 48 h incubation period to facilitate cell adherence, cells were exposed to dendrons across a concentration range of 0.01 to 100 μM. After an additional 24 h incubation, MTT solution in PBS was added to each well. Four hours later, the medium was removed, and the formazan precipitate was dissolved in dimethyl sulfoxide (DMSO) for absorbance measurements at 580 nm with a reference at 700 nm. Cell viability is graphically represented as a percentage of the control values (without dendrons).

### 2.8 Confocal Fluorescence Microscopy

The interaction of amphiphilic dendrons with mammalian cells, their cellular internalization, and localization were investigated by confocal fluorescence microscopy. Briefly, the cells were seeded in a sterile nunc 24-well plate at the densities of 5.0 × 10^4^ cells/well for the HTB177, MCF7, and BJ cell lines and 7.0 × 10^3^ cells/well for the U373 cell line and cultured for 48 h. The cells were later treated with the mixture of two investigated amp-DDNs (mixture of DnP_3_-2C_18_ (**3b**) with 5% of DnP_3_-1C_18_/BDP (**17**) (DnP_3_-2C_18_@DnP_3_-1C_18_/BDP)) in a final concentration of 5 μM and incubated for 6 h at 37 °C in a humidified atmosphere containing 5.0% of CO_2_. After double washing the cells with PBS, fresh complete DMEM was added to the wells and cells were then placed inside the microscope observation chamber at 37 °C in humidified atmosphere containing 5.0% of CO_2_.

Fluorescence images were acquired by using a Leica CLSM SP8 microscope equipped with an HC PL FLUOTAR 10*x*/0.30 DRY objective. A sequential scan mode was employed to capture the red channel with 633 nm excitation and 640–695 nm emission wavelengths, alongside bright-field images of the same area. The acquired images were processed using Corel Photo-PAINT software (version 2017).

### 2.9 Evaluation of Antimicrobial Properties

In vitro antibacterial activity of the prepared amp-DDNs (DnP_6_-1C_12_, DnP_3_-1C_12_, DnP_3_-2C_12_, and DnP_3_-2C_18_) was tested on a panel of four G+ (*Staphylococcus aureus* CCM 4516, methicillin-resistant *S. aureus* clinical isolate C1923, *Staphylococcus epidermidis* clinical isolate C1936, and vancomycin-resistant *Enterococcus faecium* clinical isolate S2484) and four G-bacterial strains (*Escherichia coli* K12, CCM 7929, *E. coli* clinical isolate A1235, extended-spectrum β-lactamase-producing (ESBL+) *Klebsiella pneumoniae* clinical isolate C1914, and multiresistant *Pseudomonas aeruginosa* clinical isolate A1245). CCM collection strains were obtained from the Czech Collection of Microorganisms. All clinical isolates were obtained during routine procedures in the clinical laboratory at the University Hospital Hradec Králové. All strains were stored at −70 °C in the Cryobank according to the manufacturer’s instructions. Subsequently, when needed, each bacterial strain was inoculated and cultivated on Mueller–Hinton agar (HiMedia, Cadersky-Envitek, Prague, Czech Republic).

The antibacterial susceptibility was determined by a microdilution broth method according to standard M07-A10,^55^ and the optimized protocol has been published previously.^56,57^ Briefly, Mueller–Hinton broth (MHB) adjusted to pH 7.4 (±0.2) was used as the test medium. DMSO served as a diluent for compounds, and its final concentration did not exceed 1% in the test medium. The wells of the microdilution tray contained 200 μL of the MHB with 2-fold serial dilutions of the compounds and were inoculated with 10 μL of the bacterial suspension. The bacterial suspensions were controlled densitometrically to reach 1.5 × 10^8^ viable CFU per 1 mL. The minimum inhibitory concentration (MIC) values, defined as 95% inhibition of bacterial growth, were determined after 24 h of incubation at 35 °C ± 1 °C.

## 3 Results and Discussion

### 3.1 Synthesis and Characterization of Phosphonium Dendrons

**Scheme 1:**
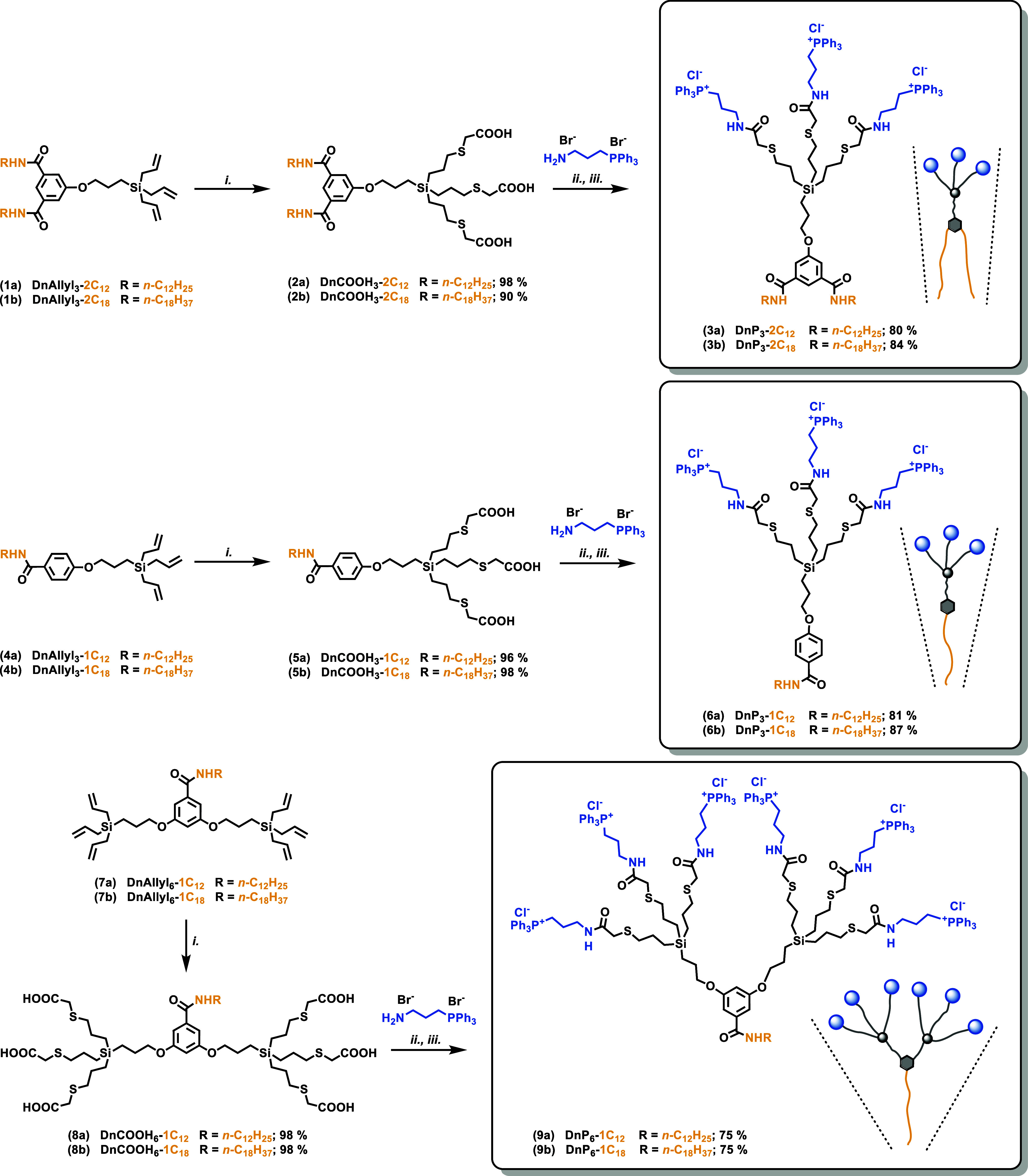
Synthetic Route toward amp-DDNs for the Multivalent Presentation of TPP Groups at the Periphery Conditions: (i) thioglycolic acid, DMPA, *h*ν, THF/MeOH, RT, 10 min; (ii) TBTU, DIPEA, DMF, RT, 4 h; (iii) Amberlite IRA-400 Cl^–^ cycle.

The presented synthetic approach (Scheme 1) was established in the course of the previously published study focused on the preparation of ammonium amp-DDNs.^35^ Allyl-terminated dendrons **1a**,**b**, **4a**,**b**, and **7a**,**b** reported there were used as starting materials for the new library. These substrates were functionalized with carboxyl moieties via TEC with thioglycolic acid to produce the corresponding carboxylic acids **2a**,**b**, **5a**,**b**, and **8a**,**b**. In the next step, TPP groups were attached via amide bonds with subsequent purification by OSN either in pure MeOH or in DCM/MeOH mixture, applying the method we have previously introduced for the purification of dendritic materials.^37^ Due to a larger molar volume of the TPP peripheral groups compared to dimethylammonium ones, we encountered much smaller losses during OSN than in the case of the precedent ammonium series; the smallest dendron with a 12-carbon chain and three TPP groups was obtained in 81% yield compared to 50% in case of its ammonium analogue.^35^ Finally, anion exchange to chlorides afforded a library of amphiphilic phosphonium dendrons DnP_3_-2C_12_, DnP_3_-2C_18_, DnP_3_-1C_12_, DnP_3_-1C_18_, DnP_6_-1C_12_, and DnP_6_-1C_18_ as white crystalline solids. Analogically to the previously reported ammonium series, these six dendrons cover a spectrum of geometries from compounds with a large lipophilic domain and cylindrical shape DnP_3_-2C_12_ and DnP_3_-2C_18_ to those with a large polar domain and conical shape DnP_6_-1C_12_ and DnP_6_-1C_18_.

**Scheme 2:**
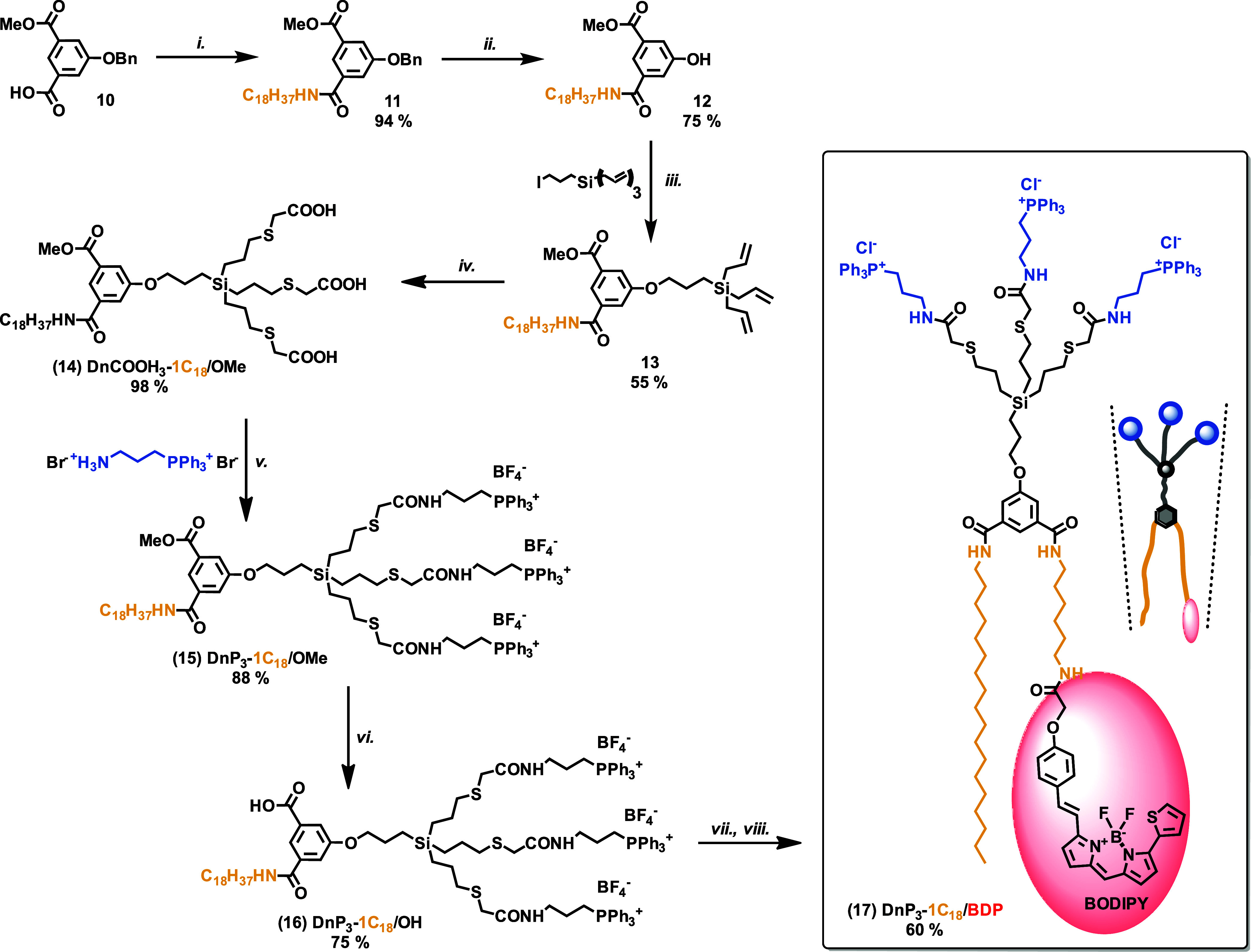
Synthetic Route toward Unsymmetrical Fluorescent Dendron DnP_3_-1C_18_/BDP Conditions: (i) CDI, NH_2_C_18_H_37_, DCM, RT, 6 h; (ii) H_2_, Pd/C, THF/MeOH, RT, 6 h; (iii) K_2_CO_3_, AcCN, reflux, 18 h; (iv) thioglycolic acid, DMPA, *h*ν, THF/MeOH, RT, 10 min; (v) TBTU, DIPEA, DMF, RT, 24 h; (vi) NaOH, MeOH, 55 °C, 20 h; (vii) BODIPY 630/650 amine, TBTU, DIPEA, DMF, RT, 2 h, darkness; (viii) Amberlite, DCM/MeOH.

To better address the needs of diverse bioapplications, our effort was further directed to the construction of amp-DDN functionalized with a fluorescent probe to enable the monitoring of micelles in a given environment via confocal microscopy. The labeled phosphonium dendron DnP_3_-1C_18_/BDP (**17**) with an 18-carbon chain and BODIPY (fluorescent tag) was synthesized according to Scheme 2, utilizing the general method developed recently in our group for the preparation of unsymmetrical dendrons with auxiliary groups.^35^ Initially, the carboxyl group of 5-benzyloxyisophthalic acid monomethyl ester **10** was used for attachment of the octadecyl amine by amidic coupling. Then, the hydroxyl group of the unsymmetrical amide **11** was deprotected yielding phenol **12**, which was reacted with the branched triallyl unit to afford an allyl-terminated dendron **13**. TEC with thioglycolic acid was performed to give compound **14** decorated with three carboxyl groups, which were subsequently utilized for the introduction of TPP groups by amidic coupling. As confirmed by HRMS, the resulting structure **15** contained mainly BF_4_^–^ anions due to the applied excess of the coupling agent TBTU. The ester group was hydrolyzed, affording dendron **16** with a new free carboxyl group, which was used to attach the BODIPY 630/650 amine with a pendant six-carbon linker ending with an amino group. Finally, anions were converted to chlorides, and dendron DnP_3_-1C_18_/BDP was purified by OSN. Its fluorescence properties were examined and compared to the parent BODIPY tag. Its grafting to the dendron resulted in a slight bathochromic shift of the red emission peak from 641 to 644 nm, whereas the other emission maximum at 573 nm remained unchanged, and the QY decreased to 55% of the free tag value.

### 3.2 Self-Assembly Behavior of Symmetrical Dendrons

**Figure 1:**
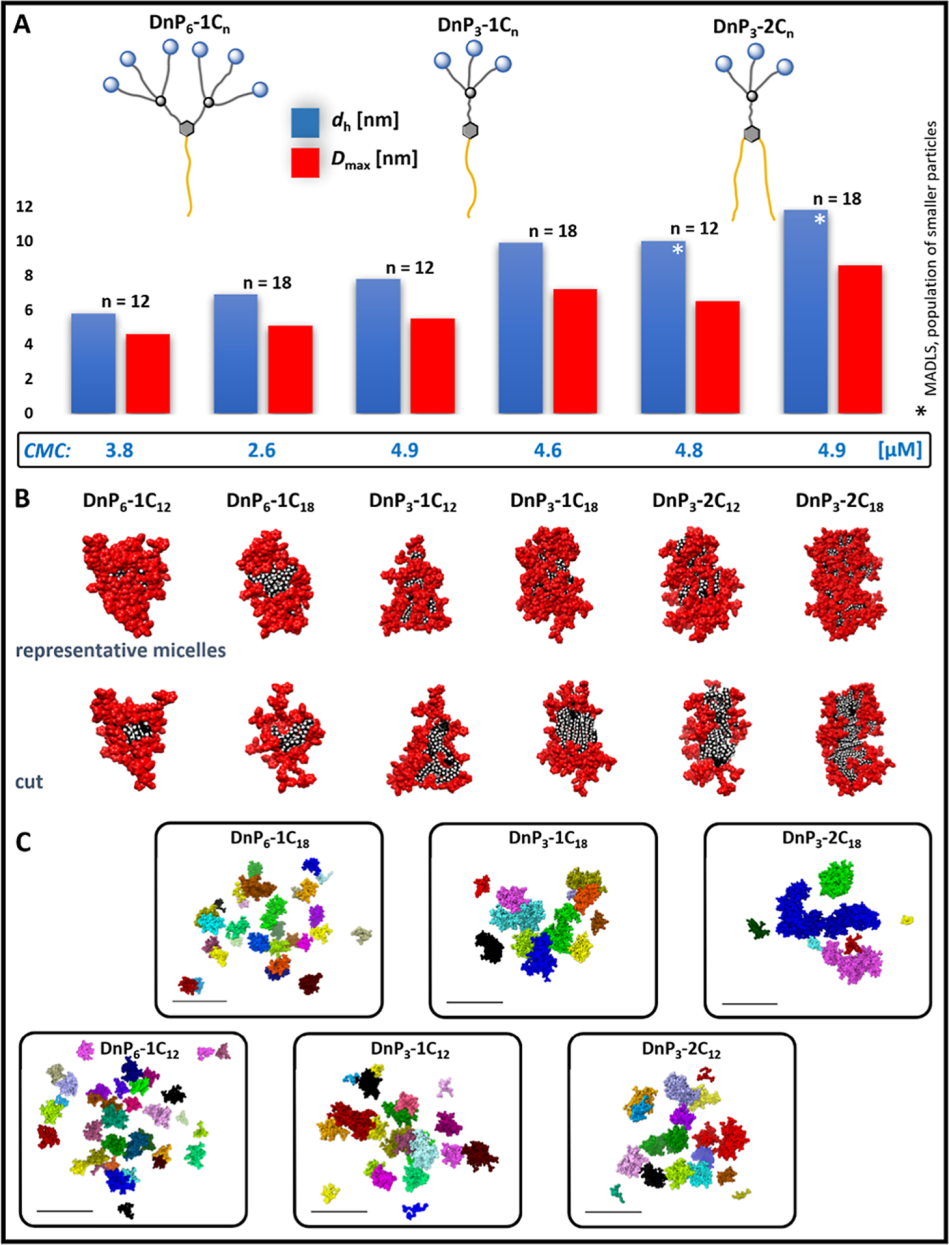
Self-assembly behavior of amp-DDNs. (A) Comparison of observed hydrodynamic diameters of particles in physiological solutions (*d*_h_) and average biggest dimensions of supramolecular structures obtained by molecular simulations in PBS (*D*_max_); estimated CMC values. (B) Representative micellar structures composed of phosphonium amp-DDNs obtained from molecular dynamics simulations (upper line); each dendron cluster is appended by its cut (lower line). Central aromatic ring and aliphatic chain carbons are highlighted in black and the rest of the structure in red. (C) Visualization of the final dendron systems simulated at 22 °C. Scale bar corresponds to 100 Å.

After the synthesis was completed, we turned our attention to the self-assembly behavior of the prepared amphiphiles in aqueous media. At first, the CMC of unlabeled amp-DDNs in phosphate-buffered saline was determined using the method based on significant difference between Nile Red fluorescence intensity in a hydrophilic aqueous environment and in a hydrophobic environment of a micelle core^38,39^ (Figures 1A and S1–S6 and Table S1). The obtained values are exceptionally low and fall into a narrow range between 2.6 and 4.9 μM. For comparison, CMC values of standard cationic surfactants such as cetyltrimethylammonium bromide are nearly 3 orders of magnitude higher and oscillate around 1 mM.^58^ Recently reported synthetic phosphorus dendrons with 10 pyrrolidinium end groups prepared in Majoral’s lab showed CMC 0.16 mM.^59^ Much lower values (12–25 μM), however, still significantly higher than those reported here, were reached with trimethylammonium-decorated carbosilane dendrons.^17^ The results indicate excellent thermodynamic stability of the nanoparticles formed by our amp-DDNs. Within the library, the two dendrons with a larger hydrophilic domain (DnP_6_-1C_12_ and DnP_6_-1C_18_) show somewhat lower CMCs than the rest which illustrates a stabilizing effect of the conical shape of the amphiphile on the micelle formation.

Next, we used DLS to determine the diameter of particles formed by dendrons in physiological solution partially mimicking the blood environment. The observed size consistently reflects the shape and structure of the particular molecular constituting units. As expected, dendrons with C_18_ chains formed larger micelles than their C_12_ counterparts; more generally, the particle size decreased with decreasing critical packing parameter (CPP). Cylindrical compounds (DnP_3_-2C_12_ and DnP_3_-2C_18_) formed the largest particles, whereas the smallest ones originated from conically shaped dendrons (DnP_6_-1C_12_ and DnP_6_-1C_18_). Although the differences in size were small, it is interesting to see the effect of systematic structural changes within the series. The dendron structure also has consequences for further aggregation. Whereas all dendrons with one alkyl chain formed highly uniform micelles (PDI 0.03–0.13), in which size and polydispersity (PI) were stable over months, the samples of DnP_3_-2C_12_ and DnP_3_-2C_18_ bearing two alkyl chains showed somewhat higher but still low PI (PDI ≈ 0.2) and much higher mean size (21 and 14 nm, respectively). Moreover, during the 75 day testing period, their mean size gradually increased to 25 and 26 nm, respectively (Table S2 and Figures S7–S12). To better understand the process behind this dynamic behavior, we applied multiple-angle DLS (MADLS; a method increasing the resolution of the multimodal size distribution) to the samples of DnP_3_-2C_12_ and DnP_3_-2C_18_. The results showed the presence of two populations of nanoparticles differing approximately 4–7 times in size (Figures S13 and S14). Mean diameter of the smaller particles (10.0 and 11.8 nm, respectively) corresponds to the presence of isolated micelles, while the second population (mean size 37 and 80 nm, respectively) is likely due to aggregates. The proportion of these two populations changes in time. So, a progressive aggregation of micelles rather than change of their actual size is responsible for the observed trend of increasing mean diameter.

### 3.3 Computer Modeling of Self-Assembly Behavior

A relationship between the structure of amp-DDNs and the size and structure of assembled particles was also studied on a molecular level by using molecular simulations in saline. In addition to a comparison between the outputs of the model and experimental observation in the case of symmetrical dendrons, the modeling also offered some insight into systems containing fluorescently labeled dendrons, which could not be characterized experimentally by methods discussed in the previous section due to their strong absorption and fluorescence. Computer models of dendrons were created, and the final molecular systems were obtained through Molecular Dynamics simulations at a reference temperature of 22 °C (in case of one-component systems also at temperatures 37 and 47 °C).

Similarly to DLS characterization, the results highlighted in Figure 1 and summarized in the Supporting Information (Figure S16, Table S3) show that DnP_3_-2C_18_ forms distinctly larger supramolecular structures than the remaining dendrons, with nearly 26 molecules in one cluster (N_mol-1-C_) on average. Then, structures assembled from DnP_3_-1C_18_ and DnP_3_-2C_12_ follow, with approximately 15 and 11 dendrons on average per cluster. Dendron DnP_3_-1C_12_ forms somewhat smaller particles comprising nearly eight molecules on average, while both dendrons with six TPP groups DnP_6_-1C_18_ and DnP_6_-1C_12_ similarly assemble to very small clusters of only three units on average, which corresponds well to their experimentally confirmed highest stability. Overall, the results are in good agreement with a prediction based on the molecular structures of particular dendrons as well as the experimentally obtained size characteristics of their assemblies in solution. Only in one case (the dimensionally closest micelles of DnP_3_-1C_18_ and DnP_3_-2C_12_), a reversed size order compared to the experiment is found. The slightly smaller theoretical values compared to experimental data can be attributed to the fact that DLS provides the hydrodynamic radius of hydrated particles (i.e., including solvation shell), while the size characteristics of clusters obtained from molecular modeling (average radius of gyration *R*_g_, average biggest dimension *D*_max_) refer to bare micelles. Also, both different nature of the dimensional characteristics used in experiments and theoretical calculations and the limited accuracy of molecular models must be taken into account when comparing theoretical and experimental values. In general, computer models confirm the expected relations between size of the nanoparticles and structure of the dendron: an increase of particle diameter with the size of the hydrophobic domain, i.e., the length and number of aliphatic chains, and, on the contrary, a decrease of its diameter with the size of the hydrophilic domain, probably due to increasing electrical repulsion and larger steric hindrance associated with this part of the dendron. Again, the most conical shape of amp-DDNs is shown to favor the formation of stable small micelles.

To evaluate the influence of temperature on the resulting one-component molecular systems, the simulations were repeated at different temperatures (22 °C, 37 °C, 47 °C) (Figure S17). Within the studied temperature range, we can observe a negative correlation between cluster size and temperature, whose significance increases with the average particle size. It is most evident for DnP_3_-2C_18_ forming the largest nanoparticles, followed by DnP_3_-1C_18_ and DnP_3_-2C_12_. Smaller nanoparticles composed of the remaining dendrons undergo negligible size changes in the studied temperature range, which confirm their high thermodynamic stability.

With the results for one-component systems in hand, we proceeded to study more complex two-component systems, each consisting of 95 mol % of the particular amp-DDN and 5 mol % of fluorescently labeled dendron DnP_3_-1C_18_/BDP. In all cases, the molecular modeling indicated the ability of labeled dendrons to interact with the unlabeled ones, integrate into the amphiphilic layers, and form stable composite supramolecular assemblies. The resulting clusters are depicted in Figure S18, with the labeled molecules marked in black to highlight their spatial distribution. In the case of DnP_3_-2C_18_, the addition of DnP_3_-1C_18_/BDP molecules resulted in a significant size increase of the nanoparticles compared to that of a one-component system. Similar, albeit less pronounced, effects can be observed for DnP_3_-2C_12_ and weakly even for DnP_3_-1C_18_-containing systems. For the remaining dendrons, no effect of adding DnP_3_-1C_18_/BDP on the particle size is noticeable (Figure S19). We suppose that the large size difference between two-component DnP_3_-2C_n_@DnP_3_-1C_18_/BDP particles and the parent one-component system originates mainly from two factors. First, the dendrons with two alkyl chains are characterized by most cylindrical shape from the series, thus the lowest driving force to form small micellar structures and consequently the highest tendency to reassembly.^4^ Second, the polyaromatic BODIPY moiety attached on a long alkyl spacer can interact not only with the aliphatic hydrophobic domain but also with phenyl groups of peripheral TPP cations or with the central aromatic ring (Figures S20–S22). Such interactions, most likely based on π-stacking, are widely reported in the literature in connection with this moiety.^60,61^ Thus, the labeled molecules act as a glue, stabilizing the large dendron assemblies via multiple interactions and clearly increasing the average size of the resulting nanoparticles. Results suggesting the preferential localization of the BODIPY moiety in the proximity of TPP groups in the hydrophilic domain at the aggregate periphery are in agreement with the observed decrease in QY after grafting the dye to the dendron. If the fluorescent label was preferentially localized in the lipophilic domain, we should observe the increase in emission due to shielding against the polar aqueous environment.

In addition, a one-component system consisting of 180 DnP_3_-1C_18_/BDP molecules at 22 °C was also modeled. Here, again, a single nanoparticle was formed in which, besides the interactions described above, the mutual BODIPY–BODIPY interaction is also prominent. Apparently, this supramolecular object has a somewhat different structure than nanoparticles formed within one-component systems of unlabeled dendrons (Figure S23). Its individual segments are made up of narrow elongated objects (tentacles) or rings with internal structure different from the spherical nanoparticles or segments of larger aggregates in the case of unlabeled dendrons. This structural difference is also apparent in the two-component system DnP_3_-2C_18_@DnP_3_-1C_18_/BDP, when comparing the sections that contain or lack the labeled dendrons.

### 3.4 Confocal Microscopy

**Figure 2:**
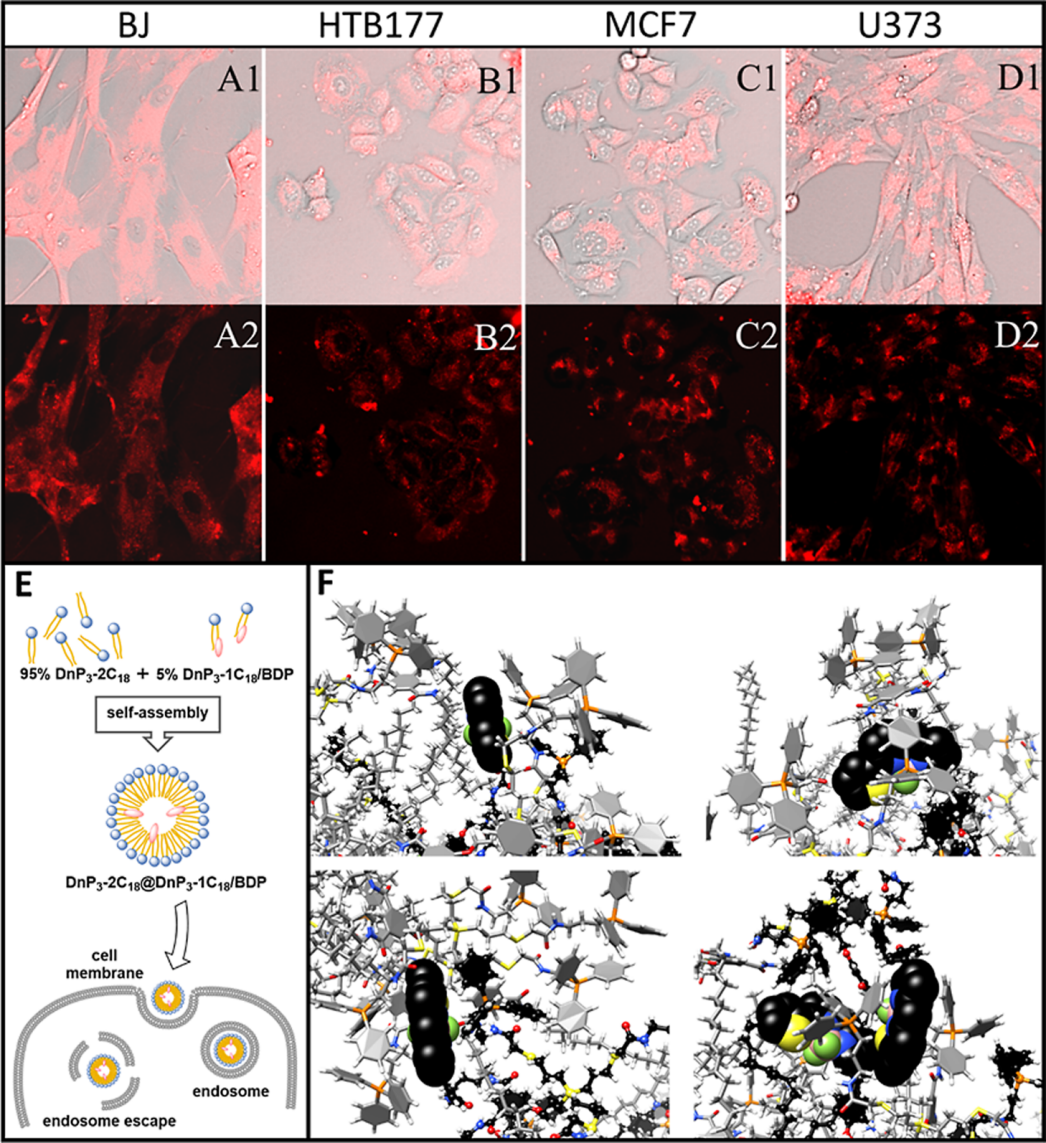
Representative confocal laser scanning fluorescence microscopy images of the internalization of DnP_3_-2C_18_@DnP_3_-1C_18_/BDP in four cell lines: (A) BJ; (B) HTB177; (C) MCF7; and (D) U373. (E) Schematic view of micelle formation and internalization into cells. (F) Details of the DnP_3_-2C_18_@DnP_3_-1C_18_/BDP system obtained by molecular simulation. The images show the ability of BODIPY (a spherical representation) to interact with different parts of the surrounding dendrons. Color coding: O—red, N—blue, S—yellow, F—green, H—white (not shown on BODIPY), C—gray (DnP_3_-2C_18_)/black (DnP_3_-1C_18_/BDP). For colored presentation, see Figures S20–S22.

To experimentally examine the distribution of amp-DDNs within cells, confocal microscopy was employed. Two-component system DnP_3_-2C_18_@DnP_3_-1C_18_/BDP containing 5 mol % of the fluorescently labeled dendron was studied on four cell lines, selected to represent different metabolism types from normal, noncancerous cells (BJ) through hormone-responsive breast cancer (HTB177) and progressive type lung cancer (MCF7) to a highly malignant brain cancer (U373), providing an insight into the behavior of nanoparticles in different environments. Obtained results depicted in Figure 2a revealed an efficient cellular internalization of amp-DDNs. Operative tracking was facilitated by the labeled dendron DnP_3_-1C_18_/BDP with covalently attached fluorescent dye, BODIPY. Remarkably, the addition of only 5 mol % of DnP_3_-1C_18_/BDP was sufficient to obtain a fluorescence signal of robust intensity, indicative of cellular uptake. Upon internalization, the dendrons accumulated to form fluorescent spots present inside the cytoplasm of the cells. These fluorescent puncta suggest endosomal internalization of amp-DDNs, which can then be delivered to specific cellular compartments. This observation is in line with several previous reports wherein endosomal internalization of other amp-DDNs was noted, evidenced by the presence of fluorescent puncta within the cytoplasm in confocal microscopy images.^62,63^ Interestingly, other studies indicate that not all dendron-based amphiphilic structures exhibit this mode of cellular uptake; a uniform distribution in the cytoplasm without preferential accumulation within a specific organelle was also observed in other cases.^64,65^

### 3.5 Cytotoxicity

**Figure 3:**
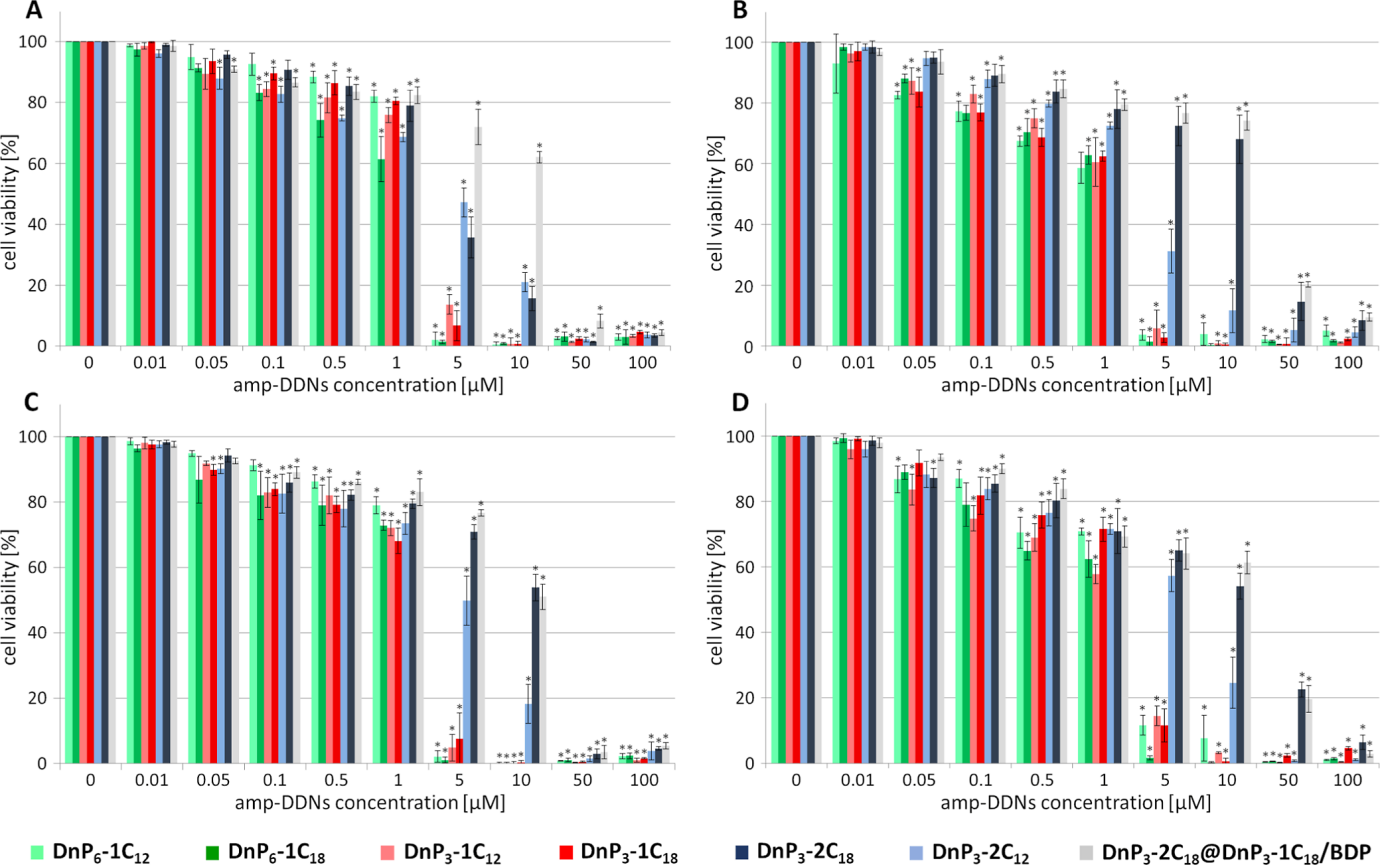
Cell viability of BJ (A), U373 (B), MCF7 (C), and HTB177 (D) cell lines after 24 h incubation with different concentrations of amp-DDNs. Two-way ANOVA comparison test was used to quantify the p values between the samples and control (* statistically significant *p* < 0.05). Data are presented as an average ± SD, *n* = 3.

Cytotoxicity of all unlabeled compounds and one two-component mixture DnP_3_-2C_18_@DnP_3_-1C_18_/BDP containing a fluorescently labeled dendron was tested on the same cell lines that we used for the internalization study presented above. The results of cell viability tests were analyzed on the basis of a graphical representation of data, which provided a comprehensive overview of the cytotoxicity profiles (Figure 3). Overall, a majority of tested amp-DDNs exhibited similar cytotoxicity patterns, indicating consistent behavior across the series. Noteworthy exceptions were observed in the case of dendrons with two alkyl groups, DnP_3_-2C_12_ and DnP_3_-2C_18_, and especially for the composite sample DnP_3_-2C_18_@DnP_3_-1C_18_/BDP, whose toxicity was markedly lower compared to the remaining species. Additionally, the cytotoxicity profiles varied depending on the cell line. Whereas BJ and MCF7 cell lines exhibited high viability up to 1 μM concentration with subsequent steep decrease at 5 μM, the viability of U373 and HTB177 decreased more gradually. On the other hand, the latter two cell lines were more resistant toward high concentrations of DnP_3_-2C_12_, DnP_3_-2C_18_, and DnP_3_-2C_18_@DnP_3_-1C_18_/BDP. The results of toxicity tests were used to determine IC50 concentrations for each variant of amp-DDN, as well as for the composite DnP_3_-2C_18_@DnP_3_-1C_18_/BDP (Table 1). Again, the highest values were observed for dendrons DnP_3_-2C_18_ and DnP_3_-2C_12_, along with the DnP_3_-2C_18_@DnP_3_-1C_18_/BDP composite. While DnP_3_-2C_18_ alone is unfortunately most toxic for normal cells, in combination with the labeled dendron, it exerts lower cytotoxicity, comparable for all tested cell lines. The IC50 values of amp-DDNs with one aliphatic chain displayed notable consistency, falling within a narrow range of 1.2–2.0 μM across all cell lines. Results reported recently by Majoral’s group also show no substantial differences between investigated cell lines for all tested amphiphilic phosphorus dendrons, exerting negligible toxicity up to concentration 2 μM.^66^

**Table 1:** Calculated IC50 Values (μM) of All amp-DDNs and One Two-Component Mixture

cell line	DnP_6_-1C_12_	DnP_6_-1C_18_	DnP_3_-1C_12_	DnP_3_-1C_18_	DnP_3_-2C_12_	DnP_3_-2C_18_	DnP_3_-2C_18_@DnP_3_-1C_18_/BDP
BJ	1.7 ± 0.2	1.2 ± 0.2	2.0 ± 0.2	1.8 ± 0.3	5.3 ± 1.9	3.1 ± 0.6	13.2 ± 2.7
U373	1.2 ± 0.1	1.3 ± 0.1	1.4 ± 0.3	1.4 ± 0.1	2.9 ± 1.3	17.5 ± 3.0	21.0 ± 1.0
HTB177	1.7 ± 0.1	1.2 ± 0.2	1.4 ± 0.2	1.7 ± 0.1	11.3 ± 6.4	12.0 ± 4.0	12.8 ± 3.4
MCF7	1.7 ± 0.1	1.6 ± 0.1	1.6 ± 0.1	1.6 ± 0.2	4.5 ± 1.1	20.0 ± 7.0	11.0 ± 1.0

The obtained results suggest a correlation between toxicity and dendron structure due to its influence on the aggregation behavior. The toxicity increases with decreasing CPP and decreasing CMC and mean particle size, being the highest for both conical dendrons and much lower for both dendrons with two alkyl chains; the lowest cytotoxicity and highest IC50 values were attributed to the two-component composite DnP_3_-2C_18_@DnP_3_-1C_18_/BDP, which is supposed to form large aggregates in solution (see Section 3.3). From the cell lines under study, U373 cells showed the largest dependence of IC50 values on the dendron structure (1.2 μM for DnP_6_-1C_12_ vs 21.0 μM for DnP_3_-2C_18_@DnP_3_-1C_18_/BDP). A rapid decrease of cell viability is associated with the dendron concentrations near the CMC, which suggests a rapid increase in the toxicity of aggregates compared to free dendrons. Above the CMC, the concentration of free dendrons remains nearly constant, while the concentration of aggregates increases, the increase being faster for smaller particles. This is in line with the observed inverse correlation between the size of the aggregates and their cytotoxicity. It was reported previously that the ability of amphiphiles to form larger structures can lead to differences in the toxicity level.^67,68^ In addition to the fact that the size of a nanoparticle can directly affect its interaction with a cell and hence its toxicity, for a given number of molecules in the system (i.e., equal molar concentration), the size of assemblies also determines their quantity, which may also contribute to the observed reduction of the IC50 with increasing particle size.

### 3.6 Antimicrobial Properties

With an increasing number of multiresistant bacterial strains, the demand for alternative antimicrobial agents preventing acquired resistance is growing. Among them, amphiphilic dendritic architectures have gained considerable attention as antimicrobial protein mimics. The primary mechanism of action of cationic amphiphiles relies on electrostatic interactions with the negatively charged bacterial surface. Subsequent penetration of their hydrophobic part into the bacteria’s cell membrane causes membrane disruption and, hence, cell death.^9^ Although phosphonium groups are known to provide antimicrobial properties to polymeric materials, dendritic phosphonium materials have been somewhat overlooked in this respect so far. The antibacterial activity of all unlabeled amp-DDNs was tested on selected Gram-positive (G+: *S. aureus* incl. methicillin-resistant form—MRSA, *S. epidermidis,E. faecium*) as well as Gram-negative (G–: *E. coli*, *K. pneumoniae,P. aeruginosa*) bacteria, related to healthcare-associated infections and antibiotic or multidrug resistance. The dendrons were incubated with the bacteria for 24 h, and then the MIC was determined. As already recognized, the structure of the amphiphiles, namely, the hydrophilic–hydrophobic balance, has a substantial impact on their antimicrobial activity.^9^ This also applies to the results summarized in Table 2: while both dendrons with two lipophilic chains did not show any activity in the studied concentration range, more conical dendrons with one lipophilic chain displayed excellent MICs ranging from 1 to 6 μmol/L for G+ bacteria and from 6 to 13 μmol/L for G– bacteria. The dendrons with shorter alkyl chains (DnP_6_-1C_12_ and DnP_3_-1C_12_) mostly expressed slightly higher antimicrobial activity, by 1 or 2 dilutions in 2-fold dilution series, compared to their analogues with longer alkyl chains (DnP_6_-1C_18_ and DnP_3_-1C_18_). Regarding G– bacteria, the MICs could not always be clearly determined because all amp-DDNs caused precipitation of culture broth at quite low concentrations (6 or 13 μmol/L), likely due to coaggregation with nutrient macromolecules present in the medium. We can hypothesize that the lower antibacterial activity of cylindrical dendrons DnP_3_-2C_12_ and DnP_3_-2C_18_, as well as their lower observed toxicity for human cells, is a consequence of their higher tendency to form bilayers instead of micelles. This can result in their easier incorporation into the cell membrane without extensive disorganization of the lipidic bilayer and limited damage to the cell at a lower concentration. Also, their higher CMC can play a role, as micelles are much more efficient in the coordination to the membrane surface due to multivalency effect.^69,70^

**Table 2:** MIC (μmol/L) of amp-DDNs against Selected Bacteria

		DnP_6_-1C_12_	DnP_6_-1C_18_	DnP_3_-1C_12_	DnP_3_-1C_18_	DnP_3_-2C_12_	DnP_3_-2C_18_
G+	S. aureus CCM 4516	**6.25**	**6.25**	**3.13**	**6.25**	>12.5	>12.5
	S. aureus MRSA—clinical isolate C1926	**6.25**	**6.25**	**3.13**	**6.25**	>12.5	>12.5
	S. epidermidis—clinical isolate C1936	**1.56**	**6.25**	**1.56**	**3.13**	>12.5	>12.5
	E. faecium VRE—clinical isolate S2484	**3.13**	**6.25**	**1.15**	**6.25**	>12.5	>12.5
G–	E. coli K12, CCM 7929	**6.25**	>6.25	**6.25**	>6.25	>12.5	>12.5
	E. coli—clinical isolate A1235	**6.25**	>6.25	**12.5**	>6.25	>12.5	>12.5
	K. pneumoniae ESBL+—clinical isolate C1914	**12.5**	>6.25	>12.5	>6.25	>12.5	>12.5
	P. aeruginosa MR—clinical isolate A1245	≥12.5	>6.25	>12.5	>6.25	>12.5	>12.5

The activity of prepared amp-DDNs at the tested concentration level can be considered highly effective since determined MICs for multidrug-resistant strains were often lower than MICs of commonly used antibiotics (Table S5). Comparison to other known dendritic amphiphiles is complicated due to limited availability of MIC data for bacterial species involved in this work. Concerning MRSA, the most efficient dendritic amphiphiles showed MICs in the range 6–15 μg/mL, which is similar to our findings (5.5–13.3 μg/mL for dendrons with one alkyl chain). Interestingly, unlike the majority of previously reported polycationic amp-DDNs,^9^ our dendrons show higher activity against G+ compared to G– bacteria.

### 3.7 Oligonucleotide Complexation Studies

As the last part of preliminary evaluation of the potential of prepared amp-DDNs toward biological applications, we performed a physicochemical study of dendriplexes formed from the dendritic micelles and therapeutic oligonucleotides. As a model oligonucleotide, we used a synthetic oligo(2′-*O*-methylribonucleotide) inhibitor of a natural microRNA miR-21. Synthetic inhibitors of miR-21 showed to have a considerable activity against melanoma,^71^ their potential in the therapy of Crohn’s disease has been also recently validated.^72^

The oligonucleotide complexation with dendritic micelles occurred upon the simple mixing of dendrons with anti-microRNA-21. The hydrodynamic diameter and PI of the dendriplexes, formed due to electrostatic interactions between the positively charged dendron periphery and the negatively charged nucleic acid and prepared at different charge ratios (CR, a ratio of positive to negative charges in the complex), were estimated by DLS (Figures S24–S33). The results shown in Table 3 are categorized according to the suitability of the observed nanoparticle sizes for further biological tests. Most dendriplexes with size suitable for bioapplications (50–250 nm)^73−75^ showed excellent PI (<0.20). In all cases, the best results in terms of size and PI combination were obtained at the lowest tested CR 1.2.

**Table 3:** Size and PI of Dendriplexes at Different Charge Ratios (±) as Estimated by DLS^a^

	mean diameter [μm] (PI)				
dendron	CR 1.2	CR 2.5	CR 5	CR 10	CR 20
DnP_6_-1C_12_	**0.22 (0.08)**	0.8 (0.2)	0.8 (0.10)	0.8 (0.05)	
DnP_6_-1C_18_	**0.18 (0.05)**	>1 (0.3)	>1 (0.3)	>1 (0.3)	
DnP_3_-1C_12_	**0.14 (0.07)**	0.9 (0.2)	>1 (0.3)	>1 (0.1)	
DnP_3_-1C_18_	**0.23 (0.14)**	0.5 (0.2)	>1 (0.4)	>1 (0.3)	
DnP_3_-2C_12_	**0.13 (0.11)**	0.8 (0.2)	1.0 (0.3)	**0.18 (0.14)**	**0.14 (0.17)**
DnP_3_-2C_18_	**0.20 (0.12)**	0.9 (0.2)	>1 (0.3)	**0.22 (0.23)**	**0.17 (0.29)**

a Sizes suitable for bioapplication (below 0.25 μm) are in bold, and the most promising results (below 0.15 μm) are underlined. The rest of dendriplexes are too large for cell internalization.

## 4 Conclusions

In summary, we have demonstrated the synthesis of novel phosphonium amp-DDNs with finely tunable properties of both hydrophilic and hydrophobic domain. The presented versatile synthetic protocol enables straightforward access to a library of dendrons decorated by charged TPP groups. The choice of the aromatic core allows one to adjust the number of polar dendritic wedges and the number and length of aliphatic chains. The feasibility of introducing additional functionality to the dendron molecule was demonstrated by the preparation of fluorescently labeled BODIPY-conjugated derivative DnP_3_-1C_18_/BDP.

Both experiments and molecular simulations have shown that all of the prepared TPP-modified dendrons self-assemble to micelles under the studied conditions. The size of micelles generally increases with increasing size of the dendrons’ hydrophobic domain, whereas an enlargement of the hydrophilic peripheral domain has the opposite effect. According to the obtained CMC values (3–5 μmol/L), all systems possess high thermodynamic stability.

The addition of the BODIPY-labeled dendron to the excess of the unlabeled symmetrical molecules resulted in its instant integration into the supramolecular structure and formation of fluorescent composite assemblies. Confocal microscopy showed an efficient cellular internalization of the composite micelles into both cancerous and noncancerous cell lines. Molecular modeling indicated that DnP_3_-1C_18_/BDP prefers even distribution over a micelle surface to self-aggregation and its addition in some cases, especially for DnP_3_-2C_18_, leads to an increase of mean particle size compared to the parent one-component system; for DnP_3_-2C_18_@DnP_3_-1C_18_/BDP composite, characterized by the smallest structure difference between the labeled and unlabeled dendron, the simulation resulted in the formation of a single large nanoparticle.

Preliminary experiments aimed at the evaluation of the application potential of the prepared amphiphiles also showed an unambiguous structure–activity relationship. The cytotoxicity of all amp-DDNs was low at submicromolar concentrations with a rapid increase at 1–5 μmol/L. The IC50 value and the rate of toxicity increase depend on the size of dendritic nanoparticles and the particular cell line, the lowest toxicity being observed for the dendrons forming the largest assemblies and especially for the fluorescently labeled composite. As a high efficiency of cellular uptake of these dendrons was demonstrated, this knowledge can be utilized in drug delivery and cellular imaging applications. Some of the dendrons also showed significant efficacy against pathogenic and multiresistant G*+* and G– bacteria which can be exploited in the field of medically targeted materials. Due to their inherent positive charge, all prepared dendrons are able to interact with nucleic acids; under appropriate conditions, dendriplexes of a desired size range and low PI were formed with anti-miRNA. Altogether, phosphonium dendrons demonstrated their potential as novel well-defined biomaterials in multiple areas.

## Data Availability

The data generated during the current study are available from the corresponding authors upon reasonable requests.

## Supporting Information Available

Full synthesis and analytical data of phosphonium amp-DDNs; ^1^H, ^13^C, ^29^Si, COSY, HSQC, and HMBC NMR spectra and mass spectra of phosphonium amp-DDNs; characterization of micelles: CMC estimation, DLS size and stability data, molecular modeling parameters, and results; DLS of dendriplexes; and antibacterial activity of common antibiotics (PDF)
