# Supplementary material for: Adaptive Synthesis, Supramolecular Behavior, and Biological Properties of Amphiphilic Carbosilane-Phosphonium Dendrons with Tunable Structure
Source: Biomacromolecules. 2024 Nov 11;25(12):7799–813. doi: 10.1021/acs.biomac.4c01092 (PMC11632778; doi:10.1021/acs.biomac.4c01092)
Supplement: Supplementary file 1 — bm4c01092_si_001.pdf [file bm4c01092_si_001.pdf]

Supplementary information

Adaptive synthesis, supramolecular behavior and biological properties of amphiphilic carbosilane-phosphonium dendrons with tunable structure

Antonín Edr[1], Dominika Wrobel[2], Alena Krupková[1], Lucie Červenková Šťastná[1], Evgeny Apartsin[5], Michaela Hympánová[4], Jan Marek[4,6], Jan Malý[2], Marek Malý[3], Tomáš Strašák[1*]

[1] The Czech Academy of Sciences, Institute of Chemical Process Fundamentals, 165 02 Prague, Czech Republic;

[2] Centre for Nanomaterials and Biotechnology Faculty of Science, Jan Evangelista Purkyně University in Ústí nad Labem, Pasteurova 3632/15, 400 96 Ústí nad Labem, Czech Republic

[3] Department of Physics, Faculty of Science, University of Jan Evangelista Purkyně in Ústí nad Labem, 400 96 Ústí nad Labem, Czech Republic

[4] Biomedical Research Centre, University Hospital Hradec Kralove, Sokolska 581, 500 05 Hradec Kralove, Czech Republic

[5] Univ. Bordeaux, CNRS, Bordeaux INP, CBMN, UMR 5248, F-33600 Pessac, France

[6] Department of Epidemiology, Military Faculty of Medicine, University of Defence, Trebesska 1575, 500 05 Hradec Kralove, Czech Republic

Table of contents

1. Detailed synthesis and characterization of compounds S2
2. CMC estimation S13
3. Summary of supramolecular properties S15
4. Micelle size and stability measurements S15
5. Molecular modelling S21
6. Dendriplex size measurements S29
7. Antibacterial activity of clinically used antibiotics S33
8. References S34
9. NMR spectra of amphiphilic dendrons S36
10. Mass spectra of amphiphilic dendrons S61

1. Detailed synthesis and characterization of compounds

Materials

Symmetrical dendritic precursors **2a**, **1b**, **4a**, **4b**, **7a** and **7b** and unsymmetrical benzoic acid **10** were prepared according to previously published procedure[1]. (3-aminopropyl)triphenylphosphonium bromide hydrobromide was prepared in analogy to literature procedure using 1.2 instead of 1.0 equivalent of triphenylphosphine to avoid traces of starting material in product[2].
THF was purified by distillation from sodium in argon atmosphere. K_2CO_3 was calcinated at 350 °C for 2 h and let cool down in a desiccator. Amberlite® IRA-400 was recycled using 1% NaOH solution for purification and 4% HCl solution for full conversion to Cl^- cycle.

Synthesis

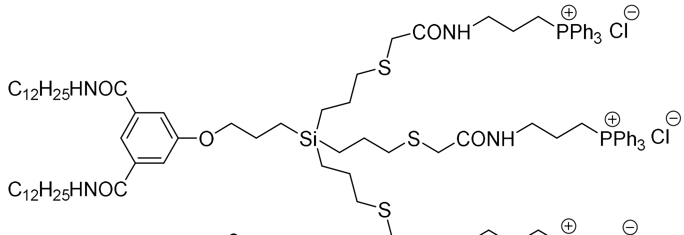

3a

Dendron DnP$_3$-2C$_{12}$ (3a). Dendron **2a** (375 mg, 0.381 mmol) and (3-ammoniumpropyl)triphenylphosphonium bromide (0.66 g, 1.4 mmol) were dissolved in dry DMF (6 mL) in argon atmosphere and stirred for 5 min. TBTU (404 mg, 1.26 mmol) in dry DMF (6 mL) was added and the solution was stirred for 15 min. DIPEA (0.53 mL, 3.0 mmol) was added and the reaction mixture was stirred at RT for another 4 h. Then, the volume of solvent was reduced, and excess reagents were removed by nanofiltration in MeOH. Retentate was applied to a column of ion-exchange resin Amberlite® IRA-400 in Cl^- cycle and was slowly let to flow through the column. The product was washed from the column by additional MeOH, the volume of solvent was reduced and the procedure repeated once. Finally, the solvent was removed to afford dendron **3a** (614 mg, 80 %, white foam). ^{1}H NMR (400 MHz, DMSO-d_6, ^{1}H-^{1}H COSY): δ 8.76 (t, J = 5.6 Hz, 2H, NH), 8.59 (t, J = 5.6 Hz, 3H, NH), 8.09 (t, J = 1.8 Hz, 1H, CH_{Ph}), 7.90–7.72 (m, 45H, CH_{Ph}), 7.51 (t, J = 1.8 Hz, 2H, CH_{Ph}), 3.98 (t, J = 6.3 Hz, 2H, OCH_2), 3.72–3.64 (m, 6H, PCH_2), 3.23 (td, J = 6.3, 5.6 Hz, 10H, CH_2NH, CH_2CH$_2$CH$_2$P), 3.12 (s, 6H, SCH_2CO), 2.52 (t, J = 6.6 Hz, 6H, SCH_2CH$_2$), 1.69–1.63 (m, 8H, PCH$_2$CH_2, OCH$_2$CH_2), 1.50–1.45 (m, 10H, CH_2CH$_2$S, CH_2), 1.28–1.21 (m, 36H, CH_2), 0.83 (t, J = 6.6 Hz, 6H, Me), 0.60–0.51 (m, 8H, SiCH_2). ^{13}C {^{1}H} NMR (101 MHz, DMSO-d_6, ^{1}H-^{13}C HSQC, ^{1}H-^{13}C HMBC): δ 169.4 (3CONH), 165.3 (2CONH), 158.5 (C_qO), 136.0 (2$C_{q(Ph)}$), 134.9 (d, $^4J_{(C\text{-}P)}$ = 3.2 Hz, CH$_{Ph}$), 133.6 (d, $^2J_{(C\text{-}P)}$ = 10.1 Hz, 2CH$_{Ph}$), 130.3 (d, $^3J_{(C\text{-}P)}$ = 12.5 Hz, 2CH$_{Ph}$), 118.3 (d, $^1J_{(C\text{-}P)}$ = 86.2 Hz, C_qP), 118.4 (CH$_{Ph}$), 115.6 (2CH$_{Ph}$), 70.6 (OCH$_2$), overlapped with DMSO from HSQC 38.8 (CH$_2$NH, CH$_2$CH$_2$CH$_2$P), 35.5 (SCH$_2$CH$_2$), 34.4 (SCH$_2$CO), 31.3, 29.03, 29.00 (3×2CH$_2$), 28.98 (4CH$_2$), 28.97, 28.8, 28.7, 26.5 (4×2CH$_2$), 23.4 (SCH$_2$$CH_2$), 23.3 (OCH$_2$$CH_2CH_2$), 24.6, 24.5 (2×$CH_2$), 22.2 (d, $^2J_{(C\text{-}P)}$ = 3.3 Hz, CH$_2$CH$_2$P), 22.1 (2CH$_2$), 18.5 (d, $^1J_{(C\text{-}P)}$ = 51.8 Hz, CH$_2$P), 13.9 (2Me), 11.2 (CH$_2$Si), 7.7 (OCH$_2$CH$_2$$CH_2$). ^{29}Si {^{1}H} NMR (79 MHz, dmso-d_6): δ 3.94. ^{31}P {^{1}H} NMR (162 MHz, DMSO-d_6): δ 7.16. HRMS (ESI+): Calcd. for $[C_{113}H_{151}Cl_2N_5O_6P_3S_3Si]^+$ 1960.9180, found 1960.9159 $[M\text{-}Cl]^+$; calcd. for $[C_{113}H_{151}ClN_5O_6P_3S_3Si]^{2+}$ 962.9743, found 962.9740 $[M\text{-}2Cl]^{2+}$; calcd. for $[C_{113}H_{151}N_5O_6P_3S_3Si]^{3+}$ 630.3264, found 630.3268 $[M\text{-}3Cl]^{3+}$.

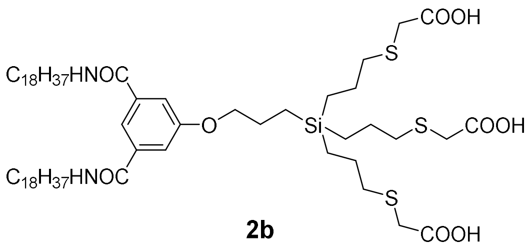

2b

Dendron 2b. Dendron **1b** (0.50 g, 0.57 mmol), 2-mercaptoacetic acid (0.47 g, 5.1 mmol, 0.36 mL) and DMPA (15 mg, 0.059 mmol) were dissolved in distilled THF (6 mL) in 10 mL vial and deoxygenated. The reaction mixture was stirred in argon atmosphere and irradiated for 15 min. Then, Et_2O (150 mL) was added and the mixture was washed (3 × 75 mL H_2O and 75 mL brine). The organic layer was dried by anhydrous $MgSO_4$ and the solvent was removed (at 35 °C). The residue was purified by nanofiltration in a 4:1 mixture of DCM and MeOH. The solvents were removed to afford dendron **2b** (593 mg, 90 %, colorless viscous liquid). ^{1}H NMR (400 MHz, DMSO-d_6, ^{1}H-^{1}H COSY): δ 8.46 (t, J = 5.6 Hz, 2H, NH), 7.86 (s, 1H, CH_{Ph}), 7.47 (s, 2H, CH_{Ph}), 4.00 (t, J = 6.6 Hz, 2H, OCH_2), 3.24 (td, J = 6.8, 5.6 Hz, 4H, CH_2NH), 3.18 (s, 6H, CH_2COOH), 2.58 (t, J = 7.2 Hz, 6H, CH$_2$CH_2S), 1.75–1.70 (m, 2H, OCH$_2$CH_2), 1.58–1.47 (m, 10H, CH_2CH$_2$NH, CH_2CH$_2$S), 1.28–1.22 (m, 60H, CH_2), 0.84 (t, J = 6.6 Hz, 3H, Me), 0.65–0.61 (m, 8H, SiCH_2). ^{13}C {^{1}H} NMR (101 MHz, DMSO-d_6, ^{1}H-^{13}C HSQC, ^{1}H-^{13}C HMBC): δ 171.6 (COOH), 165.4 (CONH), 158.4 (C_qO), 136.2 (2C_q), 118.5 (CH$_{Ph}$), 115.4 (2CH$_{Ph}$), 70.5 (OCH$_2$), overlapped with DMSO from HSQC 39.3 (CH$_2$NH), 35.5 (CH$_2$$CH_2$S), 33.1 ($CH_2$COOH), 31.3 (2$CH_2$), 29.1 (6$CH_2$), 29.04, 28.99 (2×8$CH_2$), 28.8, 28.7, 26.5 (3×2$CH_2$), 23.3 (OCH$_2$$CH_2$), 23.2 (SCH$_2$$CH_2$), 22.1 (2$CH_2$), 13.9 ($Me$), 11.2 ($CH_2$Si), 7.8 (OCH$_2CH_2$$CH_2$). ^{29}Si {^{1}H} NMR (79 MHz, DMSO-d_6): δ 4.06. HRMS (ESI-): Calcd. for $[C_{62}H_{111}N_2O_9S_3Si]^-$ 1151.7226, found 1151.7230 $[M-H]^-$.

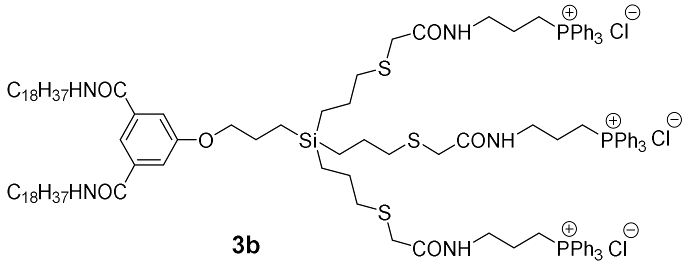

3b

Dendron DnP$_3$-2C$_{18}$ (3b). Dendron **2b** (567 mg, 0.491 mmol) and (3-ammoniumpropyl)triphenylphosphonium bromide (0.85 g, 1.8 mmol) were dissolved in dry DMF (20 mL) in argon atmosphere and stirred for 5 min. TBTU (498 mg, 1.55 mmol) was added in dry DMF (10 mL) and the solution was stirred for 15 min. DIPEA (0.65 mL, 3.7 mmol) was added and the reaction mixture was stirred at RT for another 4 h. Then, the volume of solvent was reduced, and excess reagents were removed by nanofiltration in a 1:1 mixture of DCM and MeOH. The retentate was evaporated to dryness, dissolved in MeOH (5 mL)and passed through a column of ion-exchange resin Amberlite® IRA-400 in Cl^- cycle. The product was washed from the column by additional MeOH, the volume of solvent was reduced and the procedure repeated once. Finally, the solvent was removed to afford dendron **3b** (891 mg, 84 %, white foam). ^{1}H NMR (400 MHz, DMSO-d_6, ^{1}H-^{1}H COSY): δ 8.77 (t, J = 5.8 Hz, 2H, NH), 8.58 (t, J = 5.8 Hz, 3H, NH), 8.12 (br s, 1H, CH_{Ph}), 7.90–7.72 (m, 45H, CH_{Ph}), 7.51 (br s, 2H, CH_{Ph}), 3.97 (t, J = 6.3 Hz, 2H, OCH_2), 3.73–3.64 (m, 6H, PCH_2), 3.23 (td, J = 6.5, 5.8 Hz, 8H, CH_2NH), 3.12 (s, 6H, SCH_2CO), 2.53 (t, J = 7.1 Hz, 6H, SCH_2CH$_2$), 1.69–1.63 (m, 10H, PCH$_2$CH_2, OCH$_2$CH_2), 1.52–1.45 (m, 10H, CH_2CH$_2$S, CH_2CH$_3$), 1.24–1.21 (m, 60H, CH_2), 0.83 (t, J = 6.7 Hz, 3H, Me), 0.60–0.51 (m, 8H, SiCH_2). ^{13}C {^{1}H} NMR (101 MHz, DMSO-d_6, ^{1}H-^{13}C HSQC, ^{1}H-^{13}C HMBC): δ 169.4 (3CO), 165.3 (2CO), 158.5 (C_qO), 135.9 (2C_q), 134.9 (d, $^4J_{(C-P)}$ = 3.0 Hz, CH$_{Ph}$), 133.6 (d, $^2J_{(C-P)}$ = 10.1 Hz, CH$_{Ph}$), 130.2 (d, $^3J_{(C-P)}$ = 12.5 Hz, CH$_{Ph}$), 118.3 (d, $^1J_{(C-P)}$ = 85.9 Hz, $C_{q(Ph)}$),

118.3 (CH_{Ph}), 115.6 ($2CH_{Ph}$), 70.5 (OCH_2), overlapped with DMSO from HSQC 38.8 (CH_2NH, $CH_2CH_2CH_2P$), 35.5 (SCH_2CH_2), 34.4 (SCH_2CO), 31.3 ($2CH_2$), 29.03 ($4CH_2$), 29.02 ($8CH_2$), 29.00 ($4CH_2$), 28.97 ($6CH_2$), 28.8, 28.7, 26.5 ($3{\times}2CH_2$), 23.4 (SCH_2CH_2), 23.3 ($OCH_2CH_2CH_2$), 22.2 (d, $^2J_{(C\text{-}P)}$ = 3.8 Hz, CH_2CH_2P), 22.1 ($2CH_2$), 18.5 (d, $^1J_{(C\text{-}P)}$ = 51.7 Hz, CH_2P), 13.9 (*Me*), 11.2 (CH_2Si), 7.7 ($OCH_2CH_2CH_2$). ^{29}Si {^{1}H} NMR (79 MHz, DMSO-d_6): δ 3.93. ^{31}P {^{1}H} NMR (162 MHz, DMSO-d_6): δ 7.17. HRMS (ESI+): Calcd. for $[C_{125}H_{175}ClN_5O_6P_3S_3Si]^{2+}$ 1078.0682, found 1047.0676 $[M\text{-}2Cl]^{2+}$; calcd. for $[C_{125}H_{175}N_5O_6P_3S_3Si]^{3+}$ 686.3890, found 686.3885 $[M\text{-}3Cl]^{3+}$.

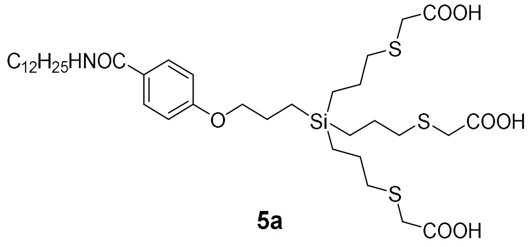

5a

Dendron 5a. Dendron **4a** (300 mg, 0.603 mmol), 2-mercaptoacetic acid (0.38 mL, 5.4 mmol) and DMPA (15 mg, 0.060 mmol) were dissolved in distilled THF (6 mL) in 10mL vial and deoxygenated. The reaction mixture was stirred in argon atmosphere and irradiated for 10 min. Then, DCM (70 mL) was added, the solution was washed with water (3 × 50 mL) and dried by anhydrous $MgSO_4$. The solvents were removed to afford dendron **5a** (448 mg, 96 %, yellowish viscous substance). ^{1}H NMR (400 MHz, DMSO-d_6, ^{1}H-^{1}H COSY): δ 8.24 (t, J = 5.6 Hz, 1H, N*H*), 7.78, 6.95 (2×d, J = 8.8 Hz, 2×2H, CH_{Ph}), 3.97 (t, J = 6.6 Hz, 2H, C_qOCH_2), 3.21 (td, J = 6.6, 5.6 Hz, 2H, CH_2NH), 3.19 (s, 6H, CH_2COOH), 2.58 (t, J = 7.2 Hz, 6H, CH_2CH_2S), 1.72–1.66 (m, 2H, OCH_2CH_2), 1.57–1.47 (m, 8H, CH_2CH_3, CH_2CH_2S), 1.27–1.23 (m, 18H, CH_2), 0.84 (t, J = 6.6 Hz, 3H, *Me*), 0.65–0.60 (m, 8H, $SiCH_2$). ^{13}C {^{1}H} NMR (101 MHz, DMSO-d_6, ^{1}H-^{13}C HSQC, ^{1}H-^{13}C HMBC): δ 171.6 ($COOH$), 165.5 ($CONH$), 160.8 (C_qO), 128.9 ($2CH_{Ph}$), 126.8 (C_q), 113.8 ($2CH_{Ph}$), 70.2 (C_qOCH_2), overlapped with DMSO from HSQC 39.1 (CH_2NH), 34.4 (CH_2CH_2S), 33.2 (CH_2COOH), 31.3, 29.2, 29.04, 29.02 ($4{\times}CH_2$), 28.99 ($2CH_2$), 28.8, 28.7, 26.5 ($3{\times}CH_2$), 23.3 ($SiCH_2CH_2$), 23.2 (OCH_2CH_2), 22.1 (CH_2), 14.0 (*Me*), 11.2 (CH_2Si), 7.6 ($OCH_2CH_2CH_2$). ^{29}Si {^{1}H} NMR (79 MHz, DMSO-d_6): δ 4.10. HRMS (ESI+): Calcd. for $[C_{37}H_{63}NO_8S_3SiNa]^+$ 796.3377, found 796.3370 $[M+Na]^+$; calcd. for $[C_{74}H_{126}N_2O_{16}S_6Si_2Na]^+$ 1569.6862, found 1569.6765 $[2M+Na]^+$.

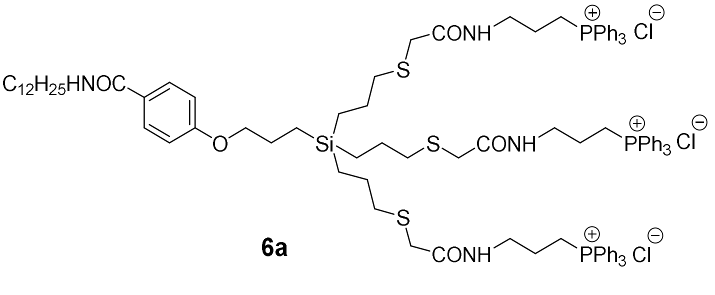

6a

Dendron DnP$_3$-1C$_{12}$ (6a). Dendron **5a** (448 mg, 0.579 mmol) and (3-ammoniumpropyl)triphenylphosphonium bromide (1.25 g, 2.60 mmol) were dissolved in dry DMF (15 mL) in argon atmosphere and stirred at 50 °C for 5 min. TBTU (669 mg, 2.08 mmol) in dry DMF (5 mL) was added and the solution was stirred at 50 °C for 15 min. DIPEA (0.81 mL, 4.6 mmol) was added and the reaction mixture was stirred at 50 °C for another 7 h. Then, the volume of solvent was reduced, and the excess reagents were removed by nanofiltration in a 1:1 mixture of DCM and MeOH. The retentate was evaporated to dryness, dissolved in MeOH (5 mL) and passed through a column of

ion-exchange resin Amberlite® IRA-400 in Cl^- cycle. The product was washed from the column by additional MeOH, the volume of solvent was reduced, and the procedure repeated once. Finally, the solvent was removed to afford dendron **6a** (840 mg, 81 %, white foam). ^{1}H NMR (400 MHz, DMSO-d_6, ^{1}H-^{1}H COSY): δ 8.72 (t, J = 5.7 Hz, 3H, NH), 8.41 (t, J = 5.4 Hz, 1H, NH), 7.90–7.73 (m, 47H, CH_{Ph}), 6.92 (d, J = 8.8 Hz, 2H, CH_{Ph}), 3.93 (t, J = 6.6 Hz, 2H, C_qOCH_2), 3.74–3.68 (m, 6H, PCH_2), 3.23 (td, J = 6.6, 5.7 Hz, 6H, CH_2NH), 3.21 (td, J = 6.6, 5.4 Hz, 2H, CH_2NH), 3.15 (s, 6H, SCH_2CO), 2.53 (t, J = 6.6 Hz, 6H, SCH_2CH$_2$), 1.69–1.63 (m, 8H, PCH$_2$CH_2, OCH$_2$CH_2), 1.50–1.44 (m, 8H, CH_2CH$_2$S, CH_2CH$_3$), 1.27–1.22 (m, 18H, CH_2), 0.83 (t, J = 6.6 Hz, 3H, Me), 0.58–0.51 (m, 8H, SiCH_2). ^{13}C {^{1}H} NMR (101 MHz, DMSO-d_6, ^{1}H-^{13}C HSQC, ^{1}H-^{13}C HMBC): δ 169.4 (3CO), 165.5 (CO), 160.7 (C_qO), 134.9 (d, $^4J_{(C\text{-}P)}$ = 3.0 Hz, CH$_{Ph}$), 133.6 (d, $^2J_{(C\text{-}P)}$ = 10.2 Hz, CH$_{Ph}$), 130.2 (d, $^3J_{(C\text{-}P)}$ = 12.5 Hz, CH$_{Ph}$), 129.0 (2CH$_{Ph}$), 126.7 (C_q), 118.4 (d, $^1J_{(C\text{-}P)}$ = 85.9 Hz, C_qP), 113.4 (2CH$_{Ph}$), 70.1 (OCH$_2$), overlapped with DMSO from HSQC 38.7 (CH$_2$NH, CH$_2$CH$_2$CH$_2$P), 35.5 (SCH$_2$CH$_2$), 34.4 (SCH$_2$CO), 31.3, 29.2, 29.02, 29.00 (4×CH$_2$), 28.98 (2CH$_2$), 28.8, 28.7, 26.5 (3×CH$_2$), 23.4 (SCH$_2$$CH_2$), 23.2 (OCH$_2$$CH_2CH_2$), 22.2 (d, $^2J_{(C\text{-}P)}$ = 3.9 Hz, CH$_2$CH$_2$P), 22.1 (CH$_2$), 18.5 (d, $^1J_{(C\text{-}P)}$ = 51.9 Hz, CH$_2$P), 13.9 (Me), 11.2 (CH$_2$Si), 7.6 (OCH$_2$CH$_2$$CH_2$). ^{29}Si {^{1}H} NMR (79 MHz, DMSO-d_6): δ 3.95. ^{31}P {^{1}H} NMR (162 MHz, DMSO-d_6): δ 7.18. HRMS (ESI+): Calcd. for $[C_{100}H_{126}Cl_2N_4O_5P_3S_3Si]^+$ 1749.7240, found 1749.7237 $[M\text{-}Cl]^+$; calcd. for $[C_{100}H_{126}ClN_4O_5P_3S_3Si]^{2+}$ 857.3775, found 857.3769 $[M\text{-}2Cl]^{2+}$; calcd. for $[C_{100}H_{126}N_4O_5P_3S_3Si]^{3+}$ 559.9285, found 559.9289 $[M\text{-}3Cl]^{3+}$.

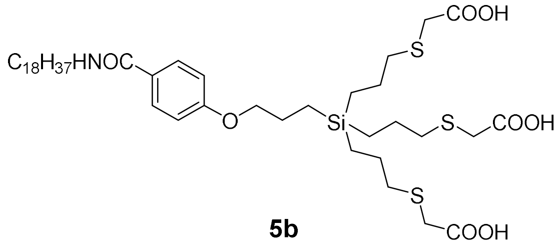

5b

Dendron 5b. Dendron **4b** (0.48 g, 0.82 mmol), 2-mercaptoacetic acid (0.51 mL, 7.4 mmol) and DMPA (21 mg, 0.082 mmol) were dissolved in distilled THF (8 mL) in 10mL vial and deoxygenated. The reaction mixture was stirred in argon atmosphere and irradiated for 10 min. Then, DCM (100 mL) was added, the solution was washed with water (3 × 80 mL) and dried by anhydrous $MgSO_4$. The solvents were removed to afford dendron **5b** (0.70 g, 98 %, yellowish viscous substance). ^{1}H NMR (400 Hz, DMSO-d_6, ^{1}H-^{1}H COSY): δ 8.24 (t, J = 5.6 Hz, 1H, NH), 7.78, 6.95 (2×d, J = 8.6 Hz, 2×2H, CH_{Ph}), 3.96 (t, J = 6.6 Hz, 2H, OCH_2CH$_2$), 3.19 (s, 8H, NHCH_2, SCH_2COOOH), 2.58 (t, J = 7.2 Hz, 6H, CH$_2$CH_2S), 1.70 (p, J = 6.7 Hz, 2H, OCH$_2$CH_2), 1.57–1.45 (m, 8H, SiCH$_2$CH_2, NHCH$_2$CH_2), 1.30–1.23 (m, 30H, CH_2), 0.85 (t, J = 6.6 Hz, 3H, Me), 0.65–0.60 (m, 8H, SiCH_2). ^{13}C {^{1}H} NMR (101 MHz, DMSO-d_6, ^{1}H-^{13}C HSQC, ^{1}H-^{13}C HMBC): δ 171.6 (3CO), 165.5 (CO), 160.8 (C_qO), 128.9 (2CH$_{Ph}$), 126.8 (C_q), 113.8 (2CH$_{Ph}$), 70.2 (OCH$_2$), overlapped with DMSO from HSQC 39.1 (NCH$_2$), 35.5 (CH$_2$$CH_2$S), 33.1 (S$CH_2$COOH), 31.3, 29.2 (2×$CH_2$), 29.05 (2$CH_2$), 29.03 (4$CH_2$), 28.99 (2$CH_2$, octadecyl), 28.8, 28.7, 26.5, 26.0, 23.3 (5×CH$_2$), 23.24 (CH$_2$CH$_2$S), 23.20 (OCH$_2$$CH_2$), 22.1 ($CH_2$), 14.0 ($Me$), 11.2 (3Si$CH_2$), 7.6 (1$CH_2$Si). ^{29}Si {^{1}H} NMR (79 MHz, DMSO-d_6): δ 4.10. HRMS (ESI+): Calcd. for $[C_{43}H_{75}NO_8S_3SiNa]^+$ 880.4316, found 880.4304 (100%) $[M+Na]^+$.

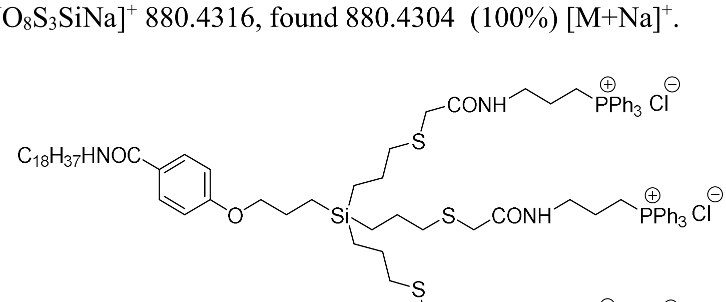

6b

Dendron DnP$_3$-1C$_{18}$ (6b). Dendron **5b** (200 mg, 0.233 mmol) and (3-ammoniumpropyl)triphenylphosphonium bromide (505 mg, 1.05 mmol) were dissolved in dry DMF (6 mL) in argon atmosphere and stirred at 50 °C for 5 min. TBTU (247 mg, 0.769 mmol) was added in dry DMF (4 mL) and the solution was stirred at 50 °C for 15 min. DIPEA (0.33 mL, 1.9 mmol) was added and the reaction mixture was stirred at 50 °C for another 4 h. Then, the volume of solvent was reduced, and excess reagents were removed by nanofiltration in MeOH. The retentate was added to a column of ion-exchange resin Amberlite® IRA-400 in Cl^- cycle and was let slowly flow through the column. The product was washed from the column by additional MeOH, the volume of solvent was reduced, and the procedure repeated once. Finally, the solvent was removed to afford dendron **6b** (380 mg, 87 %, white foam). ^{1}H NMR (400 MHz, DMSO-d_6, ^{1}H-^{1}H COSY): δ 8.70 (t, J = 5.7 Hz, 3H, N*H*), 8.40 (t, J = 5.8 Hz, 1H, N*H*), 7.87–7.74 (m, 47H, CH_{Ph}), 6.91 (d, J = 8.4 Hz, 2H, CH_{Ph}), 3.92 (t, J = 6.4 Hz, 2H, OCH_2), 3.74–3.67 (m, 6H, PCH_2), 3.24–3.19 (m, 8H, CH_2NH), 3.12 (s, 6H, SCH_2CO), 2.51 (t, J = 6.6 Hz, 6H, SCH_2CH$_2$), 1.68–1.62 (m, 8H, PCH$_2$CH_2, OCH$_2$CH_2), 1.49–1.43 (m, 8H, CH_2CH$_2$S, CH_2CH$_3$), 1.24–1.20 (m, 30H, CH_2), 0.82 (t, J = 6.6 Hz, 6H, *Me*), 0.54–0.51 (m, 8H, SiCH_2). ^{13}C {^{1}H} NMR (101 MHz, DMSO-d_6, ^{1}H-^{13}C HSQC, ^{1}H-^{13}C HMBC): δ 169.4 (3*C*O), 165.5 (2*C*O), 160.7 (C_qO), 134.9 (d, $^4J_{(C-P)}$ = 3.3 Hz, *C*H$_{Ph}$), 133.6 (d, $^2J_{(C-P)}$ = 10.1 Hz, *C*H$_{Ph}$), 130.2 (d, $^3J_{(C-P)}$ = 12.4 Hz, *C*H$_{Ph}$), 129.0 (C_q), 126.7 (2*C*H$_{Ph}$), 118.4 (d, $^1J_{(C-P)}$ = 86.9 Hz, C_qP), 113.7 (2*C*H$_{Ph}$), 70.1 (O*C*H$_2$), overlapped with DMSO from HSQC 38.6 (*C*H$_2$NH, *C*H$_2$CH$_2$CH$_2$P), 35.5 (S*C*H$_2$CH$_2$), 34.4 (S*C*H$_2$CO), 31.3, 29.2 (2×*C*H$_2$), 29.02, 29.00, 28.98 (3×*C*H$_2$), 28.8 (*C*H$_2$), 28.7 (2*C*H$_2$), 26.5 (*C*H$_2$), 23.4 (SCH$_2$*C*H$_2$), 23.2 (OCH$_2$*C*H$_2$CH$_2$), 22.2 (d, $^2J_{(C-P)}$ = 3.8 Hz, *C*H$_2$CH$_2$P), 22.1 (*C*H$_2$), 18.5 (d, $^1J_{(C-P)}$ = 51.8 Hz, *C*H$_2$P), 13.9 (*Me*), 11.2 (*C*H$_2$Si), 7.6 (OCH$_2$CH$_2$*C*H$_2$).^{29}Si {^{1}H} NMR (79 MHz, DMSO-d_6): δ 3.95. ^{31}P {^{1}H} NMR (162 MHz, DMSO-d_6): δ 7.18. HRMS (ESI+): Calcd. for $[C_{106}H_{138}Cl_2N_4O_5P_3S_3Si]^+$ 1833.8183, found 1833.8169 $[M-Cl]^+$; calcd. for $[C_{106}H_{138}ClN_4O_5P_3S_3Si]^{2+}$ 899.4245, found 899.4236 $[M-2Cl]^{2+}$; calcd. for $[C_{106}H_{138}N_4O_5P_3S_3Si]^{3+}$ 587.9598, found 587.9590 $[M-3Cl]^{3+}$.

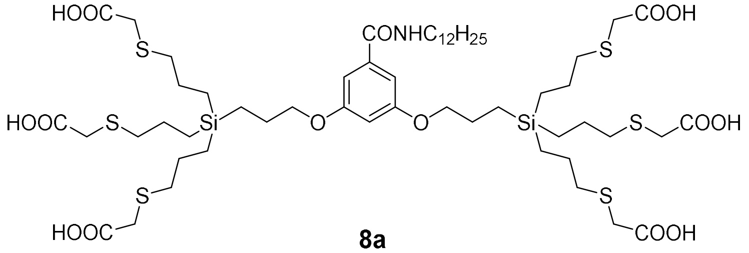

8a

Dendron 8a. Dendron **7a** (200 mg, 0.283 mmol), 2-mercaptoacetic acid (0.35 mL, 5.1 mmol) and DMPA (7 mg, 0.03 mmol) were dissolved in distilled THF (8 mL) in 10mL vial and deoxygenated. The reaction mixture was stirred in argon atmosphere and irradiated for 10 min. Then, DCM (75 mL) was added, and the solution was washed with water (3 × 50 mL) and dried by anhydrous $MgSO_4$. The solvents were removed to afford dendron **8a** (343 mg, 96 %, yellowish viscous substance). ^{1}H NMR (400 MHz, DMSO-d_6, ^{1}H-^{1}H COSY): δ 8.33 (t, J = 5.6 Hz, 1H, N*H*), 6.95 (d, J = 2.2 Hz, 2H, CH_{Ph}), 6.58 (t, J = 2.2 Hz, 1H, CH_{Ph}), 3.93 (t, J = 6.6 Hz, 4H, OCH_2), 3.18 (s, 14H, CH_2COOH, CH_2NH), 2.58 (t, J = 7.1 Hz, 12H, CH$_2$CH_2S), 1.72–1.66 (m, 4H, OCH$_2$CH_2), 1.58–1.50 (m, 14H, CH_2CH$_3$, CH_2CH$_2$S), 1.27–1.23 (m, 18H, CH_2), 0.88 (t, J = 6.6 Hz, 3H, *Me*), 0.64–0.60 (m, 16H, SiCH_2). ^{13}C {^{1}H} NMR (101 MHz, DMSO-d_6, ^{1}H-^{13}C HSQC, ^{1}H-^{13}C HMBC): δ 171.6 (*C*OOH), 165.6 (*C*ONH), 159.7 (C_qO), 136.7 ($C_{q(Ph)}$), 105.5 (2*C*H$_{Ph}$), 103.5 (*C*H$_{Ph}$), 70.3 (O*C*H$_2$), overlapped with DMSO from HSQC 39.0 (*C*H$_2$NH), 34.5 (CH$_2$*C*H$_2$S), 33.2 (*C*H$_2$COOH), 31.3, 29.1 (2×*C*H$_2$), 29.03, 29.00 (2×2*C*H$_2$), 28.8, 28.7, 26.5 (3×*C*H$_2$), 23.3 (SiCH$_2$*C*H$_2$), 22.1 (*C*H$_2$), 14.0 (*Me*), 11.2 (*C*H$_2$Si), 7.7 (OCH$_2$CH$_2$*C*H$_2$). ^{29}Si {^{1}H} NMR (79 MHz, DMSO-d_6): δ 4.07. HRMS (ESI+): Calcd. for $[C_{55}H_{95}NO_{15}S_6Si_2Na]^+$ 1280.4456, found 1280.4422 $[M+Na]^+$.

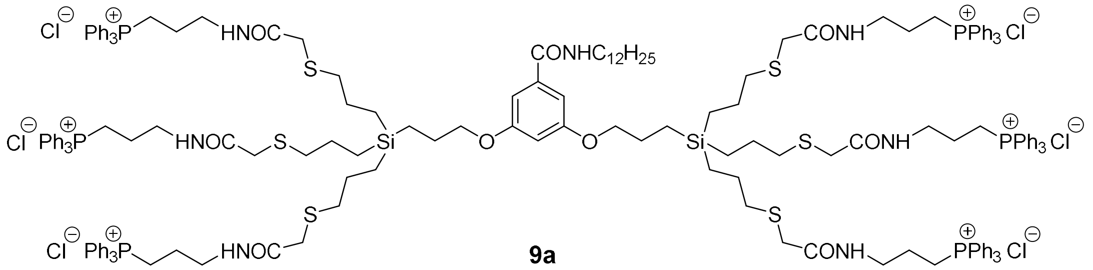

9a

Dendron DnP$_6$-1C$_{12}$ (9a). Dendron **8a** (343 mg, 0.272 mmol) and (3-ammoniumpropyl)triphenylphosphonium bromide (1.2 g, 2.46 mmol) were dissolved in dry DMF (15 mL) in argon atmosphere and stirred at 40 °C for 5 min. TBTU (576 mg, 1.80 mmol) in dry DMF (5 mL) was added and the solution was stirred at 40 °C for 15 min. DIPEA (0.77 mL, 4.4 mmol) was added and the reaction mixture was stirred at 40 °C for another 12 h. Then, the volume of solvent was reduced and excess reagents were removed by nanofiltration in MeOH. Retentate was added to a column of ion-exchange resin Amberlite® IRA-400 in Cl^- cycle and was slowly let to flow through the column. The product was washed from the column by additional MeOH, the volume of solvent was reduced, and the procedure was repeated once. Finally, the solvent was removed to afford dendron **9a** (754 mg, 84 %, white foam). ^{1}H NMR (400 MHz, DMSO-d_6, ^{1}H-^{1}H COSY): δ 8.73 (t, J = 5.7 Hz, 3H, NH), 8.59 (br s, 1H, NH), 7.88–7.73 (m, 90H, CH_{Ph}), 7.01 (br s, 2H, CH_{Ph}), 6.53 (br s, 1H, CH_{Ph}), 3.88 (t, J = 6.4 Hz, 4H, OCH_2), 3.75–3.68 (m, 12H, PCH_2), 3.21 (td, J = 6.4, 5.7 Hz, 12H, CH_2NH), 3.12 (s, 14H, SCH_2CO, CH_2NH), 2.52 (t, J = 6.6 Hz, 12H, SCH_2CH$_2$), 1.67–1.60 (m, 16H, PCH$_2$CH_2, OCH$_2$CH_2), 1.48–1.41 (m, 14H, CH_2CH$_2$S, CH_2CH$_3$), 1.22–1.19 (m, 18H, CH_2), 0.81 (t, J = 6.6 Hz, 3H, Me), 0.57–0.50 (m, 16H, SiCH_2). ^{13}C {^{1}H} NMR (101 MHz, DMSO-d_6, ^{1}H-^{13}C HSQC, ^{1}H-^{13}C HMBC): δ 169.4 (6CO), 165.5 (CO), 159.6 (C_qO), 136.6 (2$C_{q(Ph)}$), 134.9 (d, $^4J_{(C-P)}$ = 3.0 Hz, CH$_{Ph}$), 133.6 (d, $^2J_{(C-P)}$ = 10.1 Hz, CH$_{Ph}$), 130.2 (d, $^3J_{(C-P)}$ = 12.4 Hz, CH$_{Ph}$), 118.4 (d, $^1J_{(C-P)}$ = 85.8 Hz, $C_{q(Ph)}$), 105.6 (2CH$_{Ph}$), 103.6 (CH$_{Ph}$), 70.1 (OCH$_2$), overlapped with DMSO from HSQC 38.9 (CH$_2$NH), 38.6 (CH$_2$CH$_2$CH$_2$P), 35.5 (SCH$_2$CH$_2$), 34.4 (SCH$_2$CO), 31.3, 29.00 (2×CH$_2$), 28.98, 28.95 (2×2CH$_2$), 28.73, 28.67, 26.5 (3×CH$_2$), 23.4 (SCH$_2$$CH_2$), 23.3 (OCH$_2$$CH_2CH_2$), 22.2 (d, $^2J_{(C-P)}$ = 3.9 Hz, CH$_2$CH$_2$P), 22.1 (CH$_2$), 18.5 (d, $^1J_{(C-P)}$ = 51.7 Hz, CH$_2$P), 14.0 (Me), 11.2 (CH$_2$Si), 7.8 (OCH$_2$CH$_2$$CH_2$). ^{29}Si {^{1}H} NMR (79 MHz, DMSO-d_6): δ 3.91. ^{31}P {^{1}H} NMR (162 MHz, DMSO-d_6): δ 7.16. HRMS (ESI+): Calcd. for $[C_{181}H_{221}Cl_4N_7O_9P_6S_6Si_2]^{2+}$ 1605.1041, found 1605.1043 $[M-2Cl]^{2+}$; calcd. for $[C_{181}H_{221}Cl_3N_7O_9P_6S_6Si_2]^{3+}$ 1058.4129, found 1058.4147 $[M-3Cl]^{3+}$; calcd. for $[C_{181}H_{221}Cl_2N_7O_9P_6S_6Si_2]^{4+}$ 785.0674, found 785.0687 $[M-4Cl]^{4+}$; calcd. for $[C_{181}H_{221}ClN_7O_9P_6S_6Si_2]^{5+}$ 621.0600, found 621.0606 $[M-5Cl]^{5+}$; calcd. for $[C_{181}H_{221}N_7O_9P_6S_6Si_2]^{6+}$ 511.7218 (100 %), found 511.7223 $[M-6Cl]^{6+}$.

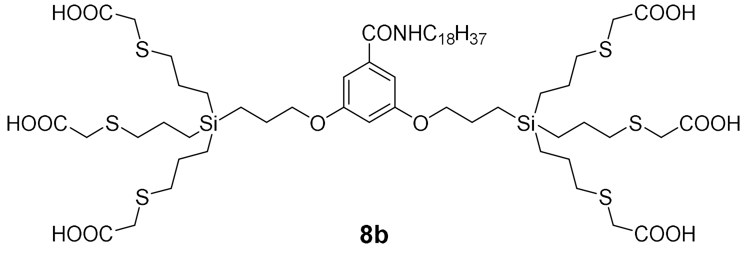

8b

Dendron 8b. Dendron **7b** (200 mg, 0.253 mmol), 2-mercaptoacetic acid (0.32 mL, 4.6 mmol) and DMPA (6 mg, 0.03 mmol) were dissolved in distilled THF (8 mL) in 10mL vial and deoxygenated. The reaction mixture was stirred in argon atmosphere and irradiated for 10 min. Then, DCM (75 mL) was added, the solution was washed with water (3 × 50 mL) and dried by anhydrous $MgSO_4$. The solvents

were removed to afford dendron **8b** (326 mg, 96 %, yellowish viscous substance). ^{1}H NMR (400 MHz, DMSO-d_6, ^{1}H-^{1}H COSY): δ 8.31 (t, J = 6.1 Hz, 1H, NH), 6.94 (d, J = 2.2 Hz, 2H, CH_{Ph}), 6.57 (t, J = 2.2 Hz, 1H, CH_{Ph}), 3.91 (t, J = 6.6 Hz, 4H, OCH_2), 3.17 (s, 14H, CH_2COOH, CH_2NH), 2.57 (t, J = 7.1 Hz, 12H, CH$_2$CH_2S), 1.71–1.64 (m, 4H, OCH$_2$CH_2), 1.56–1.47 (m, 14H, CH_2CH$_3$, CH_2CH$_2$S), 1.26–1.21 (m, 30H, CH_2), 0.83 (t, J = 6.6 Hz, 3H, Me), 0.65–0.59 (m, 16H, SiCH_2). ^{13}C {^{1}H} NMR (101 MHz, DMSO-d_6, ^{1}H-^{13}C HSQC, ^{1}H-^{13}C HMBC): δ 171.6 (COOH), 165.6 (CONH), 159.6 (C_qO), 136.7 (2$C_{q(Ph)}$), 105.5 (2CH$_{Ph}$), 103.5 (CH$_{Ph}$), 70.3 (OCH$_2$), overlapped with DMSO from HSQC 39.1 (CH$_2$NH), 34.5 (CH$_2$$CH_2$S), 33.2 ($CH_2$COOH), 31.3, 29.1 (2×$CH_2$), 29.03 (7$CH_2$), 29.02 (2$CH_2$), 28.97, 28.74, 28.72, 25.1 (4×CH$_2$), 23.3 (SiCH$_2$$CH_2$), 22.1 ($CH_2$), 14.0 ($Me$), 11.2 ($CH_2$Si), 7.7 (OCH$_2CH_2$$CH_2$). ^{29}Si {^{1}H} NMR (79 MHz, DMOS-d_6): δ 4.06. HRMS (ESI+): Calcd. for $[C_{61}H_{107}NO_{15}S_6Si_2Na]^+$ 1364.5396, found 1364.5380 $[M+Na]^+$.

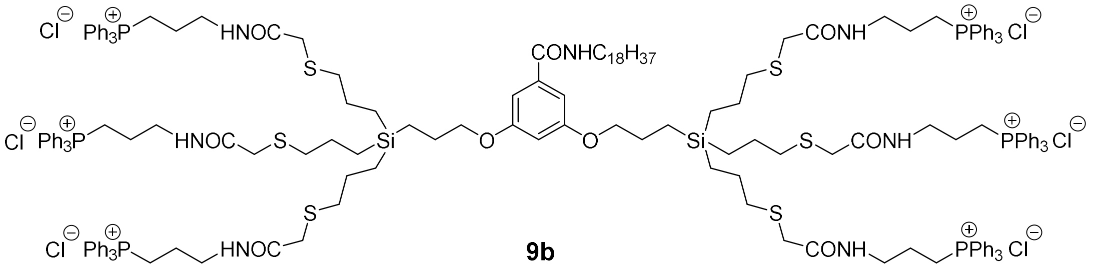

9b

Dendron DnP$_6$-1C$_{18}$ (9b). Dendron **8b** (303 mg, 0.226 mmol) and (3-ammoniumpropyl)triphenylphosphonium bromide (1.1 g, 2.0 mmol) were dissolved in dry DMF (15 mL) in argon atmosphere and stirred at 50 °C for 5 min. TBTU (508 mg, 1.58 mmol) was added in dry DMF (5 mL) and the solution was stirred at 50 °C for 15 min. DIPEA (0.63 mL, 3.6 mmol) was added and the reaction mixture was stirred at 50 °C for another 6 h. Then, the volume of solvent was reduced, and excess reagents were removed by nanofiltration in MeOH. Retentate was added to a column of ion-exchange resin Amberlite® IRA-400 in Cl$^-$ cycle and was slowly let to flow through the column. The product was washed from the column by additional MeOH, the volume of solvent was reduced, and the procedure was repeated once. Finally, the solvent was removed to afford dendron **9b** (676 mg, 87 %, white foam). ^{1}H NMR (400 MHz, DMSO-d_6, ^{1}H-^{1}H COSY): δ 8.71 (t, J = 5.2 Hz, 6H, NH), 8.58 (br s, 1H, NH), 7.89–7.73 (m, 90H, CH_{Ph}), 7.02 (br s, 2H, CH_{Ph}), 6.55 (br s, 1H, CH_{Ph}), 3.89 (br s, 4H, OCH_2), 3.75–3.68 (m, 12H, PCH_2), 3.22 (td, J = 6.4, 5.7 Hz, 12H, CH_2NH), 3.13 (s, 14H, SCH_2CO, CH_2NH), 2.53 (t, J = 6.8 Hz, 12H, SCH_2CH$_2$), 1.68–1.62 (m, 16H, PCH$_2$CH_2, OCH$_2$CH_2), 1.50–1.44 (m, 14H, CH_2CH$_2$S, CH_2CH$_3$), 1.21–1.19 (m, 30H, CH_2), 0.82 (t, J = 6.6 Hz, 3H, Me), 0.54–0.51 (m, 8H, SiCH_2). ^{13}C {^{1}H} NMR (101 MHz, DMSO-d_6, ^{1}H-^{13}C HSQC, ^{1}H-^{13}C HMBC): δ 169.4 (6CO), 165.4 (CO), 159.6 (C_qO), 136.6 (2$C_{q(Ph)}$), 134.9 (d, $^{4}J_{(C-P)}$ = 3.0 Hz, CH$_{Ph}$), 133.6 (d, $^{2}J_{(C-P)}$ = 10.1 Hz, CH$_{Ph}$), 130.2 (d, $^{3}J_{(C-P)}$ = 12.5 Hz, CH$_{Ph}$), 118.4 (d, $^{1}J_{(C-P)}$ = 86.0 Hz, C_qP), 105.6 (2CH$_{Ph}$), 103.6 (CH$_{Ph}$), 70.4 (OCH$_2$), overlapped with DMSO from HSQC 38.9 (CH$_2$CH$_2$CH$_2$P), overlapped with DMSO from HSQC 38.6, (CH$_2$NH), 35.5 (SCH$_2$CH$_2$), 34.4 (SCH$_2$CO), 31.3 (CH$_2$), 29.00, 28.99, 28.98 (3×3CH$_2$), 28.97, 28.9, 28.73, 28.67, 26.5 (5×CH$_2$), 23.4 (SCH$_2$$CH_2$), 23.3 (OCH$_2$$CH_2CH_2$), 22.2 (d, $^{2}J_{(C-P)}$ = 3.0 Hz, CH$_2$CH$_2$P), 22.1 (CH$_2$), 18.5 (d, $^{1}J_{(C-P)}$ = 51.7 Hz, CH$_2$P), 14.0 (Me), 11.2 (CH$_2$Si), 7.7 (OCH$_2$CH$_2$$CH_2$). ^{29}Si {^{1}H} NMR (79 MHz, DMSO-d_6): δ 3.91. ^{31}P {^{1}H} NMR (162 MHz, DMSO-d_6): δ 7.16. HRMS (ESI+): Calcd. for $[C_{187}H_{233}Cl_4N_7O_9P_6S_6Si_2]^{2+}$ 1647.1511, found 1647.1519 $[M-2Cl]^{2+}$; calcd. for $[C_{187}H_{233}Cl_3N_7O_9P_6S_6Si_2]^{3+}$ 1086.4442, found 1086.4460 $[M-3Cl]^{3+}$; calcd. for $[C_{187}H_{233}Cl_2N_7O_9P_6S_6Si_2]^{4+}$ 806.0908, found 806.0901 $[M-4Cl]^{4+}$; calcd. for $[C_{187}H_{233}ClN_7O_9P_6S_6Si_2]^{5+}$ 637.8788, found 637.8790 $[M-5Cl]^{5+}$; calcd. for $[C_{187}H_{233}N_7O_9P_6S_6Si_2]^{6+}$ 525.7374, found 525.7377 $[M-6Cl]^{6+}$.

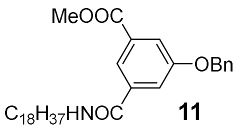

11

Methyl 3-(benzyloxy)-5-(octadecylaminocarbonyl)benzoate (**11**)**.** Benzoic acid **10** (500 mg, 1.75 mmol) and CDI (283 mg, 1.75 mmol) were dissolved in DCM (13 mL) and stirred at RT for 20 min. 1-octadecylamine (802 mg, 2.98 mmol) was added and the reaction mixture was stirred at RT for 6 h. Then, the solvent was removed, the residue was dissolved in EtOAc (60 mL) and 1M HCl (40 mL) was added. A white precipitate was filtered off using filter paper, the aqueous phase was separated and the organic phase further washed (40 mL H_2O, 2 × 40 mL 10% aq. sol. K_2CO_3, again 40 mL H_2O and finally 40 mL brine) and dried by anhydrous $MgSO_4$. The solvent was removed giving product **11** (887 mg, 94 %, white amorphous substance). ^{1}H NMR (400 MHz, $CDCl_3$, ^{1}H-^{1}H COSY): δ 7.91 (dd, J = 1.5, 1.3 Hz, 1H, CH_{Ph}), 7.76 (dd, J = 2.6, 1.3 Hz, 1H, CH_{Ph}), 7.69 (dd, J = 2.6, 1.5 Hz, 1H, CH_{Ph}), 7.45–7.34 (m, 5H, CH_{Bn}), 6.18 (t, J = 5.8 Hz, 1H, NH), 5.14 (s, 2H, C$H_{2(Bn)}$), 3.93 (s, 3H, MeO), 3.45 (td, J = 7.3, 5.8 Hz, 2H, NCH_2), 1.62 (p, J = 7.3 Hz, 2H, NCH_2CH_2), 1.39–1.25 (m, 30H, CH_2), 0.89–0.86 (m, 3H, Me). ^{13}C {^{1}H} NMR (101 MHz, $CDCl_3$, ^{1}H-^{13}C HSQC, ^{1}H-^{13}C HMBC): δ 166.4, 166.3 (2×CO), 159.2, 136.7 (2×C_q), 136.3 ($C_{q(Bn)}$), 131.8 (C_q), 128.8 (2CH$_{Bn}$), 128.4 (CH$_{Bn}$), 127.8 (2CH$_{Bn}$), 119.7, 118.8, 118.6 (3×CH$_{Ph}$), 70.6 (CH$_{2(Bn)}$), 52.6 (MeO), 40.4 (NCH$_2$), 32.1 (CH$_2$), 29.9 (5CH$_2$), 29.82 (CH$_2$), 29.80 (2CH$_2$), 29.77, 29.75, 29.7, 29.51, 29.47, 27.2, 22.8 (7×CH$_2$), 14.3 (Me). HRMS (APCI+): Calcd. for $[C_{34}H_{52}NO_4]^+$ 538.3890, found 538.3889 $[M+H]^+$.

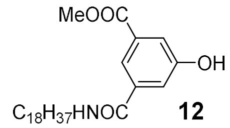

12

Methyl 3-hydroxy-5-(octadecylaminocarbonyl)benzoate (**12**)**.** Benzoate **11** (997 mg, 1.85 mmol) and 10% palladium on active carbon (158 mg, 0.148 mmol) were mixed with MeOH (18 mL) and distilled THF (18 mL) and the mixture was stirred in hydrogen atmosphere at 45 °C for 6 h. Then, palladium catalyst was filtered off using Celite and the filtrate was evaporated to dryness. The solid residue was dispersed in Et_2O, applied on top of a short column of silicagel (20 g) and eluted by 200 mL of Et_2O. The solvent was removed affording product **12** (628 mg, 75 %, white powder). ^{1}H NMR (400 MHz, THF-d_8, ^{1}H-^{1}H COSY): δ 8.85 (s, 1H, OH), 7.88 (dd, J = 1.5, 1.4 Hz, 1H, CH_{Ph}), 7.76 (t, J = 5.7 Hz, 1H, NH), 7.52 (dd, J = 2.6, 1.5 Hz, 1H, CH_{Ph}), 7.48 (dd, J = 2.6, 1.4 Hz, 1H, CH_{Ph}), 3.85 (s, 3H, COOMe), 3.35 (td, J = 7.3, 5.7 Hz, 2H, NCH_2), 1.58 (p, J = 7.3 Hz, 2H, NCH_2CH_2),1.40–1.26 (m, 30 H, CH_2), 0.88 (t, J = 1.5 Hz, 3H, Me). ^{13}C {^{1}H} NMR (101 MHz, THF-d_8, ^{1}H-^{13}C HSQC, ^{1}H-^{13}C HMBC): δ 166.8 (COOMe), 166.1 (CO), 158.8 (OC_q), 132.3, 138.3 (2×$C_{q(Ph)}$), 120.1, 119.3, 119.1 (3×CH$_{Ph}$), 52.2 (COOMe), 40.7 (NCH$_2$), 32.9, 30.8 (2×CH$_2$), 30.70 (8CH$_2$), 30.68 (2CH$_2$), 30.65, 30.4, 28.1, 23.6 (4×CH$_2$), 14.5 (Me). HRMS (APCI+): Calcd. for $[C_{27}H_{46}NO_4]^+$ 448.3421, found 448.3420 $[M+H]^+$.

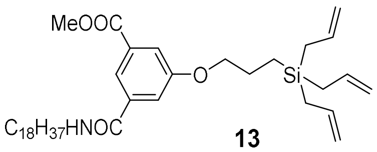

13

Dendron 13. Benzoate **12** (612 mg, 1.37 mmol) and calcinated K_2CO_3 (284 mg, 2.06 mmol) were mixed with dry MeCN (25 mL) in argon atmosphere and stirred at 60 °C for 20 min. Triallyl(3-iodopropyl)silane (482 mg, 1.50 mmol) was added and the reaction mixture was stirred at reflux temperature for another 48 h. Then, the solvent was removed and the residue was purified using column chromatography (eluent petroleum ether (b.p. 40 – 65 °C)/Et_2O 3:1) affording product **13** (R_f = 0.30,

486 mg, 55 %, white amorphous solid). ^{1}H NMR (400 MHz, CDCl$_3$*d*, ^{1}H-^{1}H COSY): δ 7.87 (dd, J = 1.5, 1.3 Hz, 1H, CH_{Ph}), 7.65 (dd, J = 2.6, 1.3 Hz, 1H, CH_{Ph}), 7.58 (dd, J = 2.6, 1.5 Hz, 1H, CH_{Ph}), 6.18 (t, J = 5.8 Hz, 1H, NH), 5.80 (ddt, J = 16.5, 10.1, 8.1 Hz, 3H, CHCH$_2$), 4.93–4.87 (m, 6H, CHCH_2), 3.99 (t, J = 6.6 Hz, 4H, OCH_2), 3.93 (s, 3H, COOMe), 3.45 (td, J = 7.3, 5.8 Hz, 2H, NCH_2), 1.87–1.80 (m, 2H, OCH$_2$CH_2), 1.63 (dt, J = 8.1, 1.2 Hz, 6H, SiCH_2CH), 1.60 (br s, 2H, NCH$_2$CH_2), 1.38–1.21 (m, 30H, CH_2), 0.88 (t, J = 6.8 Hz, 3H, Me), 0.74–0.70 (m, 2H, SiCH_2CH$_2$). ^{13}C {^{1}H} NMR (101 MHz, CDCl$_3$-*d*, ^{1}H-^{13}C HSQC, ^{1}H-^{13}C HMBC): δ 166.5, 166.4 (2 × *C*O), 159.5 (OC_q), 136.6 ($C_{q(Ph)}$), 134.2 (*C*HCH$_2$), 131.7 ($C_{q(Ph)}$), 119.2 (CH$_{Ph}$), 118.4 (2CH$_{Ph}$), 114.0 (CH*C*H$_2$), 71.1 (O*C*H$_2$), 52.6 (COOMe), 40.4 (N*C*H$_2$), 32.1 (*C*H$_2$), 29.84 (5*C*H$_2$), 29.82, 29.80, 29.79, 29.78, 29.75, 29.7, 29.50, 29.47, 27.2 (9 × *C*H$_2$ octadecyl), 23.4 (OCH$_2$*C*H$_2$), 22.8 (*C*H$_2$), 19.7 (Si*C*H$_2$CH), 14.3 (Me), 7.7 (Si*C*H$_2$CH$_2$). ^{29}Si {^{1}H} NMR (79 MHz, CDCl$_3$-*d*): δ –0.12. HRMS (APCI+): Calcd. for $[C_{39}H_{66}NO_4Si]^+$ 640.4756, found 640.4758 $[M+H]^+$.

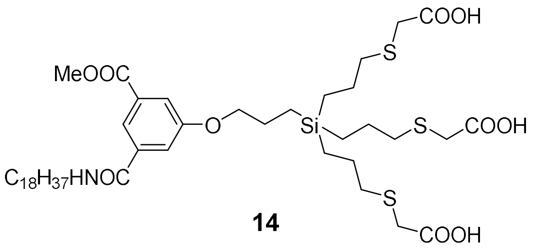

14

Dendron 14. Dendron **13** (173 mg, 0.270 mmol), 2-mercaptoacetic acid (0.22 g, 2.4 mmol, 0.17 mL) and DMPA (7 mg, 0.027 mmol) were dissolved in distilled THF (6 mL) in 10mL vial and deoxygenated. The reaction mixture was stirred in argon atmosphere and irradiated for 10 min. Then, DCM (50 mL) was added and the mixture was washed with water (3 × 30 mL). The organic layer was dried by anhydrous $MgSO_4$ and the solvent was evaporated at 40 °C. The residue was dried on a lyophilizer to afford dendron **14** (241 mg, 98 %, colorless viscous liquid). ^{1}H NMR (400 MHz, DMSO-d_6, ^{1}H-^{1}H COSY): δ 8.60 (t, J = 5.6 Hz, 1H, NH), 8.01, 7.64, 7.54 (3×s, 3×1H, CH_{Ph}), 4.02 (t, J = 6.3 Hz, 2H, C$_q$OCH_2), 3.18 (s, 3H, OMe), 3.26 (s, 6H, SCH_2COO), 3.24 (td, J = 7.5, 5.6 Hz, 2H, CH_2NH), 3.18 (s, 6H, SCH_2COOH), 2.58 (t, J = 7.2 Hz, 6H, SCH_2CH$_2$), 1.76–1.70 (m, 2H, OCH$_2$CH_2), 1.58–1.49 (m, 8H, CH_2CH$_2$S, CH_2CH$_2$NH), 1.26–1.21 (m, 30H, CH_2), 0.84 (t, J = 6.6 Hz, 3H, Me), 0.67–0.60 (m, 8H, SiCH_2). ^{13}C {^{1}H} NMR (101 MHz, DMSO-d_6, ^{1}H-^{13}C HSQC, ^{1}H-^{13}C HMBC): δ 171.6 (3*C*O), 165.7 (*C*ONH), 164.7 (*C*OOMe), 158.7 (C_qO), 136.8, 131.0 (2×$C_{q(Ph)}$), 120.1, 118.2, 116.8 (3×CH$_{Ph}$), 70.6 (O*C*H$_2$), 52.4 (OMe), overlapped with DMSO from HSQC 39.3 (*C*H$_2$NH), 35.5 (S*C*H$_2$CH$_2$), 33.2 (S*C*H$_2$CO), 31.3 (*C*H$_2$), 29.04 (2*C*H$_2$), 29.02 (6*C*H$_2$), 28.9 (2*C*H$_2$), 28.7, 26.4 (2×2*C*H$_2$), 23.3 (SCH$_2$*C*H$_2$), 23.2 (SiCH$_2$*C*H$_2$), 22.1 (*C*H$_2$), 14.0 (Me), 11.2 (*C*H$_2$Si), 7.7 (OCH$_2$CH$_2$*C*H$_2$). ^{29}Si {^{1}H} NMR (79 MHz, DMSO-*d$_6$*): δ 4.08. HRMS (ESI-): Calcd. for $[C_{45}H_{76}NO_{10}S_3Si]^-$ 914.4406, found 914.4402 $[M-H]^-$.

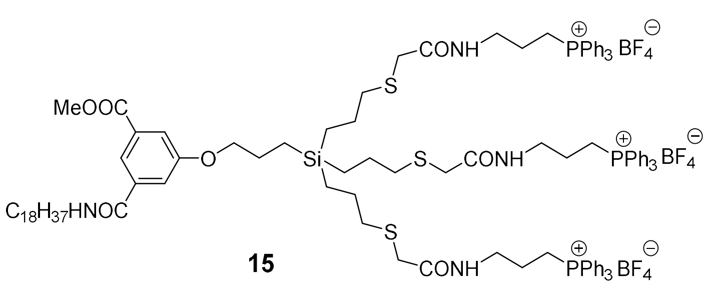

15

Dendron 15. Dendron **14** (241 mg, 0.263 mmol) and (3-ammoniumpropyl)triphenylphosphonium bromide (0.76 g, 1.6 mmol) were dissolved in dry DMF (12 mL) in argon atmosphere and stirred for 5 min. TBTU (507 mg, 1.58 mmol) in dry DMF (12 mL) was added and the solution was stirred for 15

min. DIPEA (0.41 mL, 2.4 mmol) was added and the reaction mixture was stirred at RT for another 24 h. Then, the volume was reduced to 2 mL, DCM was added (100 mL) and the solution was washed (2 × 70 mL 10 % aq. citric acid, 2 × 70 mL H_2O) and dried by anhydrous $MgSO_4$. The solvent was removed and the residue was purified by nanofiltration in a mixture of DCM and MeOH 1:1 giving dendron **15** (484 mg, 88 %, white foam). ^{1}H NMR (400 MHz, DMSO-d_6, ^{1}H-^{1}H COSY): δ 8.72 (m, 1H, N*H*), 8.44 (t, *J* = 5.7 Hz, 3H, N*H*), 8.02 (s, 1H, CH_{Ph}), 7.91–7.73 (m, 45H, CH_{Ph}), 7.68, 7.52 (2×s, 2×1H, CH_{Ph}), 4.00 (t, *J* = 6.4 Hz, 2H, C_qOCH_2), 3.85 (s, 3H, O*Me*), 3.67–3.58 (m, 6H, PCH_2), 3.22 (td, *J* = 6.8, 5.7 Hz, 8H, CH_2NH), 3.10 (s, 6H, SCH_2CO), 2.52 (t, *J* = 5.6 Hz, CH_2CH_2S), 1.67–1.65 (m, 8H, OCH_2CH_2, CH_2CH_2P), 1.49–1.43 (m, 8H, $NHCH_2CH_2$, CH_2CH_2S), 1.25–1.21 (m, 30H, CH_2), 0.84 (t, *J* = 6.7 Hz, 3H, *Me*), 0.61–0.52 (m, 8H, SiCH_2). ^{13}C {^{1}H} NMR (101 MHz, DMSO-d_6, ^{1}H-^{13}C HSQC, ^{1}H-^{13}C HMBC): δ 169.4 (3*C*O), 165.7 (*C*OOMe), 164.7 (*C*ONH), 158.6 (C_qO), 136.5 ($C_{q(Ph)}$), 135.0 (d, $^4J_{(C\text{-}P)}$ = 2.9 Hz, CH_{Ph}), 133.6 (d, $^2J_{(C\text{-}P)}$ = 9.9 Hz, CH_{Ph}), 131.0 ($C_{q(Ph)}$), 130.3 (d, $^3J_{(C\text{-}P)}$ = 12.5 Hz, CH_{Ph}), 120.1 (CH_{Ph}), 118.3 (d, $^1J_{(C\text{-}P)}$ = 86.0 Hz, $C_{q(Ph)}$), 118.0, 116.8 (2×CH_{Ph}), 70.6 (OCH_2), 52.4 (O*Me*), overlapped with DMSO from HSQC 38.8 (CH_2NH), 35.5 (CH_2CH_2S), 34.4 (SCH_2CO), 31.3, 29.01 (2×CH_2), 29.00, 28.99, 28.98 (3×2CH_2), 28.93 (4CH_2), 28.70, 28.68, 26.4 (3×CH_2), 23.4 (SCH_2CH_2), 23.1 (OCH_2CH_2), 22.2 (d, $^2J_{(C\text{-}P)}$ = 2.6 Hz, CH_2CH_2P), 22.1 (CH_2), 18.4 (d, $^1J_{(C\text{-}P)}$ = 52.0 Hz, CH_2P), 13.9 (*Me*), 11.2 ($SCH_2CH_2CH_2$), 7.6 ($OCH_2CH_2CH_2$). ^{29}Si {^{1}H} NMR (79 MHz, DMSO-d_6): δ 4.06. ^{31}P {^{1}H} NMR (162 MHz, DMSO-d_6): δ 7.16. HRMS (ESI+): Calcd. for $[C_{108}H_{140}BF_4N_4O_7P_3S_3Si]^{3+}$ 954.4442, found 954.4439 $[M\text{-}2BF_4]^{2+}$; calcd. for $[C_{108}H_{140}N_4O_7P_3S_3Si]^{3+}$ 607.2950, found 607.2957 $[M\text{-}3BF_4]^{3+}$.

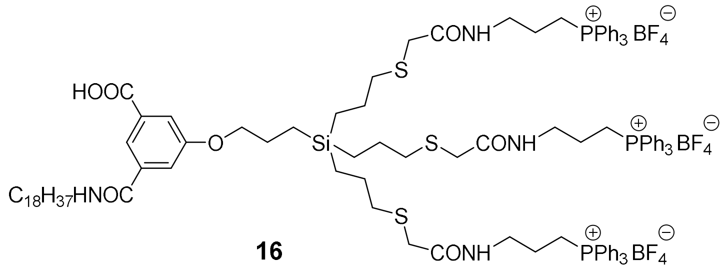

16

Dendron 16. Dendron **15** (100 mg, 48.0 μmol) and NaOH (11.3 mg, 282 μmol) were dissolved in MeOH (3 mL), a drop of water was added and the reaction mixture was stirred for 20 h at 55 °C. Then, DCM (40 mL) was added, the solution was washed (2 × 20 mL 10 % aq. citric acid, 2 × 20 mL H_2O) and dried by anhydrous $MgSO_4$. The solvent was removed giving dendron **16** (75 mg, 75 %, white foam). ^{1}H NMR (400 MHz, DMSO-d_6, ^{1}H-^{1}H COSY): δ 8.71 (m, 1H, N*H*), 8.58 (br s, 3H, N*H*), 8.01 (s, 1H, CH_{Ph}), 7.90–7.74 (m, 45H, CH_{Ph}), 7.65, 7.51 (2×s, 2×1H, CH_{Ph}), 3.99 (t, *J* = 6.1 Hz, 2H, C_qOCH_2), 3.71–3.62 (m, 6H, PCH_2), 3.24–3.19 (m, 8H, CH_2NH), 3.11 (s, 6H, SCH_2CO), 2.50 (t, *J* = 6.2 Hz, CH_2CH_2S), 1.70–1.64 (m, 8H, OCH_2CH_2, CH_2CH_2P), 1.52–1.44 (m, 8H, $NHCH_2CH_2$, CH_2CH_2S), 1.27–1.21 (m, 30H, CH_2), 0.83 (t, *J* = 6.7 Hz, 3H, *Me*), 0.60–0.51 (m, 8H, SiCH_2). ^{13}C {^{1}H} NMR (101 MHz, DMSO-d_6, ^{1}H-^{13}C HSQC, ^{1}H-^{13}C HMBC): δ 169.4 (3*C*O), 166.8 (*C*OOH), 164.9 (*C*ONH), 158.6 (C_qO), 136.3 ($C_{q(Ph)}$), 134.9 (d, $^4J_{(C\text{-}P)}$ = 2.9 Hz, CH_{Ph}), 133.6 (d, $^2J_{(C\text{-}P)}$ = 10.1 Hz, CH_{Ph}), 130.4 ($C_{q(Ph)}$), 130.3 (d, $^3J_{(C\text{-}P)}$ = 12.4 Hz, CH_{Ph}), 120.2 (CH_{Ph}), 118.3 (d, $^1J_{(C\text{-}P)}$ = 85.9 Hz, $C_{q(Ph)}$), 117.5, 117.0 (2×CH_{Ph}), 70.6 (OCH_2), overlapped with DMSO from HSQC 38.9 (CH_2NH), 35.5 (CH_2CH_2S), 34.4 (SCH_2CO), 31.3, 29.02 (2×CH_2), 29.01 (2CH_2), 29.00, 28.95 (2×4CH_2), 28.72, 28.69, 26.5 (3×CH_2), 23.4 (SCH_2CH_2), 23.2 (OCH_2CH_2), 22.2 (d, $^2J_{(C\text{-}P)}$ = 3.8 Hz, CH_2CH_2P), 22.1 (CH_2), 18.5 (d, $^1J_{(C\text{-}P)}$ = 51.7 Hz, CH_2P), 14.0 (*Me*), 11.2 ($SCH_2CH_2CH_2$), 7.7 ($OCH_2CH_2CH_2$). ^{29}Si {^{1}H} NMR (79 MHz, DMSO-d_6): δ 3.95. ^{31}P {^{1}H} NMR (162 MHz, DMSO-d_6): δ 7.16. ^{19}F {^{1}H} NMR (374 MHz, DMSO-d_6): δ –149.53 (br s, $^{10}BF_4$); –149.59 (q, $^1J_{(F\text{-}B)}$ = 1.2 Hz, $^{11}BF_4$). HRMS (ESI+): Calcd. for $[C_{107}H_{138}BF_4N_4O_7P_3S_3Si]^{3+}$ 947.4364, found 947.4341 $[M\text{-}2BF_4]^{2+}$; calcd. for $[C_{107}H_{137}N_4O_7P_3S_3Si]^{2+}$ 903.4310, found 903.4313 $[M\text{-}HBF_4\text{-}2BF_4]^{2+}$; calcd. for $[C_{107}H_{138}N_4O_7P_3S_3Si]^{3+}$ 602.6231, found 602.6229 $[M\text{-}3BF_4]^{3+}$.

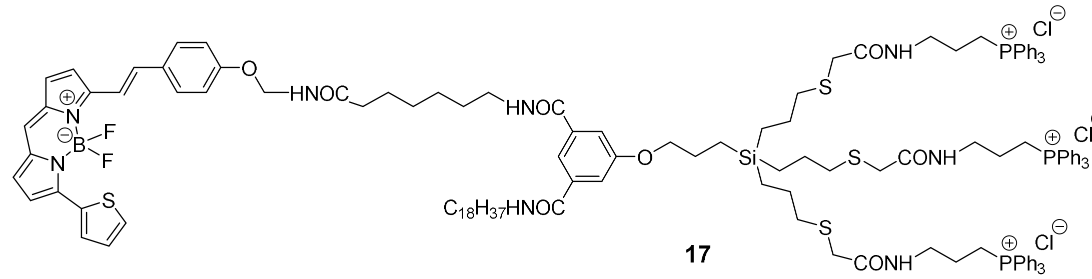

17

Dendron DnP_3-$1C_{18}$/BDP (17). Dendron **16** (16.1 mg, 7.77 µmol) and BODIPY 630/650 amine (6.8 mg, 12 µmol) were dissolved in dry DMF (2 mL) in argon atmosphere and the solution was stirred at RT for 5 min. TBTU (3.7 mg, 12 µmol) in dry DMF (0.5 mL) was added and the solution stirred at RT for 10 min. DIPEA (4.1 µL, 23 µmol) was added using Hamilton syringe and the reaction mixture was stirred at RT for 2 h. Then, the volume of solvent was reduced and excess reagents were removed by nanofiltration in a mixture of DCM and MeOH 10:1. The retentate was dissolved in DCM/MeOH 2:3 eluent (5 mL) and passed through a column of ion-exchange resin Amberlite® IRA-400 in Cl^- cycle. The product was washed from the column by the eluent, the volume of solvents was reduced at 40 °C and the procedure was repeated once. Then, the solvents were removed, deionized water was added (1 mL), the solution was frozen, cooled to –40 °C and lyophilized. Dendron **17** was obtained as an intensively blue powder (11.5 mg, 60 %). ^{1}H NMR (400 MHz, DMSO-d_6, ^{1}H-^{1}H COSY): δ 8.64 (m, 2H, NH), 8.40 (m, 3H, NH), 8.35 (1H, NH), 8.03 (d, J = 3.8 Hz, 1H, CH), 7.89–7.72 (m, 47H, CH, 45CH_{Ph}), 7.61–7.60 (m, 3H, CH, 2CH_{Ph}), 7.52–7.48 (m, 2H, CH), 7.37–7.35 (m, 2H, CH), 7.29–7.24 (m, 4H, CH_{Ph}), 7.07–7.05 (m, 2H, CH), 6.94 (d, J = 4.2 Hz, 1H, CH_{Ph}), 4.54 (s, 2H, COCH_2O), 3.97 (br s, 2H, OCH_2), 3.59 (br s, 6H, PCH_2), 3.23–3.21 (m, 6H, CH_2CH$_2$CH$_2$P), 3.15–3.11 (m, 4H, CH_2NH), 3.09 (s, 6H, SCH_2CO), 3.05–3.02 (2H, CH_2NH), overlapped with DMSO 2.53 (6H, CH$_2$CH_2S), 1.66 (br s, 6H, PCH$_2$CH_2), 1.48–1.43 (m, 14H, NHCH$_2$CH_2, SiCH$_2$CH_2), 1.29–1.19 (m, 34H, CH_2), 0.83 (t, J = 3.8 Hz, 3H, Me), 0.56 (br s, 8H, SiCH_2). ^{13}C {^{1}H} NMR (101 MHz, DMSO-d_6, ^{1}H-^{13}C HSQC, ^{1}H-^{13}C HMBC): δ 169.4 (3CO), 167.1 (2CO), 165.4 (CO), 159.4 (C_qO), 158.5 (C_qO), not detected (C_q), 156.4, 147.6 (2×C_q), 138.9 (CH), 136.5, 136.3, 136.1 (3×C_q), 135.0 (d, $^{4}J_{(C\text{-}P)}$ = 2.9 Hz, CH$_{Ph}$), 133.7 (C_q), 133.6 (d, $^{2}J_{(C\text{-}P)}$ = 10.1 Hz, CH$_{Ph}$), 131.6, 131.3, 130.4 (3×CH), 130.3 (d, $^{3}J_{(C\text{-}P)}$ = 12.4 Hz, CH$_{Ph}$), 129.6 (CH), 129.1 (2CH$_{Ph}$), 128.9, 125.2, 119.4, 118.7, (4×CH), 118.32 (d, $^{1}J_{(C\text{-}P)}$ = 86.2 Hz, $C_{q(Ph)}$), 118.26, 115.9 (2×CH), 115.6 (4CH$_{Ph}$), 70.5 (C$_q$OCH$_2$), 67.0 (COCH$_2$O), 38.7 (d, $^{3}J_{(C\text{-}P)}$ = 13.6 Hz, CH$_2$CH$_2$CH$_2$P), 38.4 (CH$_2$NH), 38.2 (2CH$_2$NH), 35.6 (CH$_2$CH$_2$$CH_2$S), 34.5 (S$CH_2$CO), 31.3, 29.8 (2×$CH_2$), 29.03, 29.02, 29.00, 28.96 (4×3CH$_2$), 28.8, 28.7, 26.5, 26.4, 26.1 (5×CH$_2$), 23.4 (CH$_2$CH$_2$Si), 22.2 (d, $^{2}J_{(C\text{-}P)}$ = 4.2 Hz, CH$_2$CH$_2$P), 22.1 (CH$_2$), 18.4 (d, $^{1}J_{(C\text{-}P)}$ = 51.7 Hz, CH$_2$P), 14.0 (Me), 11.2 (CH$_2$CH$_2$CH$_2$S), 7.8 (CH$_2$Si). ^{29}Si {^{1}H} NMR (79 MHz, DMSO-d_6): δ 3.93. ^{31}P {^{1}H} NMR (162 MHz, DMSO-d_6): δ 7.14. ^{19}F NMR (376 MHz, DMSO-d_6): δ –139.12 (q, $^{1}J_{(F\text{-}B)}$ = 32.8 Hz, 2F). HRMS (ESI+): Calcd. for $[C_{136}H_{167}ClBF_2N_8O_8P_3S_4Si]^{2+}$ 1186.5265, found 1186.5271 $[M\text{-}2Cl]^{2+}$; calcd. for $[C_{136}H_{167}BF_2N_8O_8P_3S_4Si]^{3+}$ 779.3605, found 779.3619 $[M\text{-}3Cl]^{3+}$.

2. CMC estimation

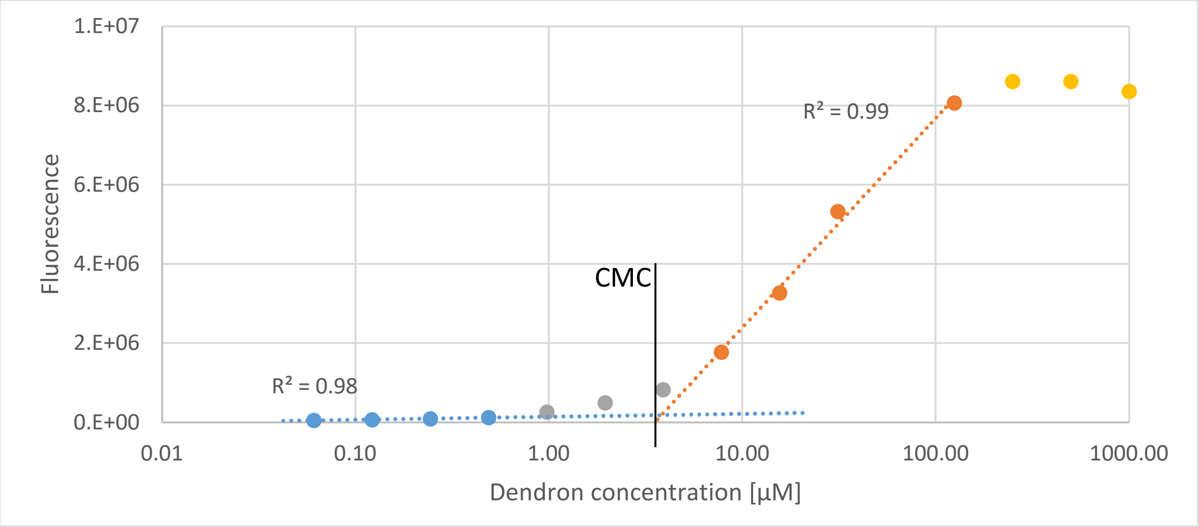

Figure S1. CMC estimation for dendron **DnP$_6$-1C$_{12}$** (**9a**)

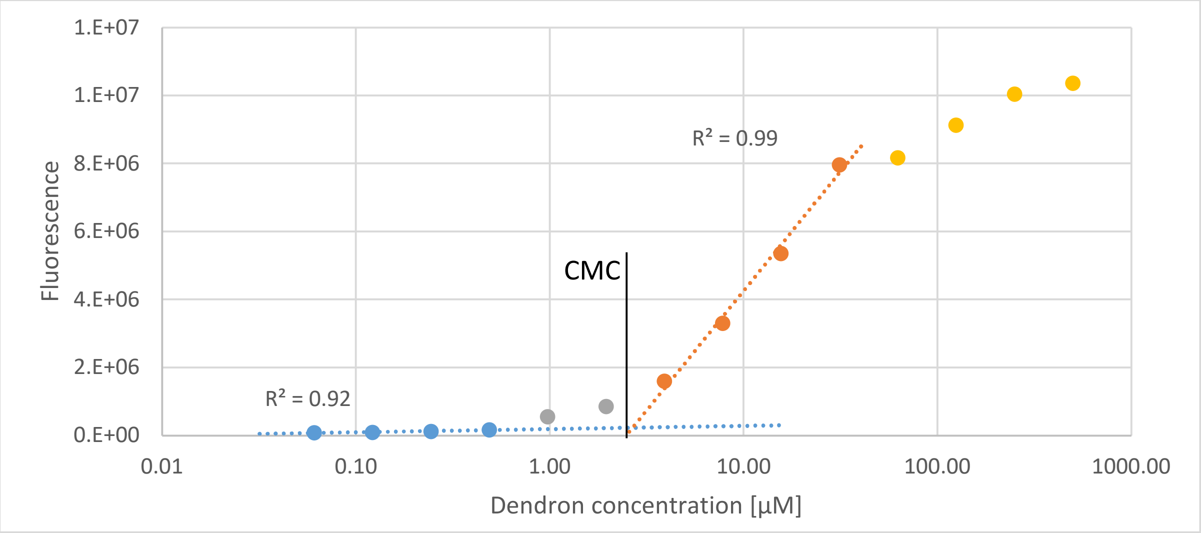

Figure S2. CMC estimation for dendron **DnP$_6$-1C$_{18}$** (**9b**)

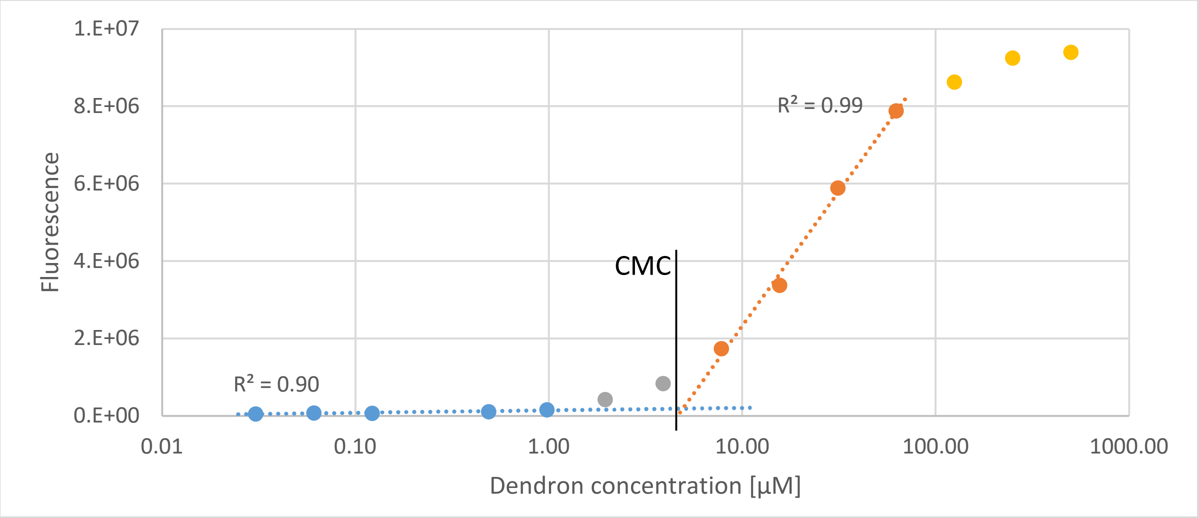

Figure S3. CMC estimation for dendron **DnP$_3$-1C$_{12}$** (**6a**)

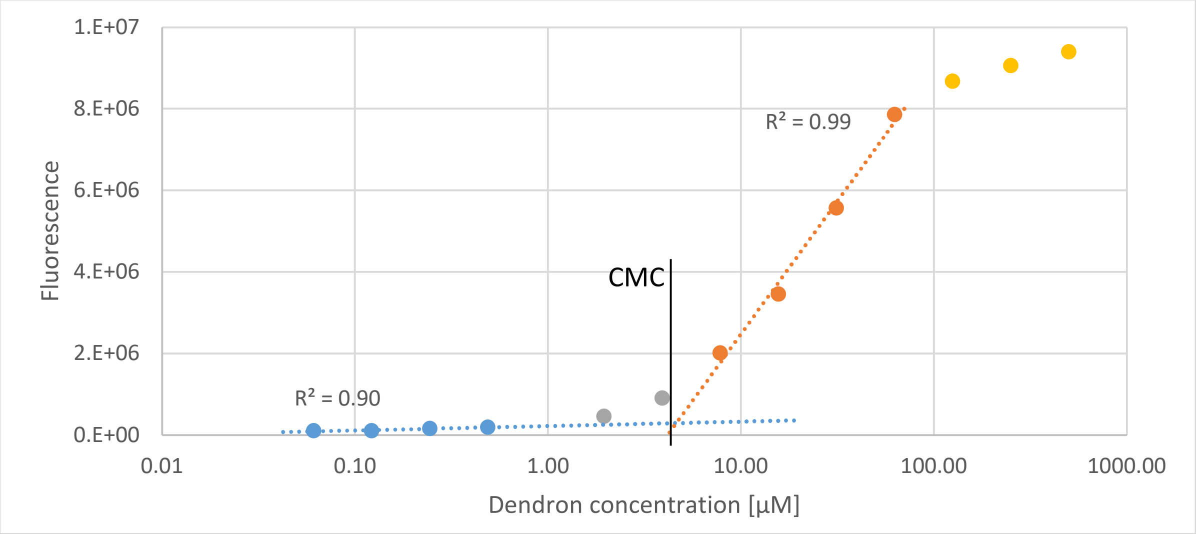

Figure S4. CMC estimation for dendron **DnP$_3$-1C$_{18}$ (6b)**

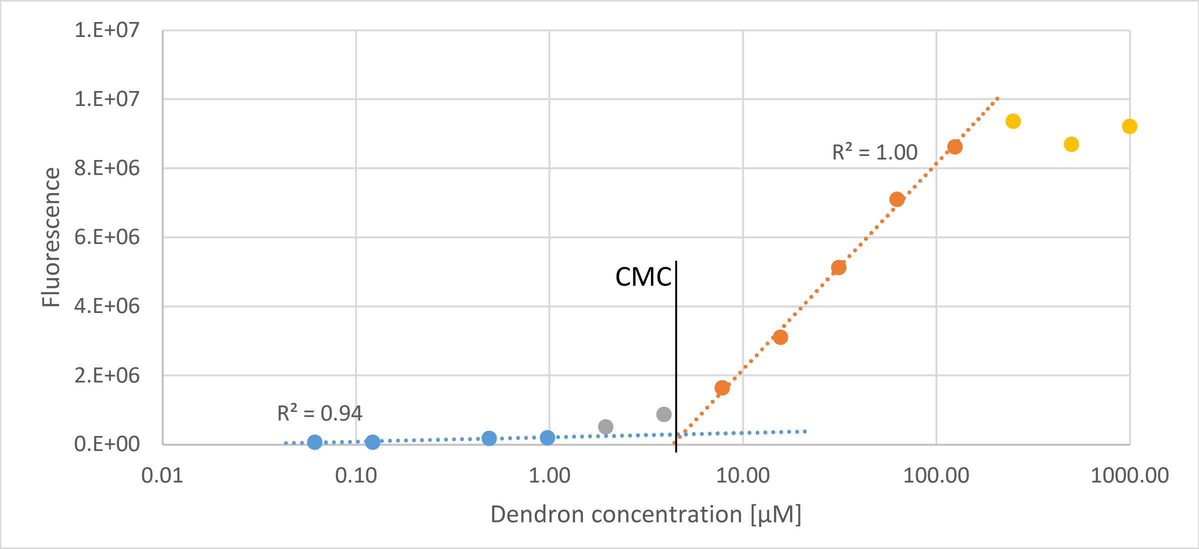

Figure S5. CMC estimation for dendron **DnP$_3$-2C$_{12}$ (3a)**

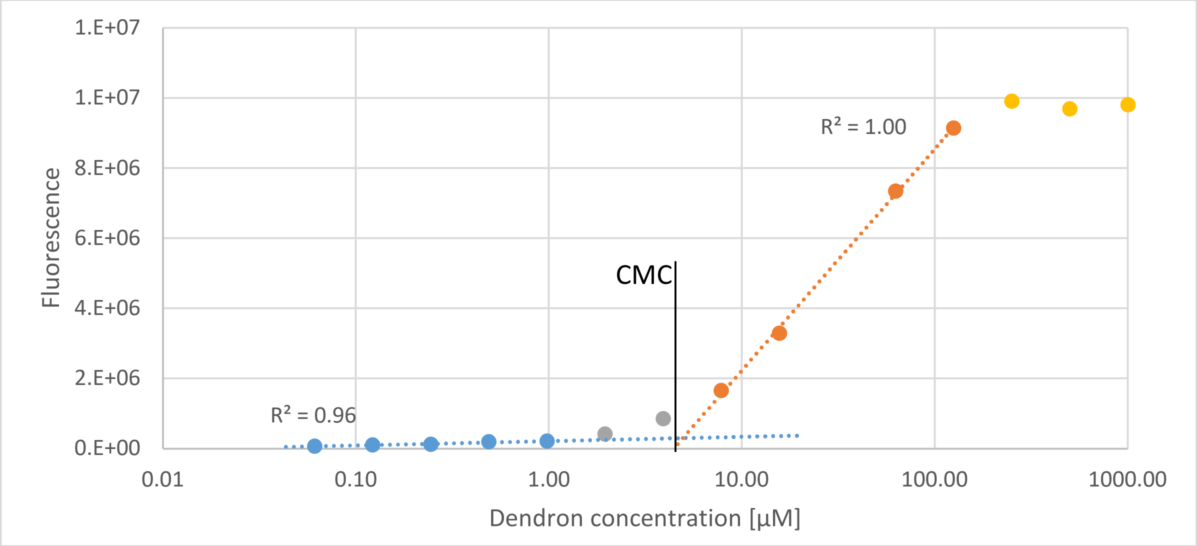

Figure S6. CMC estimation for dendron **DnP$_3$-2C$_{18}$ (3b)**

3. Summary of supramolecular properties

Table S1. CMC values, hydrodynamic diameters from DLS (d_h) and average biggest dimensions (D_{max}) of nanoassemblies of amp-DDNs obtained by molecular simulations in physiological solution.

Dendron	CMC [µM]	d_h [nm]	D_{max} [nm]
**DnP6-1C12**	3.8	5.8	4.6
**DnP6-1C18**	2.6	6.9	5.1
**DnP3-1C12**	4.9	7.8	5.5
**DnP3-1C18**	4.6	9.9	7.2
**DnP3-2C12**	4.8	10.0[a]	6.5
**DnP3-2C18**	4.9	11.8[a]	8.6

[a] value determined by MADLS analysis after stabilization of populations, mean diameter of the population of smaller nanoparticles is given

4. Micelle size and stability measurements

Table S2. Time dependent mean sizes of nanoparticles determined by DLS (Nanobrook Omni), measured at concentration 1.0 mM of dendron in physiological solution (0.9 % NaCl in DI water).

Dendron	d[a] [nm] (PDI[b])				
	1 day	2 days	5 days	18 days	75 days
**DnP6-1C12**	5.8 ± 0.2 (0.04)	-	-	5.8 ± 0.1 (0.07)	5.7 ± 0.1 (0.07)
**DnP6-1C18**	6.9 ± 0.1 (0.07)	-	-	6.8 ± 0.1 (0.06)	6.9 ± 0.2 (0.09)
**DnP3-1C12**	7.8 ± 0.2 (0.06)	-	-	8.0 ± 0.1 (0.03)	7.8 ± 0.1 (0.07)
**DnP3-1C18**	9.9 ± 0.2 (0.13)	-	-	9.6 ± 0.2 (0.11)	9.5 ± 0.1 (0.03)
**DnP3-2C12**	21.0 ± 0.6 (0.21)	23 ± 3 (0.20)	21.1 ± 0.4 (0.22)	23.0 ± 0.3 (0.21)	24.8 ± 0.1 (0.19)
**DnP3-2C18**	14.1 ± 1.1 (0.16)	16.0 ± 0.5 (0.21)	18.2 ± 0.3 (0.21)	24.8 ± 0.1 (0.23)	26.2 ± 0.2 (0.22)

[a] average value from 5 independent measurements, selected results are shown in Figures S7-S12; [b] standard deviation of PDI never exceeded 0.03

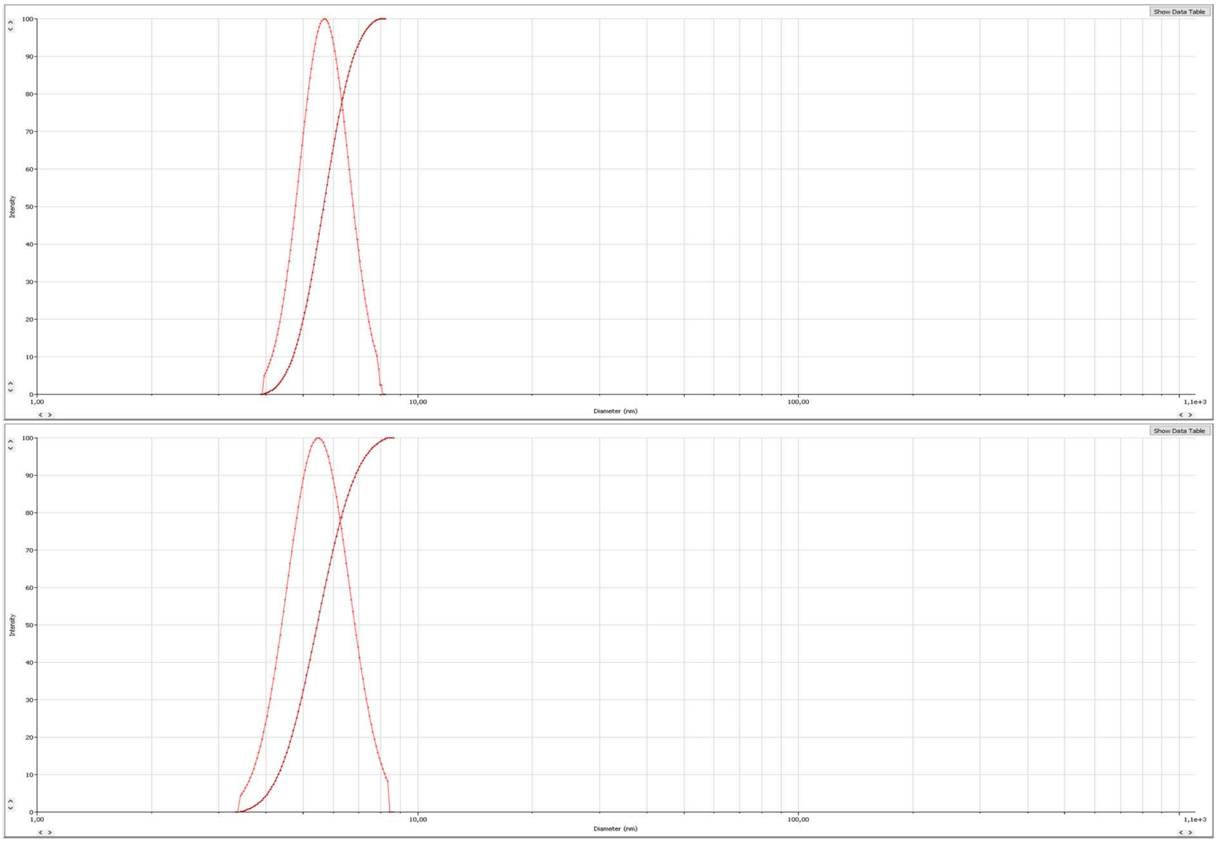

Figure S7. Intensity-weighted size distribution in the sample of dendron **DnP$_6$-1C$_{12}$** (**9a**) at day 1 (top) and day 75 (bottom) as determined by DLS

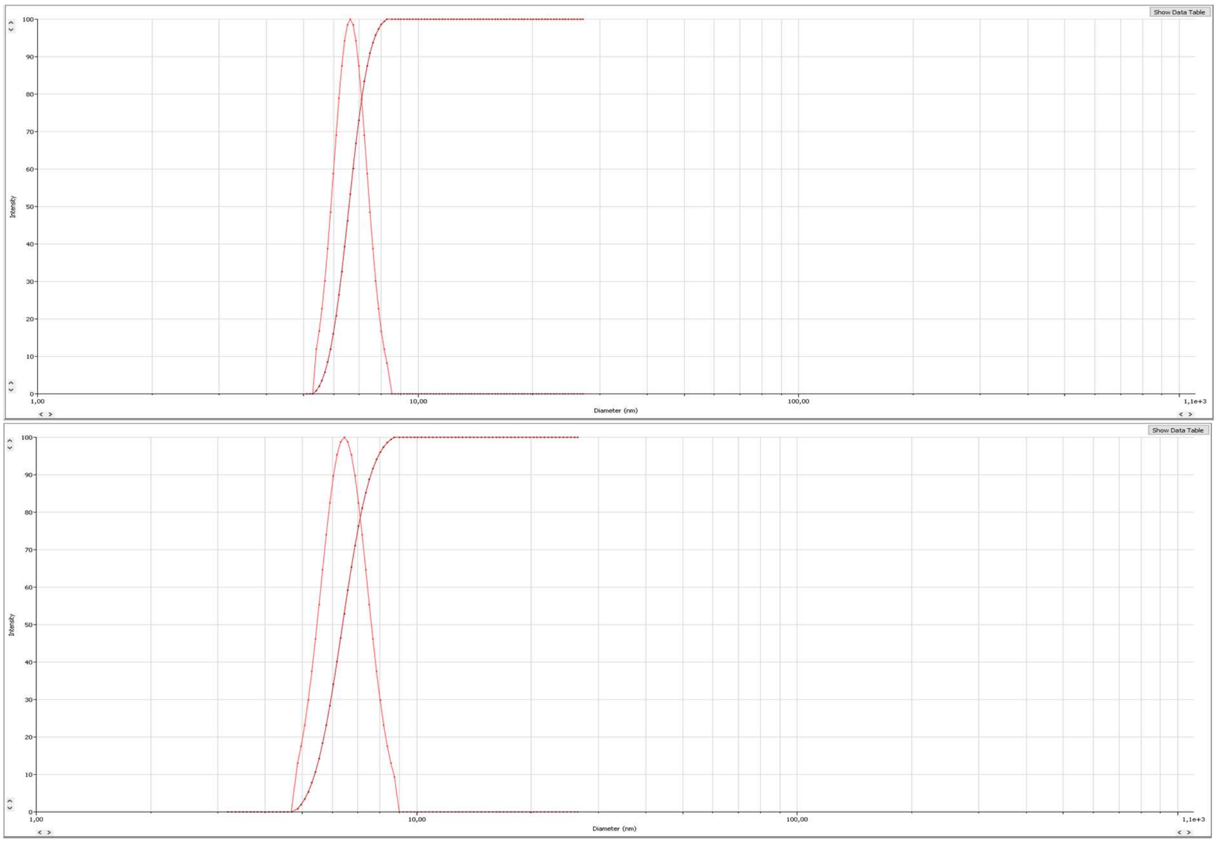

Figure S8. Intensity-weighted size distribution in the sample of dendron **DnP$_6$-1C$_{18}$** (**9b**) at day 1 (top) and day 75 (bottom) as determined by DLS

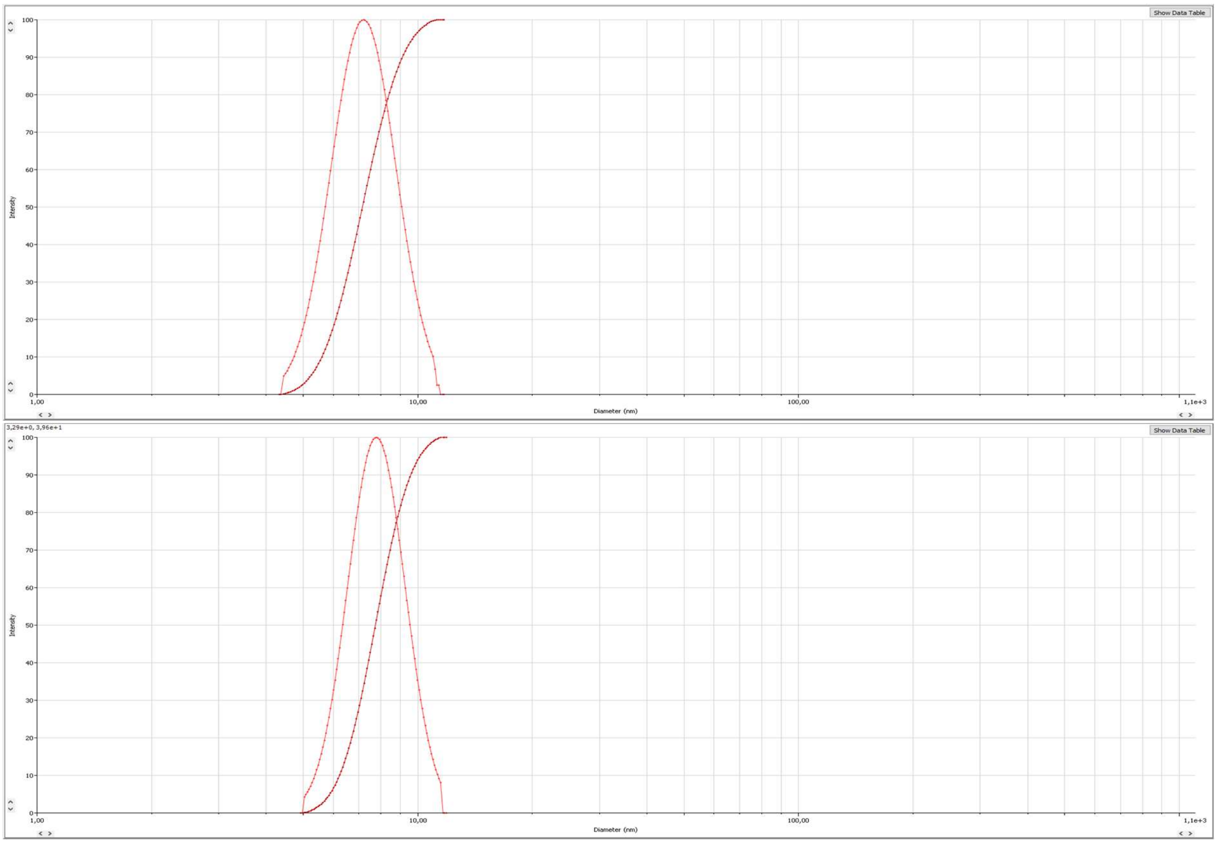

Figure S9. Intensity-weighted size distribution in the sample of dendron **DnP$_3$-1C$_{12}$** (**6a**) at day 1 (top) and day 75 (bottom) as determined by DLS

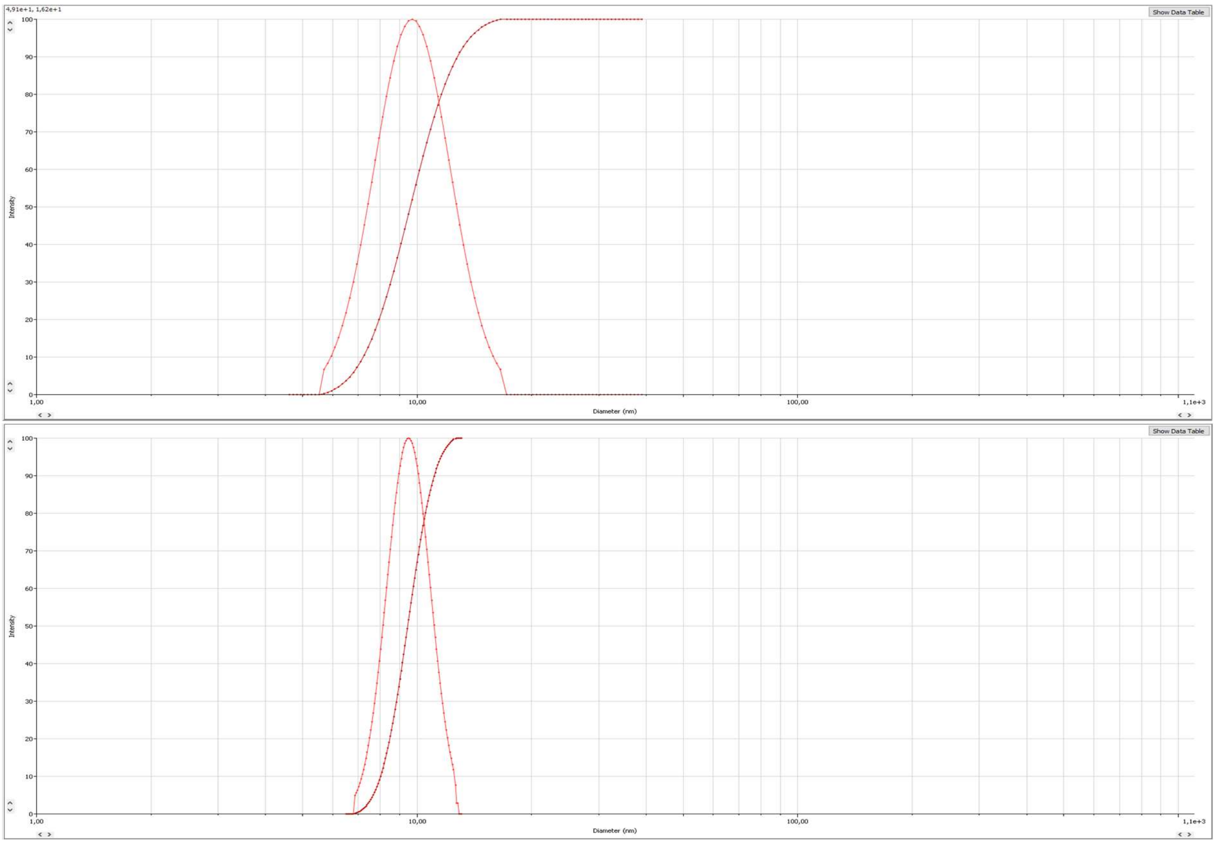

Figure S10. Intensity-weighted size distribution in the sample of dendron **DnP$_3$-1C$_{18}$** (**6b**) at day 1 (top) and day 75 (bottom) as determined by DLS

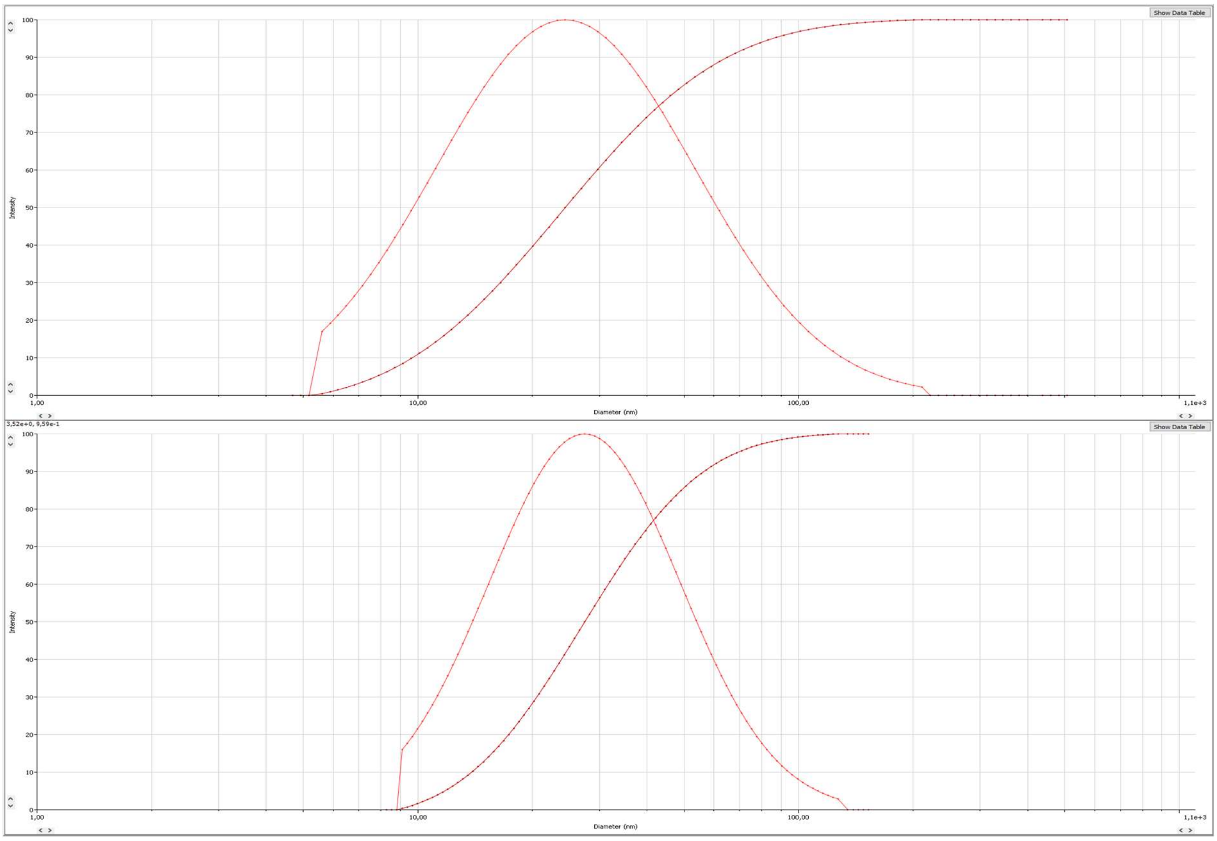

Figure S11. Intensity-weighted size distribution in the sample of dendron **DnP$_3$-2C$_{12}$** (**3a**) at day 1 (top) and day 75 (bottom) as determined by DLS

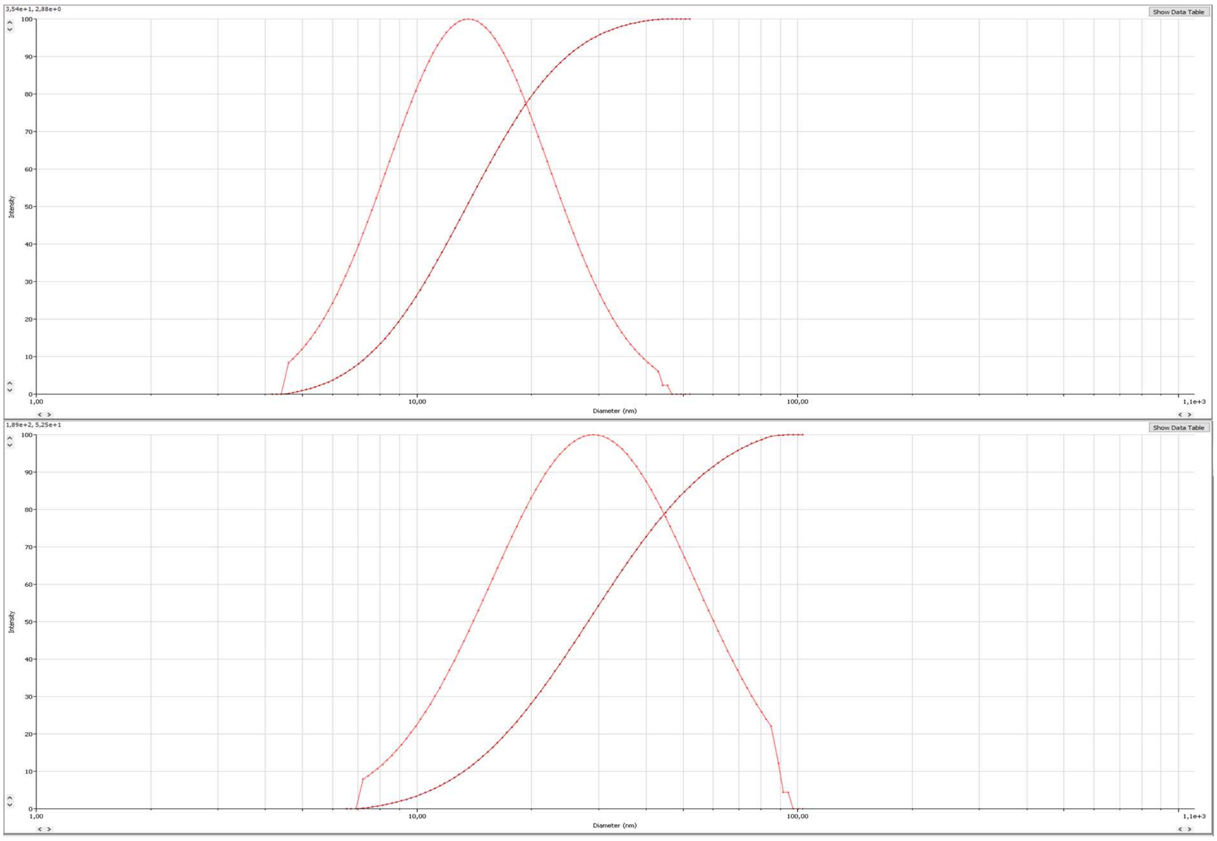

Figure S12. Intensity-weighted size distribution in the sample of dendron **DnP$_3$-2C$_{18}$** (**3b**) at day 1 (top) and day 75 (bottom) as determined by DLS

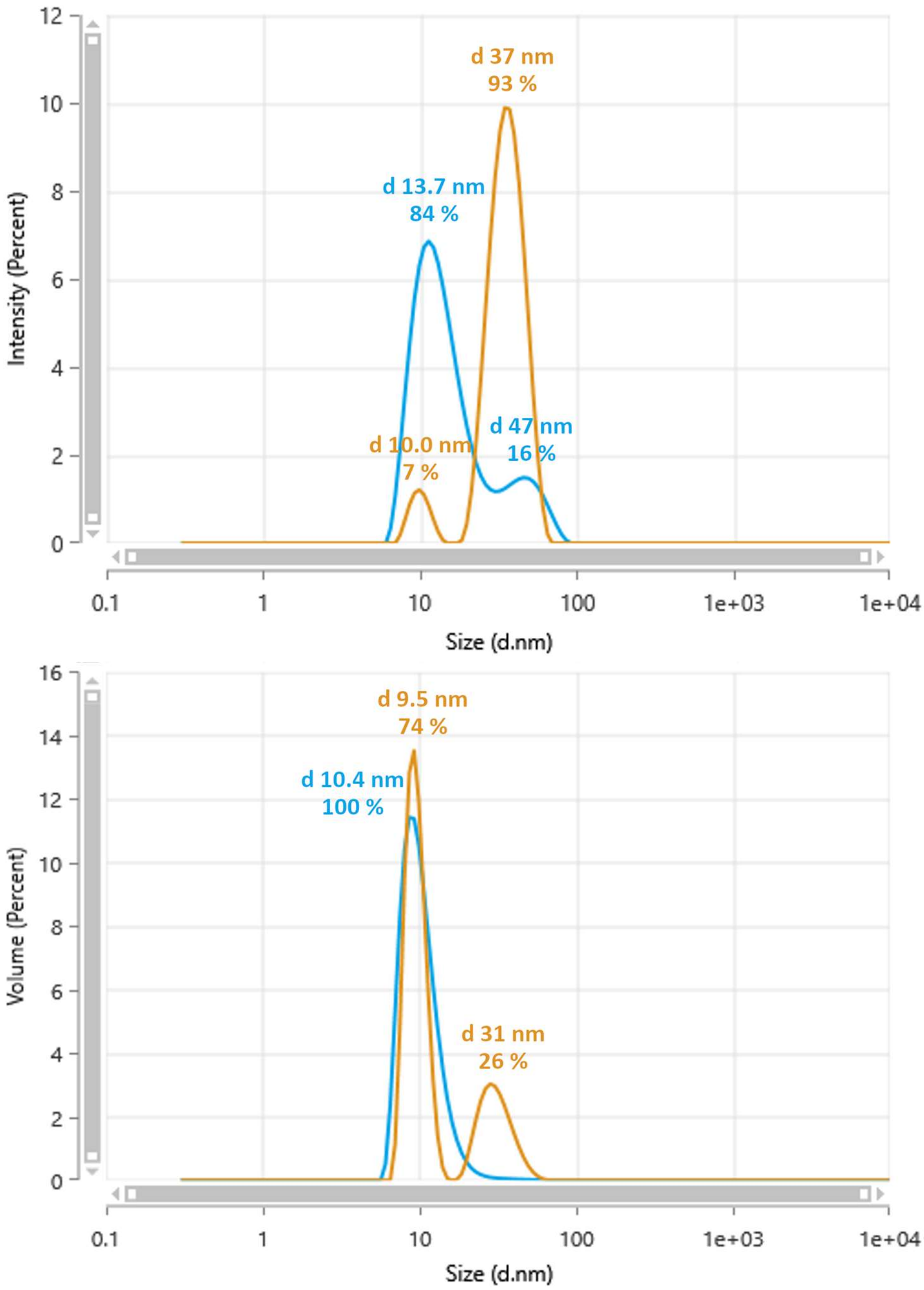

Figure S13. MADLS analysis of dendron **DnP$_3$-2C$_{12}$** (**3a**; 1.0 mM in 0.9 % saline, measured on Zetasizer Ultra Red); results obtained immediately after dissolution (blue) and after 75 days at 4 °C (orange); intensity-weighted size distribution (top) and volume-weighted size distribution (bottom)

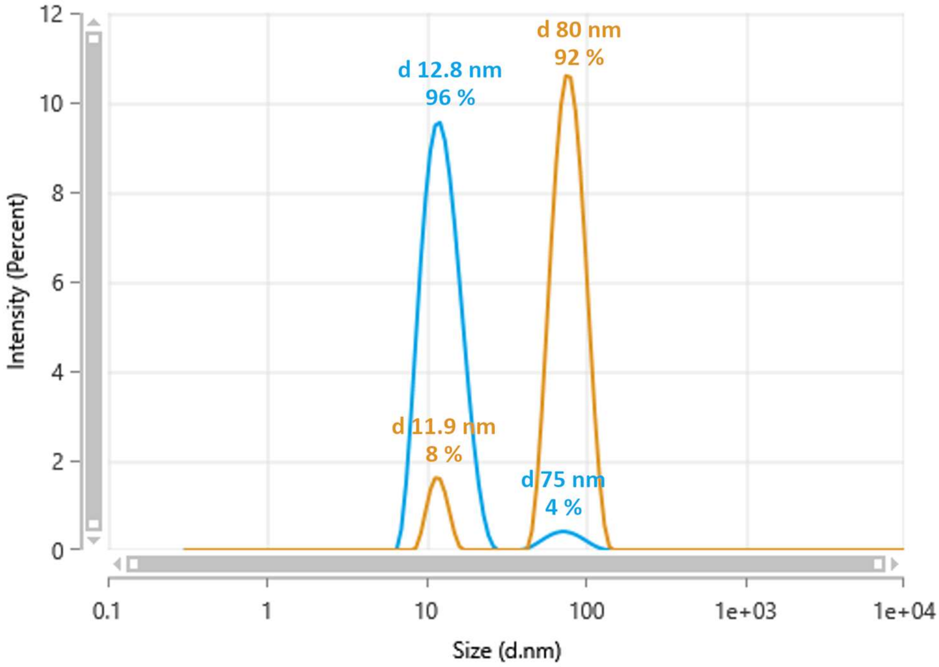

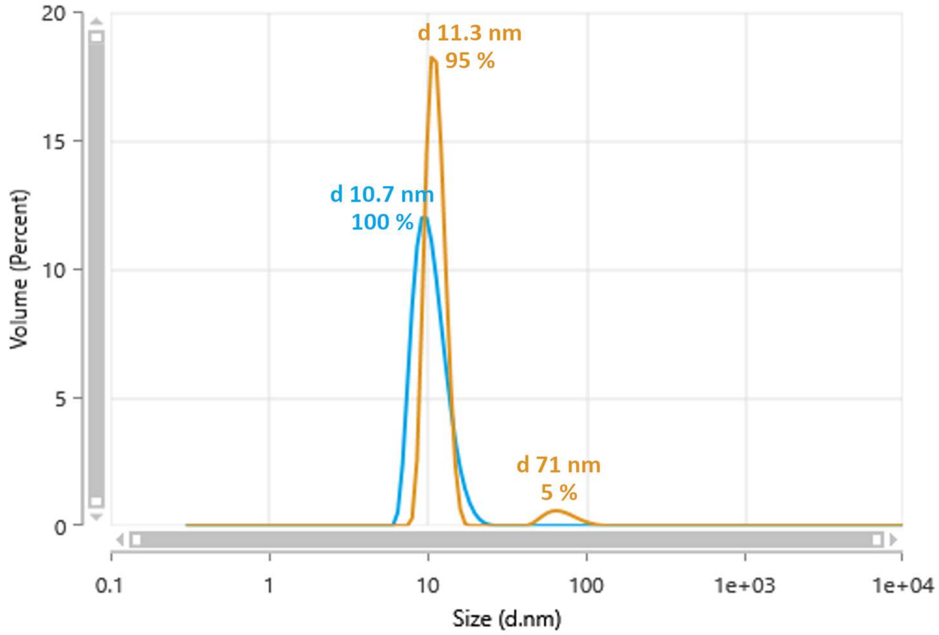

Figure S14. MADLS analysis of dendron **DnP$_3$-2C$_{18}$** (**3b**; 1.0 mM in 0.9 % saline, measured on Zetasizer Ultra Red); results obtained immediately after dissolution (blue) and after 75 days at 4 °C (orange); intensity-weighted size distribution (top) and volume-weighted size distribution (bottom)

5. Molecular modelling

METHODS

Computational details

3D computer models of dendrons were created using dendrimer builder, as implemented in the Materials Studio software package from BIOVIA (formerly Accelrys). The RESP technique[3] was used for calculation of dendrons atoms partial charges. For this charge parametrization the R.E.D. Server Development[4] was used. The necessary QM calculations (QM structure minimizations, molecular electrostatic potential (MEP) calculations) were done using GAMESS.[5,6] The default, HF/6-31G*, level of theory was used for all charge-related QM calculations and the MEP potential was fitted on Connolly molecular surface. GAFF force field (General Amber Force Field)[7] was used for parameterization of all dendrons. Missing force constants and energy barriers for "Si containing" force field terms were fitted by minimizing the differences between QM and force field based relative energies of 100 configurations of properly chosen molecular fragment (i.e. ff parameters that most accurately ensures the following requirement were used: E*i_force-field* = E*i_quantum* + K for all configurations *i* - where E*i_force-field* and E*i_quantum* are force field and QM based energy of molecular configuration *i* and K is constant). Equilibrium values of "Si containing" bonds and angles were obtained using QM optimization of the given molecular fragment. QM energies were calculated at MP2/HF/6-31G** level of theory using GAMESS and fitting was accomplished using *paramfit* routine from AMBER software.[8] Slightly adjusted van der Waals parameters for Si atoms from MM3 force field[9] were used in this study. All the calculated and used values of Si-containing force field parameters are available in the supporting information of our previous work.[1] Initial dendron molecular systems were prepared using *Packmol*.[10] In case of dendrons DnP_3-X, simulated systems are composed of 180 molecules (+ 10 DnP3-1C18/BDP dendrons in case of two-component systems) and in case of twice bigger DnP_6-X dendrons, 90 molecules (+ 5 DnP_3-$1C_{18}$/BDP dendrons in case of two-component systems) were used which ensured the same number of TPP terminal groups in all 6 simulated systems. Dendrons were initially randomly placed into a sphere with radius 110 Å, and minimal intermolecular distance requirement 7 Å. These initial molecular systems were solvated in explicit water (TIP3P model)[11] – see Figure **S15**. Diameter of the complete initial truncated octahedron simulation box was ca 281 Å. Volume of the simulation box was also similar in all 6 cases (ca 7 311 673 $Å^3$) and so also the density of terminal TPP groups. Proper number of Cl- and Na+ ions was added to preserve neutrality of the system and to ensure the physiological ionic strength of Cl-, Na+ ionic pairs (0.15 M). First, the systems were minimized (5000 steps with 2 kcal/(mol Å2) restraint + 50000 without restraint), heated (200 ps NVT) to 295 K (or also 310 K and 320 K in case of homogenous systems) and equilibrated using 400 ns long molecular dynamics simulations (NPT, T = 295 K (310 K, 320 K) , P = 0.1 MPa). The first 0.5 ns with restrained solute. Hydrogens were constrained with the SHAKE algorithm to allow 2 fs time step[12] and Langevin thermostat with collision frequency 2 ps^{-1} was used for all MD runs.[13] The pressure relaxation time for weak-coupling barostat was 2 ps. Particle mesh Ewald method (PME) was used to treat long range electrostatic interactions under periodic conditions with a direct space cutoff of 10 Å. The same cutoff was used for van der Waals interactions. The *pmemd.cuda* module[14] from Amber20 package[8] was used for all simulation steps. UCSF Chimera software was used for all visualizations.[15]

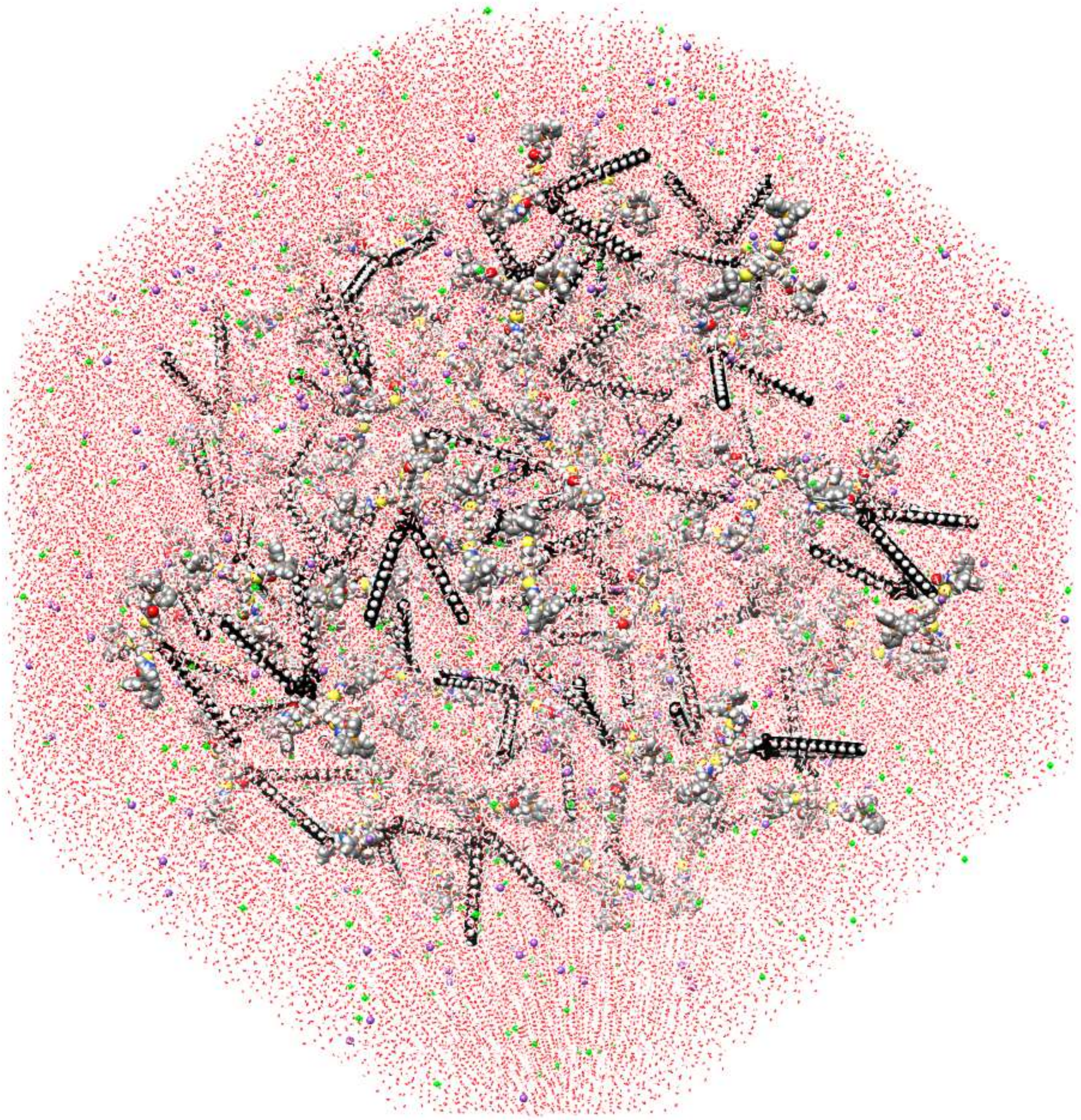

Figure S15. Example of the initial configuration of solvated system containing 180 DnP_3-$2C_{18}$ molecules; cross-section of the solvated dendron system is shown. Dendrons were randomly placed into a sphere with radius 110 Å, with a minimal intermolecular distance requirement 7 Å. Diameter of truncated octahedron simulation box is ca 281 Å. Carbons belonging to aliphatic chains are highlighted in black. Colors: C – grey/black; O – red; H – white; Si – beige; N – blue; S – yellow, Cl^- anions – green, Na^+ cations - purple.

Table S3. Overview of all characteristics of modeled homogeneous (one component) molecular systems. T - temperature, $N_{mol\text{-}1\text{-}C}$ - average number of molecules in one cluster, D_{max} - average largest dimension of molecular cluster, R_g - average radius of gyration of molecular cluster, N_{tot} - total number of molecular clusters (including isolated molecules). The standard deviations of the individual characteristics are given in parentheses.

**Dendron**	**T** [°C]	$N_{mol\text{-}1\text{-}C}$	D_{max} [Å]	R_g [Å]	N_{tot}
**DnP$_6$-1C$_{12}$**	22	2.65 (1.39)	46.47 (10.28)	13.44 (2.49)	34
	37	2.50 (1.11)	46.22 (6.28)	13.42 (1.70)	36
	47	2.57 (1.40)	46.37 (7.39)	13.50 (1.91)	35
**DnP$_6$-1C$_{18}$**	22	3.10 (1.86)	50.50 (12.51)	14.38 (3.32)	29
	37	3.21 (1.85)	50.43 (11.06)	14.32 (2.89)	28
	47	2.57 (1.38)	46.64 (8.02)	13.49 (1.86)	35
**DnP$_3$-1C$_{12}$**	22	7.83 (5.05)	55.25 (16.38)	15.88 (4.17)	23
	37	8.18 (3.78)	54.77 (10.42)	15.80 (2.69)	22
	47	6.92 (4.77)	52.06 (13.08)	15.06 (3.40)	26
**DnP$_3$-1C$_{18}$**	22	15.00 (11.76)	71.69 (25.24)	20.24 (7.34)	12
	37	10.59 (6.21)	62.94 (15.85)	17.98 (4.55)	17
	47	10.59 (5.09)	60.88 (11.20)	17.48 (2.94)	17
**DnP$_3$-2C$_{12}$**	22	11.25 (9.28)	64.93 (25.37)	18.49 (7.51)	16
	37	10.00 (7.58)	62.19 (19.96)	17.58 (5.58)	18
	47	9.47 (8.19)	58.22 (15.28)	16.66 (4.34)	19
**DnP$_3$-2C$_{18}$**	22	25.71 (35.34)	85.64 (66.08)	25.30 (21.02)	7
	37	22.50 (37.69)	78.85 (60.80)	22.66 (17.51)	8
	47	16.36 (20.93)	71.71 (33.85)	20.98 (10.62)	11
**DnP$_3$-1C$_{18}$/BDP**	22	180.00 (0.00)	195.53 (0.00)	61.17 (0.00)	1

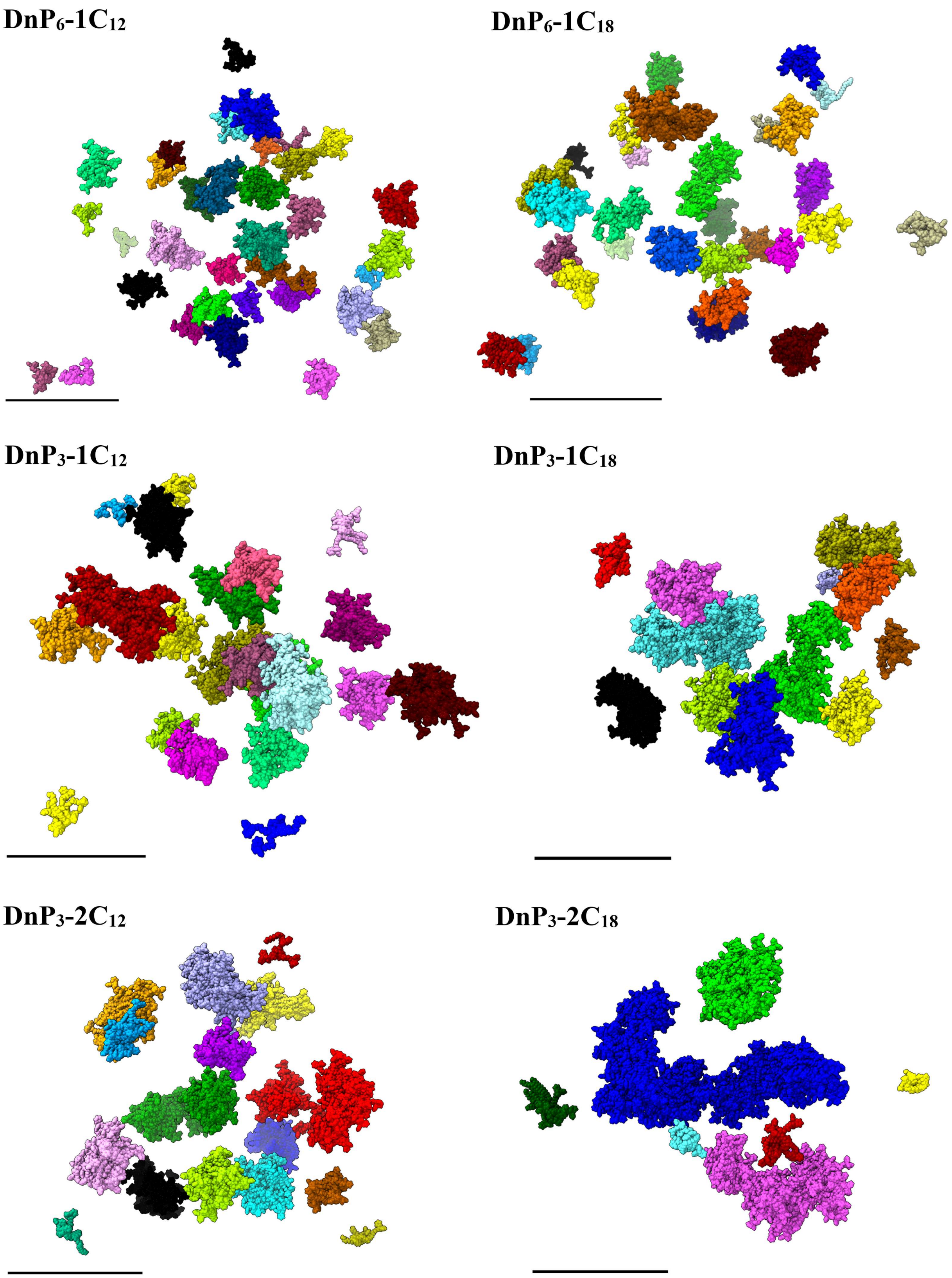

Figure S16. Visualization of the final dendron systems simulated at temperature 22 °C. Scale bar corresponds to a length of 100 Å.

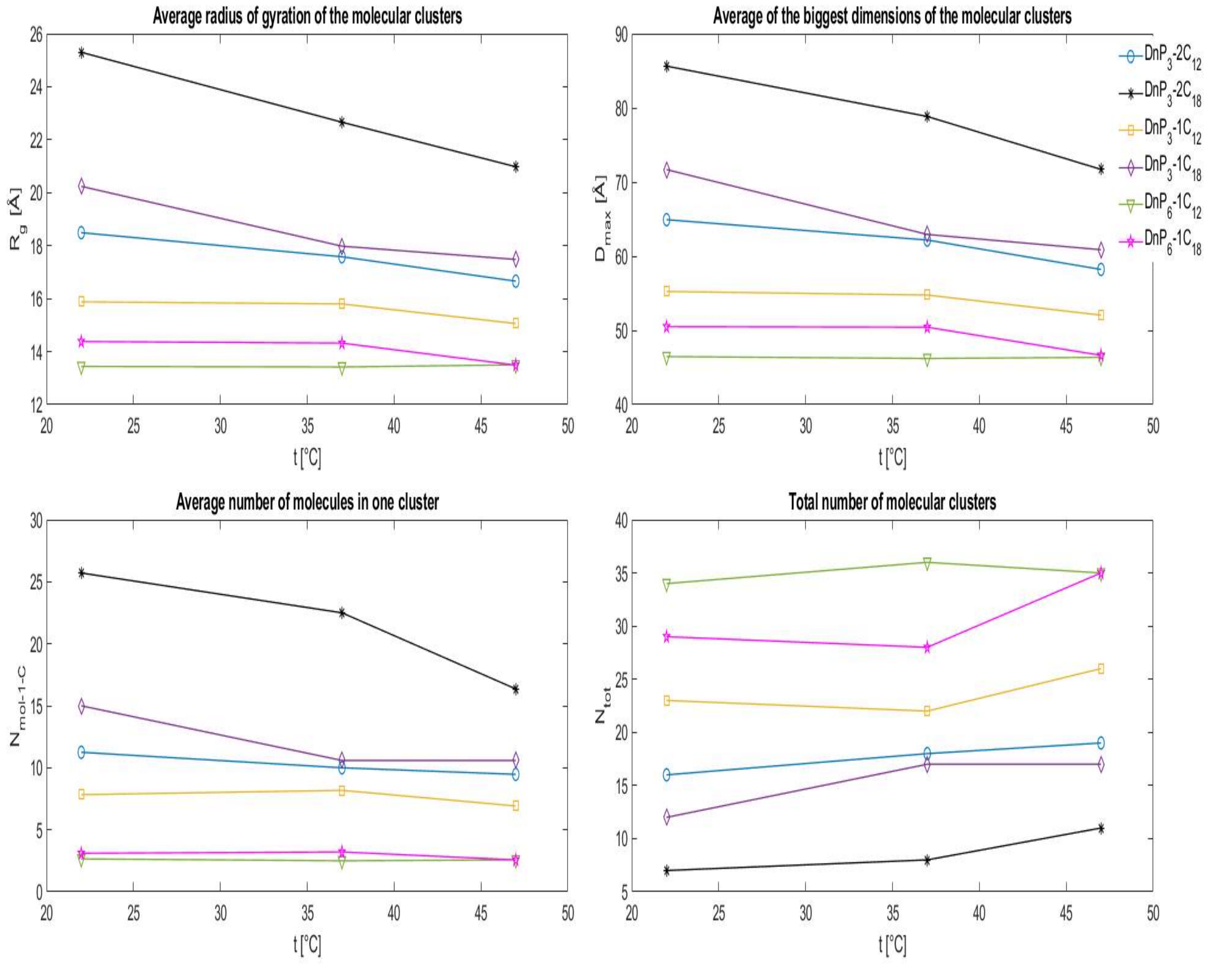

Figure S17. Structural characteristics of modeled one-component dendron complexes for three different temperatures 22 °C, 37 °C, 47 °C.

Table S4. Overview of all characteristics of modeled composite systems (i.e. containing ca 5 mol % of DnP$_3$-1C$_{18}$/BDP dendrons). $N_{mol-1-C}$ - average number of molecules in one cluster, D_{max} - average largest dimension of molecular cluster, R_g - average radius of gyration of molecular cluster, N_{tot} - total number of molecular clusters (including isolated molecules). All systems were simulated at T = 22 °C. The standard deviations of the individual characteristics are given in parentheses.

Dendron system	$N_{mol-1-C}$	D_{max} [Å]	R_g [Å]	N_{tot}
**DnP$_6$-1C$_{12}$@DnP$_3$-1C$_{18}$/BDP**	2.57 (1.19)	46.68 (7.48)	13.23 (1.79)	37
**DnP$_6$-1C$_{18}$@DnP$_3$-1C$_{18}$/BDP**	3.80 (2.81)	53.75 (16.88)	15.39 (4.56)	25
**DnP$_3$-1C$_{12}$@DnP$_3$-1C$_{18}$/BDP**	7.60 (6.96)	55.32 (22.44)	15.54 (6.04)	25
**DnP$_3$-1C$_{18}$@DnP$_3$-1C$_{18}$/BDP**	15.83 (20.71)	78.26 (53.00)	21.60 (15.12)	12
**DnP$_3$-2C$_{12}$@DnP$_3$-1C$_{18}$/BDP**	17.27 (30.42)	69.91 (53.14)	20.56 (15.99)	11
**DnP$_3$-2C$_{18}$@DnP$_3$-1C$_{18}$/BDP**	190.00 (0.00)	245.83 (0.00)	73.73 (0.00)	1

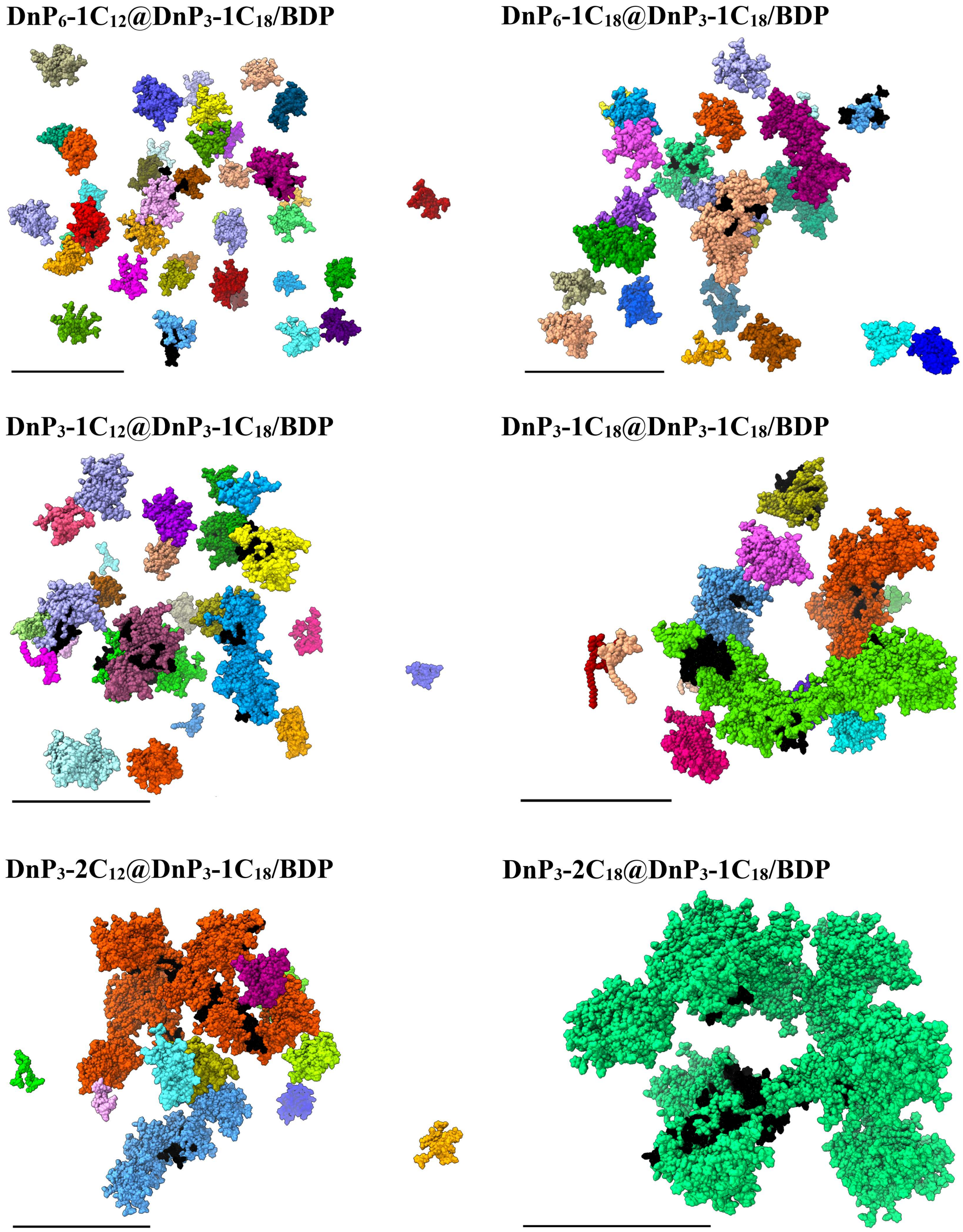

Figure S18. Visualization of the final composite dendron systems doped with 5 mol % of fluorescently labeled dendron DnP$_3$-1C$_{18}$/BDP simulated at 22 °C. DnP$_3$-1C$_{18}$/BDP molecules are depicted in black in each figure to highlight their distribution. Scale bar corresponds to a length of 100 Å.

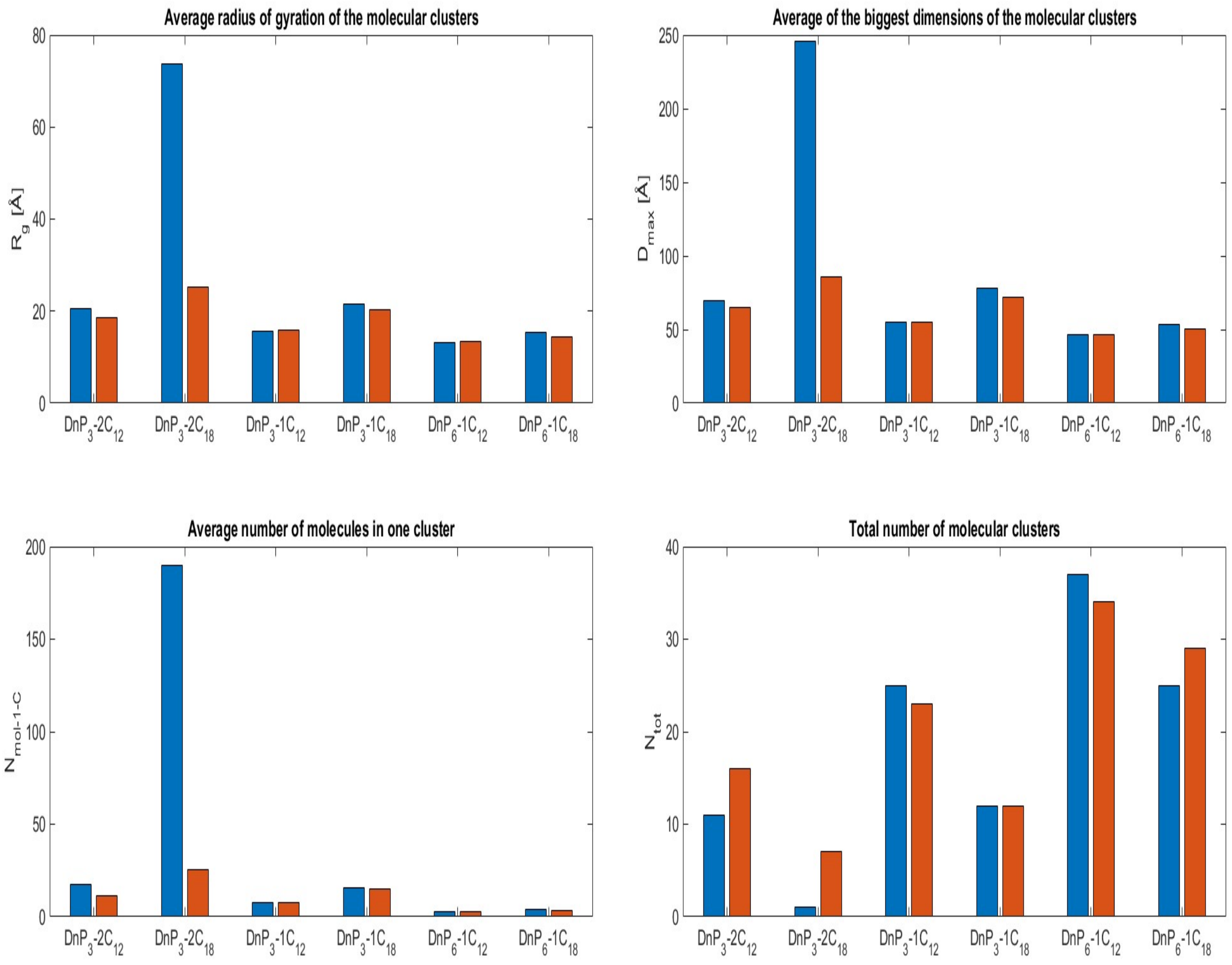

Figure S19. Comparison of structural characteristics of simulated composite dendron systems doped with 5 mol % of fluorescently labeled dendron $DnP_3\text{-}1C_{18}$/BDP (blue) and the same characteristics of homogeneous molecular systems (red), at 22 °C.

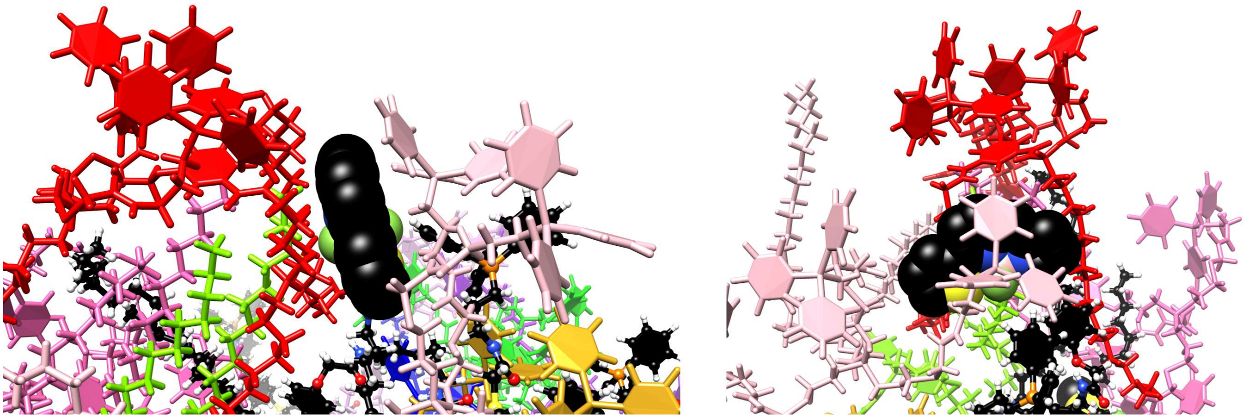

Figure S20. Interaction of the BODIPY end (sphere representation) of $DnP_3\text{-}1C_{18}$/BDP dendron with aliphatic carbon chains (red, green) and peripheral phenyl groups (pink) of neighboring $DnP_3\text{-}2C_{18}$ dendrons. Two different views are shown. Color coding of $DnP_3\text{-}1C_{18}$/BDP atoms: O – red, N – blue, S – yellow, F – green, H – white (not shown on BODIPY), C – black.

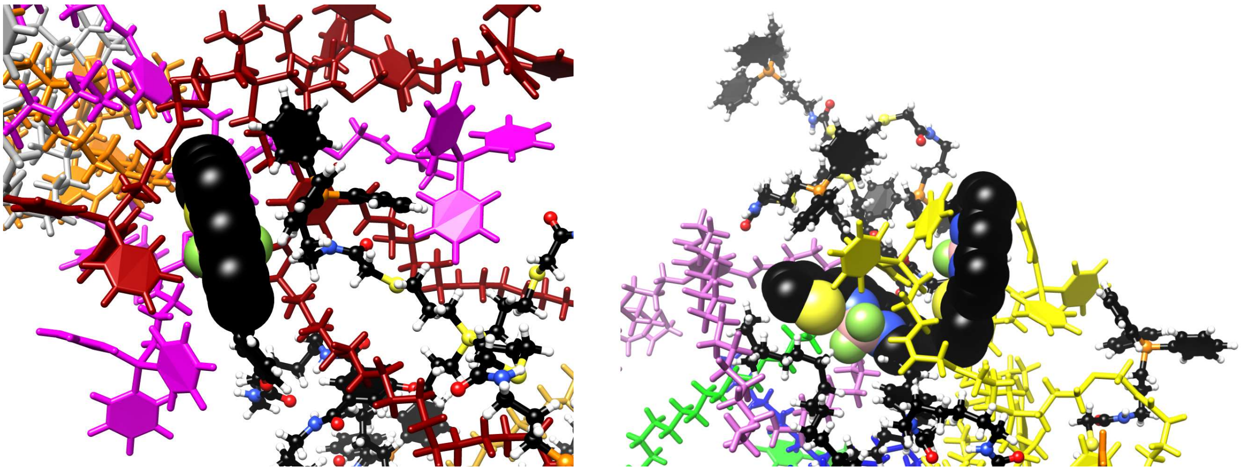

Figure S21. LEFT: Interaction of the BODIPY end (sphere representation) of DnP_3-$1C_{18}$/BDP dendron with peripheral phenyl groups of the same dendron (black) and neighboring DnP_3-$2C_{18}$ dendrons (magenta, red). RIGHT: Interaction of the BODIPY ends (sphere representation) of two DnP_3-$1C_{18}$/BDP dendrons with peripheral phenyl groups (yellow) and aliphatic carbon chains of neighboring DnP_3-$2C_{18}$ dendrons (pink, green) and one of the DnP_3-$1C_{18}$/BDP dendrons (black). Color coding of DnP_3-$1C_{18}$/BDP atoms: O – red, N – blue, S – yellow, F – green, H – white (not shown on BODIPY), C – black.

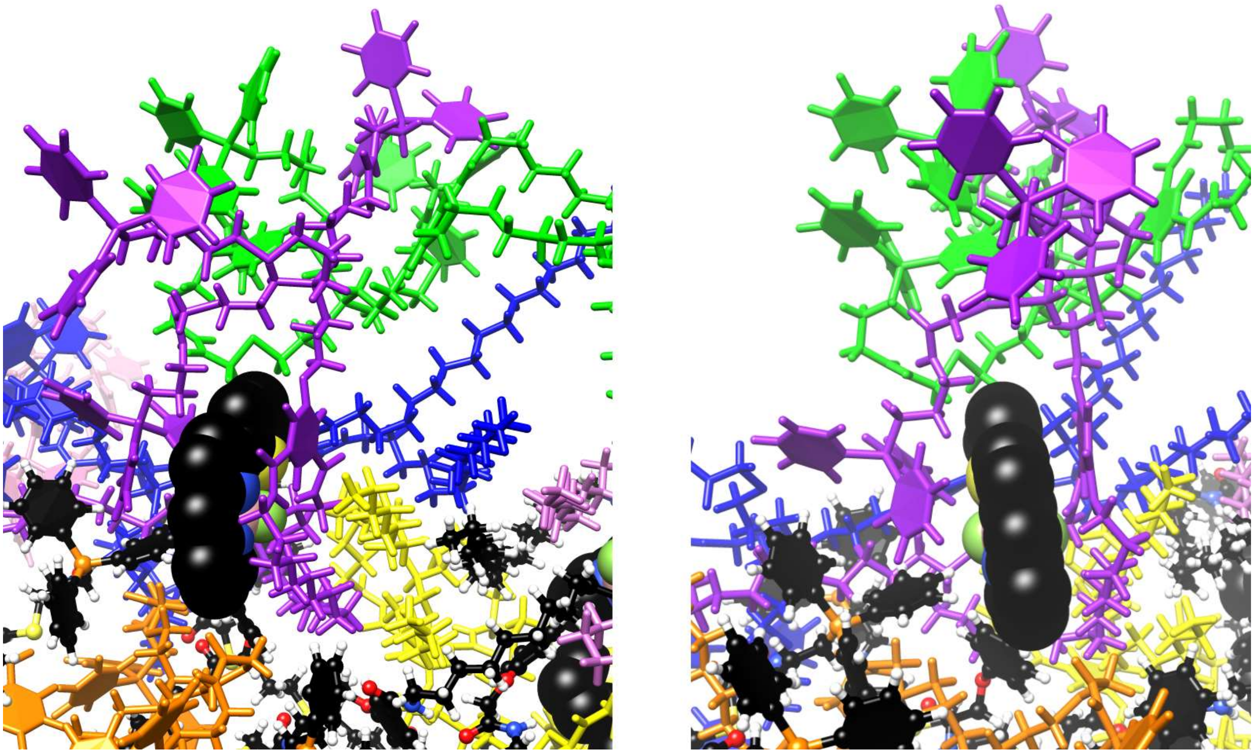

Figure S22. Interaction of the BODIPY end (sphere representation) of DnP_3-$1C_{18}$/BDP dendron with peripheral phenyl groups and central aromatic ring of the neighboring DnP_3-$2C_{18}$ dendron (purple). Two different views are shown. Color coding of DnP_3-$1C_{18}$/BDP atoms: O – red, N – blue, S – yellow, F – green, H – white (not shown on BODIPY), C – black.

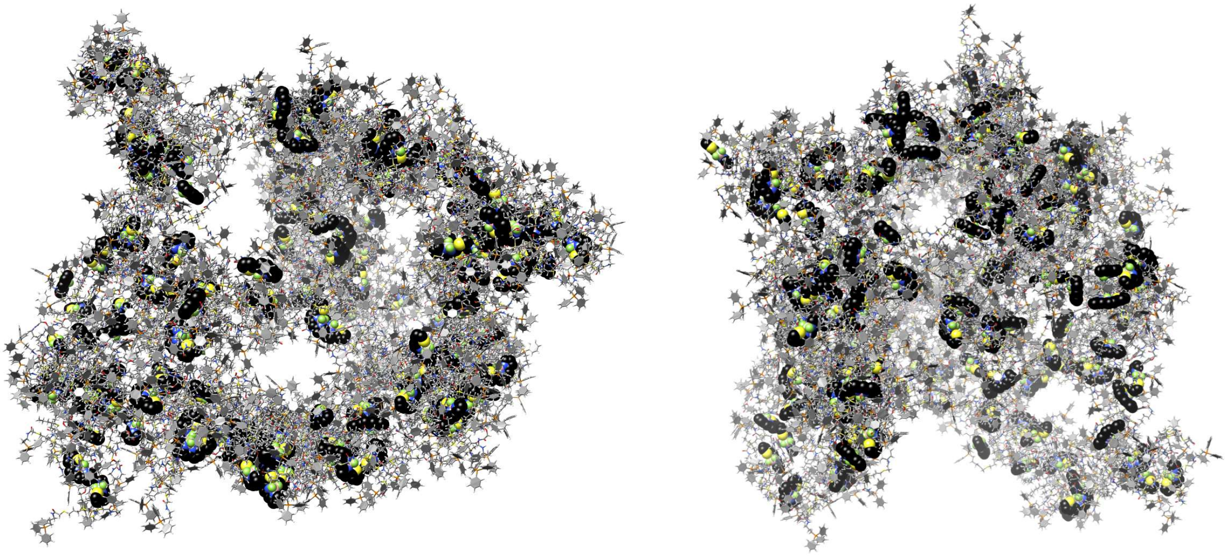

Figure S23. Simulated system of 180 dendrons DnP$_3$-1C$_{18}$/BDP at 22 °C. BODIPY ends are in sphere representation with black C atoms and omitted H atoms. Two different views are shown. Color coding of non-BODIPY atoms: O – red, N – blue, S – yellow, F – green, H – white, C – grey.

6. Dendriplex size measurements

Measured at concentration 1.5 – 73 µM of dendron and 500 nM of anti-miRNA in physiological solution (0.9 % NaCl in deionized water). Figures for results categorized as “promising” and “suitable”, i.e. with mean size under 250 nm, are given.

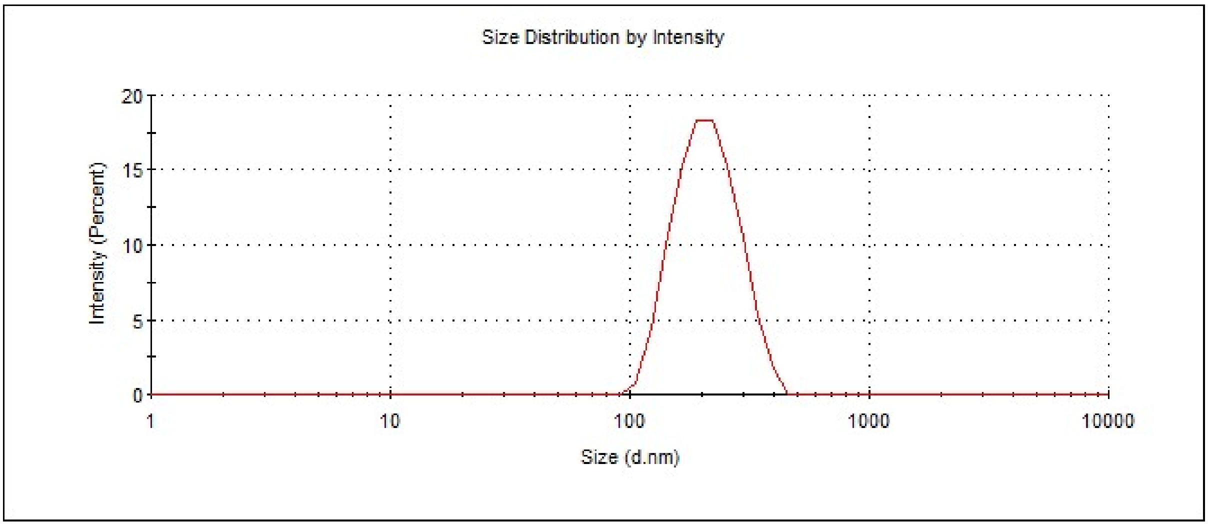

Figure S24. Dendron **DnP$_6$-1C$_{12}$** (**9a**) dendriplex size distribution at CR 1.2

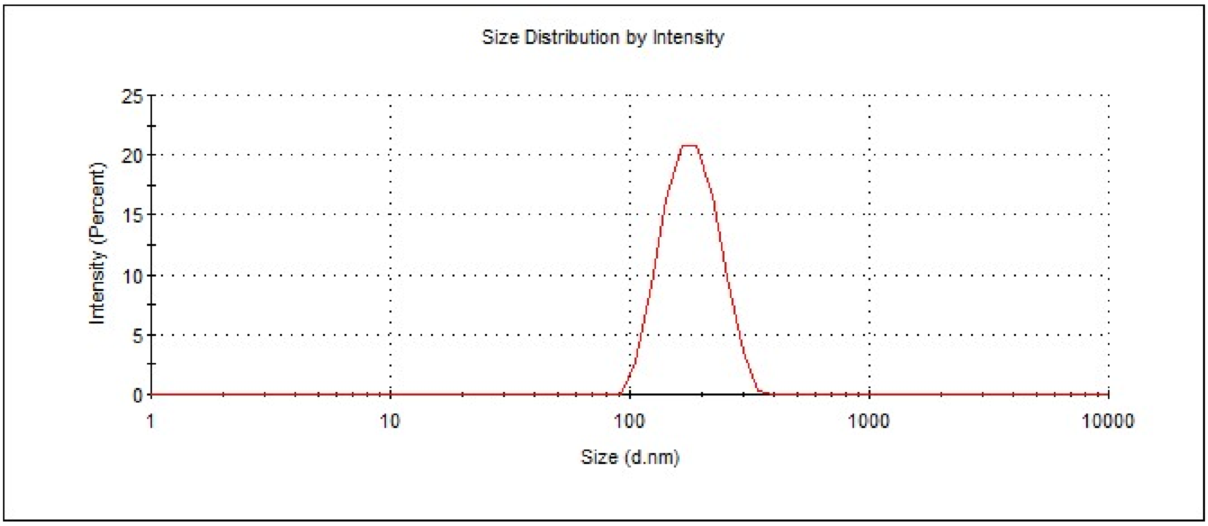

Figure S25. Dendron **DnP$_6$-1C$_{18}$** (**9b**) dendriplex size distribution at CR 1.2

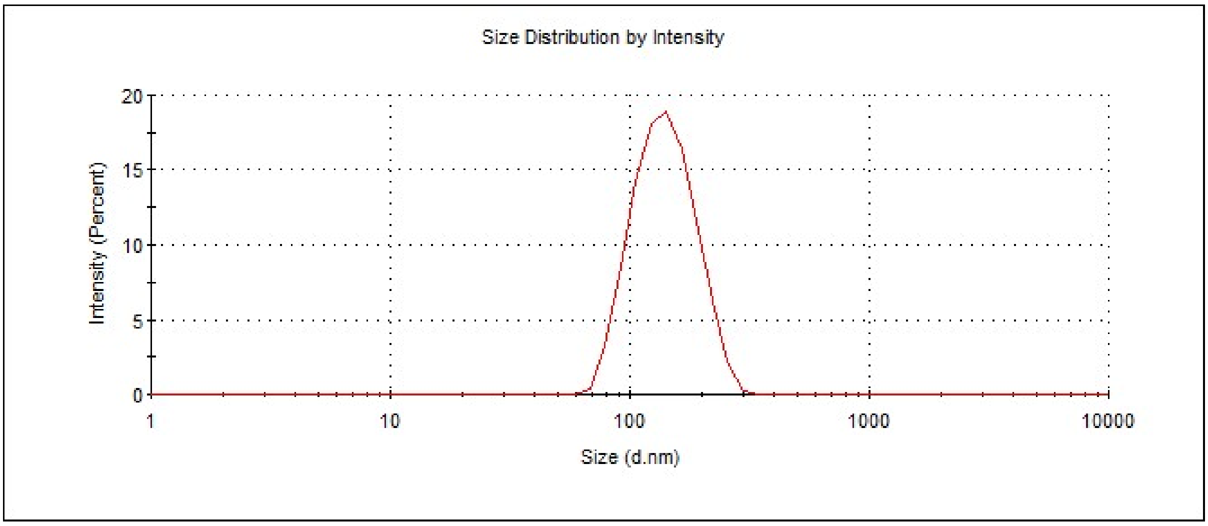

Figure S26. Dendron **DnP$_3$-1C$_{12}$** (**6a**) dendriplex size distribution at CR 1.2

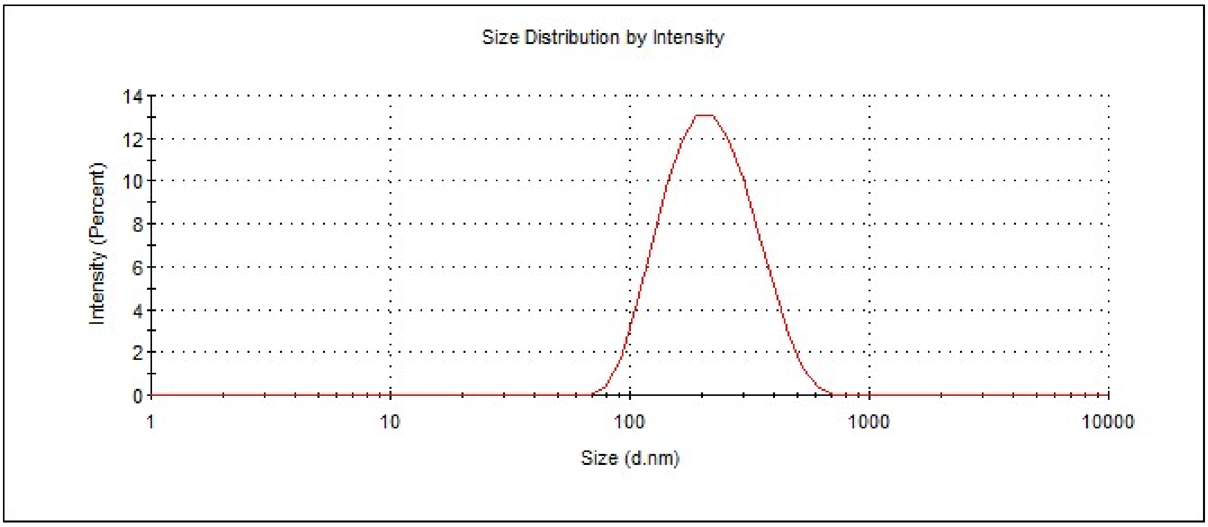

Figure S27. Dendron **DnP$_3$-1C$_{18}$** (**6b**) dendriplex size distribution at CR 1.2

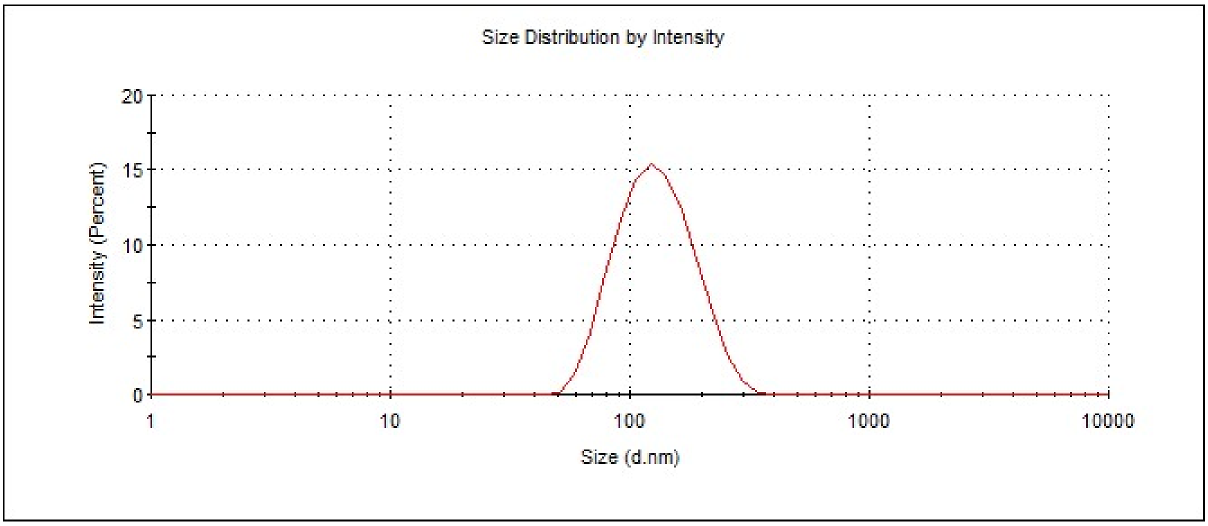

Figure S28. Dendron **DnP$_3$-2C$_{12}$** (**3a**) dendriplex size distribution at CR 1.2

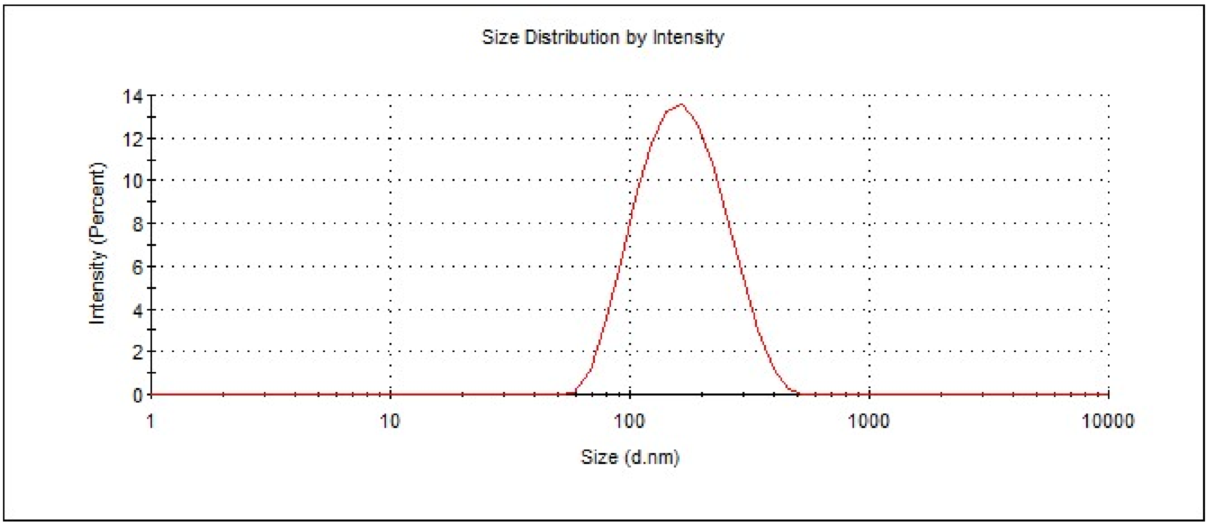

Figure S29. Dendron **DnP$_3$-2C$_{12}$** (**3a**) dendriplex size distribution at CR 10

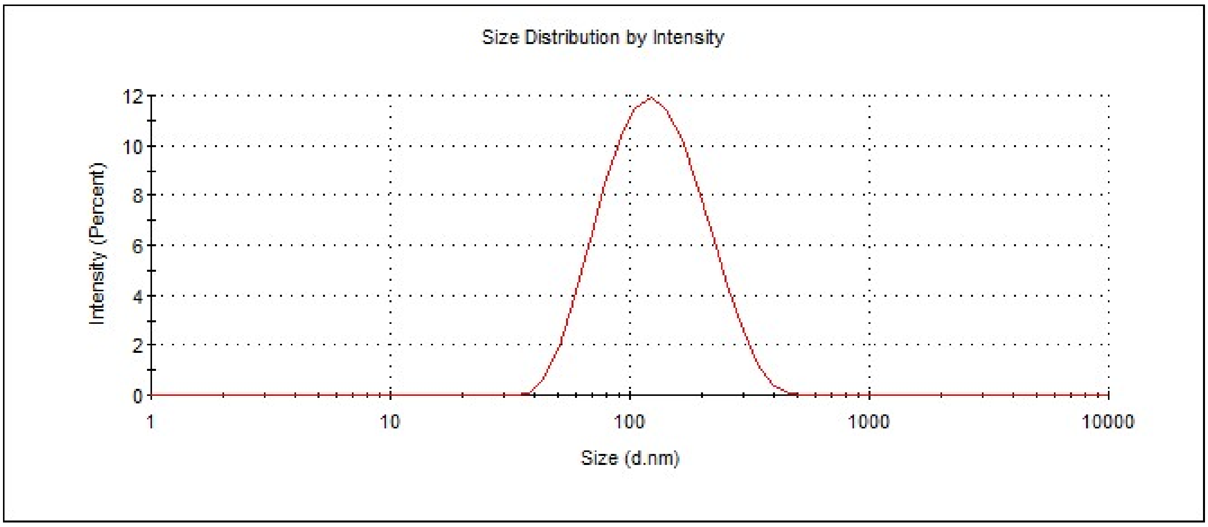

Figure S30. Dendron **DnP$_3$-2C$_{12}$** (**3a**) dendriplex size distribution at CR 20

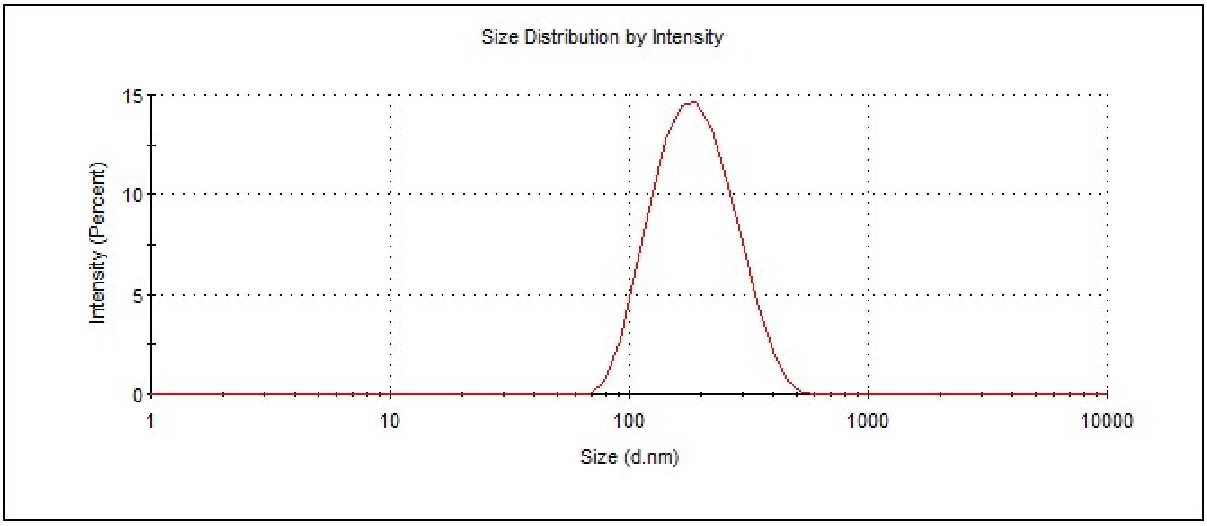

Figure S31. Dendron **DnP$_3$-2C$_{18}$** (**3b**) dendriplex size distribution at CR 1.2

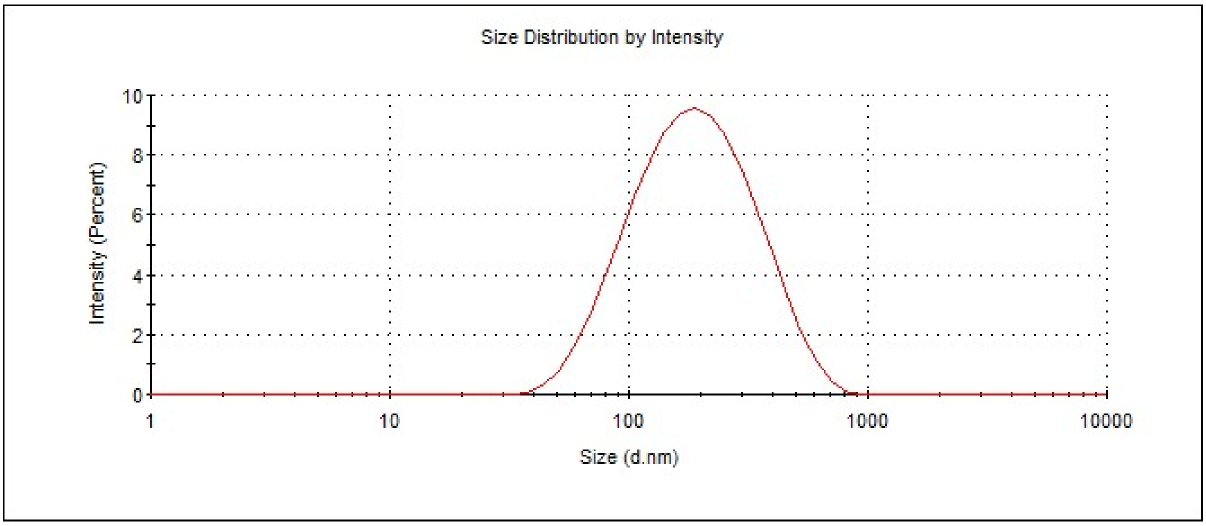

Figure S32. Dendron **DnP$_3$-2C$_{18}$** (**3b**) dendriplex size distribution at CR 10

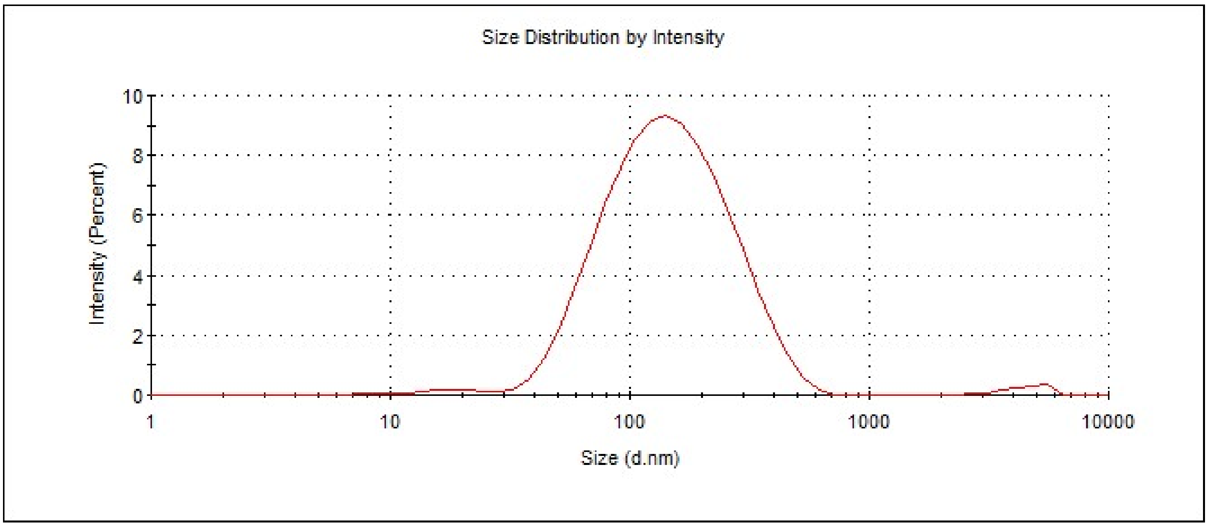

Figure S33. Dendron **DnP$_3$-2C$_{18}$** (**3b**) dendriplex size distribution at CR 20

7. Antibacterial activity of clinically used antibiotics

Table S5. Minimum inhibitory concentration (µmol/L) of selected antibiotics against tested bacteria[a]

G+	Penicillin	Ampicillin	Cefoxitin	Erythromycin	Clindamycin	Linezolid	Chloramphenicol	Tetracycline	Ciprofloxacin	Gentamicin	Vancomycin	Teicoplanin	Nitrofurantoin
***S. aureus* CCM 4516**	nd	< 0.5	nd	0.5	< 0.25	**1**	12	< 0.5	nd	1	0.75	0.12	64
***S. aureus* MRSA – clinical isolate C1926**	> 16	nd	> 37	> 10	> 8	1.5	32	0.5	> 24	0.5	1.5	nd	128
***S. epidermidis* – clinical isolate C1936**	16	nd	2	1	0.25	1	12	4.5	0.75	< 0.25	1.5	nd	50
***E. faecium* VRE – clinical isolate S2484**	> 8	> 45	nd	> 10	> 32	1	12	72	nd	8	> 12	> 8	128

G-	Ampicillin	Cefazolin	Cefuroxime	Aztreonam	Gentamicin	Meropenem	Amikacin	Colistin	Ciprofloxacin	Chloramphenicol	Tetracycline	Tigecycline
***E. coli* K12, CCM 7929**	12	4.5	15	< 0.25	1	nd	< 0.75	0.25	< 2	12	2	nd
***E. coli* – clinical isolate A1235**	> 360	35	20	< 0.25	> 64	nd	5	0.5	< 2	12	> 72	nd
***K. pneumoniae* ESBL+ – clinical isolate C1914**	> 360	> 35	> 150	> 36	> 64	nd	5	0.5	> 24	40	12	nd
***P. aeruginosa* MR – clinical isolate A1245**	360	nd	> 40	36	> 64	> 42	20	> 16	> 24	nd	nd	12

[a] The values highlighted in green indicate sensitivity, the yellow values are intermediate, and the red values indicate resistance according to EUCAST breakpoints. The values without highlighting do not have a breakpoint. Nd means not determined. These data have been produced in part under ECDC service contracts and made available at no cost by EUCAST and can be accessed freely on the EUCAST website www.eucast.org. EUCAST recommendations are frequently updated and the latest versions are available at www.eucast.org.

8. References

(1) Edr, A.; Wrobel, D.; Krupková, A.; Červenková Šťastná, L.; Cuřínová, P.; Novák, A.; Malý, J.; Kalasová, J.; Malý, J.; Malý, M.; et al. Adaptive Synthesis of Functional Amphiphilic Dendrons as a Novel Approach to Artificial Supramolecular Objects. *Int. J. Mol. Sci.* **2022**, *23* (4), 2114.

(2) Madak, J. T.; Cuthbertson, C. R.; Chen, W.; Showalter, H. D.; Neamati, N. Design, Synthesis, and Characterization of Brequinar Conjugates as Probes to Study DHODH Inhibition. *Chem. - A Eur. J.* **2017**, *23* (56), 13875–13878.

(3) Bayly, C. I.; Cieplak, P.; Cornell, W.; Kollman, P. A. A Well-Behaved Electrostatic Potential Based Method Using Charge Restraints for Deriving Atomic Charges: The RESP Model. *J. Phys. Chem.* **1993**, *97* (40), 10269–10280.

(4) Vanquelef, E.; Simon, S.; Marquant, G.; Garcia, E.; Klimerak, G.; Delepine, J. C.; Cieplak, P.; Dupradeau, F. Y. R.E.D. Server: A Web Service for Deriving RESP and ESP Charges and Building Force Field Libraries for New Molecules and Molecular Fragments. *Nucleic Acids Res.* **2011**, *39*, W511–W517.

(5) Schmidt, M. W.; Baldridge, K. K.; Boatz, J. A.; Elbert, S. T.; Gordon, M. S.; Jensen, J. H.; Koseki, S.; Matsunaga, N.; Nguyen, K. A.; Su, S.; et al. General Atomic and Molecular Electronic Structure System. *J. Comput. Chem.* **1993**, *14* (11), 1347–1363.

(6) Gordon, M. S.; Schmidt, M. W. Advances in Electronic Structure Theory: GAMESS a Decade Later. In *Theory and Applications of Computational Chemistry*; Elsevier, 2005; pp 1167–1189.

(7) Wang, J.; Wolf, R. M.; Caldwell, J. W.; Kollman, P. A.; Case, D. A. Development and Testing of a General Amber Force Field. *J. Comput. Chem.* **2004**, *25* (9), 1157–1174.

(8) D.A. Case, K. Belfon, I.Y. Ben-Shalom, S.R. Brozell, D.S. Cerutti, T.E. Cheatham, III, V.W.D. Cruzeiro, T.A. Darden, R.E. Duke, G. Giambasu, M.K. Gilson, H. Gohlke, A.W. Goetz, R Harris, S. Izadi, S.A. Izmailov, K. Kasavajhala, A. Kovalenko, R. Krasny, T. Kurtzman, T.S. Lee, S. LeGrand, P. Li, C. Lin, J. Liu, T. Luchko, R. Luo, V. Man, K.M. Merz, Y. Miao, O. Mikhailovskii, G. Monard, H. Nguyen, A. Onufriev, F.Pan, S. Pantano, R. Qi, D.R. Roe, A. Roitberg, C. Sagui, S. Schott-Verdugo, J. Shen, C.L. Simmerling, N.R.Skrynnikov, J. Smith, J. Swails, R.C. Walker, J. Wang, L. Wilson, R.M. Wolf, X. Wu, Y. Xiong, Y. Xue,D.M. York and P.A. Kollman (2020), AMBER 2020, University of California, San Francisco.

(9) Lii, J. H.; Allinger, N. L. The MM3 Force Field for Amides, Polypeptides and Proteins. *J. Comput. Chem.* **1991**, *12* (2), 186–199.

(10) Martínez, L.; Andrade, R.; Birgin, E. G.; Martínez, J. M. PACKMOL: A Package for Building Initial Configurations for Molecular Dynamics Simulations. *J. Comput. Chem.* **2009**, *30* (13), 2157–2164.

(11) Jorgensen, W. L.; Chandrasekhar, J.; Madura, J. D.; Impey, R. W.; Klein, M. L. Comparison of Simple Potential Functions for Simulating Liquid Water. *J. Chem. Phys.* **1983**, *79* (2), 926–935.

(12) Ryckaert, J.-P.; Ciccotti, G.; Berendsen, H. J. . Numerical Integration of the Cartesian Equations of Motion of a System with Constraints: Molecular Dynamics of n-Alkanes. *J. Comput. Phys.* **1977**, *23* (3), 327–341.

(13) Wu, X.; Brooks, B. R.; Vanden-Eijnden, E. Self-Guided Langevin Dynamics via Generalized Langevin Equation. *J. Comput. Chem.* **2016**, *37* (6), 595–601.

(14) Götz, A. W.; Williamson, M. J.; Xu, D.; Poole, D.; Le Grand, S.; Walker, R. C. Routine Microsecond Molecular Dynamics Simulations with AMBER on GPUs. 1. Generalized Born. *J. Chem. Theory Comput.* **2012**, *8* (5), 1542–1555.

(15) Pettersen, E. F.; Goddard, T. D.; Huang, C. C.; Couch, G. S.; Greenblatt, D. M.; Meng, E. C.; Ferrin, T. E. UCSF Chimera - A Visualization System for Exploratory Research and Analysis. *J. Comput. Chem.* **2004**, *25* (13), 1605–1612.

9. NMR spectra of amphiphilic dendrons

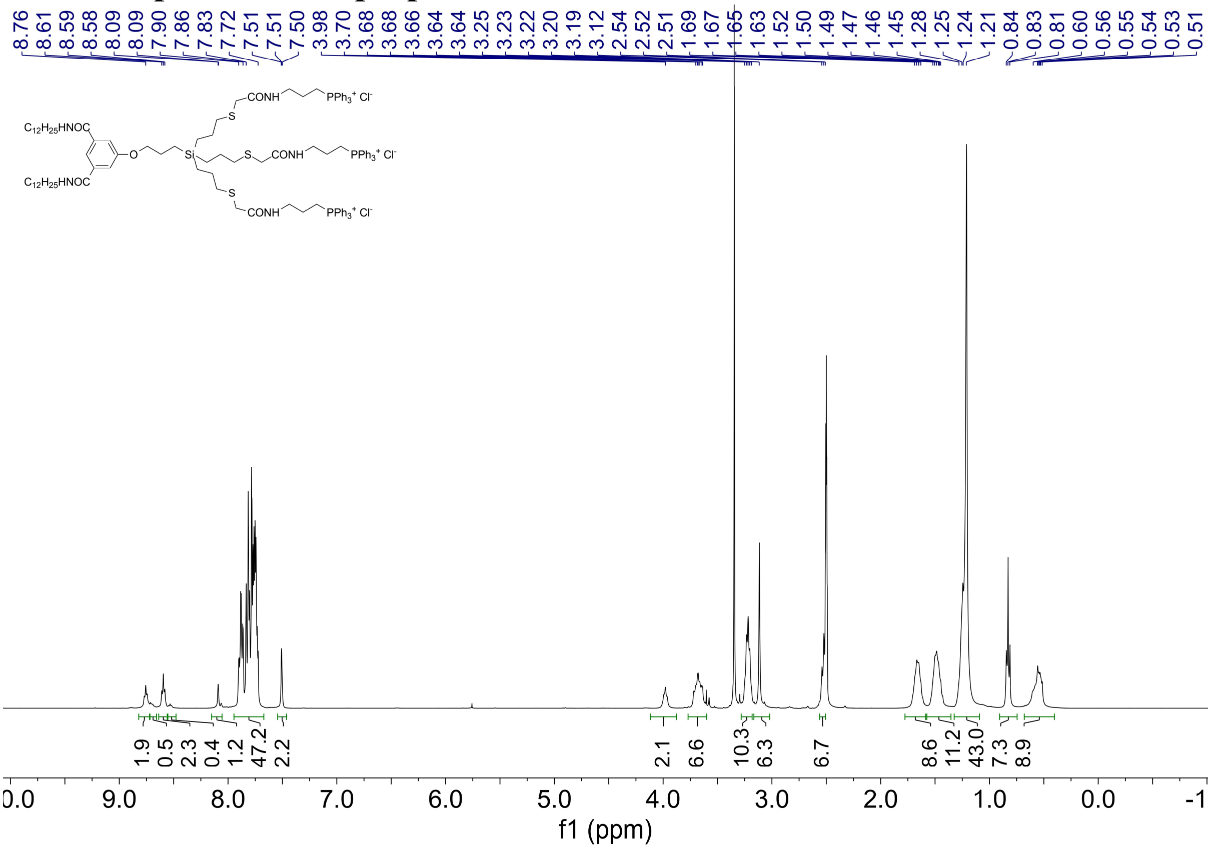

Figure S34. ^{1}H NMR (400 MHz, DMSO-d_6) of **DnP$_3$-2C$_{12}$** (**3a**).

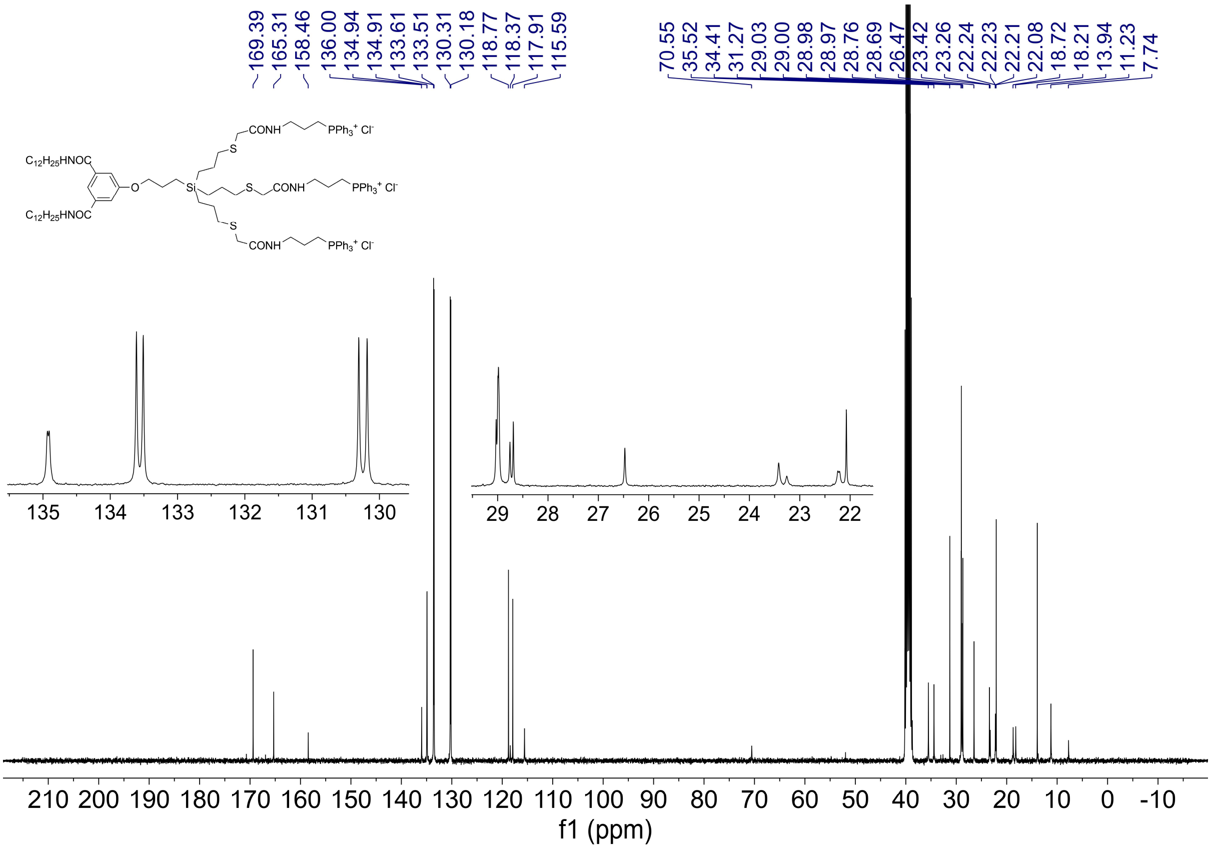

Figure S35. $^{13}C\{^{1}H\}$ NMR (400 MHz, DMSO-d_6) of **DnP$_3$-2C$_{12}$** (**3a**).

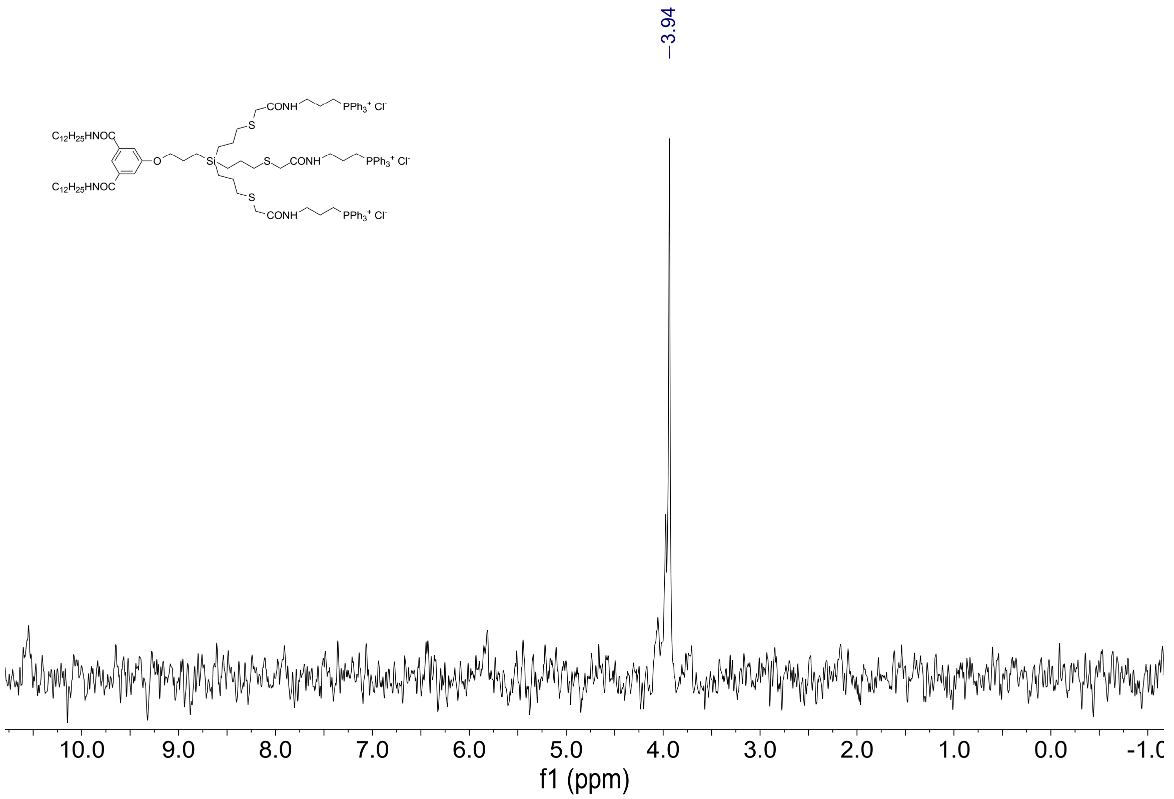

Figure S36. $^{29}Si\{^{1}H\}$ NMR (400 MHz, DMSO-d_6) of **DnP$_3$-2C$_{12}$** (**3a**).

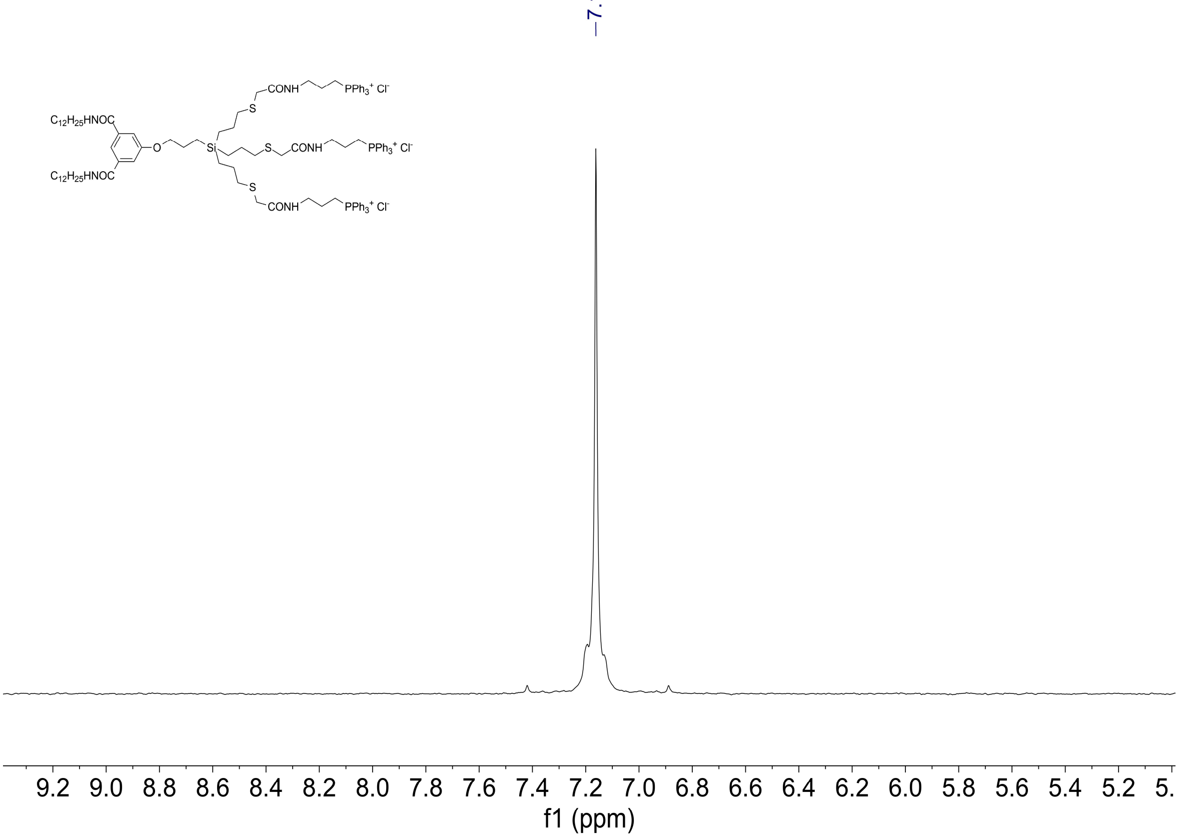

Figure S37. $^{31}P\{^{1}H\}$ NMR (400 MHz, DMSO-d_6) of **DnP$_3$-2C$_{12}$** (**3a**).

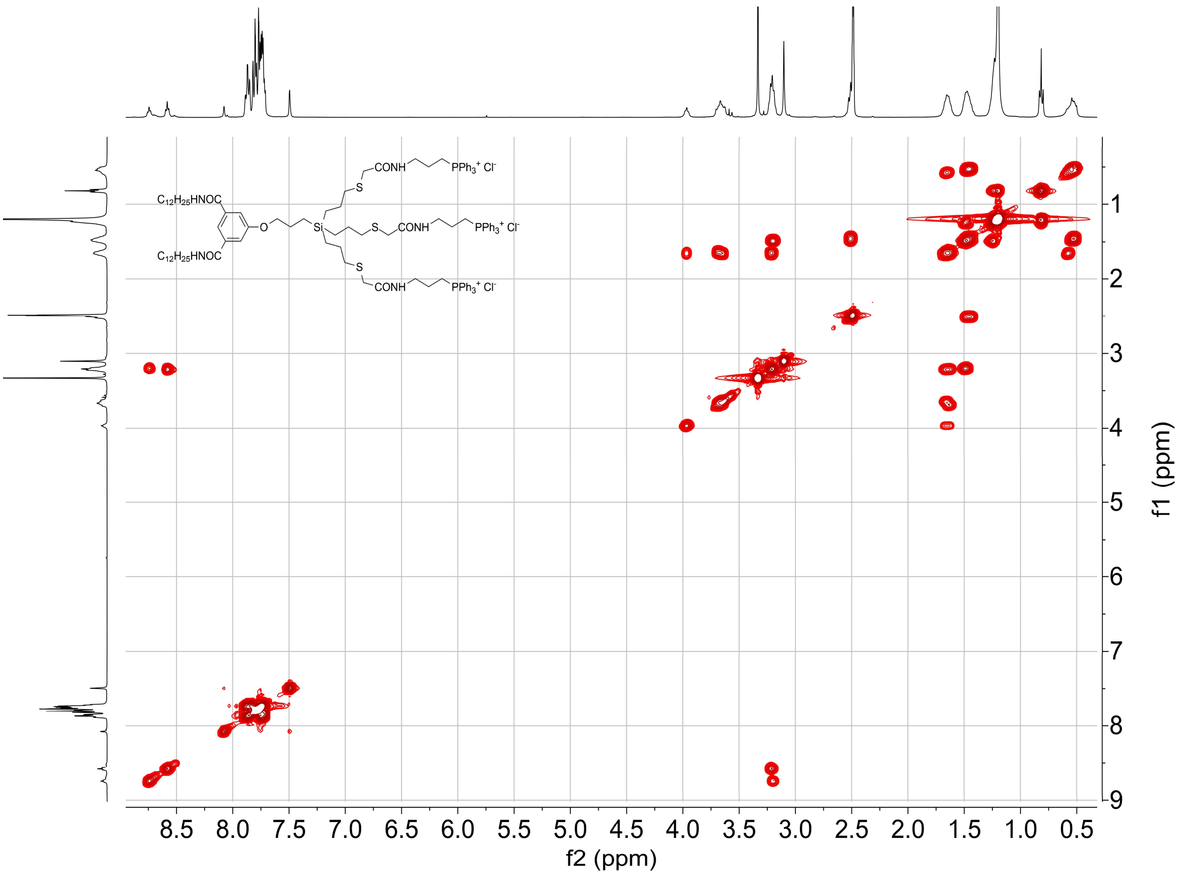

Figure S38. COSY NMR (400 MHz, DMSO-d_6) of **DnP$_3$-2C$_{12}$** (**3a**).

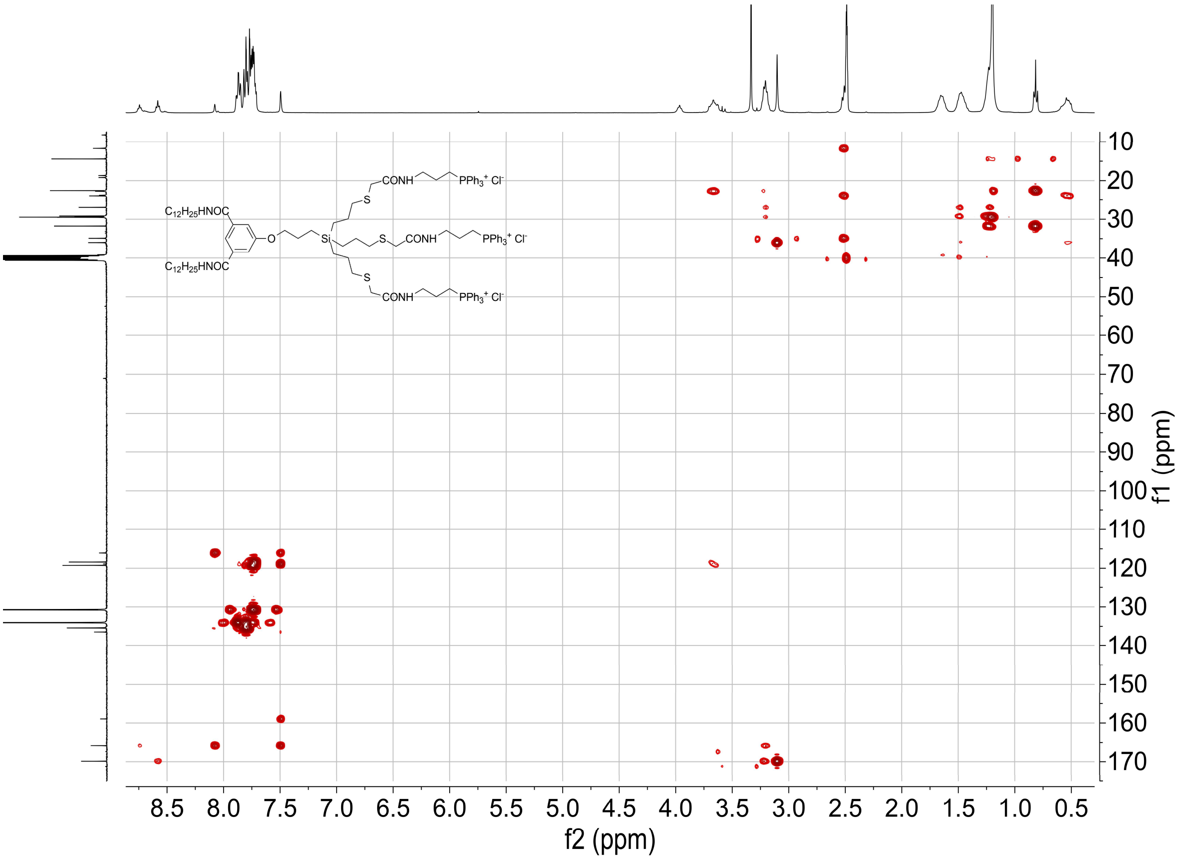

Figure S39. HMBC NMR (400 MHz, DMSO-d_6) of **DnP$_3$-2C$_{12}$** (**3a**).

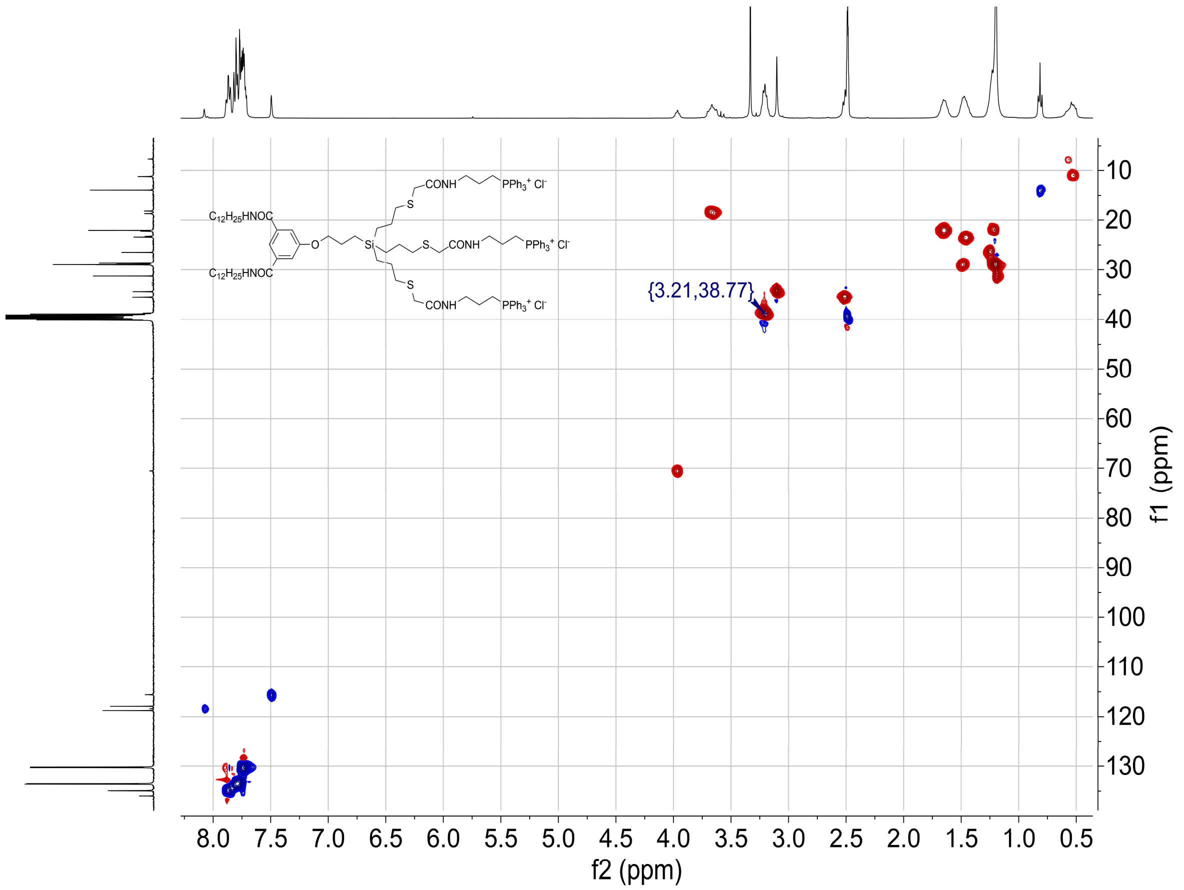

Figure S40. HSQC NMR (400 MHz, DMSO-d_6) of **DnP$_3$-2C$_{12}$** (**3a**).

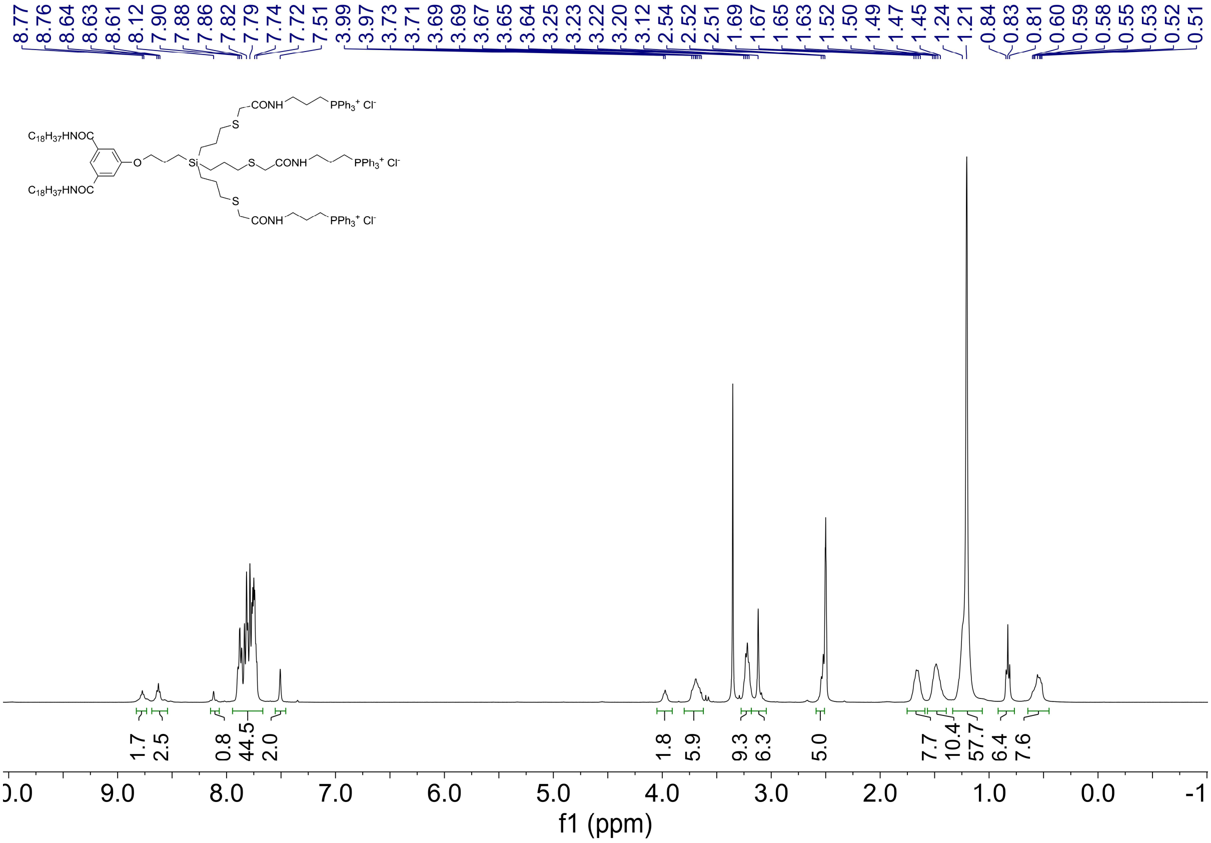

Figure S41. ^{1}H NMR (400 MHz, DMSO-d_6) of **DnP$_3$-2C$_{18}$** (**3b**).

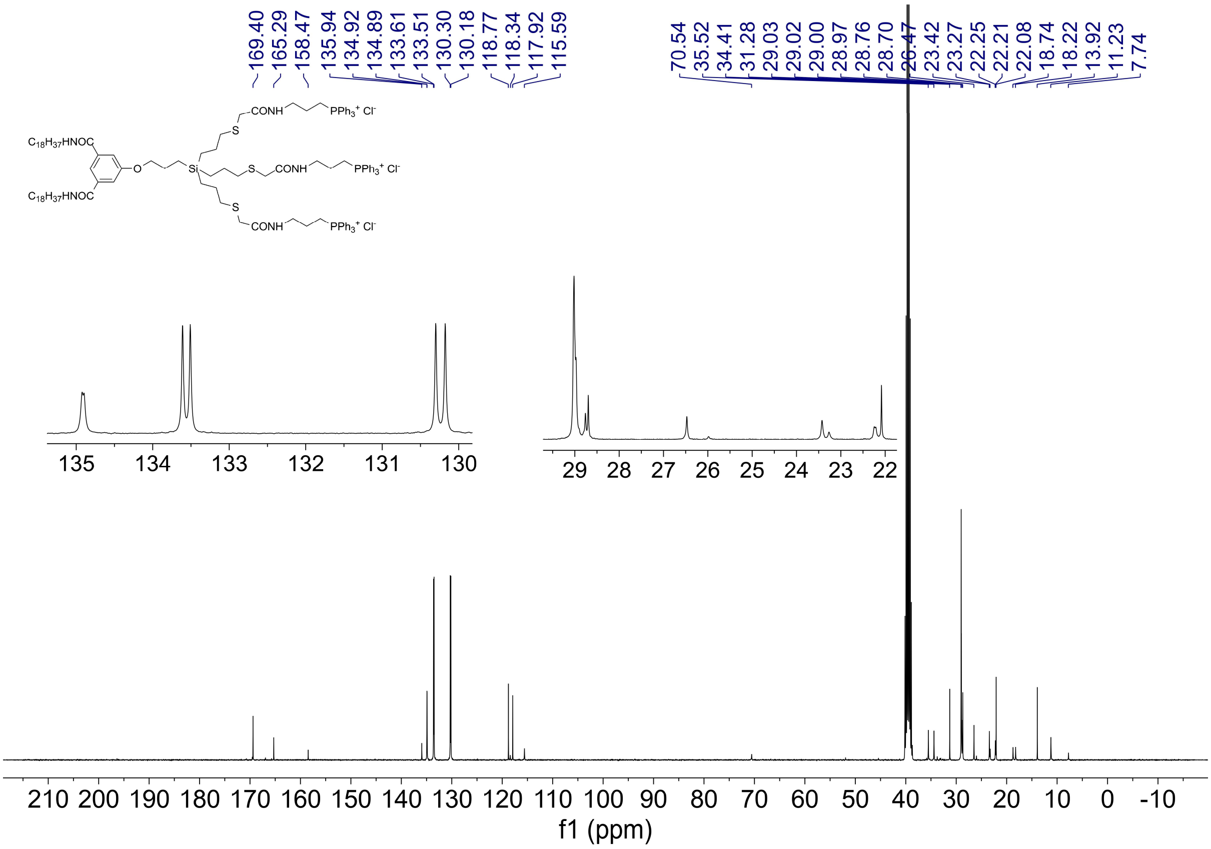

Figure S42. $^{13}C\{^1H\}$ NMR (400 MHz, DMSO-d_6) **DnP$_3$-2C$_{18}$** (**3b**).

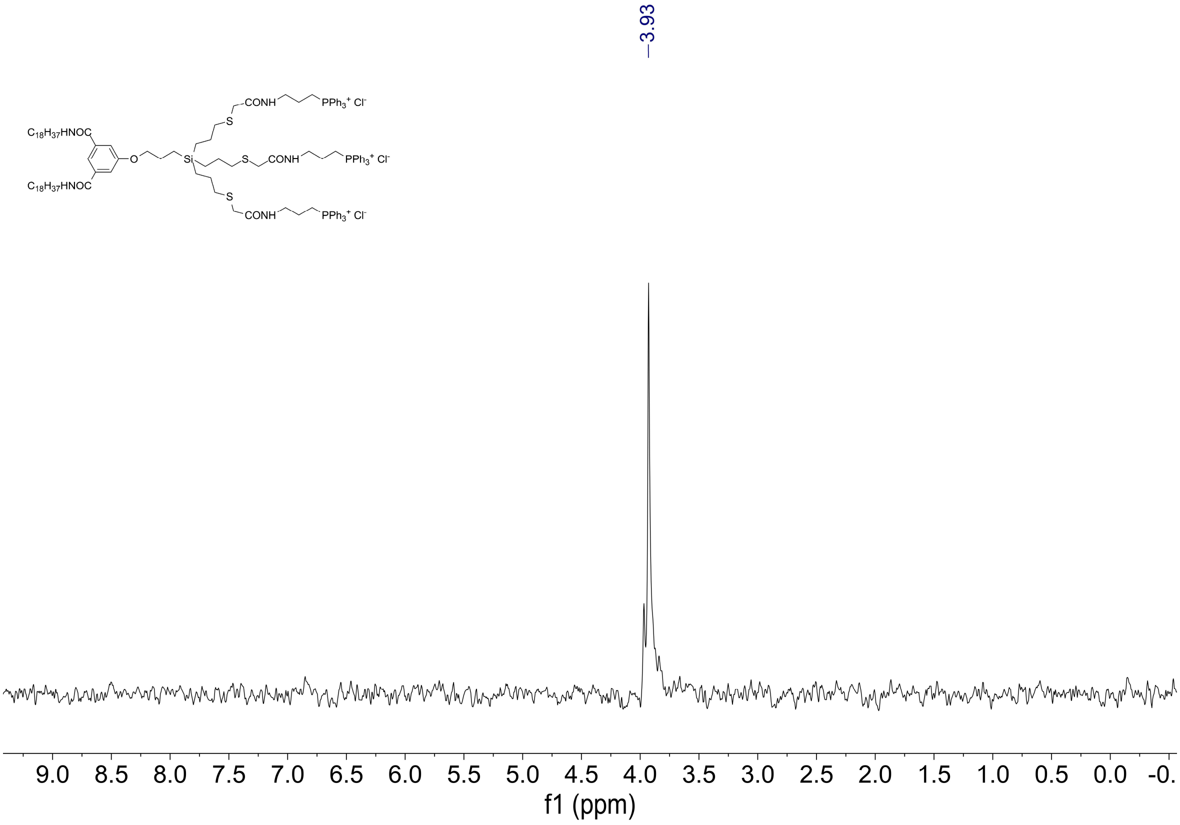

Figure S43. $^{29}Si\{^1H\}$ NMR (400 MHz, DMSO-d_6) **DnP$_3$-2C$_{18}$** (**3b**).

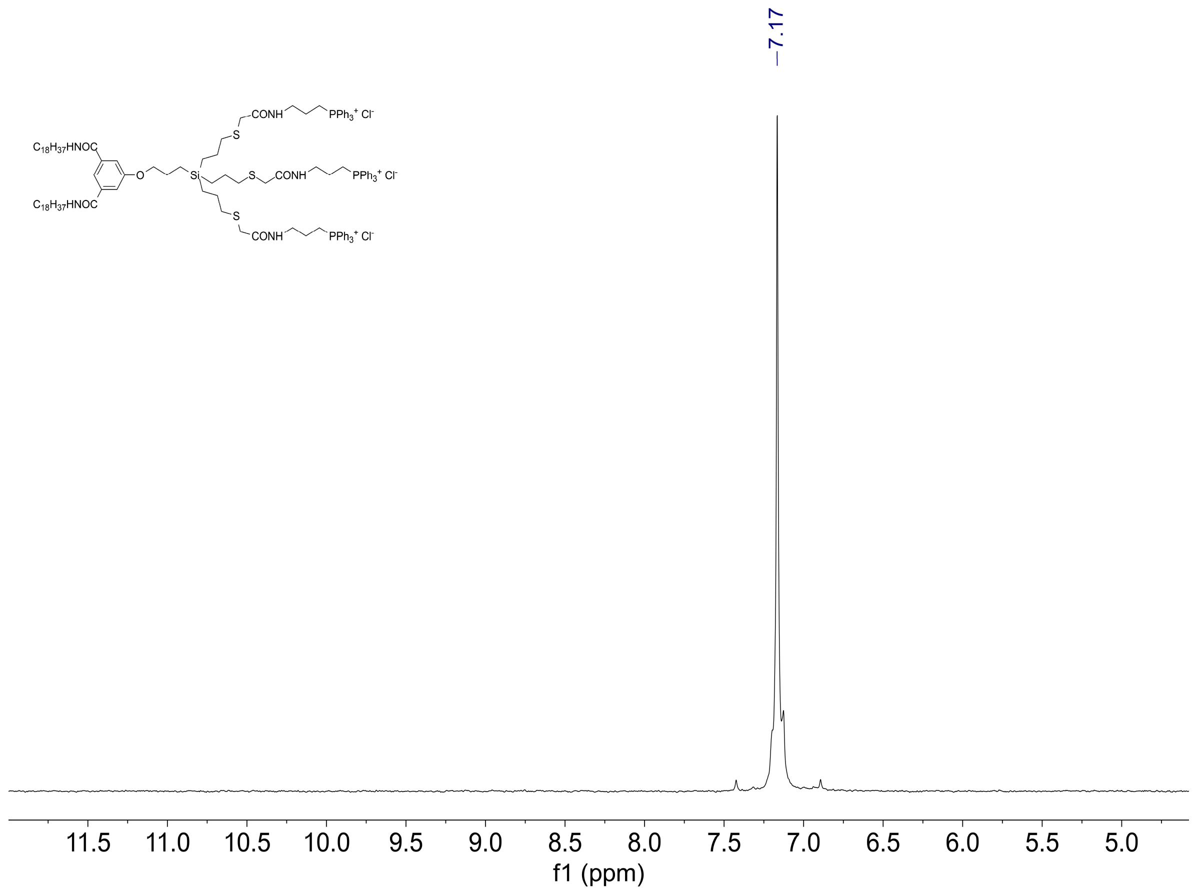

Figure S44. $^{31}P\{^1H\}$ NMR (400 MHz, DMSO-d_6) **DnP$_3$-2C$_{18}$** (**3b**).

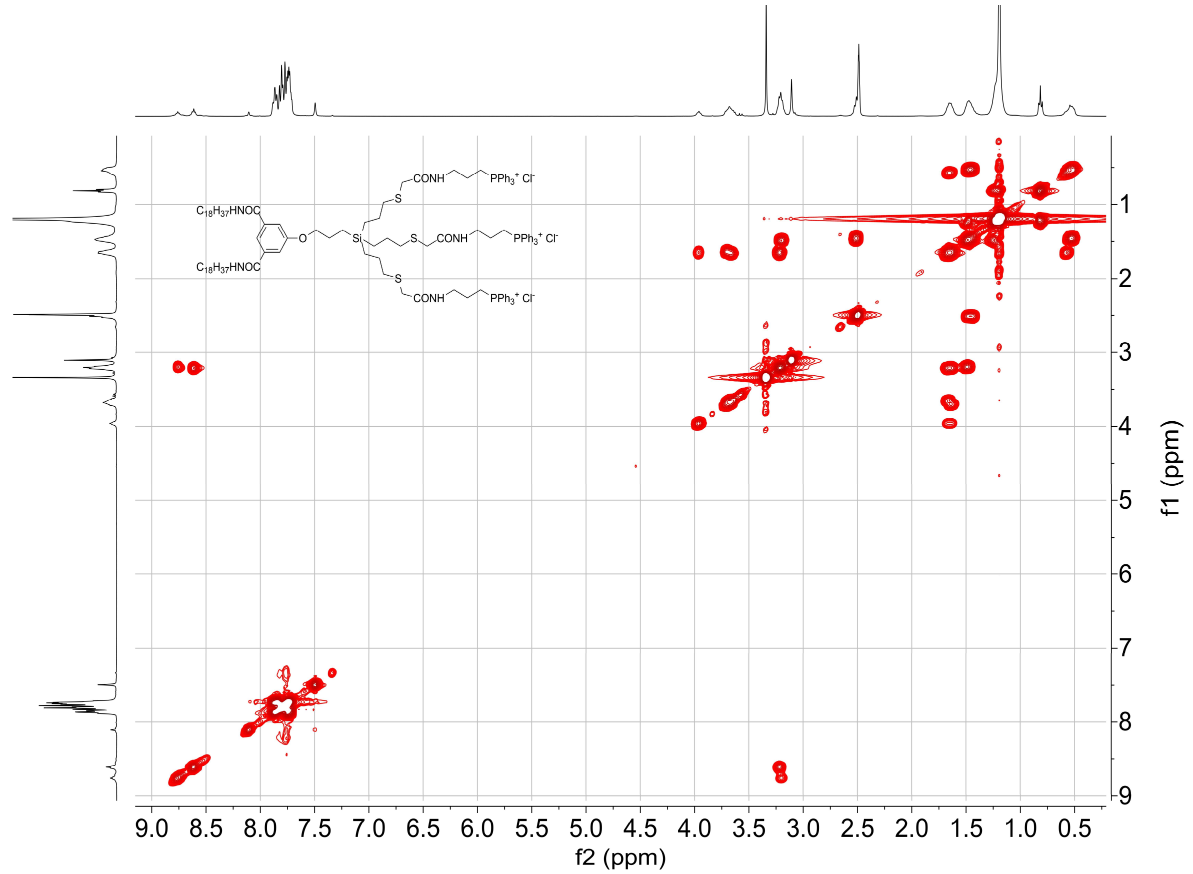

Figure S45. COSY NMR (400 MHz, DMSO-d_6) **DnP$_3$-2C$_{18}$** (**3b**).

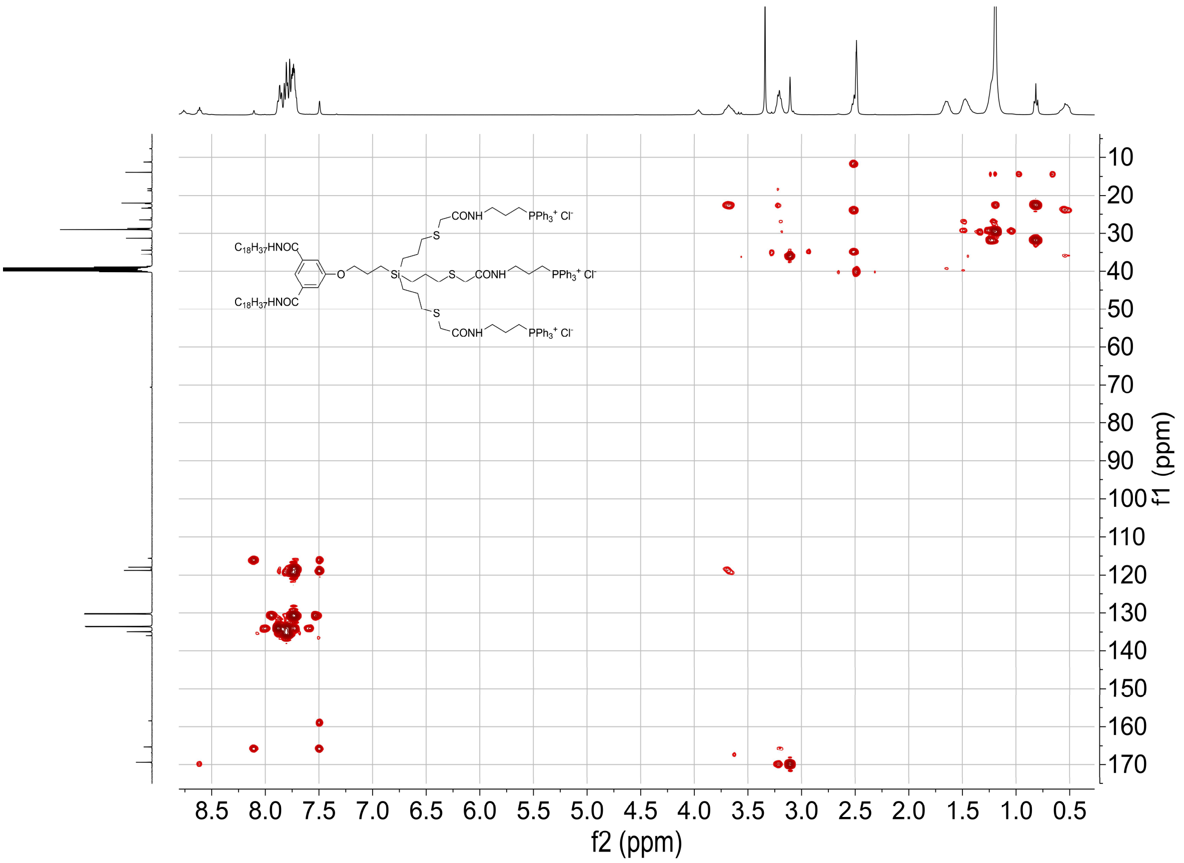

Figure S46. HMBC NMR (400 MHz, DMSO-d_6) **DnP$_3$-2C$_{18}$** (**3b**).

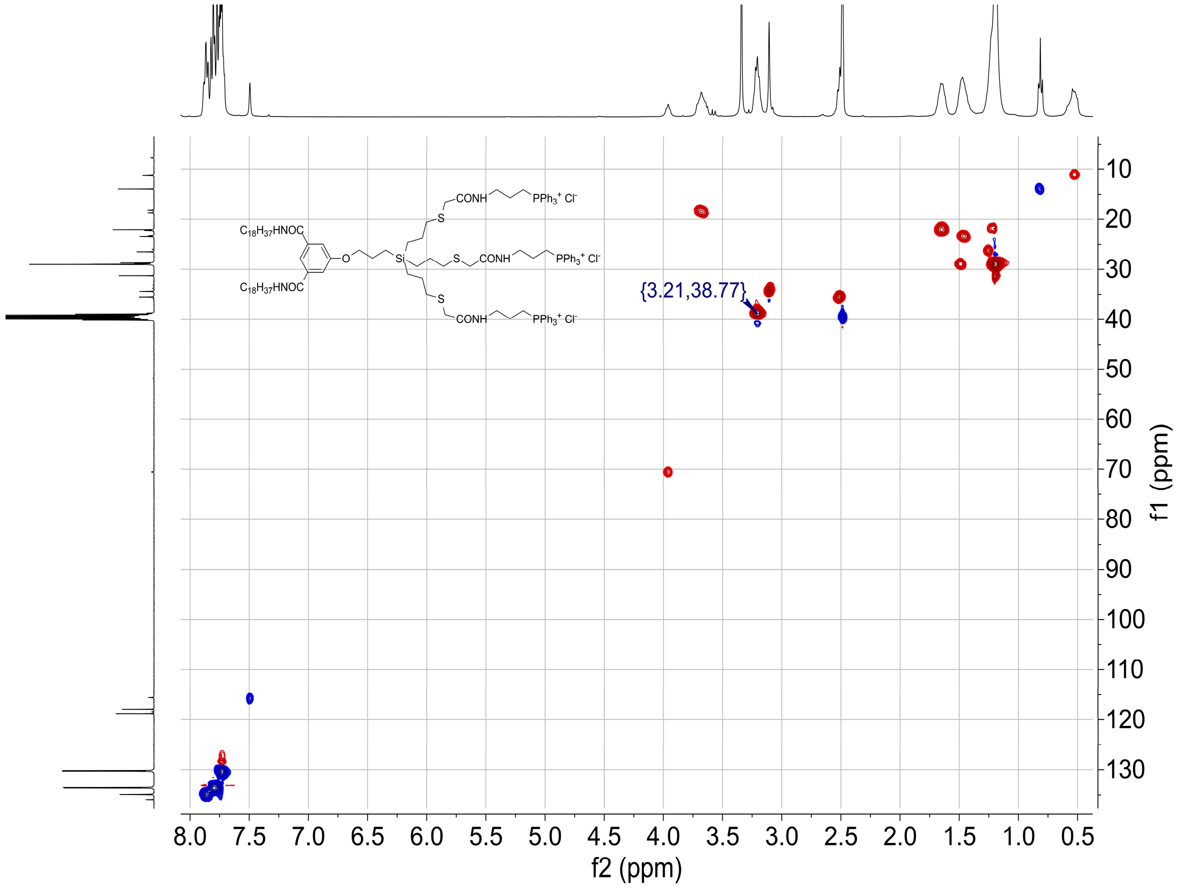

Figure S47. HSQC NMR (400 MHz, DMSO-d_6) **DnP$_3$-2C$_{18}$** (**3b**).

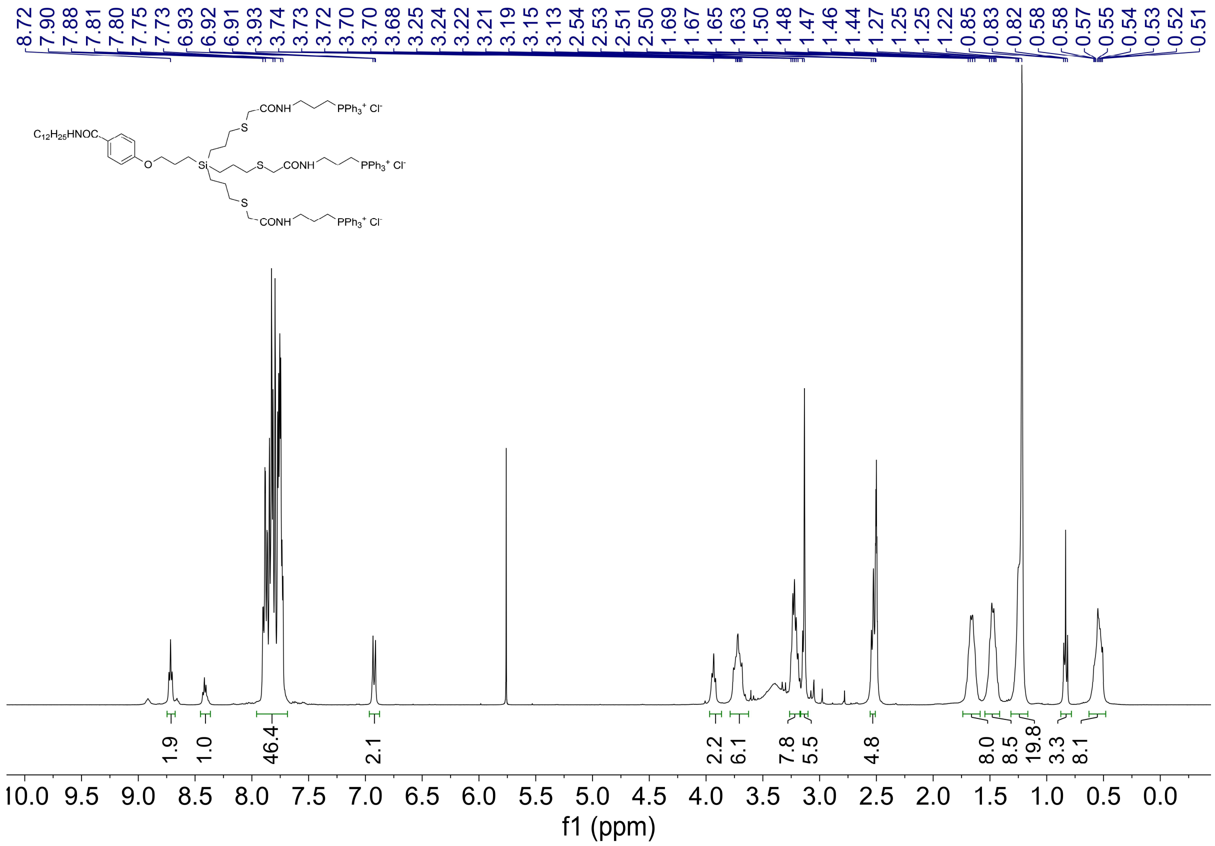

Figure S48. ^{1}H NMR (400 MHz, DMSO-d_6) of **DnP$_3$-1C$_{12}$** (**6a**).

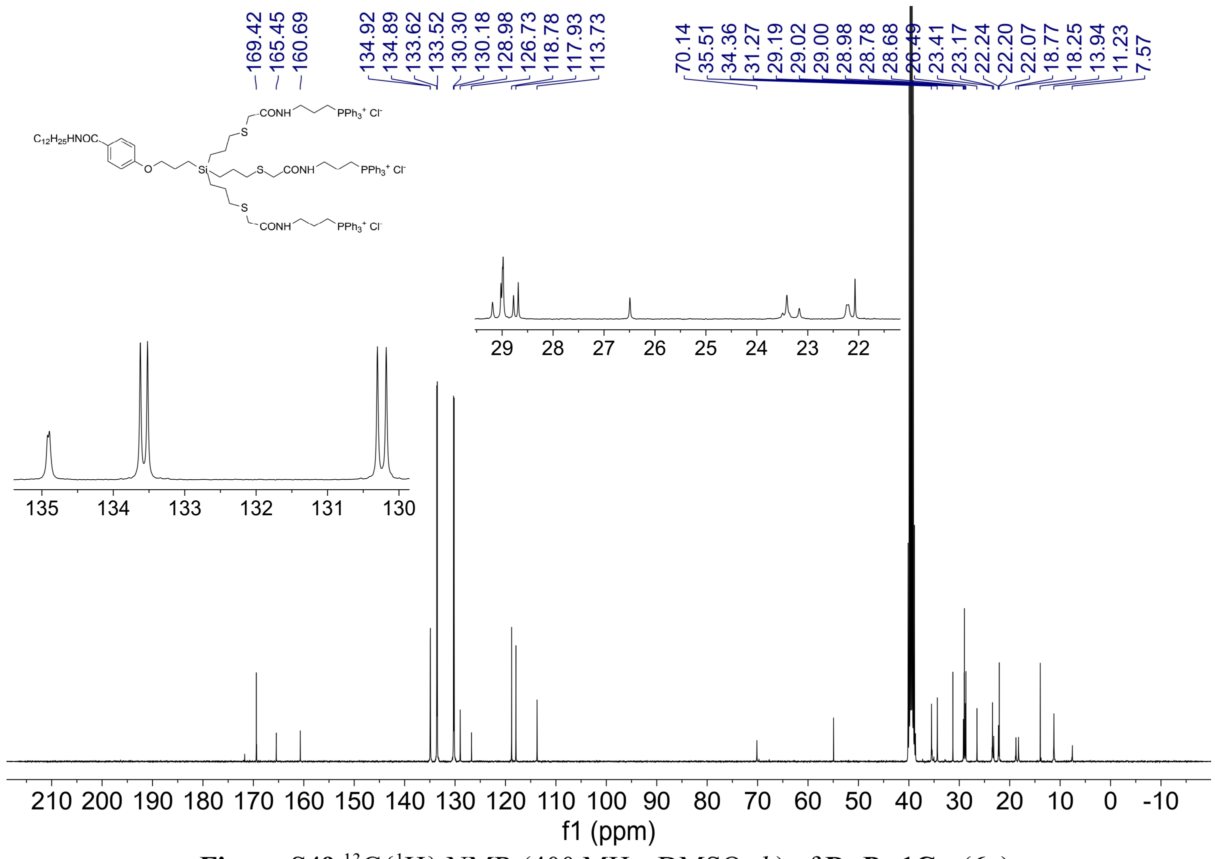

Figure S49.^{13}C{^{1}H} NMR (400 MHz, DMSO-d_6) of **DnP$_3$-1C$_{12}$** (**6a**).

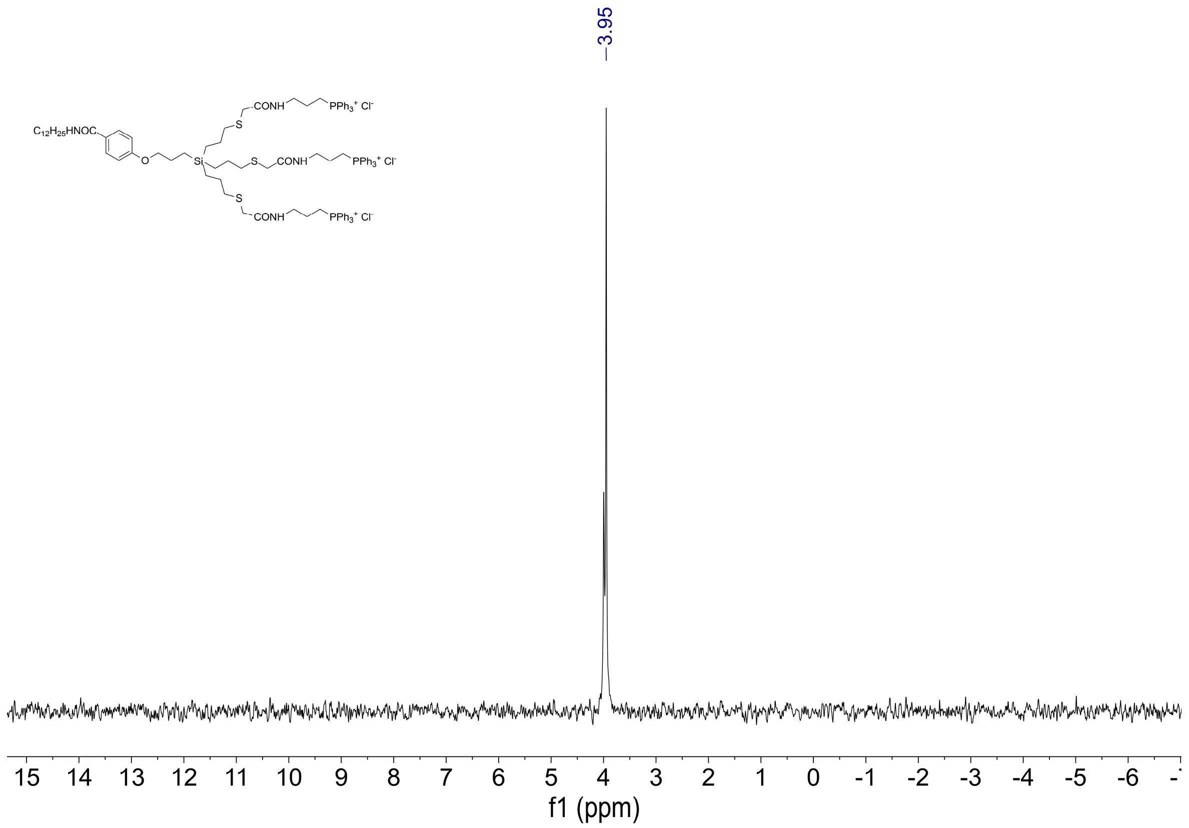

Figure S50. $^{29}Si\{^{1}H\}$ NMR (400 MHz, DMSO-d_6) of **DnP$_3$-1C$_{12}$** (**6a**).

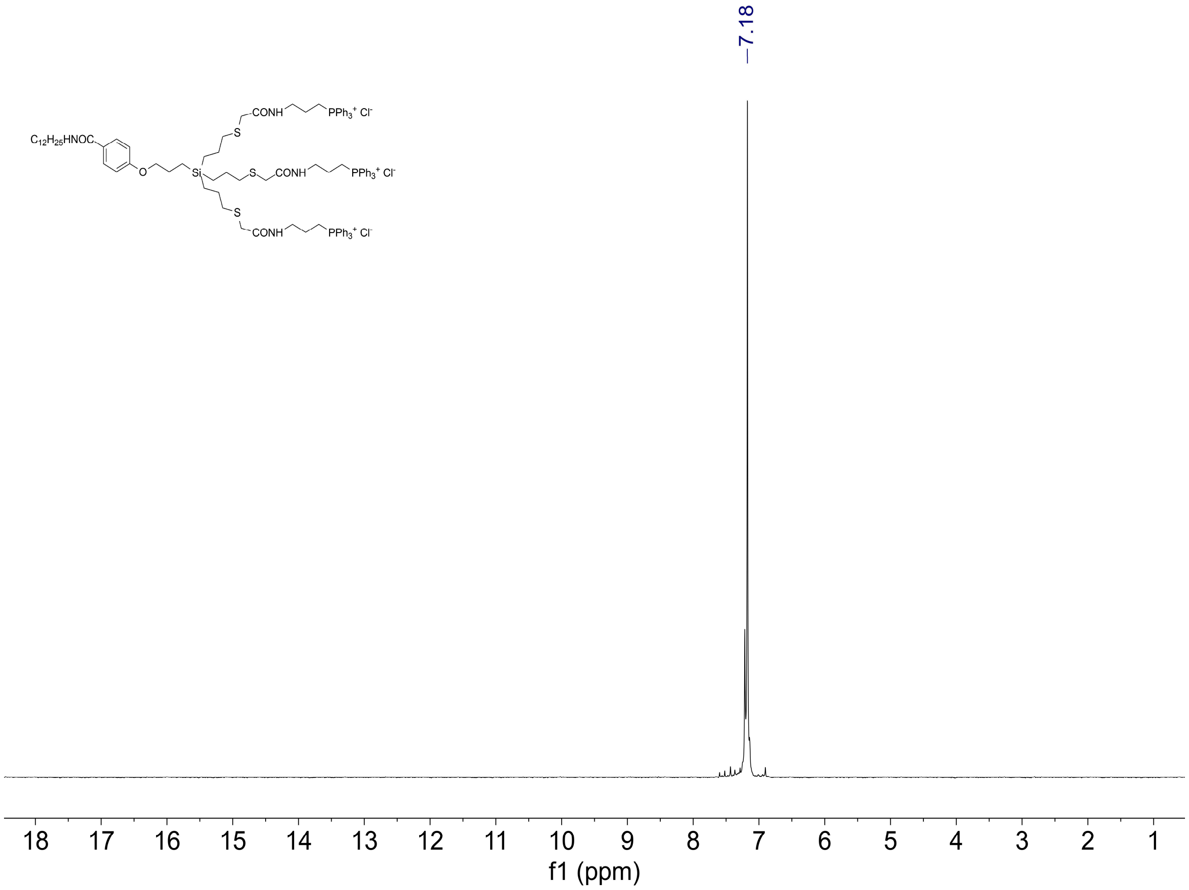

Figure S51. $^{31}P\{^{1}H\}$ NMR (400 MHz, DMSO-d_6) of **DnP$_3$-1C$_{12}$** (**6a**).

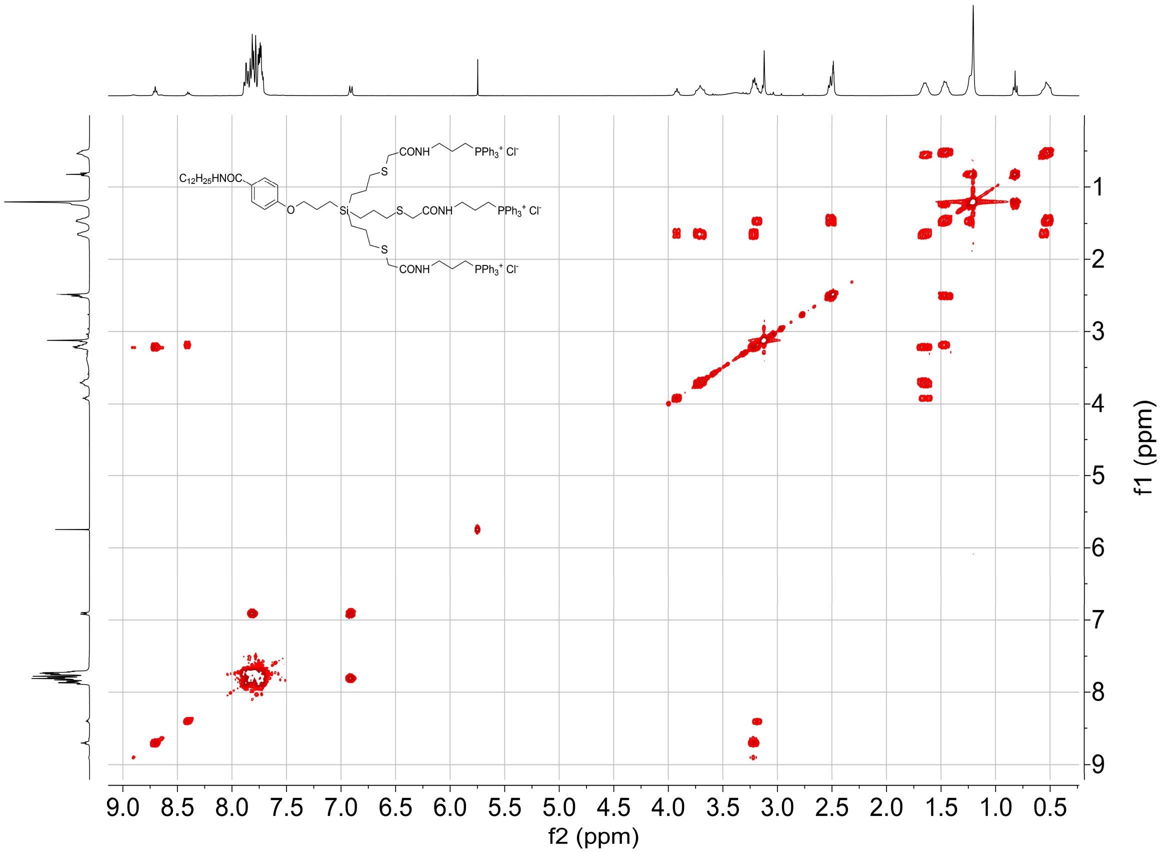

Figure S52.COSY NMR (400 MHz, DMSO-d_6) of **DnP$_3$-1C$_{12}$** (**6a**).

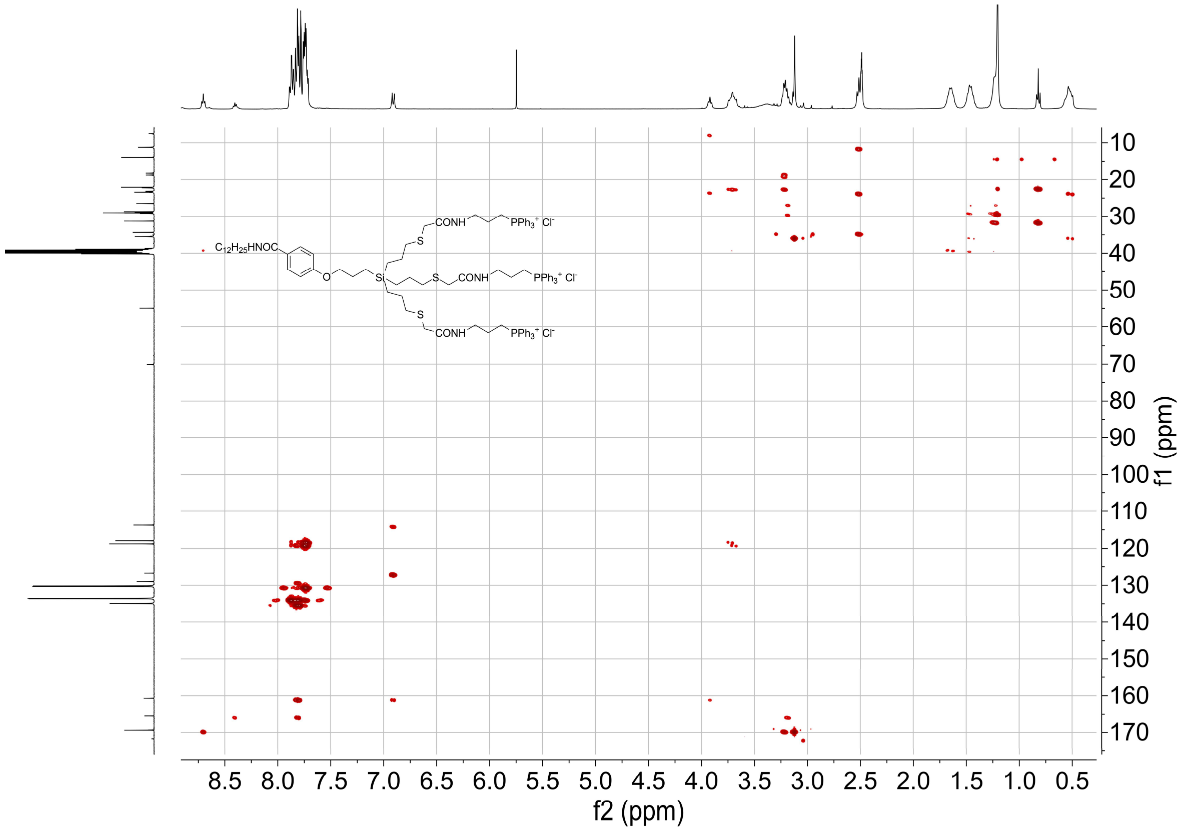

Figure S53. HMBC NMR (400 MHz, DMSO-d_6) of **DnP$_3$-1C$_{12}$** (**6a**).

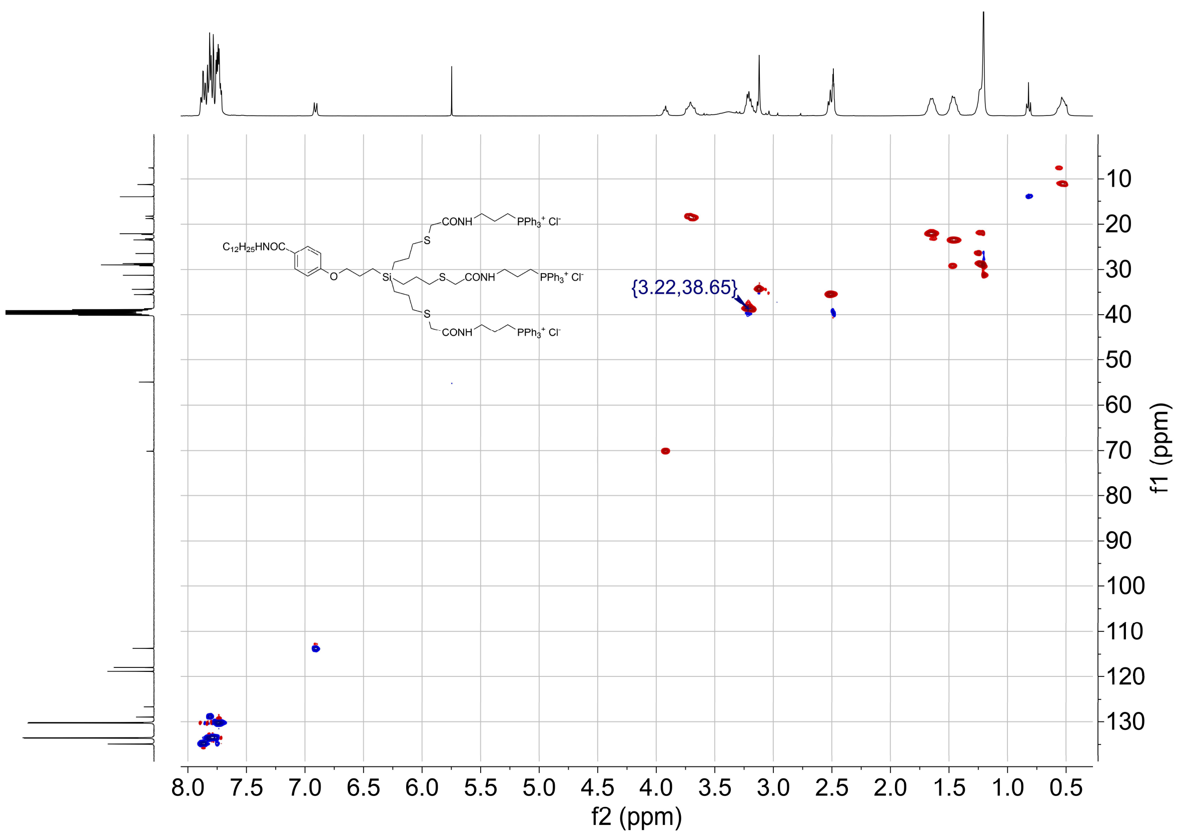

Figure S54. HSQC NMR (400 MHz, DMSO-d_6) of **DnP$_3$-1C$_{12}$** (**6a**).

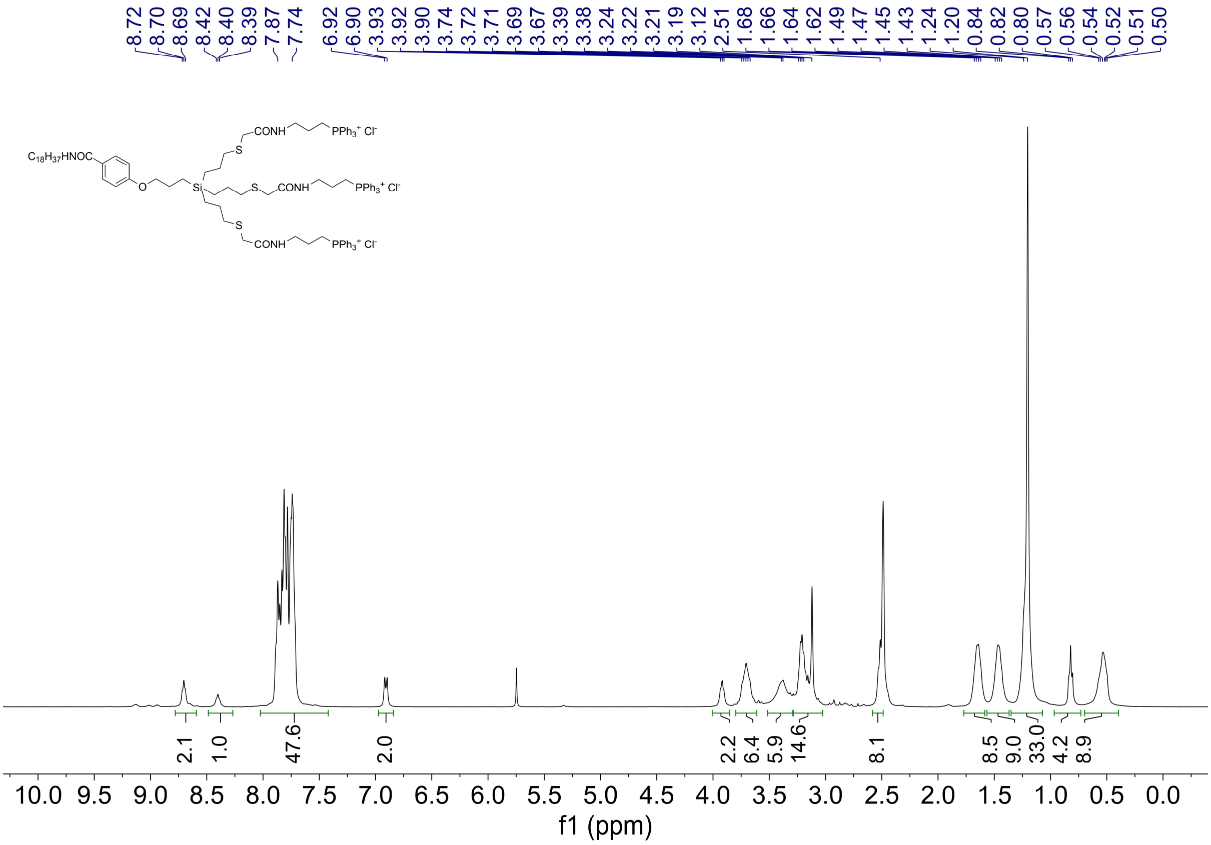

Figure S55. ^{1}H NMR (400 MHz, DMSO-d_6) of **DnP$_3$-1C$_{18}$** (**6b**).

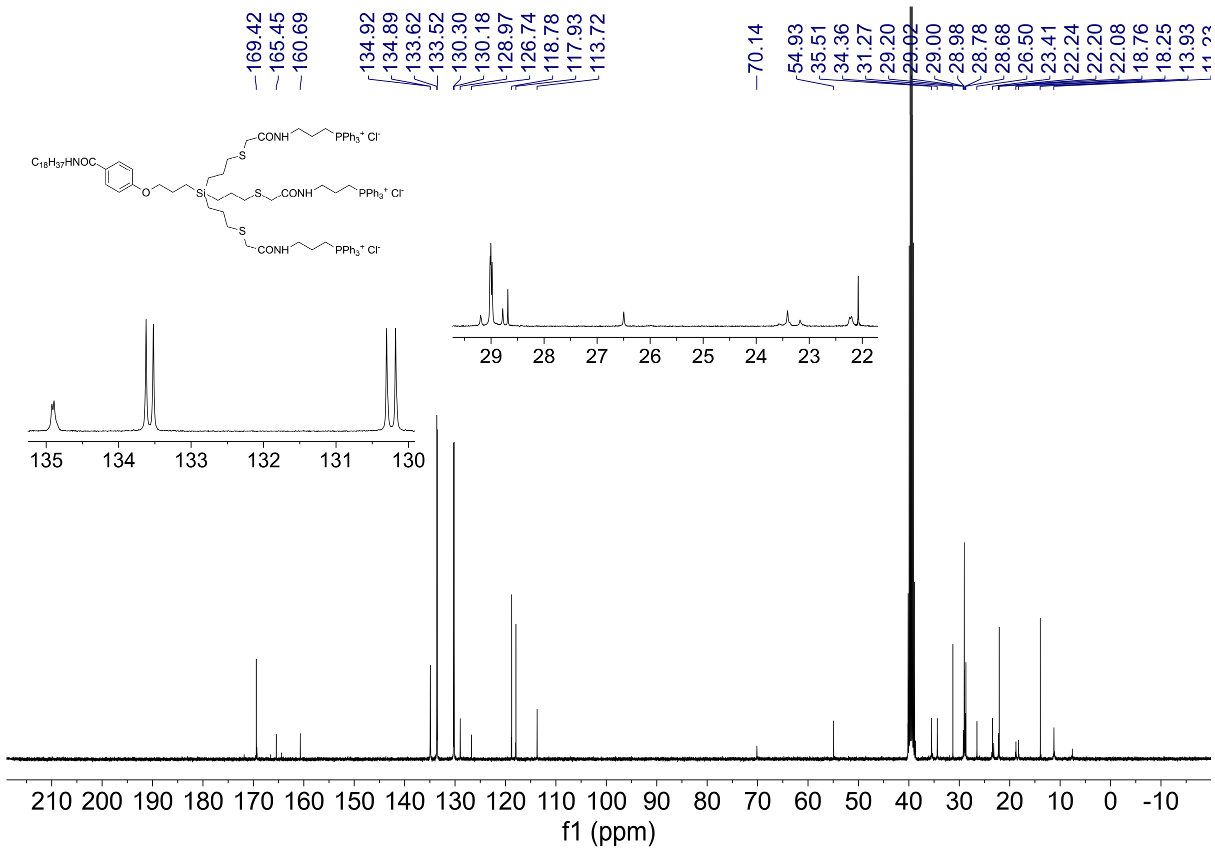

Figure S56. $^{13}C\{^{1}H\}$ NMR (400 MHz, DMSO-d_6) of **DnP$_3$-1C$_{18}$** (**6b**).

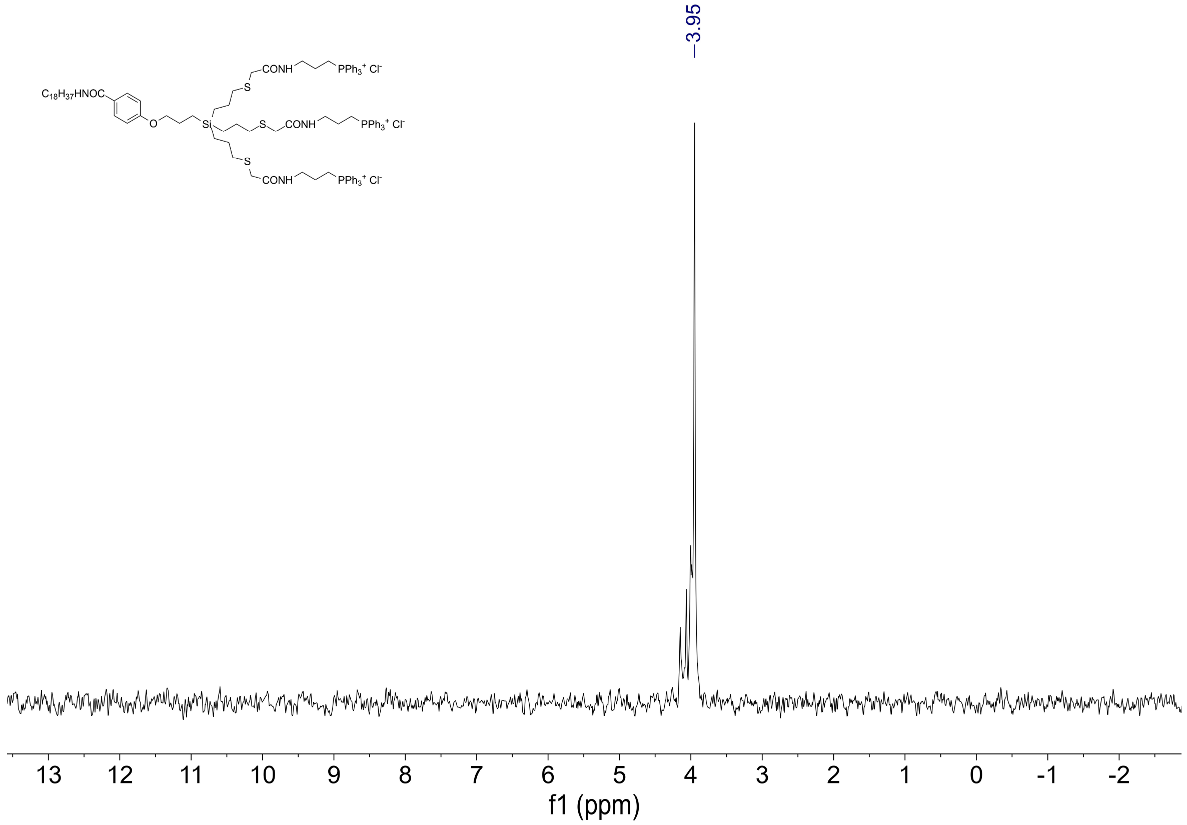

Figure S57. $^{29}Si\{^{1}H\}$ NMR (400 MHz, DMSO-d_6) of **DnP$_3$-1C$_{18}$** (**6b**).

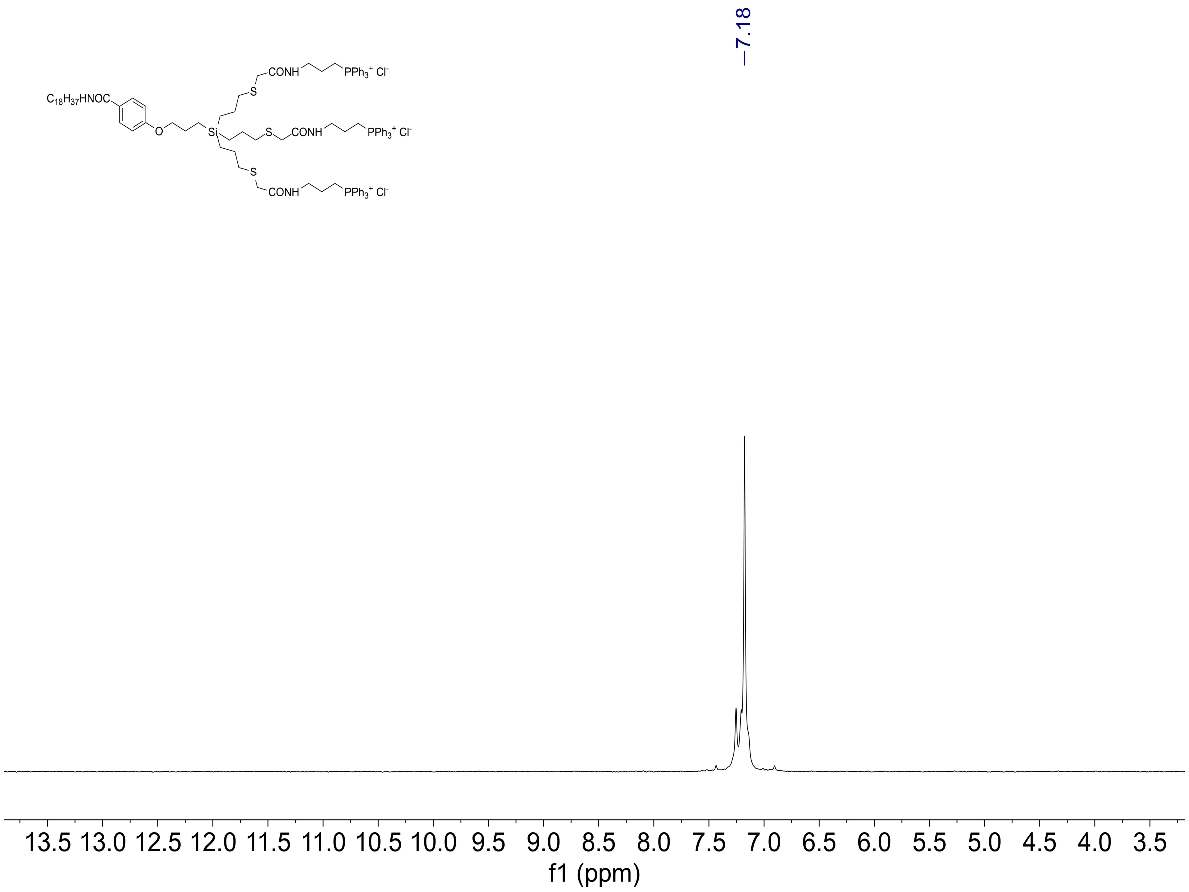

Figure S58. $^{31}P\{^{1}H\}$ NMR (400 MHz, DMSO-d_6) of **DnP$_3$-1C$_{18}$** (**6b**).

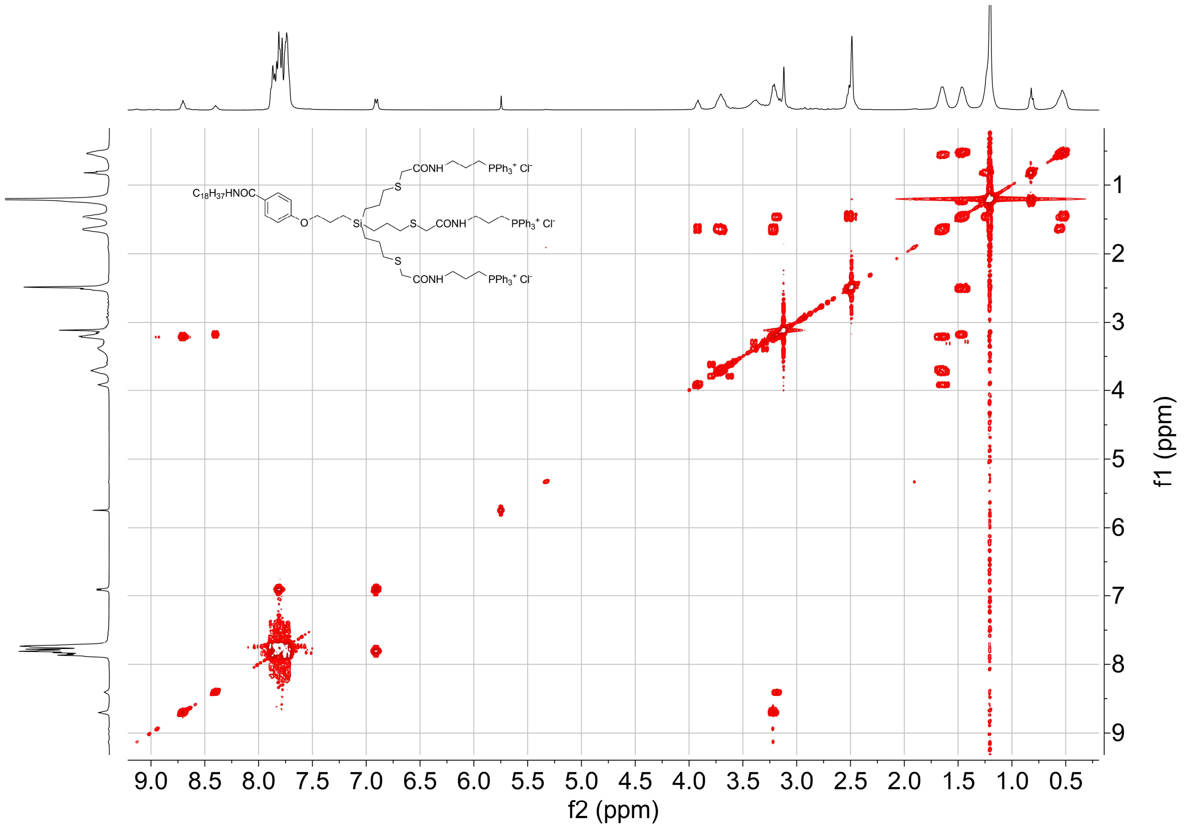

Figure S59. COSY NMR (400 MHz, DMSO-d_6) of **DnP$_3$-1C$_{18}$** (**6b**).

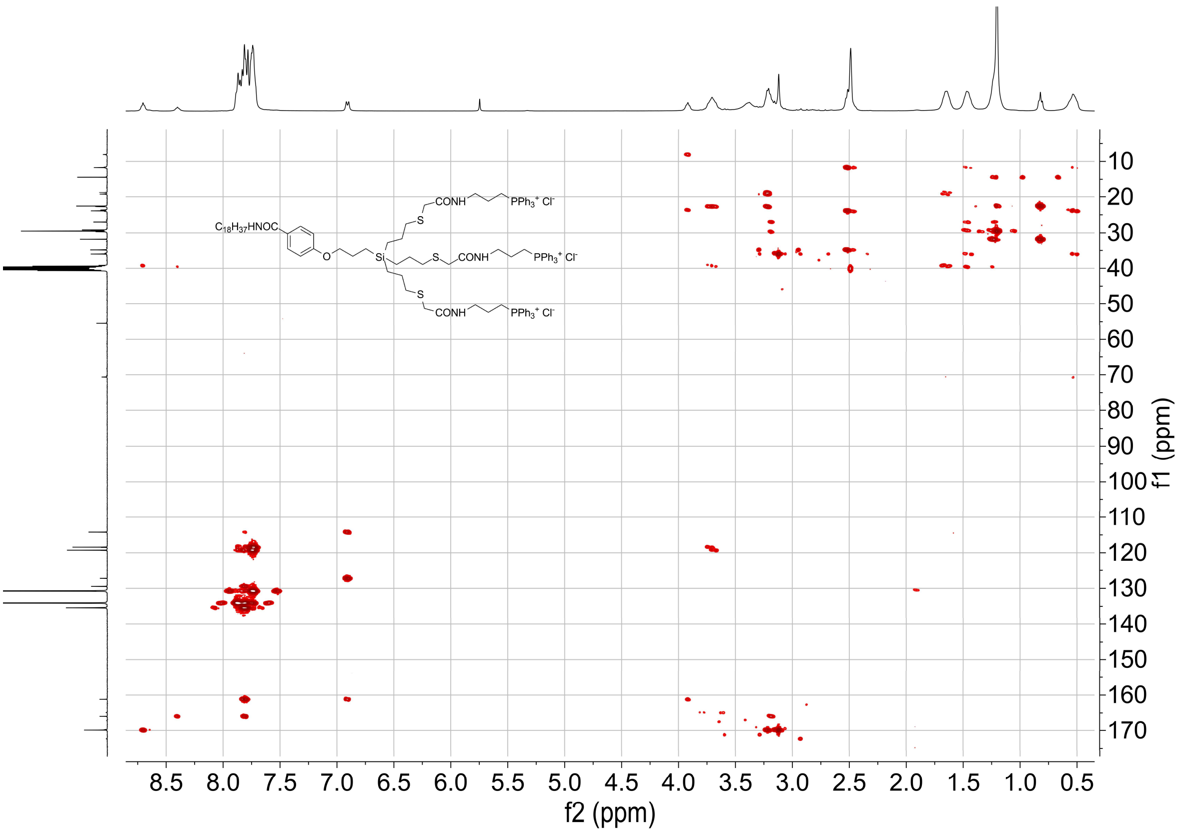

Figure S60. HMBC NMR (400 MHz, DMSO-d_6) of **DnP$_3$-1C$_{18}$ (6b)**.

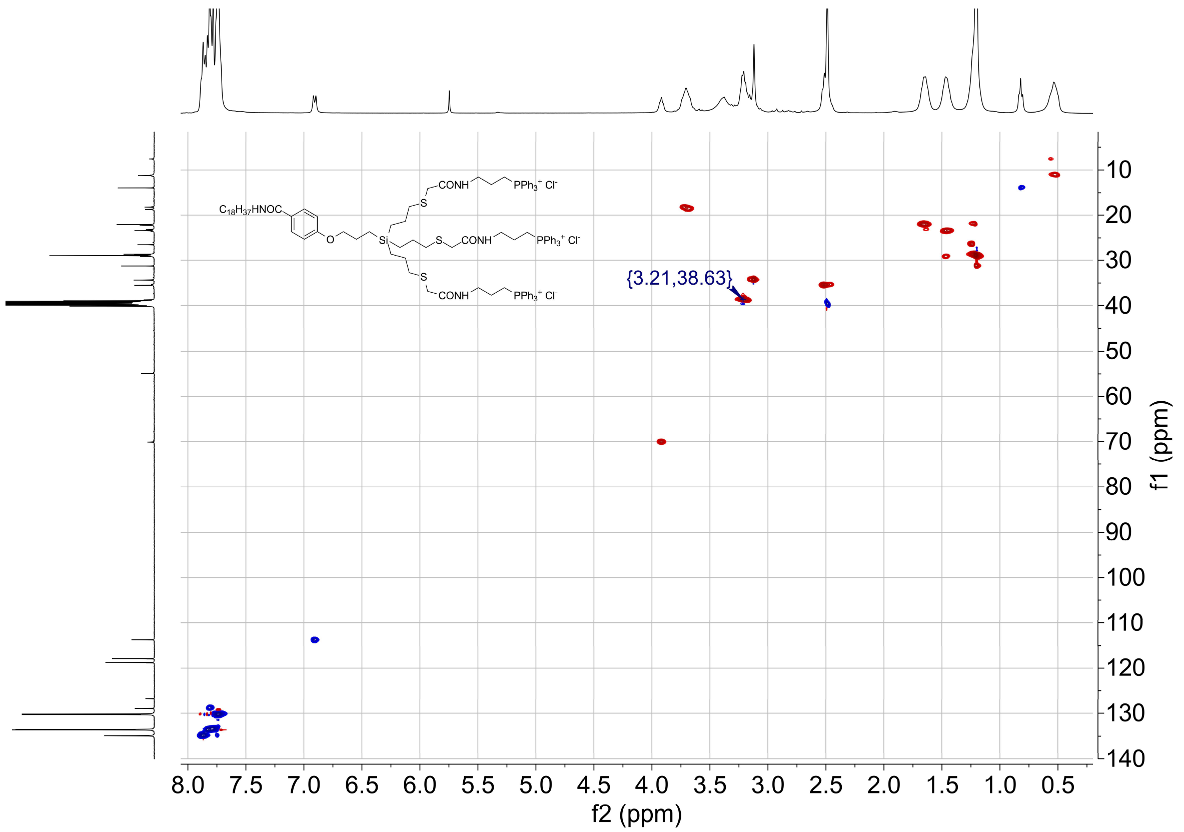

Figure S61. HSQC NMR (400 MHz, DMSO-d_6) of **DnP$_3$-1C$_{18}$ (6b)**.

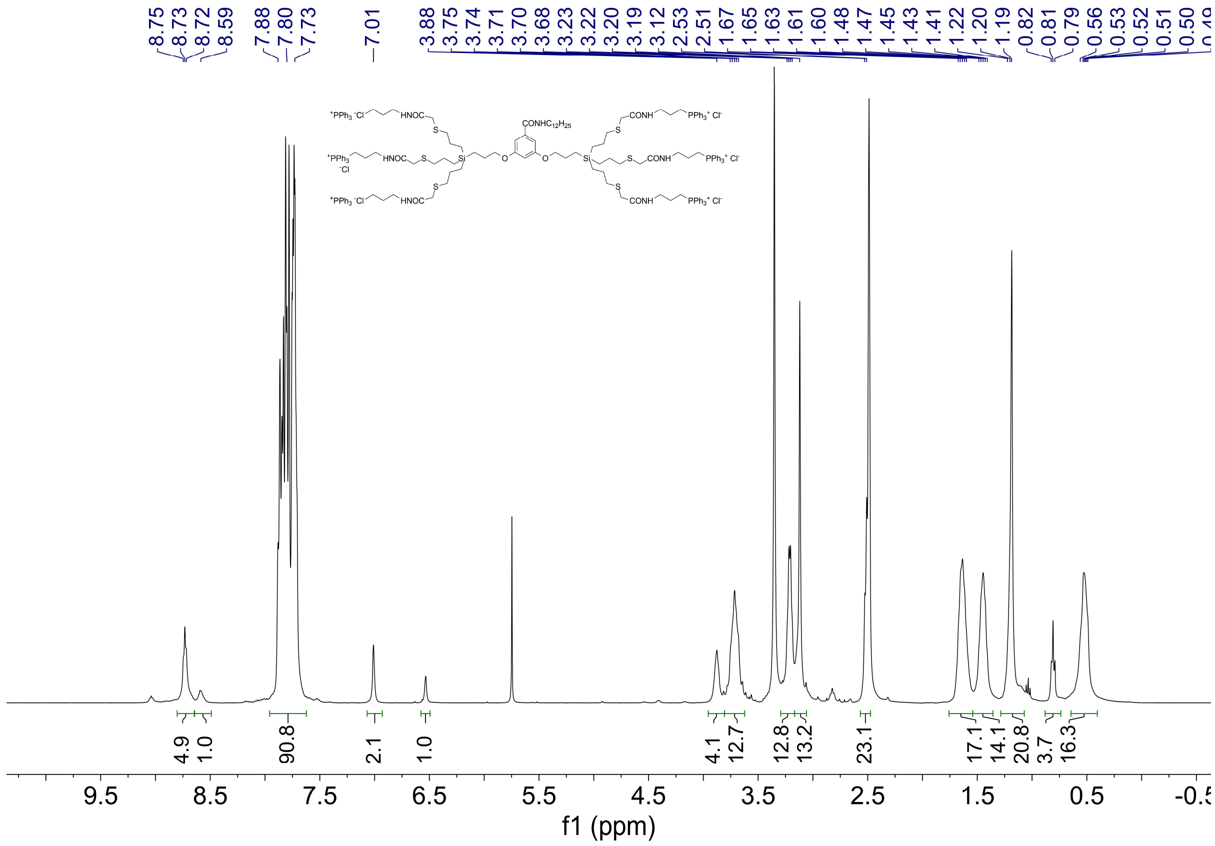

Figure S62. ^{1}H NMR (400 MHz, DMSO-d_6) of **DnP$_6$-1C$_{12}$ (9a)**.

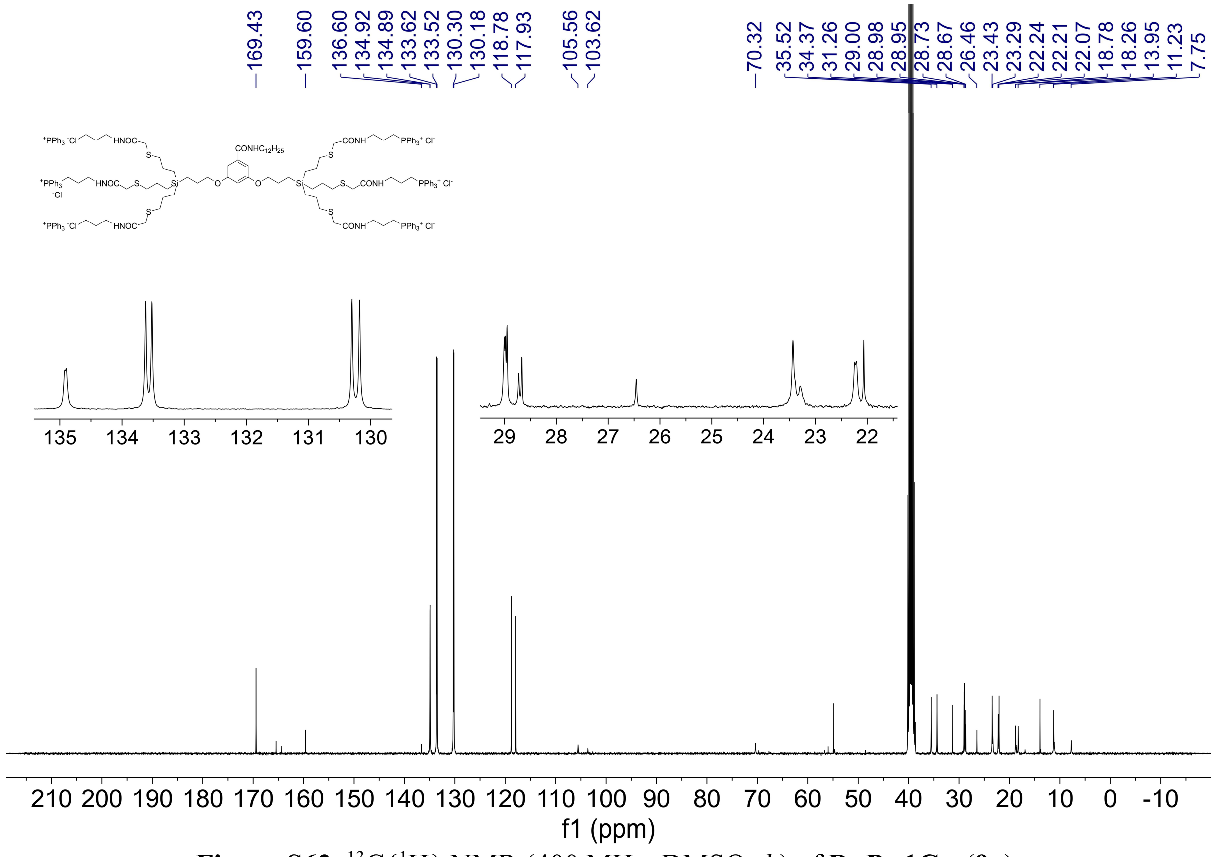

Figure S63. ^{13}C{^{1}H} NMR (400 MHz, DMSO-d_6) of **DnP$_6$-1C$_{12}$ (9a)**.

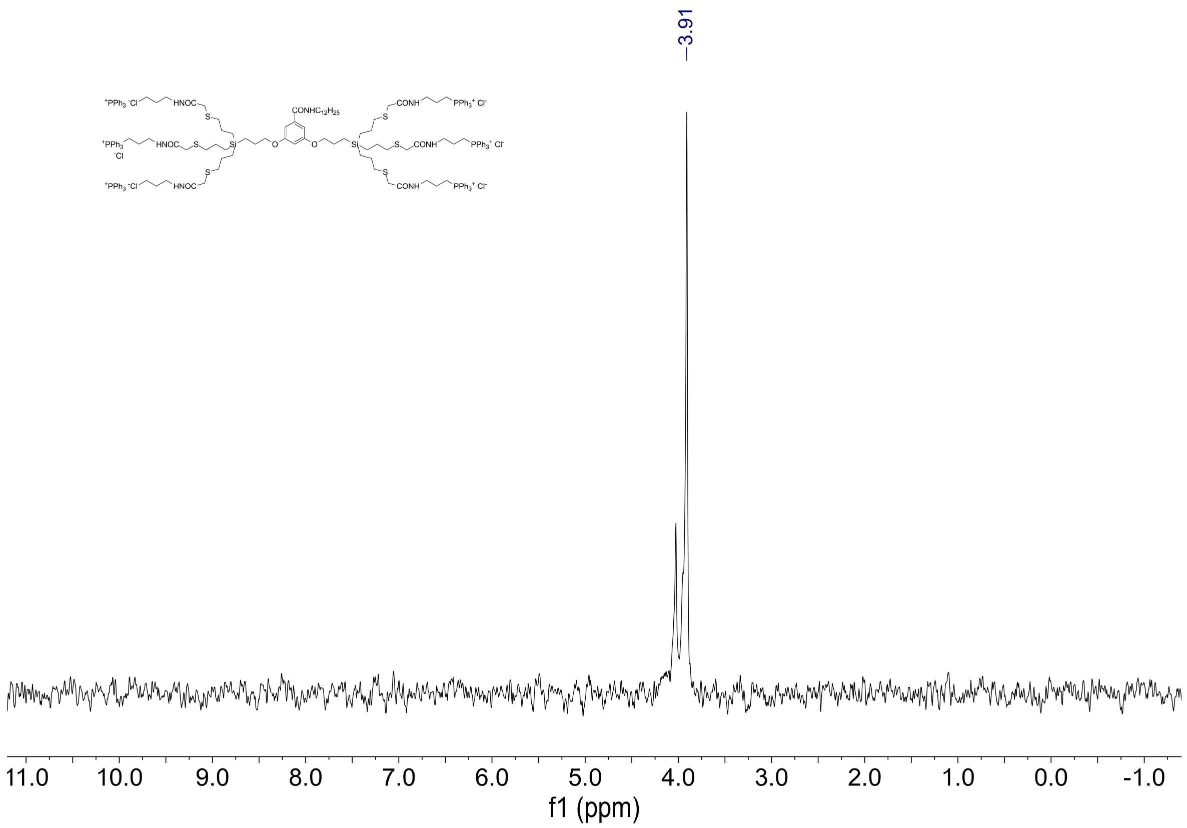

Figure S64. $^{29}Si\{^{1}H\}$ NMR (400 MHz, DMSO-d_6) of **DnP$_6$-1C$_{12}$** (**9a**).

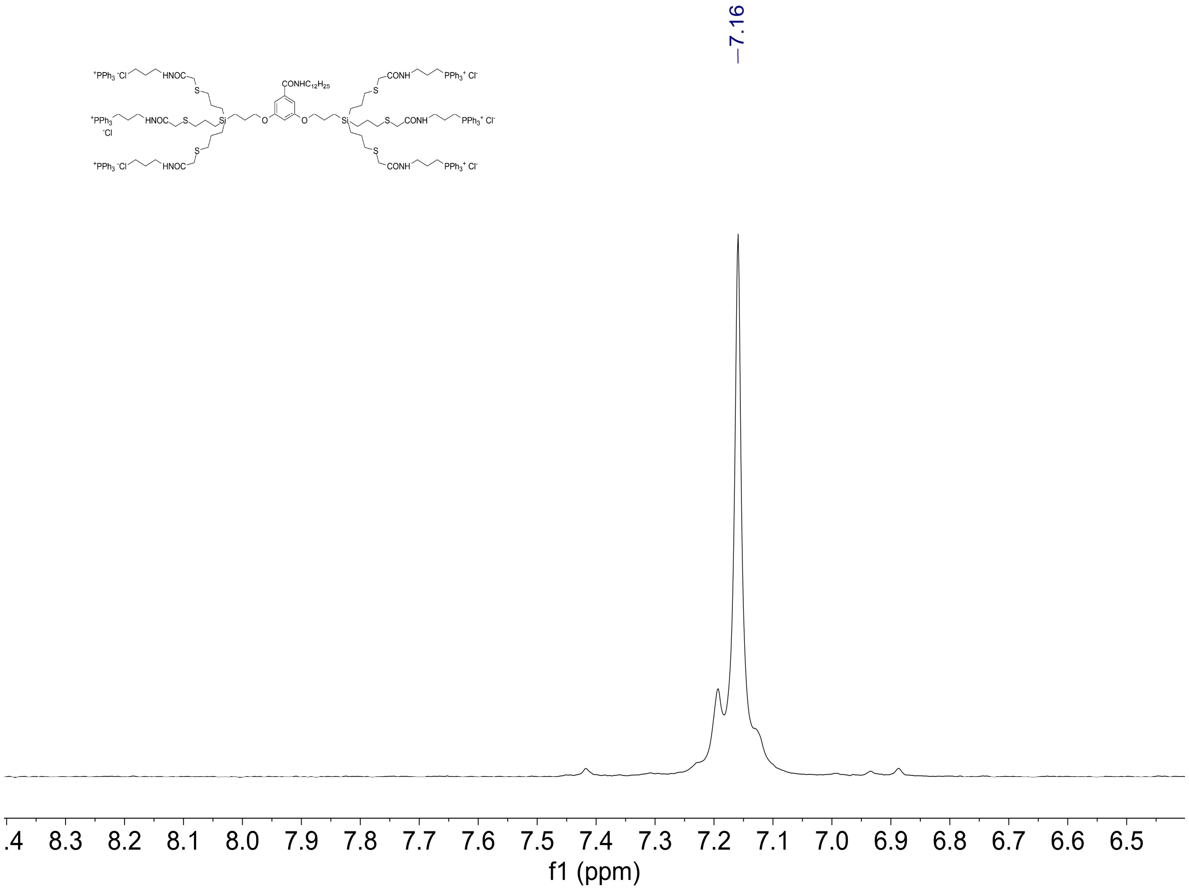

Figure S65. $^{31}P\{^{1}H\}$ NMR (400 MHz, DMSO-d_6) of **DnP$_6$-1C$_{12}$** (**9a**).

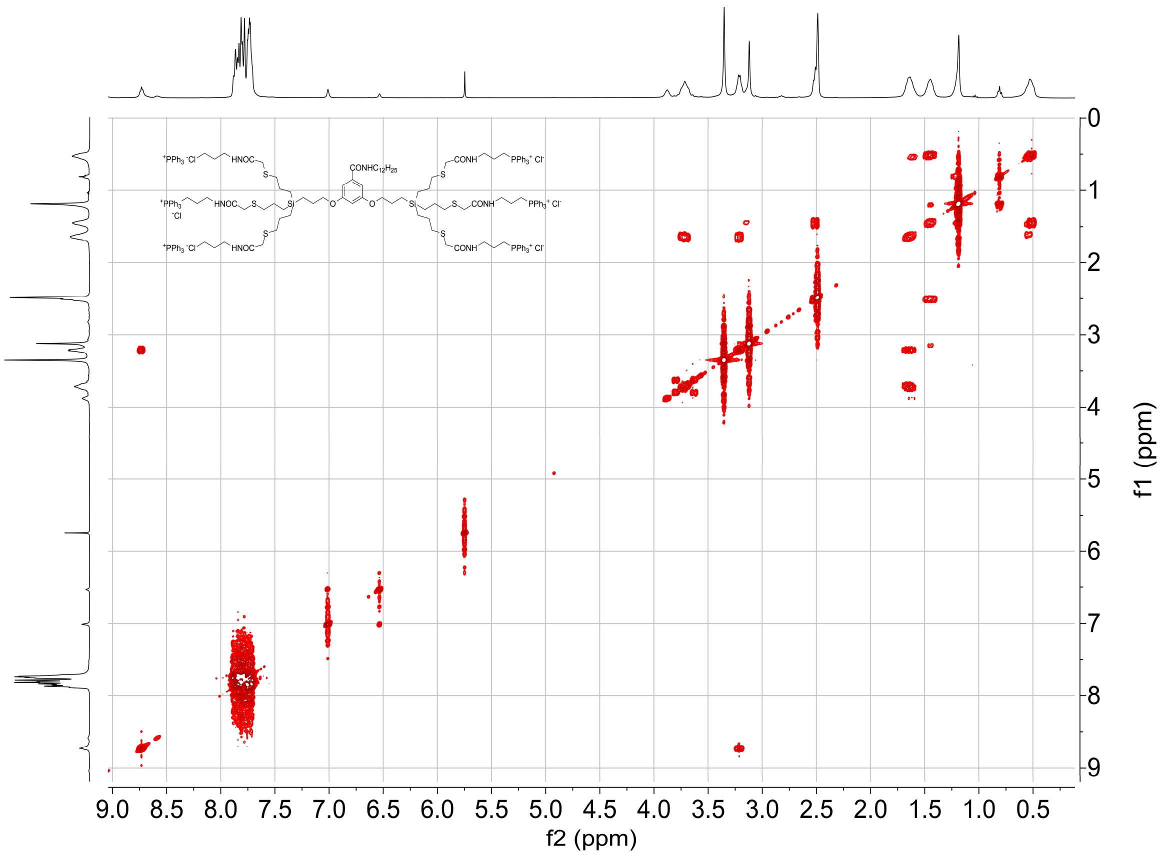

Figure S66. COSY NMR (400 MHz, DMSO-d_6) of **DnP$_6$-1C$_{12}$** (**9a**).

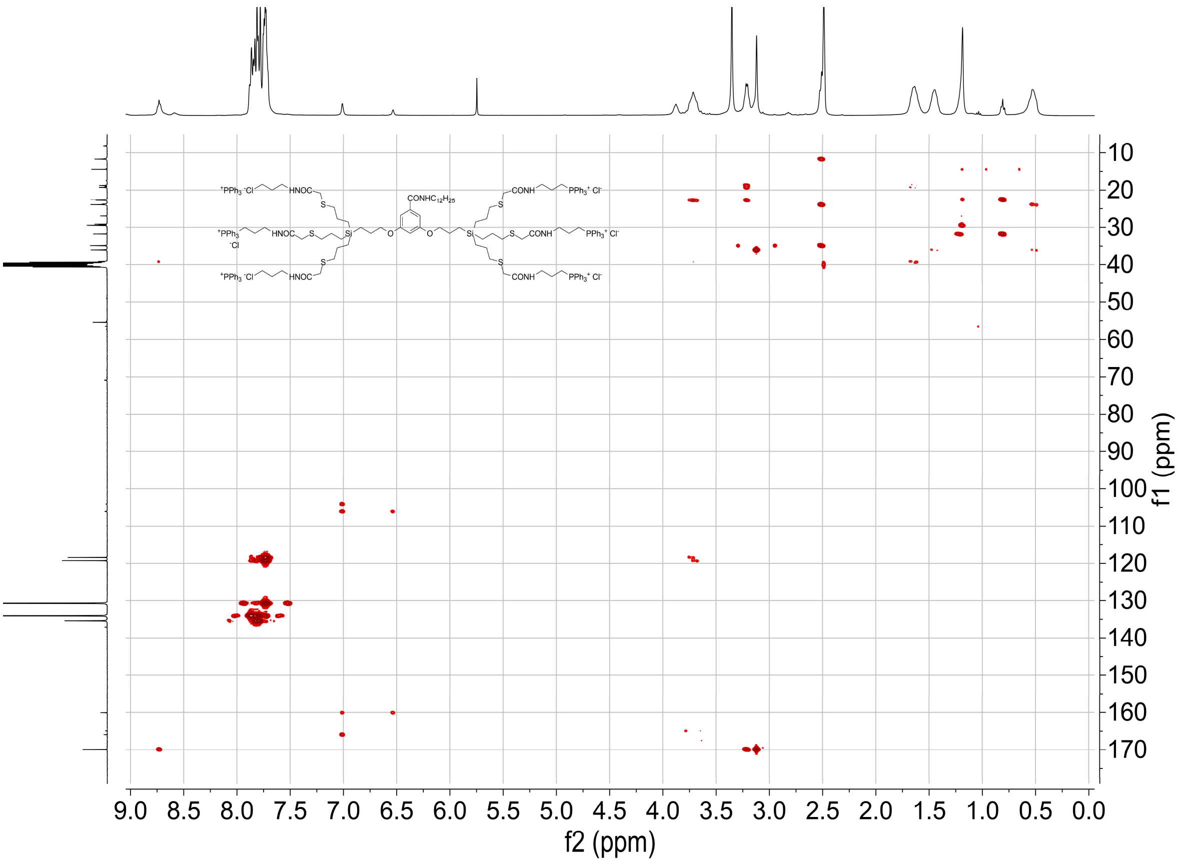

Figure S67. HMBC NMR (400 MHz, DMSO-d_6) of **DnP$_6$-1C$_{12}$** (**9a**).

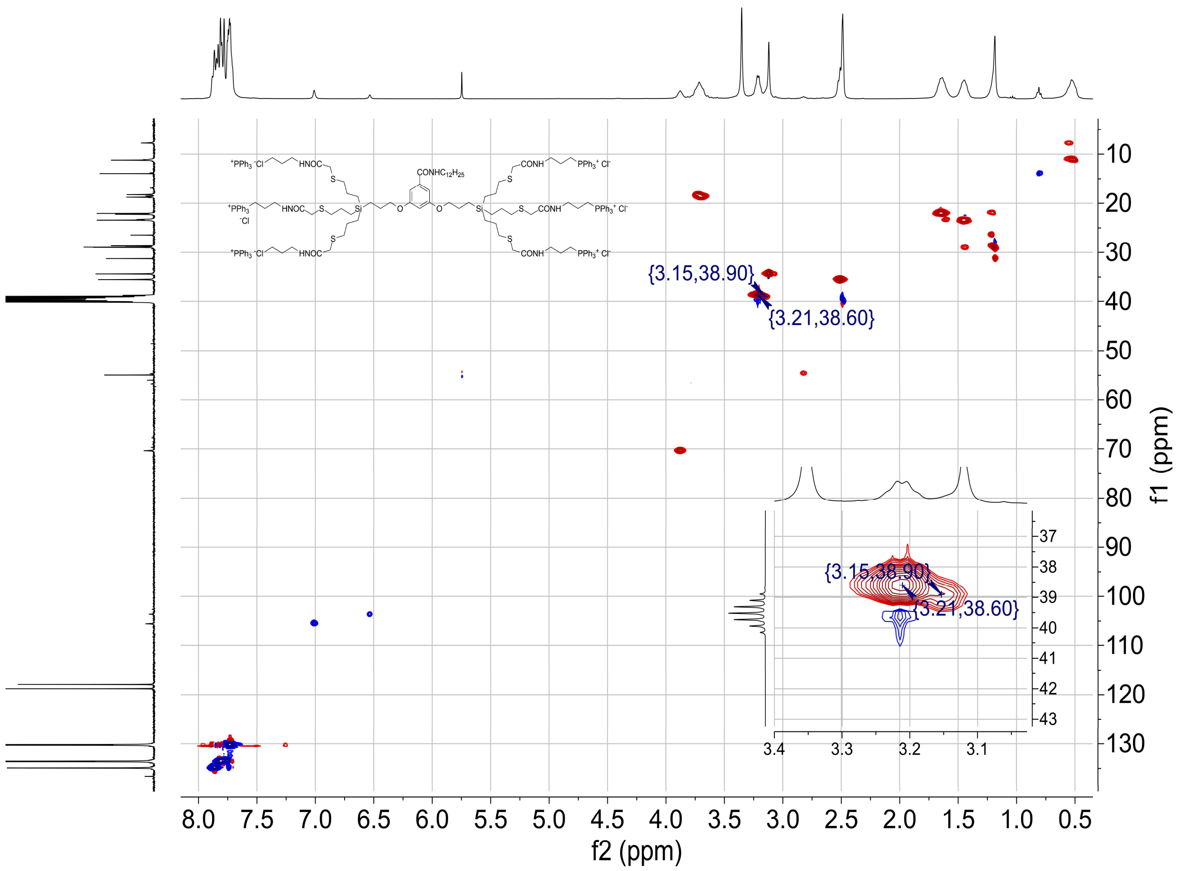

Figure S68. HSQC NMR (400 MHz, DMSO-d_6) of **DnP$_6$-1C$_{12}$** (**9a**).

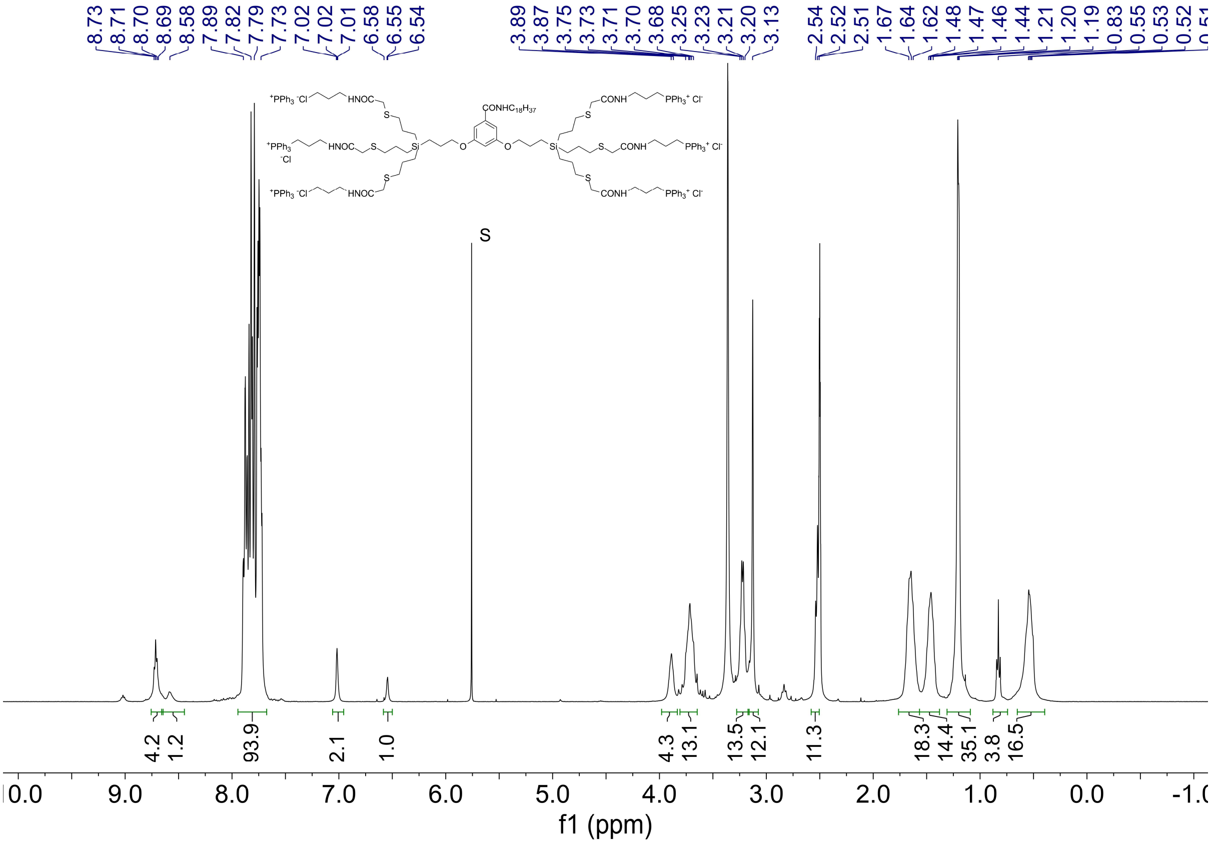

Figure S69. ^{1}H NMR (400 MHz, DMSO-d_6) of **DnP$_6$-1C$_{18}$** (**9b**).

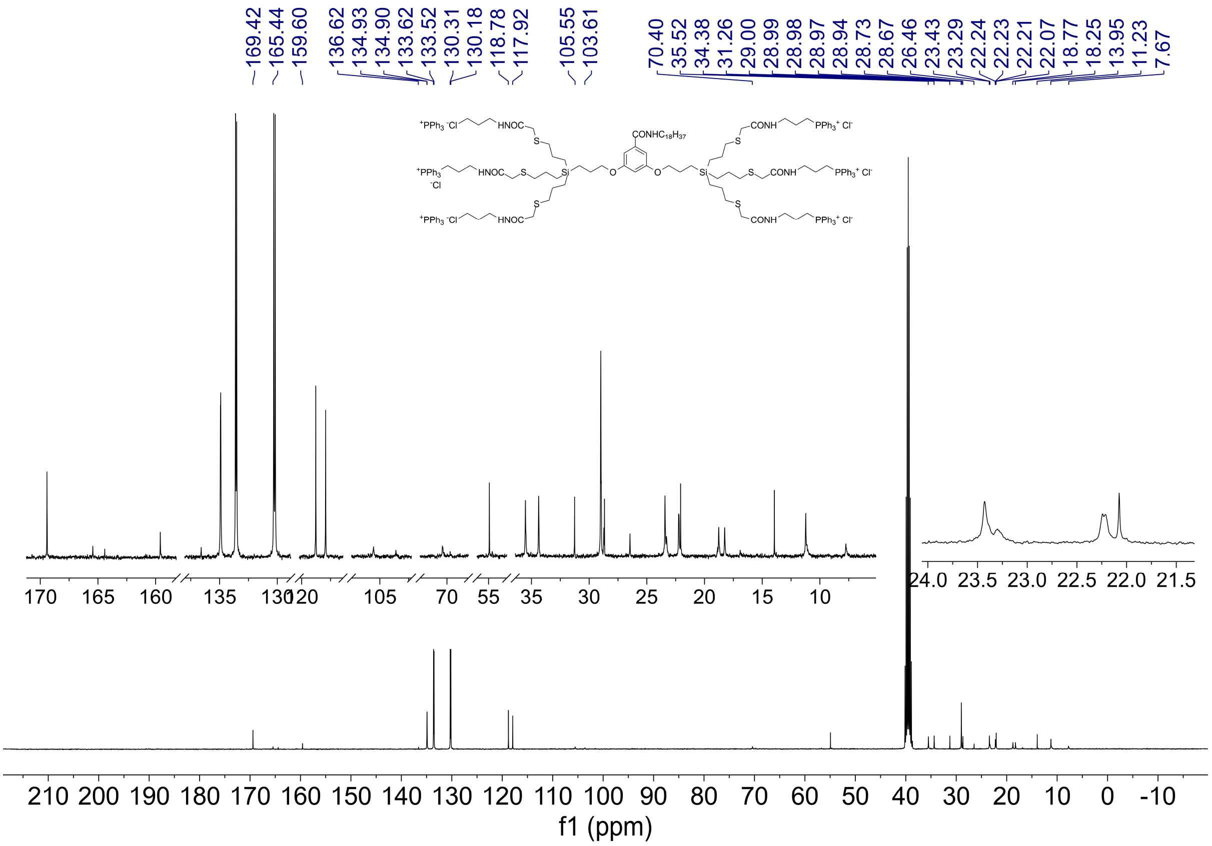

Figure S70. $^{13}C\{^1H\}$ NMR (400 MHz, DMSO-d_6) of **DnP$_6$-1C$_{18}$** (**9b**).

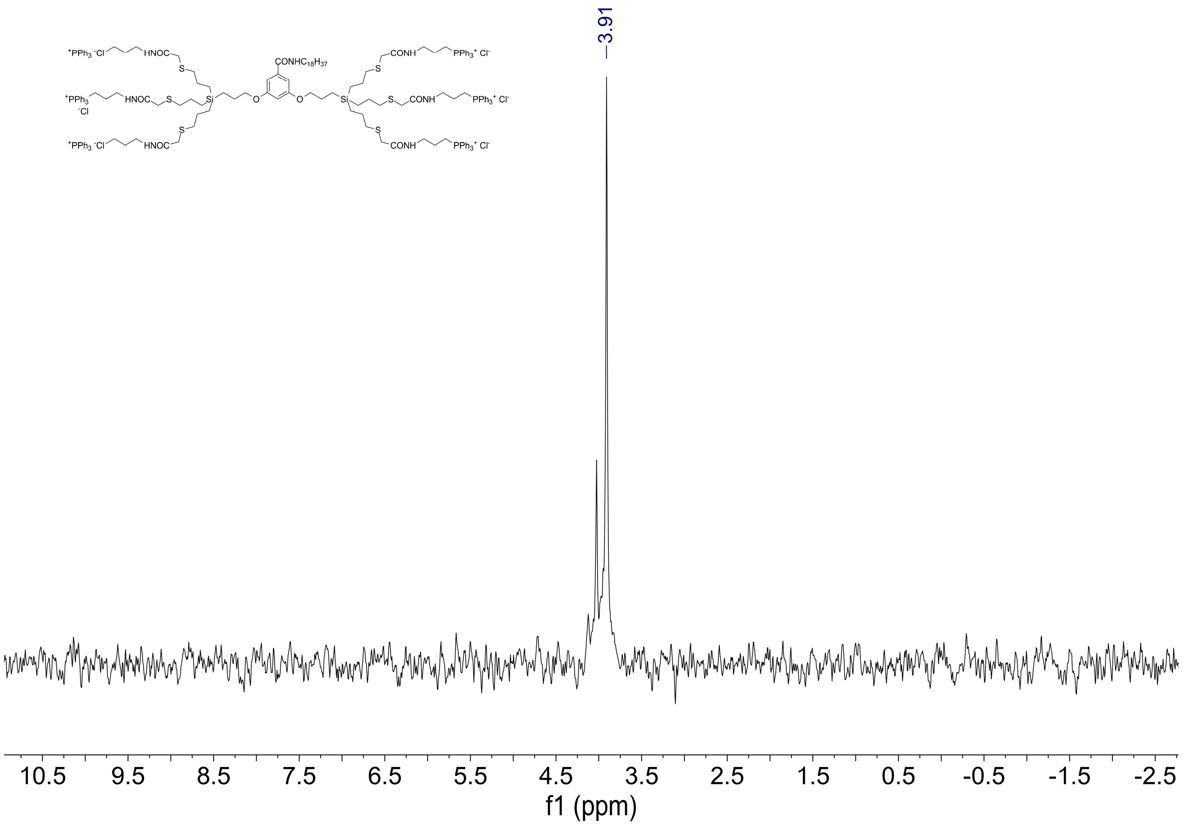

Figure S71. $^{29}Si\{^1H\}$ NMR (400 MHz, DMSO-d_6) of **DnP$_6$-1C$_{18}$** (**9b**).

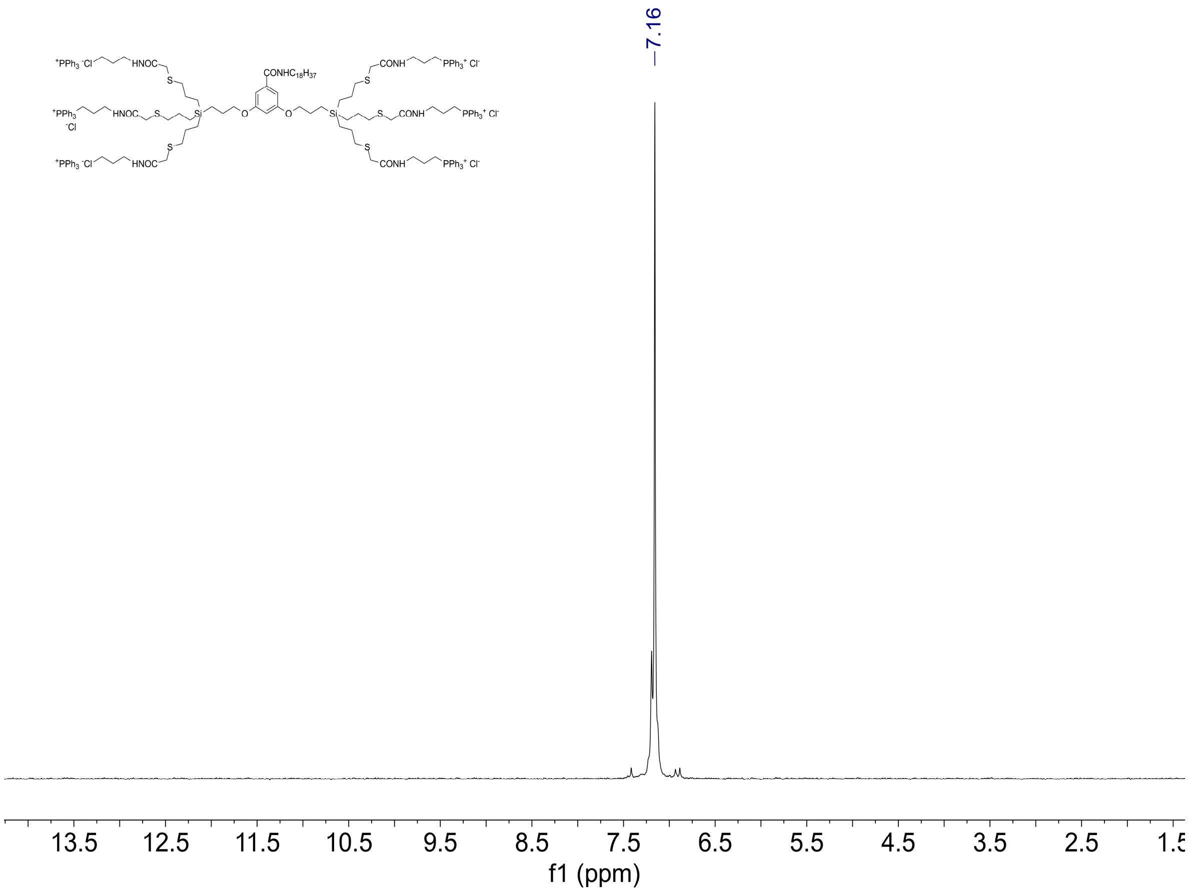

Figure S72. $^{31}P\{^{1}H\}$ NMR (400 MHz, DMSO-d_6) of **DnP$_6$-1C$_{18}$** (**9b**).

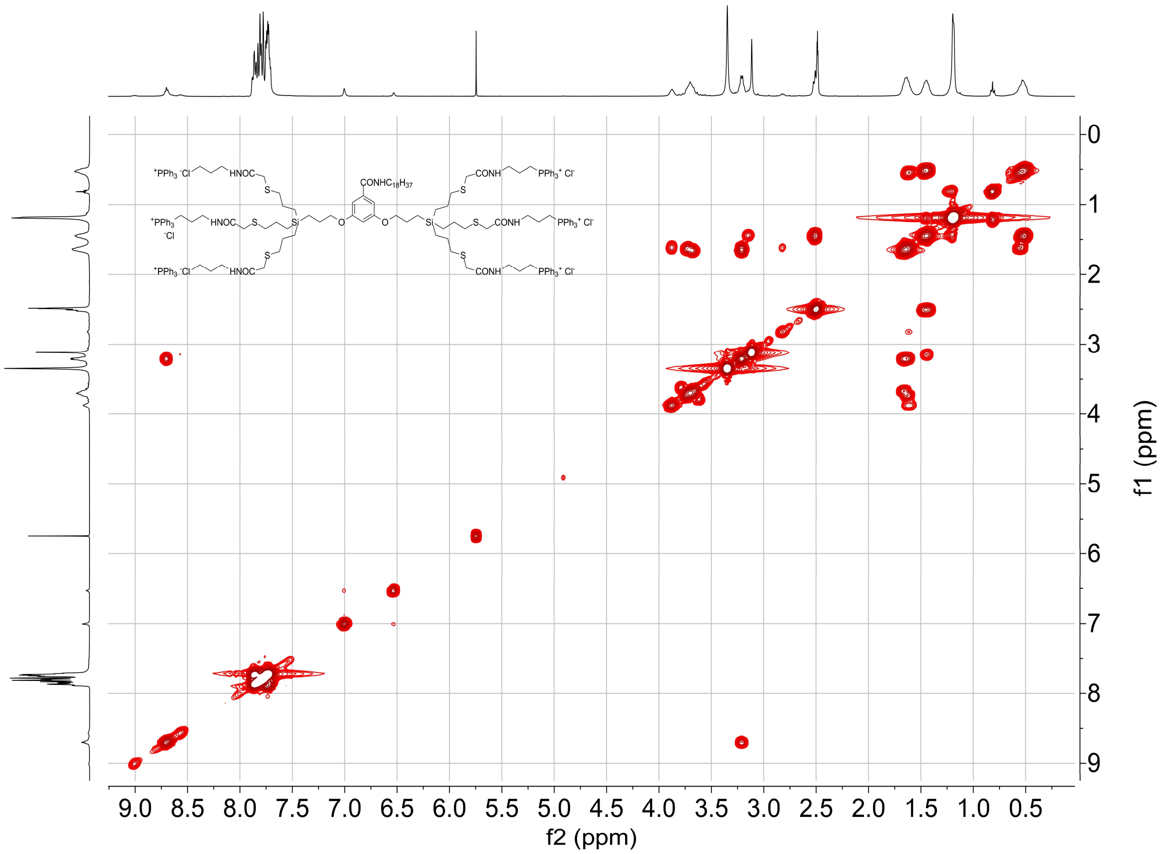

Figure S73. COSY NMR (400 MHz, DMSO-d_6) of **DnP$_6$-1C$_{18}$** (**9b**).

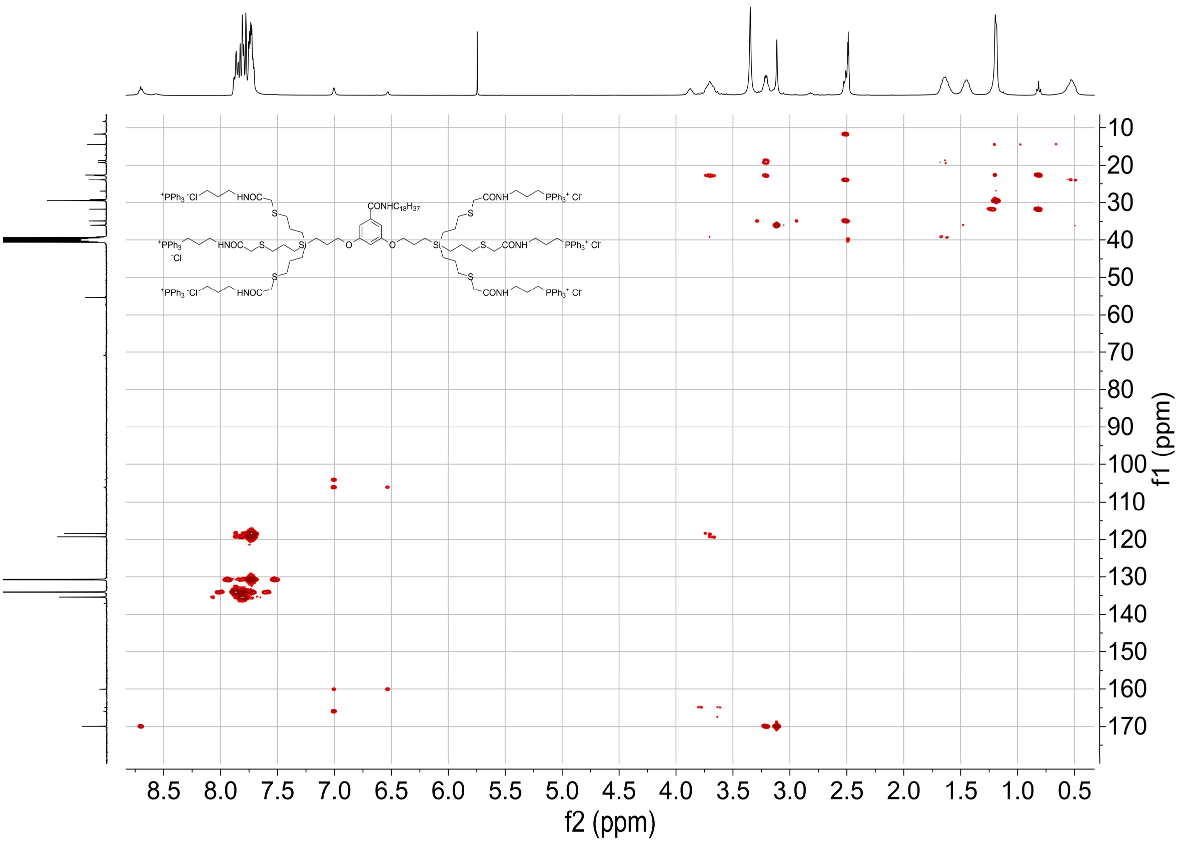

Figure S74. HMBC NMR (400 MHz, DMSO-d_6) of **DnP$_6$-1C$_{18}$ (9b)**.

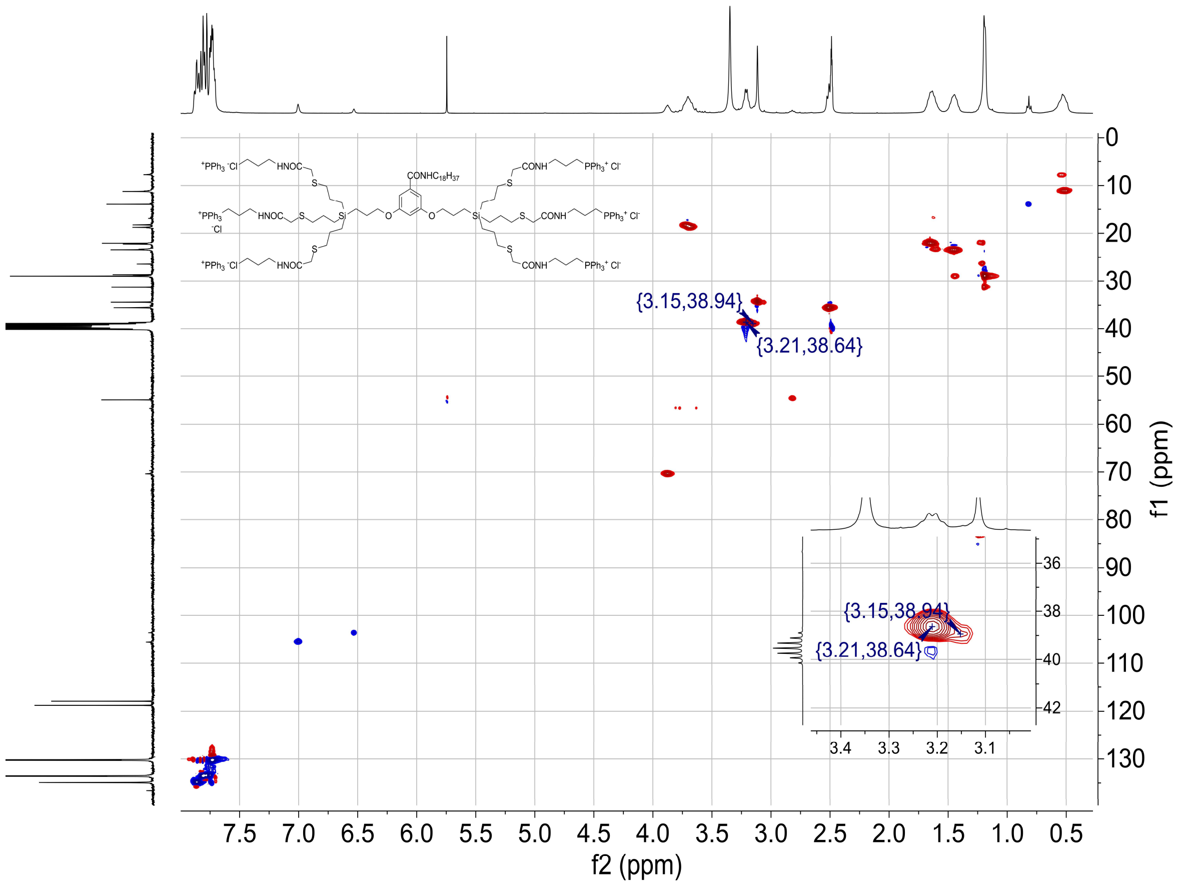

Figure S75. HSQC NMR (400 MHz, DMSO-d_6) of **DnP$_6$-1C$_{18}$ (9b)**.

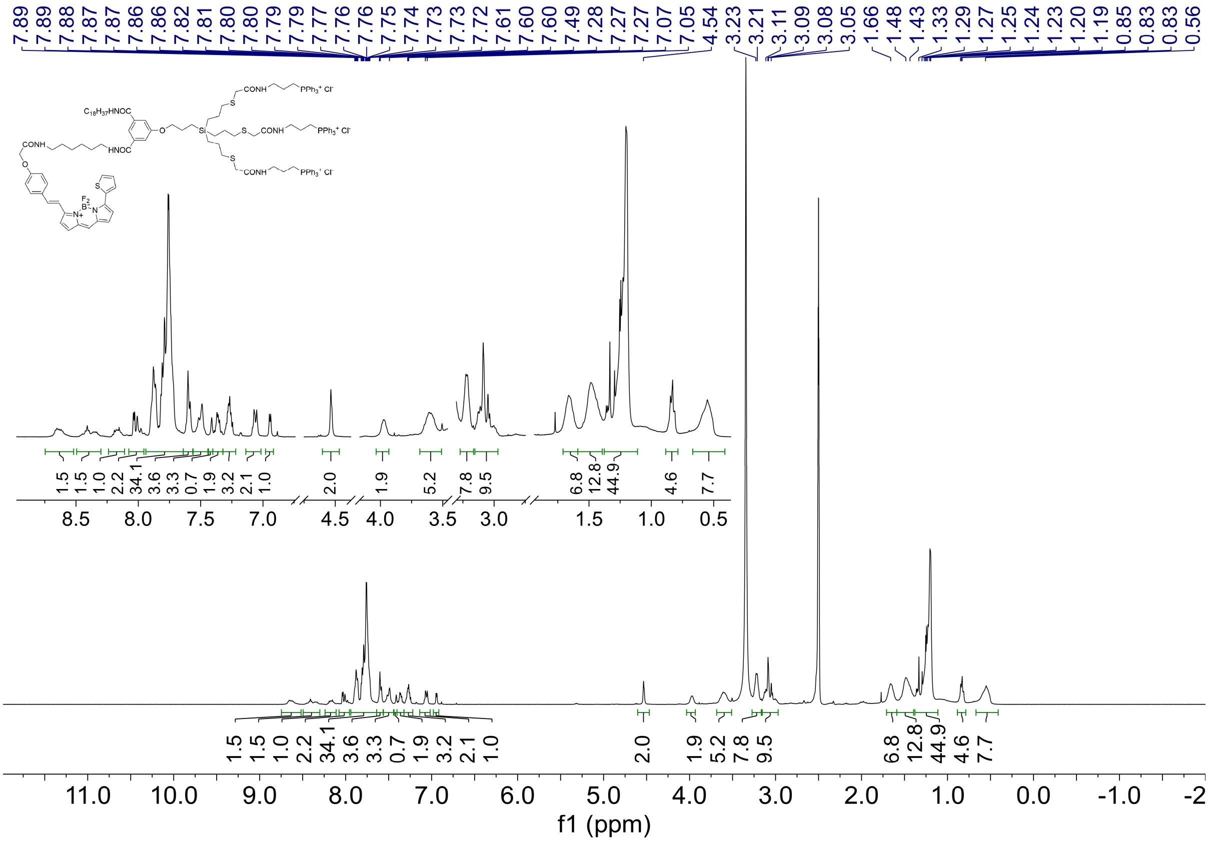

Figure S76. ^{1}H NMR (400 MHz, DMSO-d_6) of **DnP$_3$-1C$_{18}$/BDP** (**17**).

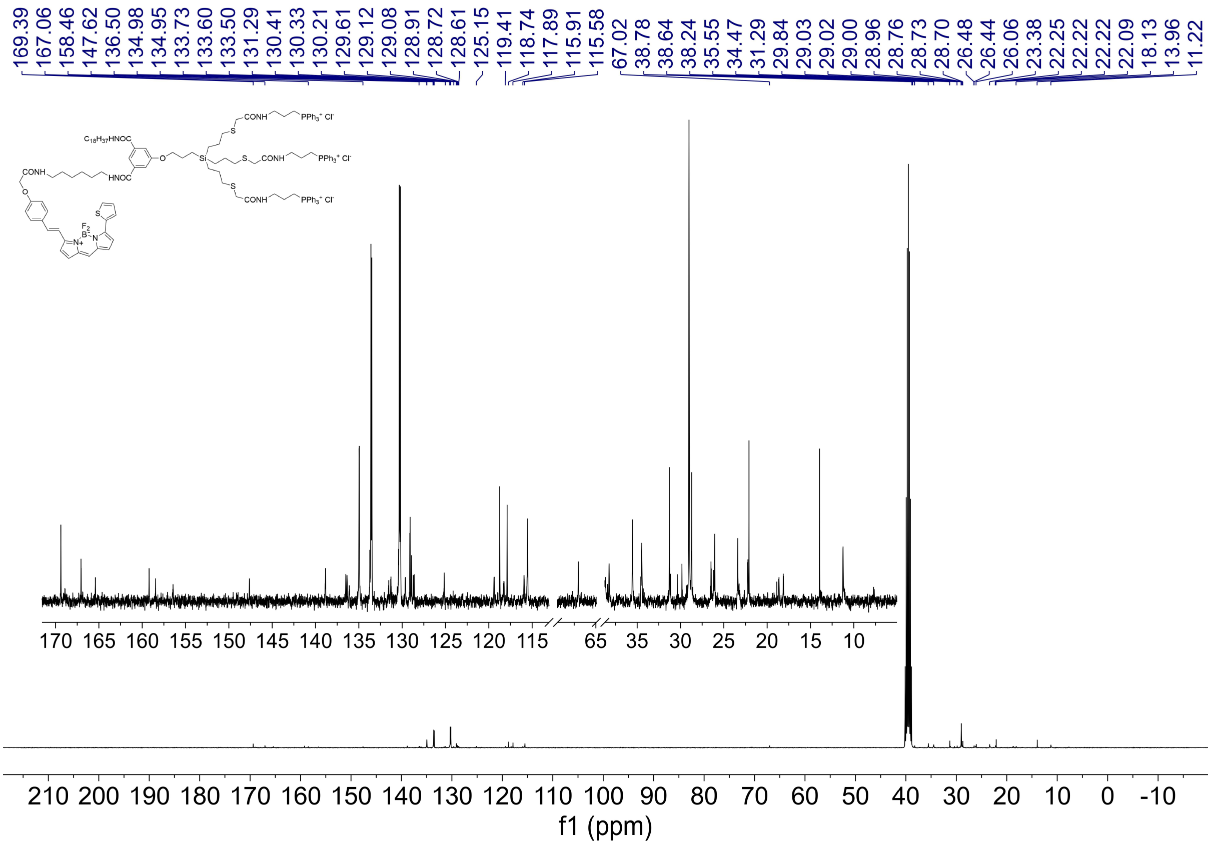

Figure S77. ^{13}C{^{1}H} NMR (400 MHz, DMSO-d_6) of **DnP$_3$-1C$_{18}$/BDP** (**17**).

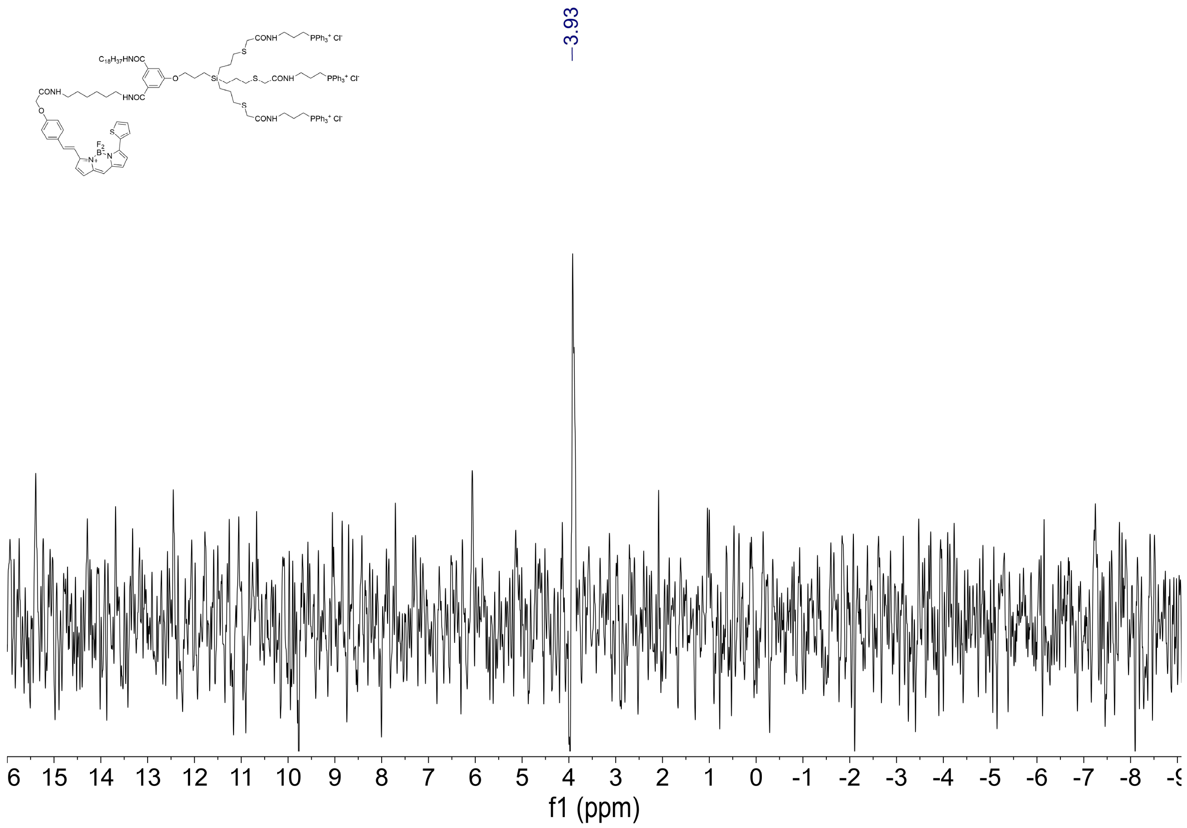

Figure S78. $^{29}Si\{^{1}H\}$ NMR (400 MHz, DMSO-d_6) of **DnP$_3$-1C$_{18}$/BDP** (**17**).

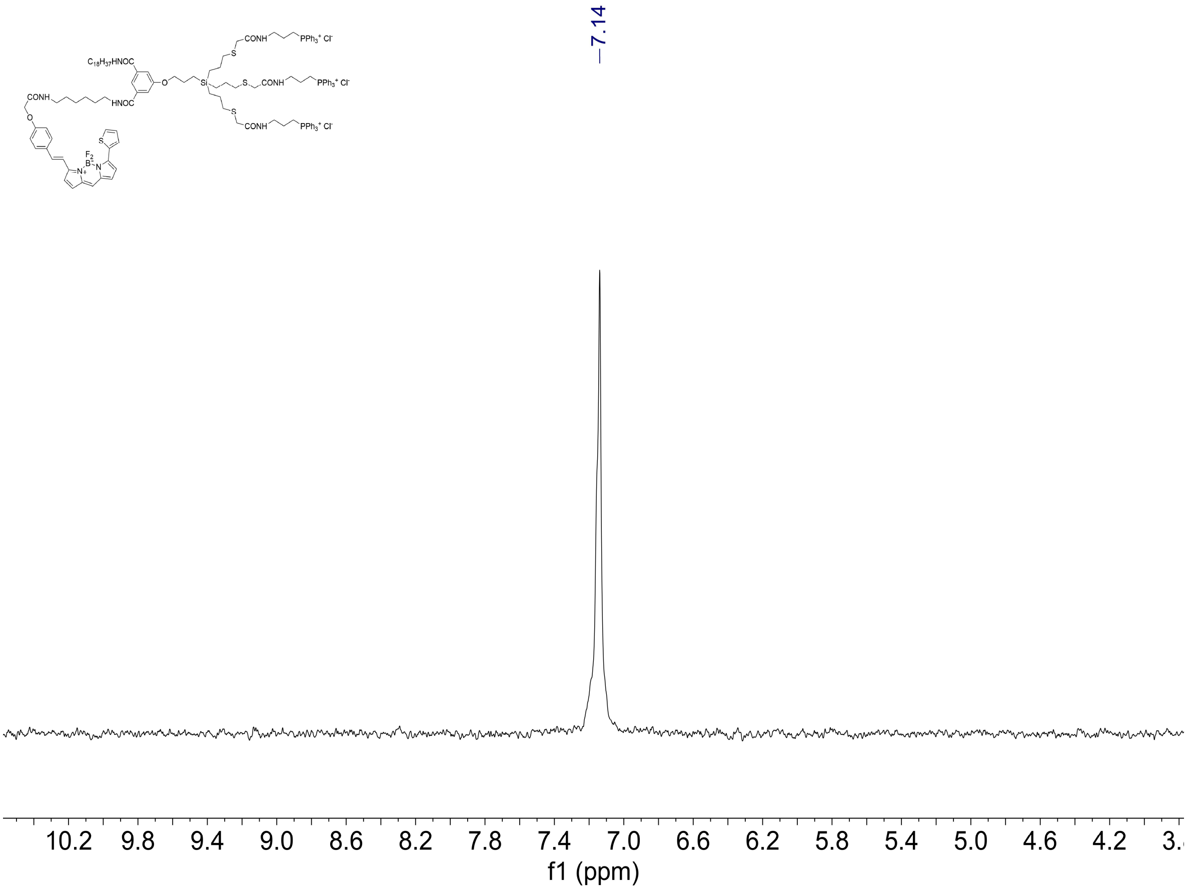

Figure S79. $^{31}P\{^{1}H\}$ NMR (400 MHz, DMSO-d_6) of **DnP$_3$-1C$_{18}$/BDP** (**17**).

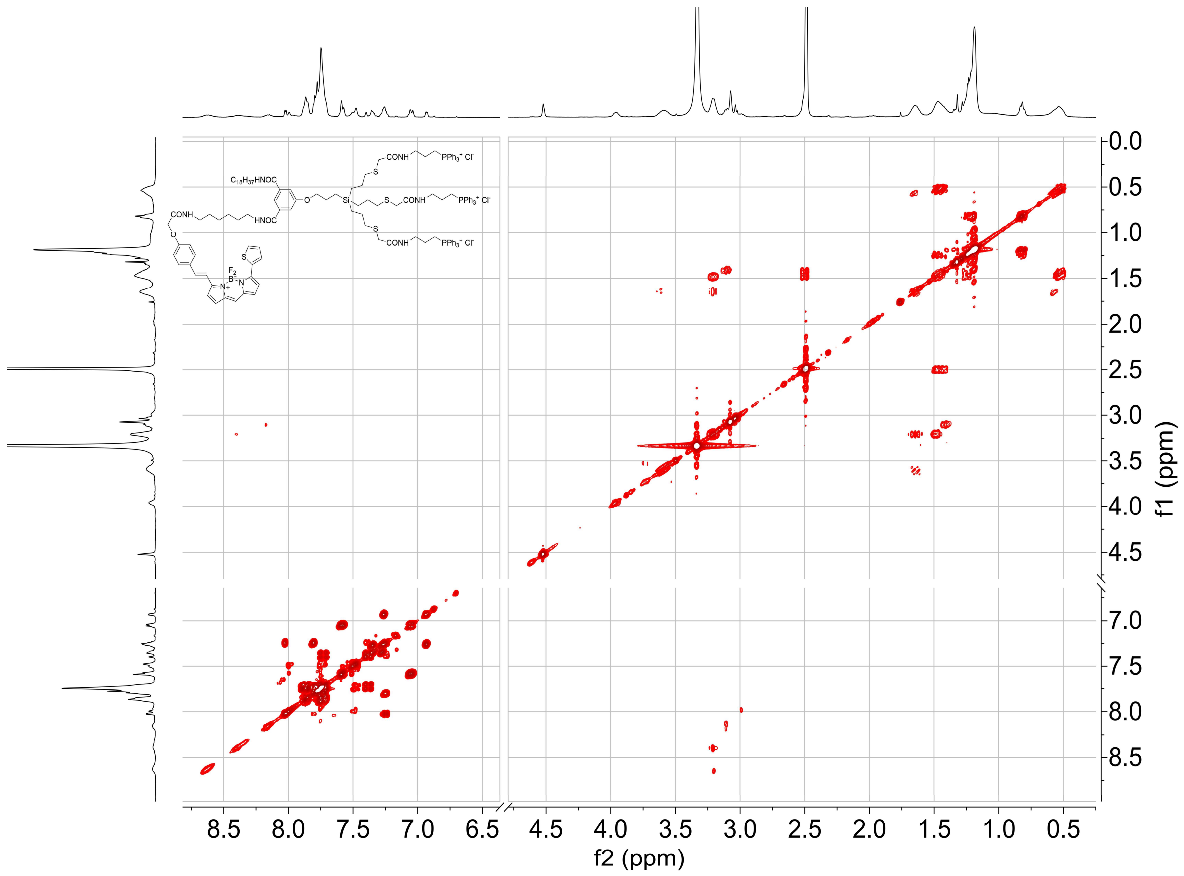

Figure S80. COSY NMR (400 MHz, DMSO-d_6) of **DnP$_3$-1C$_{18}$/BDP** (**17**).

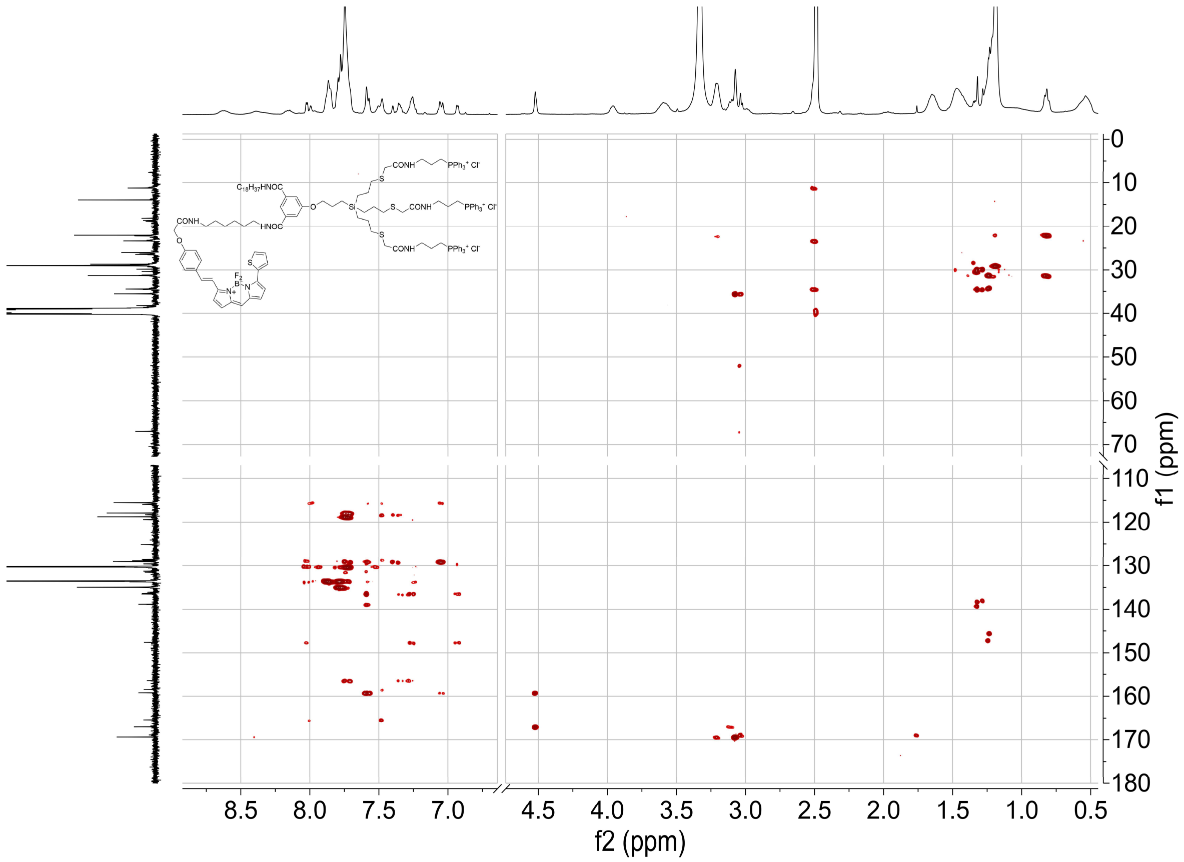

Figure S81. HMBC NMR (400 MHz, DMSO-d_6) of **DnP$_3$-1C$_{18}$/BDP** (**17**).

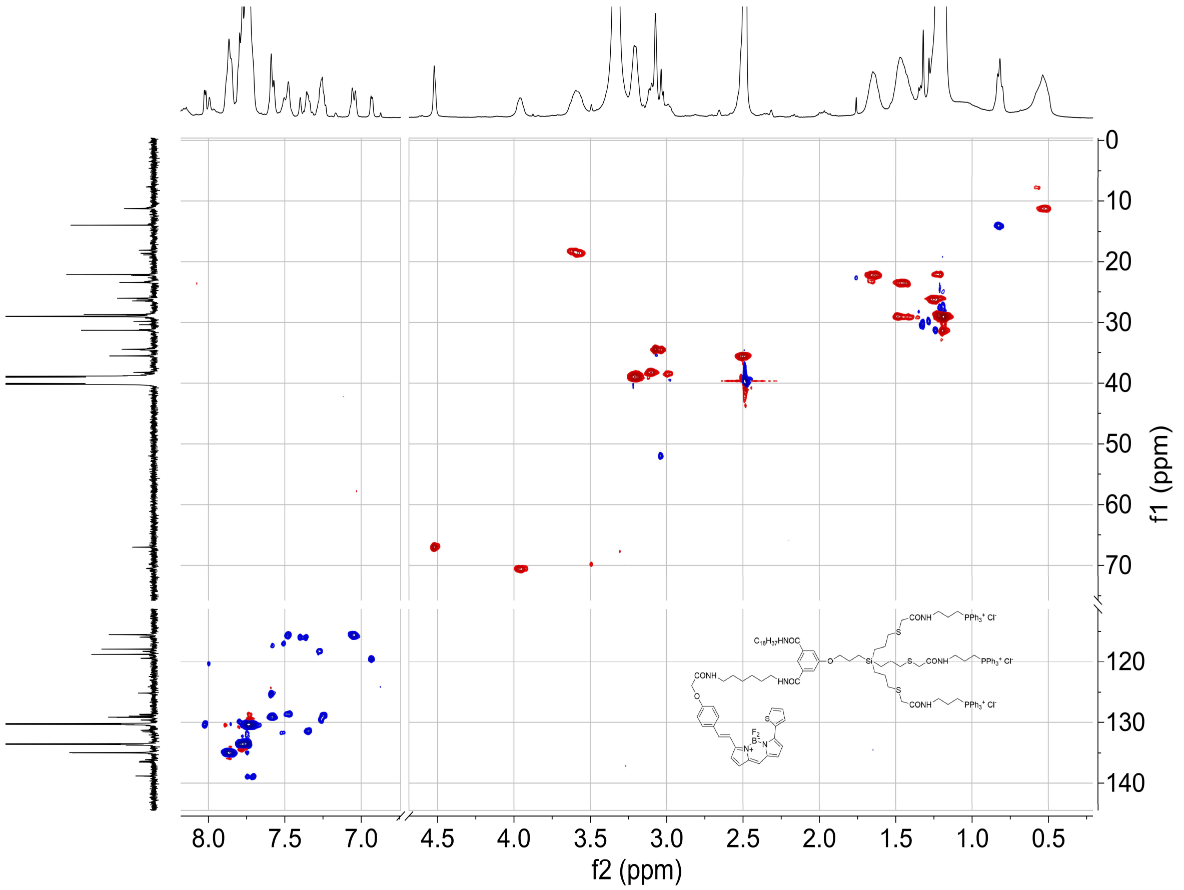

Figure S82. HSQC NMR (400 MHz, DMSO-d_6) of **DnP$_3$-1C$_{18}$/BDP** (**17**).

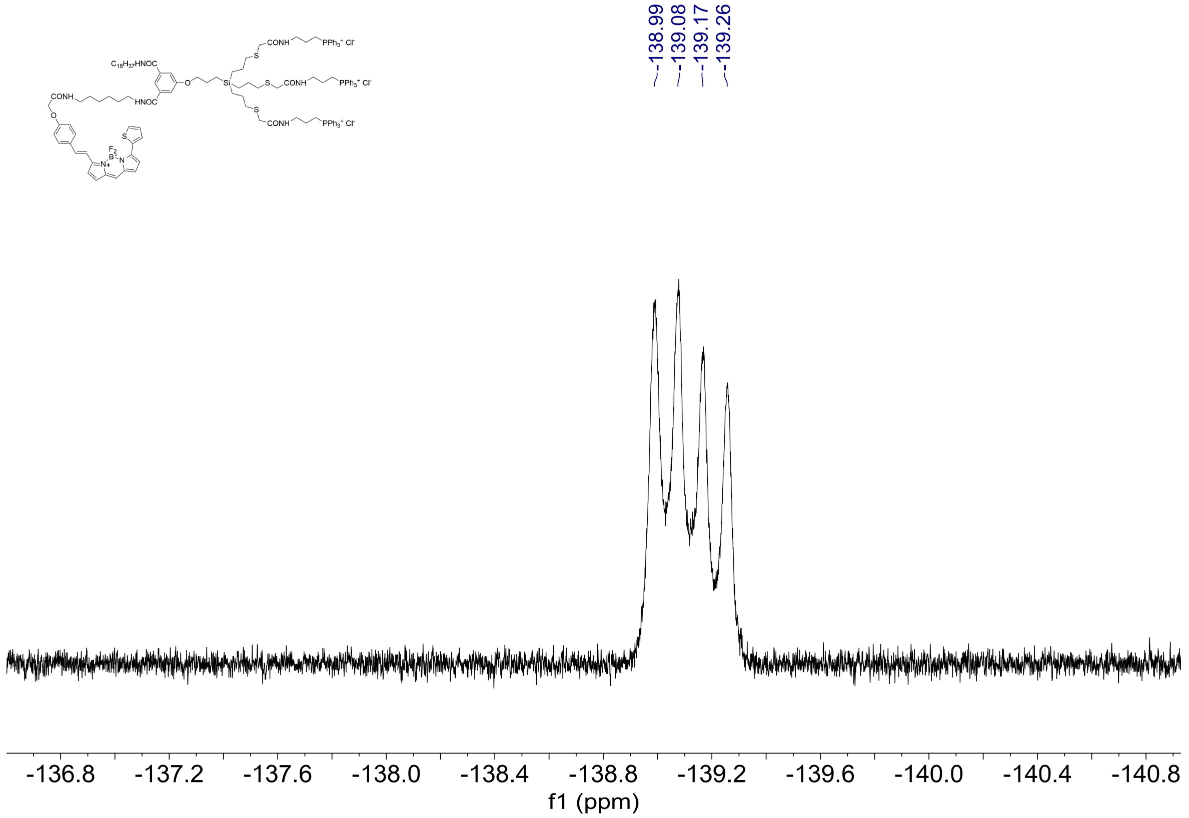

Figure S83. ^{19}F NMR (400 MHz, DMSO-d_6) of **DnP$_3$-1C$_{18}$/BDP** (**17**).

10. Mass spectra of amphiphilic dendrons

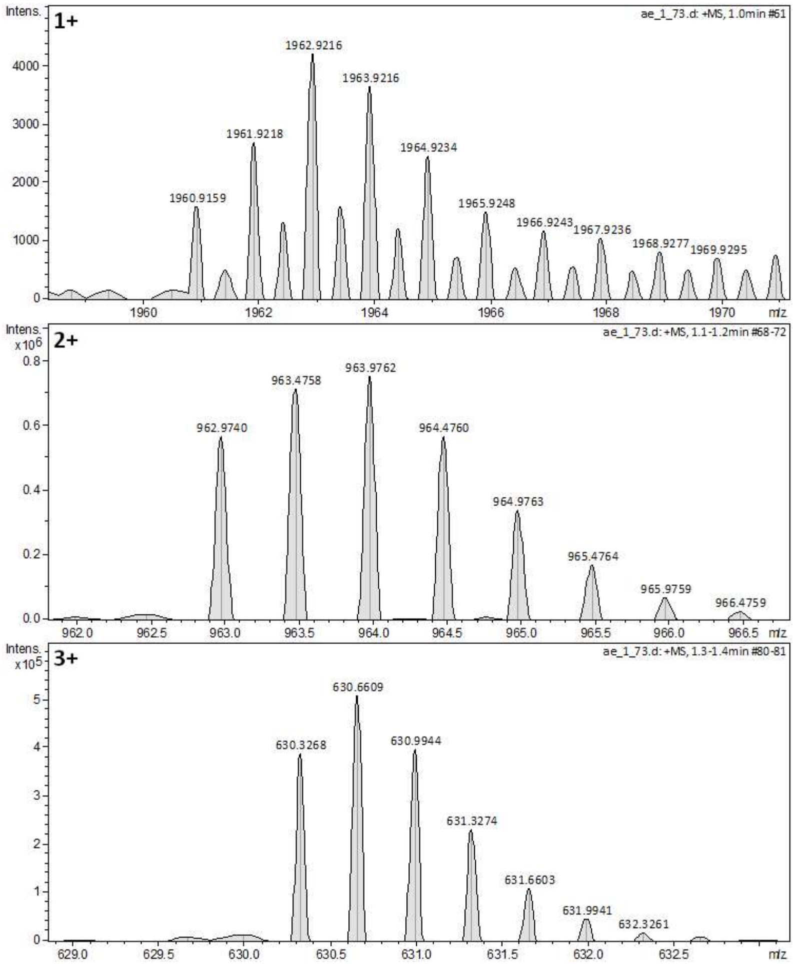

Figure S84. Sections of ESI MS spectrum of **DnP$_3$-2C$_{12}$** (**3a**), representing 1+, 2+ and 3+ ions.

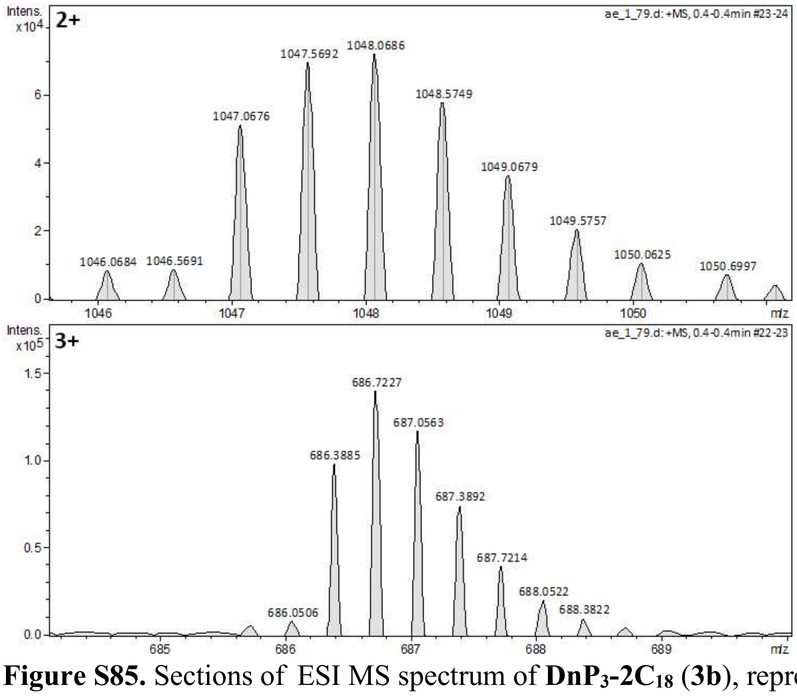

Figure S85. Sections of ESI MS spectrum of **DnP$_3$-2C$_{18}$** (**3b**), representing 2+ and 3+ ions; 1+ ions were outside the HRMS range (m/z > 2000).

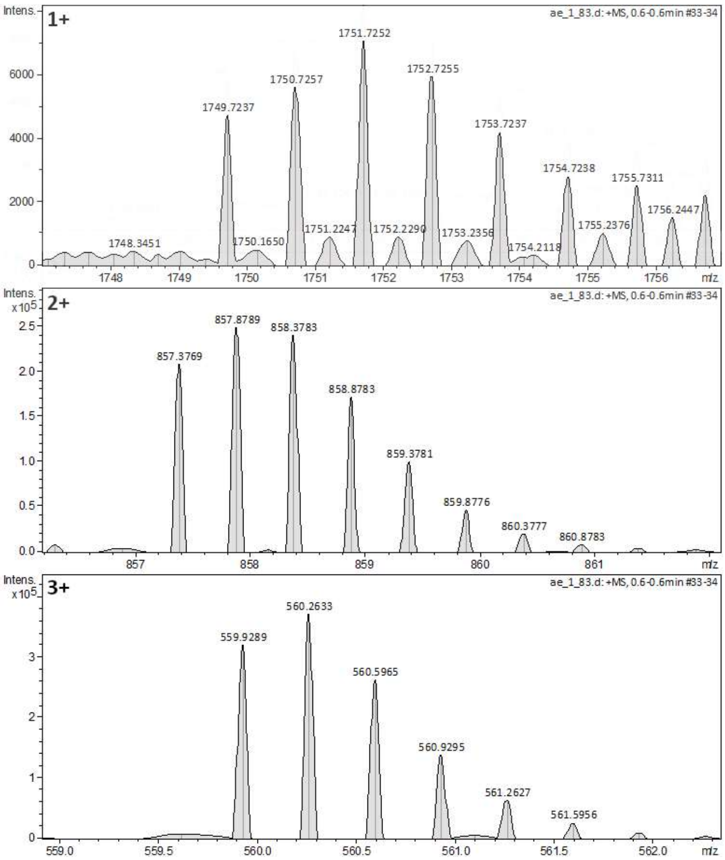

Figure S86. Sections of ESI MS spectrum of **DnP$_3$-1C$_{12}$** (**6a**), representing 1+, 2+ and 3+ ions.

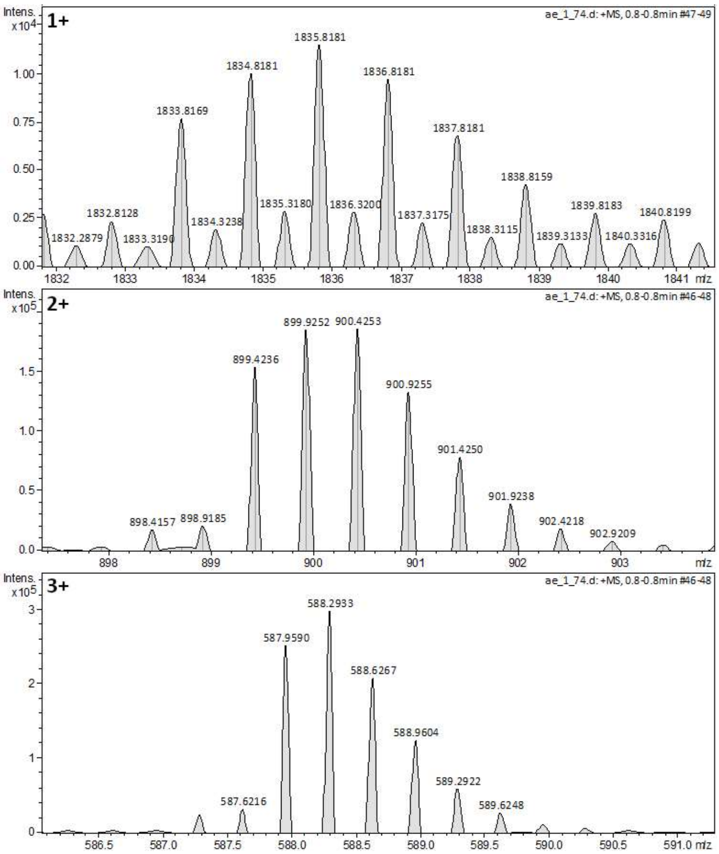

Figure S87. Sections of ESI MS spectrum of **DnP$_3$-1C$_{18}$** (**6b**), representing 1+, 2+ and 3+ ions.

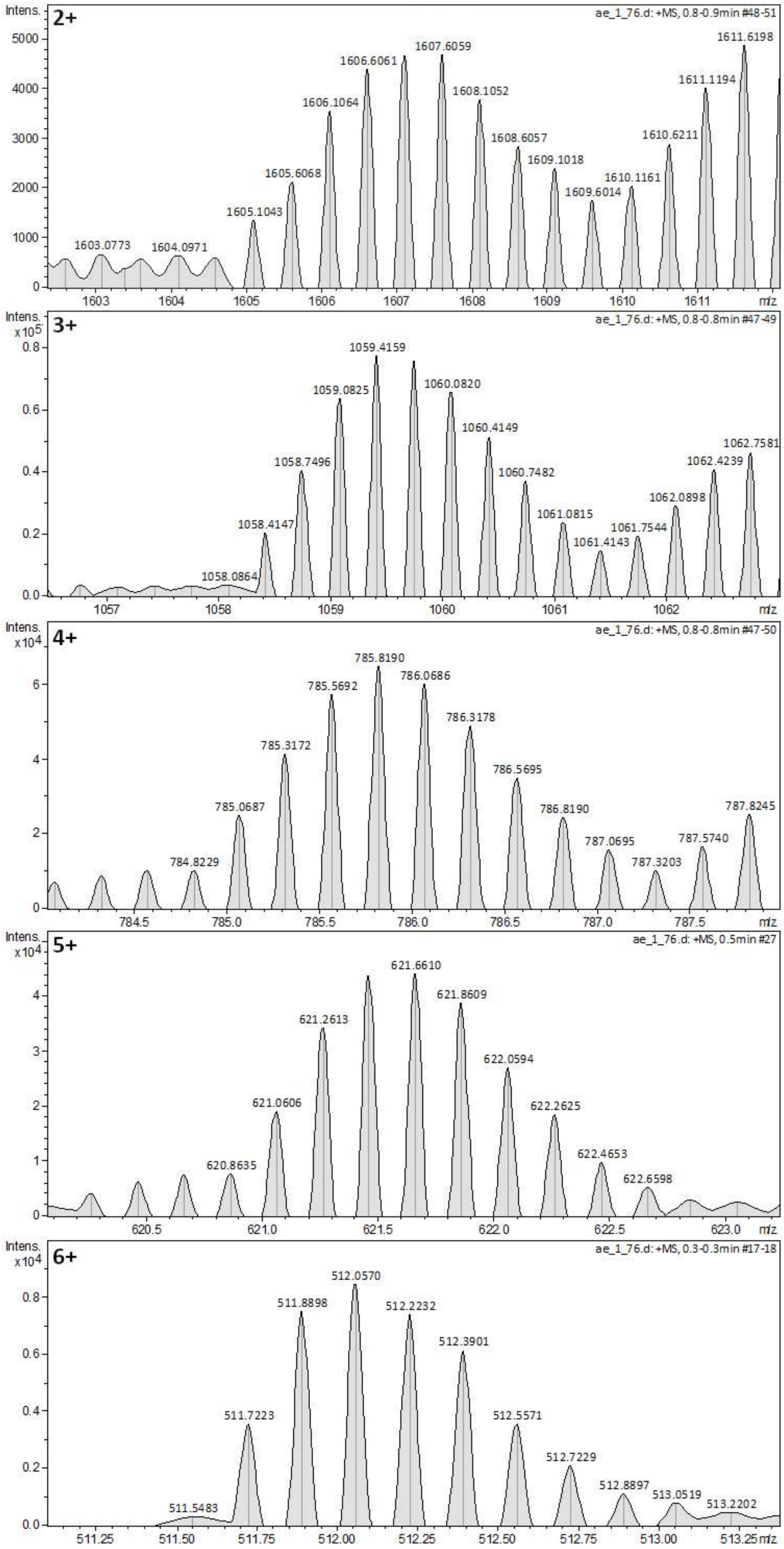

Figure S88. Sections of ESI MS spectrum of **DnP$_6$-1C$_{12}$** (**9a**), representing 2+, 3+, 4+, 5+ and 6+ ions; 1+ ions were outside the HRMS range (m/z > 2000). Peaks differing by +10.03/z from the expected values are due to ion exchange of chloride anion with formiate from the calibration mix.

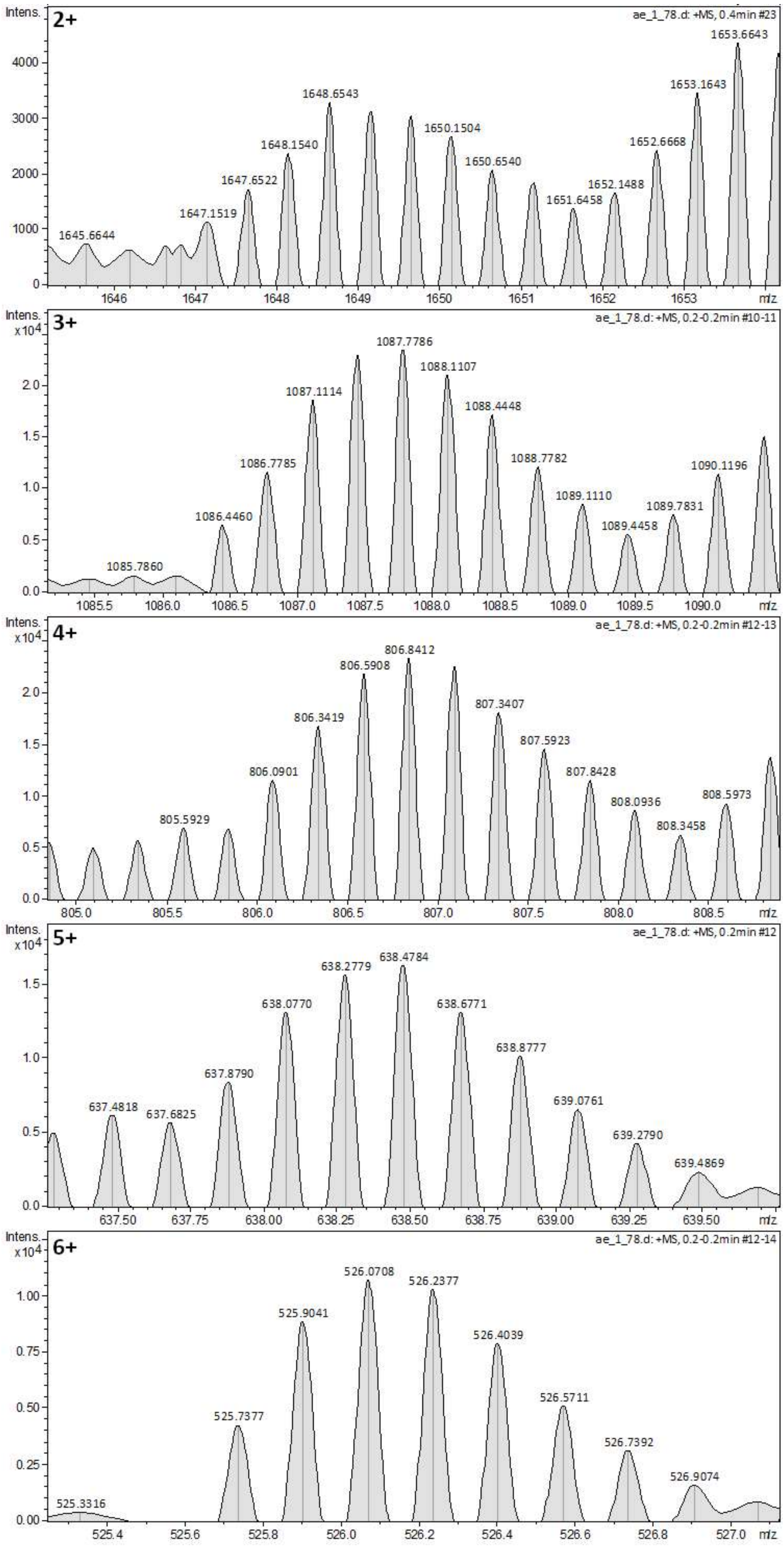

Figure S89. Sections of ESI MS spectrum of **DnP$_6$-1C$_{18}$** (**9b**), representing 2+, 3+, 4+, 5+ and 6+ ions; 1+ ions were outside the HRMS range (m/z > 2000). Peaks differing by +10.03/z from the expected values are due to ion exchange of chloride anion with formiate from the calibration mix.

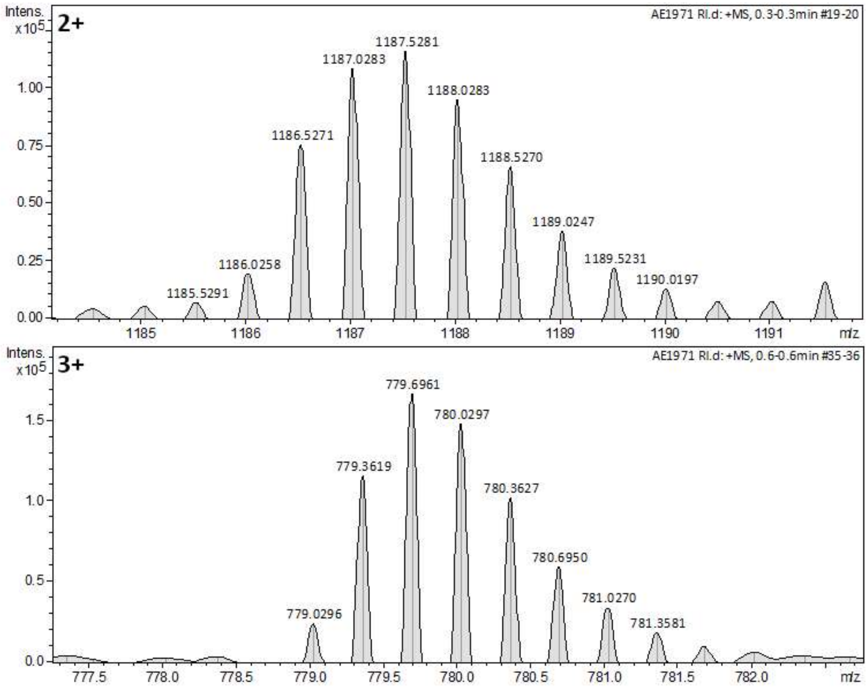

Figure S90. Sections of ESI MS spectrum of **DnP$_3$-1C$_{18}$/BPD** (**17**), representing 2+ and 3+ ions; 1+ ions were outside the HRMS range (m/z > 2000).
